# Identification and Validation of 3‑Cyano-Quinoline Ligands Targeting Integrin-Linked Kinase (ILK)

**DOI:** 10.1021/acs.jmedchem.5c03773

**Published:** 2026-05-25

**Authors:** Francesco Aleksy Greco, Kamal Rayees Abdul Azeez, Marko Mitrović, Saran Aswathaman Sivashanmugam, Martin Peter Schwalm, Franziska Preuss, Deep Chatterjee, Viktoria Morasch, Sebastian Mathea, Thomas Hanke, Susanne Müller, Stefan Knapp

**Affiliations:** † Institute of Pharmaceutical Chemistry, 9173 Goethe University Frankfurt, Max-von-Laue-Str. 9, Frankfurt am Main 60438, Germany; ‡ Structural Genomics Consortium (SGC), Buchmann Institute for Molecular Life Sciences (BMLS), Max-von-Laue-Str. 15, Frankfurt am Main 60438, Germany; § German Cancer Research Center (DKFZ), Im Neuenheimer Feld 280, Heidelberg 69120, Germany

## Abstract

Integrin-linked kinase (ILK) is a pseudokinase that directly interacts with β-integrins and plays a pivotal role in regulating focal adhesion function. ILK has been implicated in the development of various diseases, particularly cancer. However, currently, no validated ligands for ILK have been reported. Here, we describe the identification of 3-cyano-quinolines that potently bind to ILK (K_D_ = ∼250 nM), and crystallographic studies revealed a type I binding mode. A medicinal chemistry campaign exploring structure–activity relationships (SAR) using a robust parallel synthesis approach provided comprehensive SAR and identified regions amenable to modification. In addition, we demonstrated that the optimized 3-cyano-quinoline **1** (DHP) modulates actin cytoskeletal dynamics. This work highlights the first validated ILK ligands and establishes a foundation for future translational efforts, such as the development of selective PROTACs targeting ILK for degradation by the ubiquitin system.

## Graphical abstract

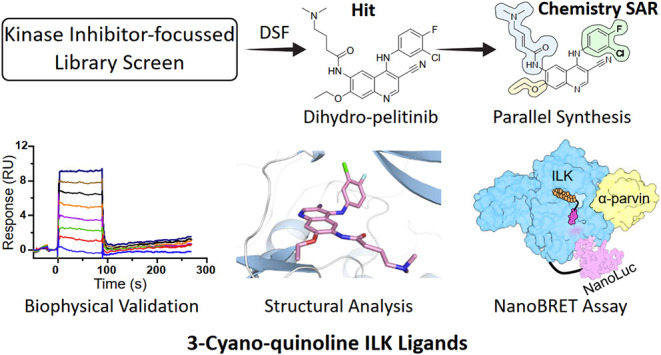

## Introduction

ILK is a catalytically inactive pseudokinase localized at focal adhesions, where it functions as a key scaffold regulating integrin signaling and actin cytoskeletal architecture. Since its discovery,^1^ understanding of ILK structure and function has continually evolved. Until 2009, ILK was believed to be a catalytically active kinase, with the cytoplasmic tails of β1 integrins, glycogen synthase kinase-3β (GSK-3β), and AKT proposed as its main phosphorylation targets.^1–3^ However, X-ray crystal structures and biochemical studies later demonstrated that ILK is an ATP-cation binding pseudokinase, and that the previously proposed activity was likely caused by contaminant kinases that copurified with ILK.^4,5^ Hence, it is now widely accepted that ILK acts as a pseudokinase and scaffolding protein, engaging the cytoplasmic tails of activated β1 and β3 integrins but lacking intrinsic phosphorylation activity. Furthermore, ILK predominantly exists as a ternary complex with its interaction partners, particularly interesting new cysteine-histidine-rich protein (PINCH) and α-parvin, forming the IPP complex.^6–9^ Importantly, this complex connects activated β1 and β3 integrins to the actin cytoskeleton, facilitating F-actin bundling through the WH2 domains of PINCH and α-parvin,^10^ enabling mechano-signal transduction. These events, in turn, drive cell spreading, migration, and stress fiber formation, as well as broader cellular functions including proliferation, growth, and survival through cross-talk with multiple signaling pathways such as those involving the receptor tyrosine kinases.^11–13^ ILK and its key cellular interaction partners are illustrated in Figure 1A and B.

**Figure 1:**
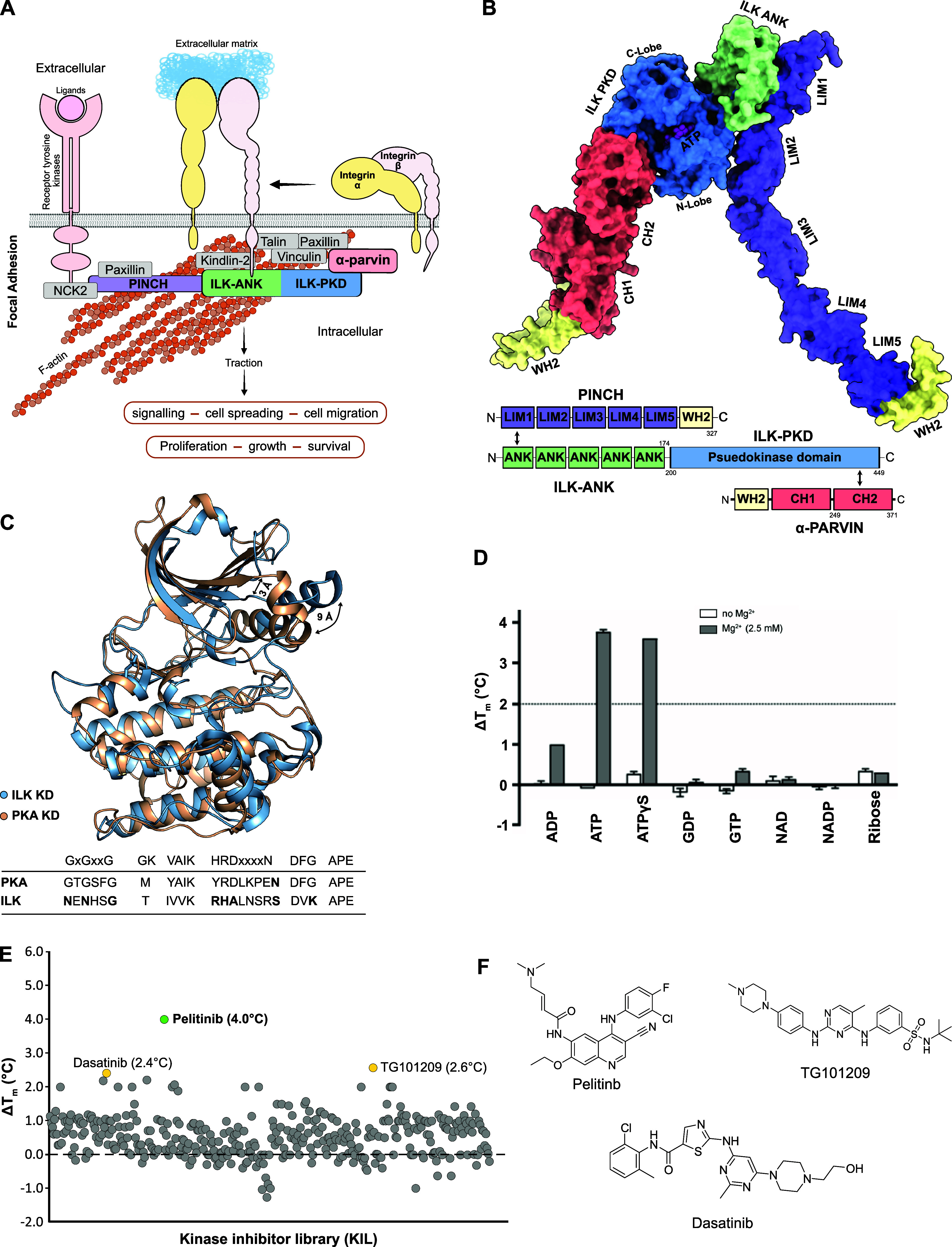
ILK pseudokinase at focal adhesions and screening data (A) ILK at focal adhesions. The key interaction landscape at focal adhesions that contributes to mechano-signaling is highlighted and labeled. (B) IPP ternary complex assembly. Top: The IPP complex was modeled using PDB: 3KMW, 4HI8, and full-length AlphaFold structures. The ILK kinase domain is bound to ATP. Bottom: IPP domain architectures. The LIM1 domain of PINCH interacts with the N-terminal ankyrin repeat domain 1 (ANK1) of ILK, while the CH2 domain of α-parvin interacts with the ILK pseudokinase domain (PKD). Both PINCH and α-parvin contain a conserved (winged helix) WH2 domain, which mediates mechanical force generation for F-actin bundling and cytoskeletal dynamics. (C) Top: Ribbon diagram of the ILK kinase domain. The ILK kinase domain (PDB: 3KMW) structure was superimposed on PKA (PDB: 1ATP) with an RMSD of 2 Å considering main chain atoms, confirming overall fold conservation of the ILK kinase domain. The main observable difference was observed in the β3−αC elements, where the αC helix was displaced by ∼9 Å at the front end. The absence of the ILK αC E238:β3 K220 salt bridge likely caused this displacement. Bottom: Key conserved kinase motifs were compared with PKA. Noncanonical residues in ILK are highlighted in bold. The nonconserved residues are highlighted in Figure S1. (D) Nucleotide screening. *ΔT_m_* values of different nucleotides were measured against the ILK−α-parvin complex. Adenosine-containing nucleotides with terminal phosphate groups (ATP, ATPγS, and ADP) produced the most substantial stabilization, but only in the presence of the cofactor Mg^2+^. (E) Initial high-throughput screen (HTS). An in-house kinase inhibitor-focused library containing ∼380 compounds was screened against the ILK−α-parvin complex using DSF assay. *ΔT_m_* values (°C) are represented as a dot plot. Top hits are highlighted in yellow (>2 °C) or green (≥4 °C). Screening data and the library of compounds are provided in the Supporting Information. (F) ILK:α-parvin stabilizing compounds. Lewis structures of the three top hits inducing the most substantial *ΔT_m_* shifts in the DSF assay are shown. Pelitinib features a 6,7-disubstituted 4-amino-3-carbonitrile quinoline scaffold, whereas TG101209 and dasatinib both contain a disubstituted pyrimidine core.

Modulating and disrupting the components of the IPP complex, in particular ILK and α-parvin, has been shown to directly impact the IPP complex and, consequently, focal adhesion-related functions.^14,15^ This highlights a promising strategy for targeting ILK in malignancies that rely on these adhesion-mediated functions. The ATP-binding pocket serves as a key regulatory control point for the stability and function of the IPP complex. Mutations within this site have been shown to disrupt IPP-mediated functions.^10,16^ Therefore, targeting the ATP-binding pocket with small-molecule binders that modulate ILK conformation dynamics and IPP complex–dependent functions represents an attractive therapeutic avenue for various cancers^17,18^ and chronic kidney disease.^19^ Currently, only a few small-molecule ligands of ILK have been reported, notably Cpd 22 (ILK-IN-2),^19–21^ QLT0267,^22^ EN300-6740186 (EN300)^23^ and a patented ligand Csbl-1e (US11524945B2), all of which feature distinct chemical scaffolds. However, direct biophysical on target binding to ILK has not been demonstrated for any of the currently reported compounds. It is plausible that they act downstream of ILK, with reported cellular effects resulting from on-pathway mechanisms rather than direct on-target activity. Thus, a comprehensively characterized ILK ligand would provide a valuable tool for investigating ILK biology at focal adhesions and associated functions.

Here, we targeted ILK using differential scanning fluorimetry (DSF) to screen a kinase inhibitor–focused library, which identified pelitinib as an initial hit and enabled the design and synthesis of a series of cyanoquinoline-containing compounds. The medicinal chemistry campaign using a robust parallel synthesis approach provided insights into the structure–activity relationships, highlighting key regions that are both sensitive and amenable to modifications. Biophysical binding assays (SPR) revealed binding affinities around 200 nM for the best ligands of this series. Finally, protein crystallography provided insights into the binding mode of this scaffold and a structural template for future rational design approaches.

## Results

### Discovery of Small-Molecule Kinase Domain Binders of ILK

ILK adopts a conserved kinase fold, but it lacks essential catalytic residues required for phosphoryl transfer. Its pseudoactive site contains degenerate motifs, including an altered G-loop, HRD motif, and a DVK motif in place of the canonical DFG. The DVK lysine (Lys341), the β3-strand lysine (Lys220), and a bound Mg^2+^ ion stabilize the ATP phosphates and maintain structural integrity. Notably, the canonical β3 Lys220−αC Glu238 salt bridge is not conserved, and the αC helix is pushed out (Figures 1C and S1).

Since ILK is an ATP-cation-binding pseudokinase stabilized by the Calponin Homology 2 (CH2) domain of α-parvin, we first confirmed nucleotide binding to the ILK kinase domain by DSF screening using an array of structurally diverse nucleotides. Since the ILK pseudokinase domain is unstable on its own and can only be expressed in a complex with α-parvin, all experiments were performed using the ILK:α-parvin complex. As expected, ILK was significantly stabilized by ATP and ATPγS. Interestingly, thermostabilization was observed only in the presence of Mg^2+^, highlighting the essential role of the cation for ATP binding to ILK. In contrast, other related analogs induced little to no thermostabilization, both in the presence and absence of Mg^2+^ (Figure 1D). These data further supported our hypothesis of targeting the ILK kinase domain with ATP-mimetic compounds. A ligand screen was conducted using the established DSF assay on an in-house library of approximately 380 kinase inhibitors. Using a *ΔT*_*m*_ (change in melting temperature) cutoff of 2.5 °C, three compounds were identified as hits, corresponding to a hit rate of 0.8%. Pelitinib, a pan-ErbB covalent inhibitor bearing a 6,4-disubstituted-3-cyano-quinoline core, showed the highest DSF shift (*ΔT*_*m*_ = 4.0 °C), while the diaminopyrimidine-containing TG101209 (a JAK2 inhibitor) and dasatinib (a nonselective dual SRC/BCR-ABL inhibitor) accounted for the other two hits (Figure 1E and F). Pelitinib exhibited the highest *ΔT*_*m*_ shift and the other two hits were close to our 2.5 °C cutoff, suggesting weak binding. We therefore selected pelitinib for subsequent biophysical validation and SAR studies.

To orthogonally validate pelitinib binding, we established a Surface Plasmon Resonance (SPR) assay. ILK was C-terminally tagged with an Avi-tag to generate biotinylated ILK:α-parvin complex, which was then immobilized onto a streptavidin-coated SPR sensor chip. The SPR assay was first validated using ATP-Mg^2+^, which showed a binding affinity (K_D_) of 3.2 μM, consistent with the previously reported value determined by ITC.^5^ In our SPR assay, pelitinib bound ILK with a K_D_ of 221 nM, confirming it as a bona fide ILK binder, and showed a 14.5-fold higher affinity compared to ATP. Since pelitinib is a covalent inhibitor bearing a reactive acrylamide warhead, we sought to exclude potential binding artifacts arising from nonspecific reactivity. To this end, we synthesized a saturated derivative of pelitinib at the Michael acceptor. Late-stage hydrogenation proved challenging, as classical metal-catalyzed protocols caused dehalogenation of the aniline ring and reduced the 3-cyano group. Milder reductive conditions, such as hydroboration–protodeboronation or diimide reduction, did not yield the desired product (Scheme S1). Ultimately, the use of an activated Stryker’s reagent^24,25^ with in situ–generated CuH provided an effective route to obtain dihydropelitinib (**1**, DHP) with satisfactory yields in a late-stage approach. **1** (DHP) exhibited a comparable binding affinity to pelitinib, with a K_D_ of 252 nM, and was subsequently employed as a tool molecule for further validation (Figure 2A–D). We additionally included the small molecules that have been reported as ligands of ILK; Cpd 22^21^ and EN300^25^ (EN300-6740186). Interestingly, both compounds exhibited typical nonspecific, “sticky” binding to the ILK:α-parvin complex, showing higher-than-expected responses and a linear steady-state curve with no discernible *R*_max_ (Figure 2E and F). We further confirmed that the two compounds were not direct ILK ligands using DSF, which, as expected, showed no significant *ΔT*_*m*_ shift for ILK:α-parvin complex (Figure 2G).

**Figure 2:**
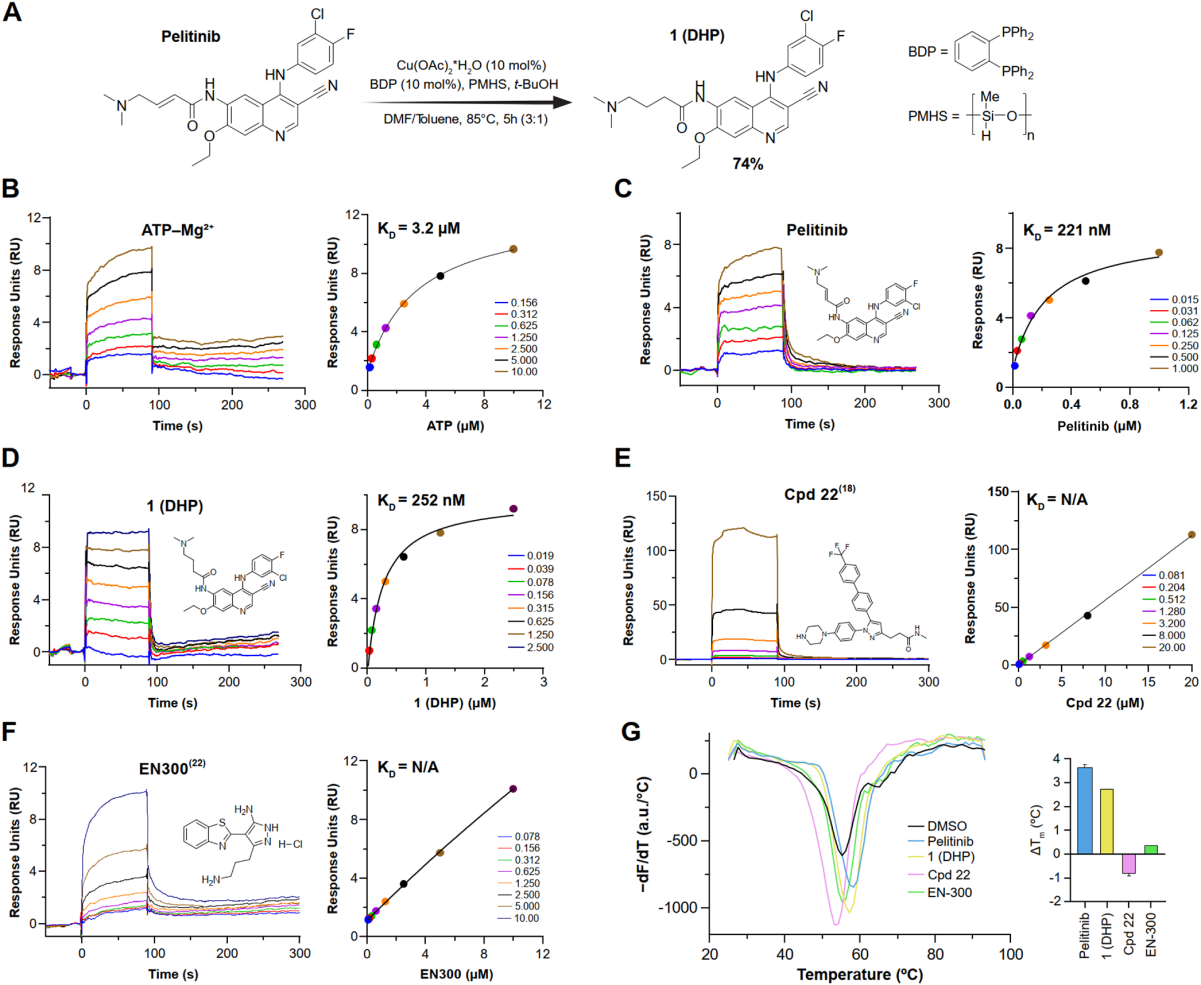
Synthesis of **1** (DHP) and binding assays (A) Reduction of **1** (DHP) using activated Stryker’s reagent. The reaction employed in situ–generated CuH to reduce the substrate, providing compound **1** (DHP) in satisfactory yields. Reaction conditions are indicated on the arrow. (B) ATP bound to ILK with an affinity of 3.2 μM. ATP was supplemented with Mg^2+^ to allow for ion coordination and stability. (C) Kinase inhibitor library screening hit, pelitinib, with a DSF shift of 4.0 °C, bound to the ILK kinase domain with a steady-state affinity of 221 nM. (D) **1** (DHP), lacking the reactive Michael acceptor double bond, interacted with a similar affinity to that of pelitinib. **1** eliminated the potential reactivity of the covalent warhead of pelitinib and resulted in a cleaner SPR steady-state. (E and F) Cpd 22^21^ and EN300,^23^ both compounds reported in the literature showed a dose-dependent response. However, the response was larger than expected based on the amount of ILK:α-parvin immobilized on the chip. Additionally, the sensorgrams did not reach near *R*_max_ (linear K_D_ plot), suggesting dose-dependent nonspecific or multiple binding events. (G) (Left) Mean DSF melt curves for pelitinib, **1** (DHP), and published ILK ligands were plotted. (Right) The thermostability shift upon compound binding, relative to DMSO, is depicted by a bar chart.

### Dihydro-Pelitinib (**1**) Stabilizes ILK in a Type I Binding Mode

The cocrystal structure of ILK:α-parvin in complex with **1** revealed a canonical ATP-mimetic, type I binding mode. An omit map confirmed unbiased electron density for **1** (DHP). The compound occupied the ATP-binding cleft, situated between the N- and C-lobes of the kinase, without perturbing the ILK:α-parvin interface. The ILK DVK valine (V340) adopted a conformation equivalent to the canonical DFG-in state. The quinoline nitrogen formed a backbone hydrogen bond with M272 in the hinge region, while the 3-cyano group provided additional stabilization through a hydrogen-bond interaction with S336. Additionally, the quinoline ring was hydrophobically sandwiched between M326 and V218. The dihalogenated aniline substituent at the 4-position extended into the back of the kinase pocket, forming weak interactions with surrounding hydrophobic residues. The 6-position 4-(dimethylamino)­butanamide of the quinoline ring bent toward the ribose-binding region of the kinase without being constrained by any contacts. The 7-ethoxy moiety resided in a groove of the hinge region formed by Y274 and G275 (Figure 3A–C).

**Figure 3:**
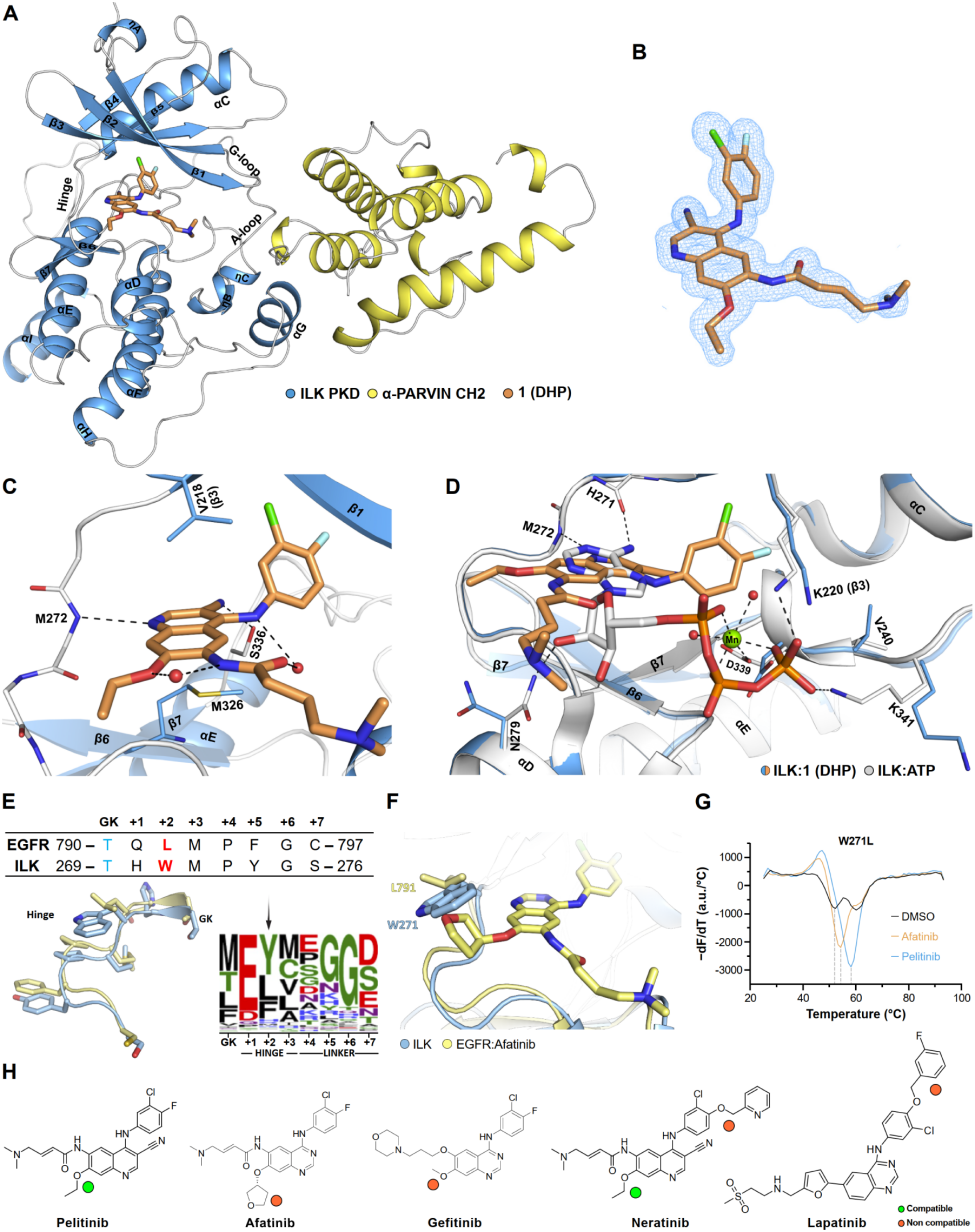
Quinoline scaffold interactions (A) Crystal structure of ILK:α-parvin:**1** (DHP) overall binding mode. The solved crystal structure of the ILK:α-parvin complex bound to the cyano-quinoline scaffold of **1** (DHP). Key kinase secondary structural elements and conserved motifs are labeled. (B) **1** (DHP) electron density map. The 2Fo–Fc electron density map (contoured at 1.0 σ) showed well-defined density for the cocrystallized ligand, defining its binding mode within the pseudoactive site of ILK. (C) ILK:**1** (DHP) binding mode. **1** (DHP) (light orange) bound to the ATP-binding pocket of ILK (sky blue) in a type-I binding mode, stabilizing the equivalent DFG-in conformation with the ILK DVK motif. Key interaction residues are highlighted and labeled. Water molecules are shown as red spheres. (D) Structural alignment of ILK:ATP and ILK:**1** (DHP) complexes. Key structural elements, interactions, and residues are labeled, and water molecules are shown as red spheres. (E) Structural basis for afatinib exclusion from the ILK ATP site. Sequence and structural alignment of the ILK (PDB: 3KMW) and EGFR (PDB: 4G5J) hinge regions is shown. The hinge regions aligned with an RMSD of 0.4 Å, and the full kinase domains with 2.0 Å. Hinge sequence alignment across the kinome was obtained from Dunbrack et al.^29^ The GK+2 position is indicated. (F) Structural analysis using the EGFR:afatinib cocrystal structures. The THF ring of afatinib clashed with the GK+2 tryptophan in ILK, whereas EGFR contained a leucine at the GK+2 position, which accommodated the THF ring. (G) DSF analysis of afatinib binding to the W271L mutant. DSF confirmed a partial rescue of afatinib binding by the W271L mutation (*ΔT_m_* = 2.0 °C). Pelitinib was used as a positive control (*ΔT_m_* = 5.8 °C). Overall, the results indicated that the GK+2 tryptophan residue contributed to binding selectivity at the 7-ethoxy position. (H) Steric exclusion observed for afatinib extended to other 3-cyano-quinoline and quinazoline scaffolds. Representative compounds bearing incompatible substituents at two key positions (the 4-aniline and/or 7-ethoxy) that clashed with the kinase back pocket and hinge region are highlighted.

We then compared the structure of ILK:α-parvin bound to **1** (DHP) with that of the ATP-bound structure,^4^ which aligned with a root-mean-square deviation (RMSD) of 0.44 Å. Notably, the two lysine residues (β3 K220 and DVK motif K341), which stabilized the ATP γ-phosphate, the DVK motif aspartate (D339), which stabilized Mn^2+^, and αD N279, which interacted with the ribose, all adopted different conformations (Figure 3D).

To assess whether the identified scaffold affected the ILK–IPP complex, we compared the apo structure of ILK:α-parvin with the ATP and **1** (DHP) bound structures. The kinase lobes exhibited no significant conformational changes, with RMSD values of 0.23 Å and 0.30 Å, respectively. All three structures crystallized in the same space group, and the N-lobe was stabilized by four symmetry-related molecules of ILK and α-parvin, which limited our ability to assess conformational changes at the kinase-lobe level (Figure S2A–C).

### **1** (DHP) and Pelitinib, but Not Related EGFR Inhibitors, Strongly Bound to ILK

The quinoline-containing pelitinib ligand was initially developed as a covalent inhibitor of EGFR.^26,27^ Numerous analogs of this chemotype, both covalent and noncovalent, have been developed to target various members of the ErbB family and other related kinases.^28^ Surprisingly, only pelitinib bound to ILK in our screening campaign, whereas other kinase inhibitors sharing a similar ATP mimetic moiety, such as the quinazoline derivatives, afatinib and gefitinib, did not bind or showed only weak *ΔT*_*m*_ shifts. We subsequently tested all seven quinoline or quinazoline-containing inhibitors from our in-house kinase inhibitor library using SPR (afatinib, gefitinib, erlotinib, canertinib, dacomitinib, lapatinib, and neratinib). Notably, all compounds showed no or very weak binding at the tested concentration range, except for canertinib, which showed a measurable K_D_ of 8.9 μM, still considerably weaker than that of pelitinib (Figure S2D). Interestingly, gefitinib, erlotinib, and bosutinib, the compounds for which ILK cocrystal structures were very recently released in the PDB (9D5E, 9D5G, and 9D5F, respectively), exhibited little or no binding in our biophysical assays. We speculate that these compounds were cocrystallized or soaked at very high compound concentrations.

### Structural Basis for the Lack of Afatinib Binding

Next, we performed a comparative analysis with afatinib, which did not bind to ILK. Afatinib is chemically highly related to pelitinib, featuring a quinazoline core, a tetrahydrofuran (THF) ring at position 7 in place of the ethoxy group, and it lacks the nitrile group at position 3. We aligned the ILK structure with that of EGFR bound to afatinib and focused on the hinge region, where the THF ring interacted.

Pair-wise sequence alignment of the hinge revealed a key variation at the GK+2 residue: ILK contained a bulkier tryptophan (W271), whereas EGFR had a smaller leucine (L791) (Figure 3E). Structural comparisons indicated that the larger THF ring of afatinib clashed with ILK W271, while EGFR L791 accommodated the THF ring. In contrast, the smaller ethoxy group in pelitinib was compatible with ILK W271, providing a likely structural basis for the lack of binding of afatinib. Interestingly, we observed a dual conformation of W271 in the ILK:**1** (DHP) structure, showing an alternate side-chain rotation. This indicated that, although the residue can flexibly rotate around the Cα, it is unlikely to fully flip into the bulk solvent to accommodate the THF ring of afatinib (Figure 3E and F, and Figure S2E–H).

We additionally analyzed the GK+2 position across the kinome and found that only the three related RAF family kinases, ARAF, BRAF, and RAF1, contained a tryptophan at this position. Structural analyses of all available BRAF structures, including both apo and inhibitor-bound forms, revealed that this residue remained largely immobile regarding its side chain conformation. These analyses reinforced the importance of the GK+2 residues in contributing to binding selectivity at this position in ILK (Figure S2I and J).

To confirm the contribution of the GK+2 residue to ligand tolerance, we generated a W271L mutant of ILK to assess whether afatinib binding could be rescued. DSF assays of the mutant ILK:α-parvin complex revealed that the mutant partially restored afatinib binding (*ΔT*_*m*_ = 2.0 °C), with pelitinib used as a control. The partial rescue, in contrast to pelitinib, can be explained by the absence of the 3-cyano group in afatinib, which forms a key hydrogen bond with ILK residue S336. Notably, the mutant ILK W271L:α-parvin also showed lower protein yield and thermostability compared to that of the wild-type protein complex, further highlighting the role of W271 in maintaining ILK structural integrity in addition to contributing to ligand selectivity (Figure 3G).

Finally, we sought to extend the observed incompatibility of afatinib to other cyano-quinoline and quinazoline analogs. Structural and chemical analyses revealed that modifications at the 7-ethoxy and 4-aniline positions were particularly sensitive. At the 7-ethoxy position, in addition to the incompatibility of the tetrahydrofuran (THF) ring, we found that methoxy substitutions, unlike ethoxy, rendered the compounds nonbinding, as observed for dacomitinib and gefitinib. Furthermore, the 4-aniline position was highly sensitive to bulky substitutions, such as those at the 3-chloro or 4-fluoro positions, as seen in lapatinib and neratinib (Figure 3H and Figure S2D). In both cases, structural analysis revealed that the bulkier substituents were sterically excluded from the smaller ILK back-pocket region.

### SAR-Driven Chemistry Identified Key Positions Determining Ligand Binding

The chemical exploration of the 3-cyano-4-amino quinoline core scaffold was centered around three main areas: substituents at the 4-position of the aniline ring that targeted the back pocket of the kinase (green), derivatization at the 6-position aimed at substituting the acrylamide Michael acceptor (blue), and the 7-position introduced different hinge targeting moieties to explore the chemical space at this position critical for binding (orange) (Figure 4A).

**Figure 4:**
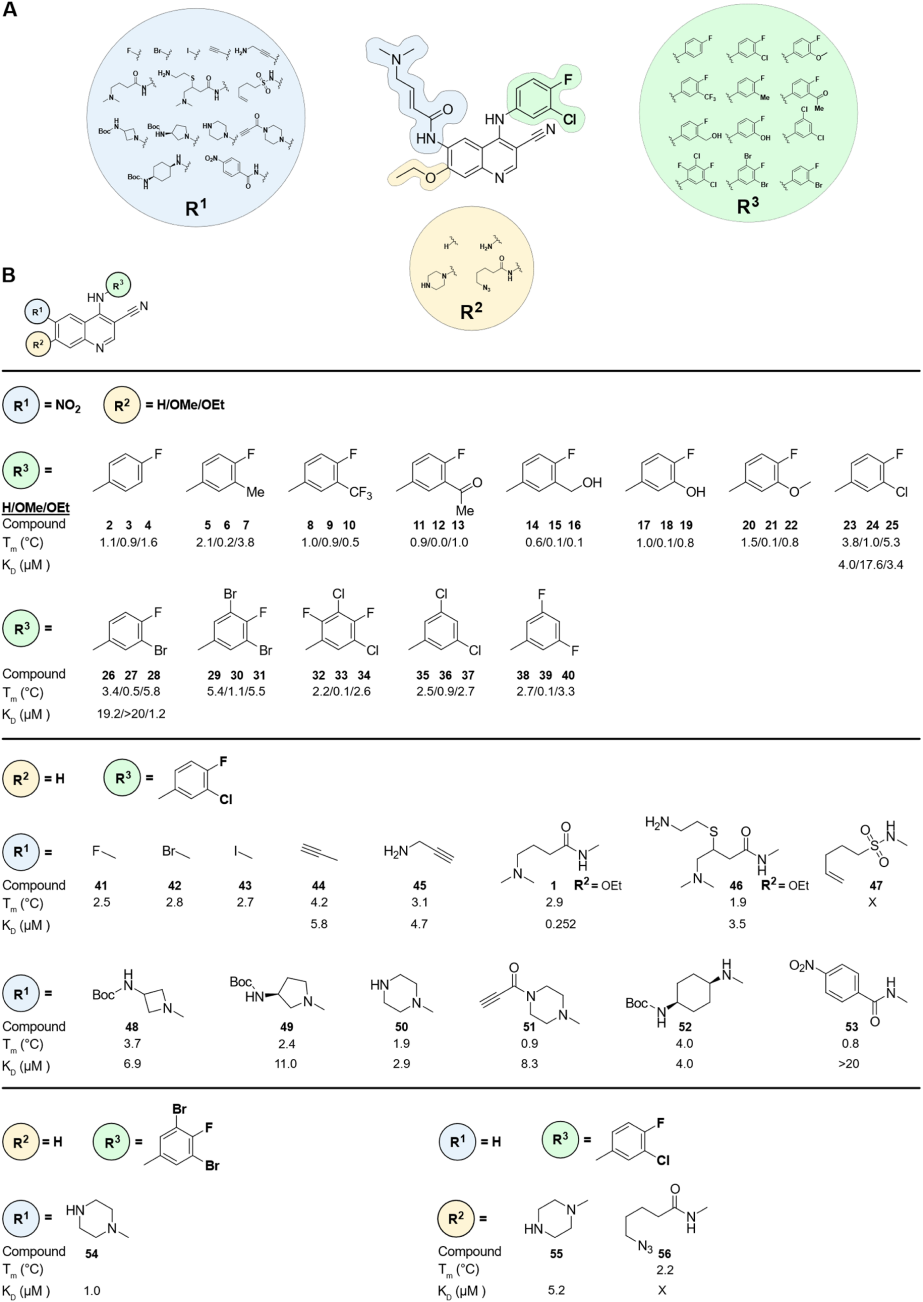
Structure–activity relationship of the 3-cyano-carbonitril scaffold (A) Overview of the SAR modification hotspots on the central quinoline-3-carbonitrile scaffold: Derivatization of the front pocket/solvent targeting region R1 (blue) with different amine-containing building blocks at the 6 position. Modification of the 7 position (yellow) with functional groups pointing toward the hinge region of kinases. Aniline derivatives R3 (green) were introduced at the 4-position of the quinoline core to target the back pocket of the kinase. (B) In vitro binding data for the series: The SAR series was screened with thermal shift assay (*T*_m_ values in °C), and a subset of compounds (>3 °C in *T*_m_) was orthogonally characterized using SPR (K_D_ in μM). Top panel: Back pocket targeting anilines with different substitution patterns. Meta–para halogenated anilines performed best in combination with the ethoxy substituent at 7-position. Middle panel: Different R1 substituents at the 6 position were introduced, featuring largely polar groups that would putatively match the polarity of the front pocket region of ILK. Most of the groups were tolerated but decreased the K_D_ (>1 μM) except for 4-(dimethylamino)­butanamide derivative **1 (DHP)**. Bottom panel: The combination of the m-dibromo and *p*-fluoro aniline and a piperazine was superior in terms of binding compared to the m-Cl, p-F analogue. Substitutions at the 7-position were largely tolerated with K_D_ values up to ∼2 μM. Compounds with no SPR values were not measured, while those indicated with an “X” showed insufficient curve quality for reliable measurement.

For the rapid evaluation of the back-pocket targeting moiety, we employed a parallel synthesis approach combined with direct screening evaluation of crude reaction mixtures, which allowed us to rapidly interrogate a large chemical space at this position (Scheme 1A). The DSF assay was used as the primary screen to evaluate the SAR series. Different anilines at the 4-position were introduced via a one-step S_N_Ar reaction with 4-chloro-6-nitroquinoline-3-carbonitriles bearing various substituents at the 7-position (H, methoxy and ethoxy), yielding compounds **2–40** (Figure 4B, top panel). Conversion rates of the parallel synthesis chemistry can be found in the Supporting Information (spectral data for synthesized compounds). In comparison to the 3-chloro-4-fluoro substitution pattern on the aniline ring, most of the compounds (**2–22**) showed poor binding in the primary assay. The introduction of polyhalogenated anilines and the substitutions with heavier halogens resulted in comparable or slightly increased thermostability. The direct exchange of the chloro residue (**23** and **25**) to bromine (**26** and **28**) led to a slight increase in thermostability for **28** (*ΔT*_*m*_ = 5.8 °C). This was confirmed by SPR measurements, which showed a K_D_ value of 1.2 μM for **28** and 3.4 μM for **25,** respectively.

**Scheme 1:**
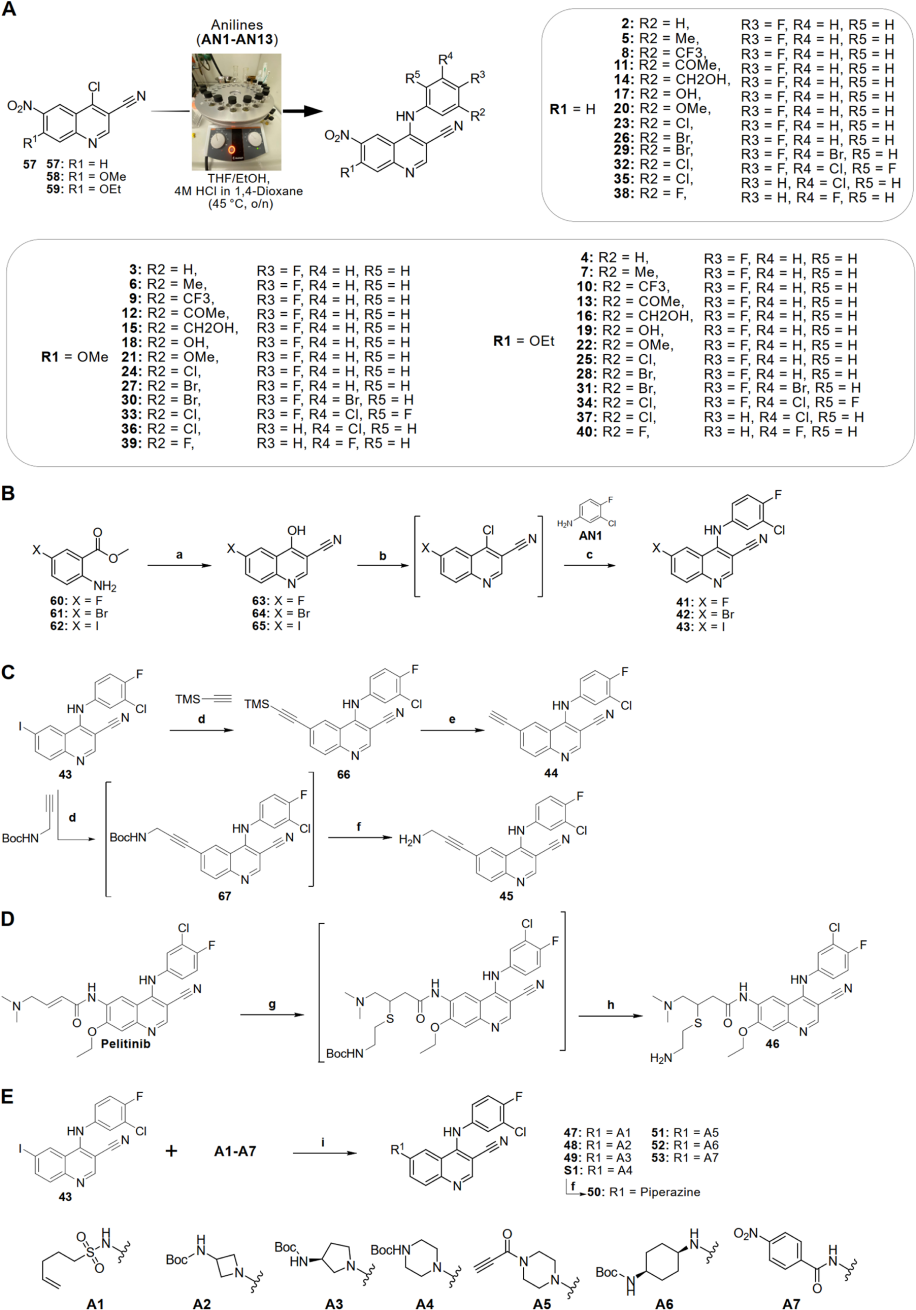
Synthetic Routes of the Core Ring Moiety and Analogs on 4, 6, and 7-Positions^a^

a Reagents and conditions (A) Parallel synthesis setup for up to 40 vials: Aniline, ACN, 40 °C, on. (B) Assembly of the central quinoline scaffold (a) i: DMF-DMA, DMF, rt, 2 h, ii: ACN, *n*-BuLi, THF, −78°C-rt, on; (b) POCl_3_, 100 °C, 1 h; (c) 4 N HCl in dioxane, THF, 0°-rt, on; (C) Sonogashira coupling and follow-up deprotections (d) Pd­(dppf)­Cl_2_, CuI, TEA, DMSO, 100 °C, on; (e) TBAF, DCM, 1 h, rt; (f) TFA, DCM, 1 h, rt; (D) Michael addition leveraging the Michael system of Pelitinib (g) *tert*-butyl (2-mercaptoethyl)­carbamate, DMF, 120° μW, 10 h (h) TFA, TES, 30 min, rt; (E) Ullmann-type coupling with various amines (i) CuI, *l* -proline, K_2_CO_3_, DMSO, closed vessel, on.

Since each aniline used in this series was coupled to three different quinoline scaffolds bearing substituents at the 7-position, we could additionally interrogate the first modifications on this hotspot. While deletion of the 7- position substituent (**23**, *ΔT*_*m*_ = 3.8 °C, and K_D_ = 4.0 μM) led to stabilization of the protein complex comparable to that of the ethoxy (OEt) residue (**25**, *ΔT*_*m*_ = 5.38 °C, and K_D_ = 3.4 μM), only weak binding was observed for the methoxy (OMe) substituent (**24**, *ΔT*_*m*_ = 1.0 °C, and K_D_ = 17.6 μM) throughout the series. For this series, we used a truncated form of the hit compound pelitinib, which replaced the 4-(dimethylamino)­but-2-enamide substituent with a NO_2_ moiety at the 6 position that facilitated quick screening of the back-pocket. This explained the overall lower binding of this series compared to **1** (DHP) and pelitinib.

A similar simplification strategy was employed for the exploration of the 6-position of the quinoline core: the back-pocket 3-chloro-4-aniline and the 7-position were kept as constants, which simplified the synthetic accessibility of the hinge region targeting moiety. Compounds **41–43** were synthesized using a cyclization reaction starting from *para-halogenated* (X = F, Br, I) anthranilic acid derivatives in a 2-step synthesis via in situ formed halo-dimethylformimidamide-benzoate, which was reacted with ACN after deprotonation with *n*-BuLi (Scheme 1B). DSF assay on compounds **41–43** revealed tolerability of these modifications (*ΔT*_*m*_ = ∼3 °C) and encouraged us to use this simplification throughout the series. These compounds were additionally used as synthetic handles for further chemical exploration of this position. Alkynes with either no substitution (**44**) or with an attached amino functionality (**45**) were introduced using a Sonogashira coupling of **43** with TMS-acetylene or *N*Boc-propargylamine, followed by deprotection with TBAF and TFA, respectively (Scheme 1C). The alkyne derivatives showed good stabilization of the ILK:α-parvin complex and were orthogonally measured using SPR (**44**, *ΔT*_*m*_ = 4.2 °C, K_D_ = 5.8 μM; **45**, *ΔT*_*m*_ = 3.1 °C, K_D_ = 4.7 μM). Initial attempts that aimed at derivatizing an amino group at position 6 (such as amide coupling) failed due to the low nucleophilicity of the amino functionality in the *para* position to the quinoline nitrogen. We opted toward a direct attachment of the amino functionality and reacted fluorine-bearing **41** with different primary and secondary amines. Notably, only the reaction with piperazine in ethanol in a microwave reactor yielded the desired nitrogen-C­(sp2) linkage in compound **50** (SI Scheme). Subsequent amide coupling of **50** with propiolic acid and HATU yielded **51**. Metal-catalyzed cross-coupling chemistry was applied, aimed at directly installing the amino functionality at the 6-position. Initial Buchwald-Hartwig amination screens using different amines, bases, catalyst-ligand pairs and solvents all failed to give the desired products. A detailed listing of failed procedures and synthetic limitations can be found in the Supporting Information (Scheme S1) The transition from palladium to copper using an Ullmann-type coupling with l-proline and K_2_CO_3_ in DMSO turned out to be a reliable way of introducing different nitrogen-containing functionalities such as sulfonamides (**47**), primary amines (**48**), secondary cyclic amines (**49, 50**), as well as benzamides (**53**) (Synthesis Scheme 1E). Cyclic amines showed binding to the target with six-membered ring systems (**50**, **52**) performing best in SPR (**50**, K_D_ = 2.7 μM; **52**, K_D_ = 4.0 μM). Extension on the N1 nitrogen ring of piperazine compound **50** via amide bond formation decreased binding to ILK:α-parvin (**51**, *ΔT*_*m*_ = 0.9 °C, K_D_ = 8.3 μM). No binding was observed in both SPR and DSF with sulfonamide (**47**) and benzamide (**53**) introduction. Next, we targeted the 7-position of the quinoline ring and used different functionalities that could be introduced late-stage on the quinoline core. Béchamp’s reduction of previously synthesized **S1** resulted in **55**. Extension of **55** via coupling to 4-chlorobutyryl chloride and subsequent azide introduction yielded **57**. Both modifications were tolerated (**55**, *ΔT*_*m*_ = 2.4 °C, K_D_ = 6.0 μM; **57**, *ΔT*_*m*_ = 2.2 °C, K_D_ = 5.2 μM), which showed that growing in this position via amino linkage is a suitable strategy. The bulkier piperazine ring directly linked to the quinoline core was tolerated as well (**56**, K_D_ = 5.2 μM). Lastly, we combined the best-performing, newly introduced substituents, which resulted in **57** (K_D_ = 1.0 μM).

Based on our SAR in conjunction with crystal structure analysis, we deemed the 7-position as most impactful in terms of binding to ILK:α-parvin. Thus, we decided to keep the ethoxy substitution in position 7 and performed late-stage modifications using pelitinib. We harnessed the acrylamide and introduced a thioether linkage via Michael addition with commercially available 2-(Boc-amino)­ethanthiol and subsequent deprotection with TFA, resulting in **46** (Scheme 1D). Contrary to our expectations, this substitution was not well tolerated as binding was decreased to K_D_ = 3.5 μM.

The extensive chemical exploration combined with crystal structure analysis resulted in notable SAR trends: The 4-position aniline prefers halogenation and profits from heavy halogen substitution (**25** -Cl to **28** -Br). Substitutions at the 6 and 7-positions of the quinoline ring were largely tolerated but could not substantially boost target binding. The most significant modification was the introduction of different ethers at the 7-position, which was crucial in differentiating between binders and nonbinders (afatinib, **24** and **27**). Although clear SAR trends emerged, none of the tested compounds exhibited higher affinity than the initially discovered parent **1** and pelitinib. Overall, the chemical space appeared less amenable to expansion, as both the back-pocket and the ethoxy positions were sensitive to substantial modifications.

### NanoBRET-Based Assay Enabled Cellular Evaluation of Target Engagement

After establishing biophysical and crystallographic screening platforms for ILK, we next evaluated how the ligands engaged with ILK in a cellular context. To set up the assay, we synthesized three custom NanoBRET tracers featuring distinct exit-vector scaffolds with PEG linkers, along with four Promega tracers selected based on structural and phylogenetic analyses from the NanoBRET tracer database (Figures S3A).^30^ We then evaluated tracer performance via titration experiments to identify the most suitable tracer, based on optimal binding signal intensity and assay window.

We initially analyzed the assembled IPP complex structure to identify optimal tag positions based on their proximity to the ATP-binding pocket. This analysis confirmed that placing NanoLuc at the ILK C-terminus was optimal and did not interfere with IPP complex assembly (Figure S3B). Initial assay setup using a C-terminal NanoLuc-tagged full-length ILK construct with transient overexpression failed to generate a measurable BRET signal change with any of the tracers, upon displacement with pelitinib and **1** in both intact and digitonin-permeabilized cells. Given that ILK is known to be unstable in isolation and requires components of the IPP complex for stability, we next explored coexpression strategies with α-parvin and PINCH.

Initial tracer titration experiments revealed that **68** showed the highest BRET signal and the widest assay window among all tracers tested (Figure S3C). We further validated this tracer warhead and its piperazine exit vector (**50**) by solving its X-ray cocrystal structure with ILK and measuring binding affinity by SPR (Figure 5A and B; Figure S3D and E). Unfortunately, our NanoBRET assay setup functioned effectively only in the digitonin-permeabilized cells, not in live cells, consistent across all tracers tested. To further assess the assay behavior in live cells, we tested tracer **68** in all the coexpression systems with PINCH and α-parvin (Figure S3F and G). However, the data confirmed that the assay only functioned in digitonin-permeabilized cells. The optimal coexpression system was using C-terminal ILK and α-parvin, which resulted in the highest BRET signal.

**Figure 5:**
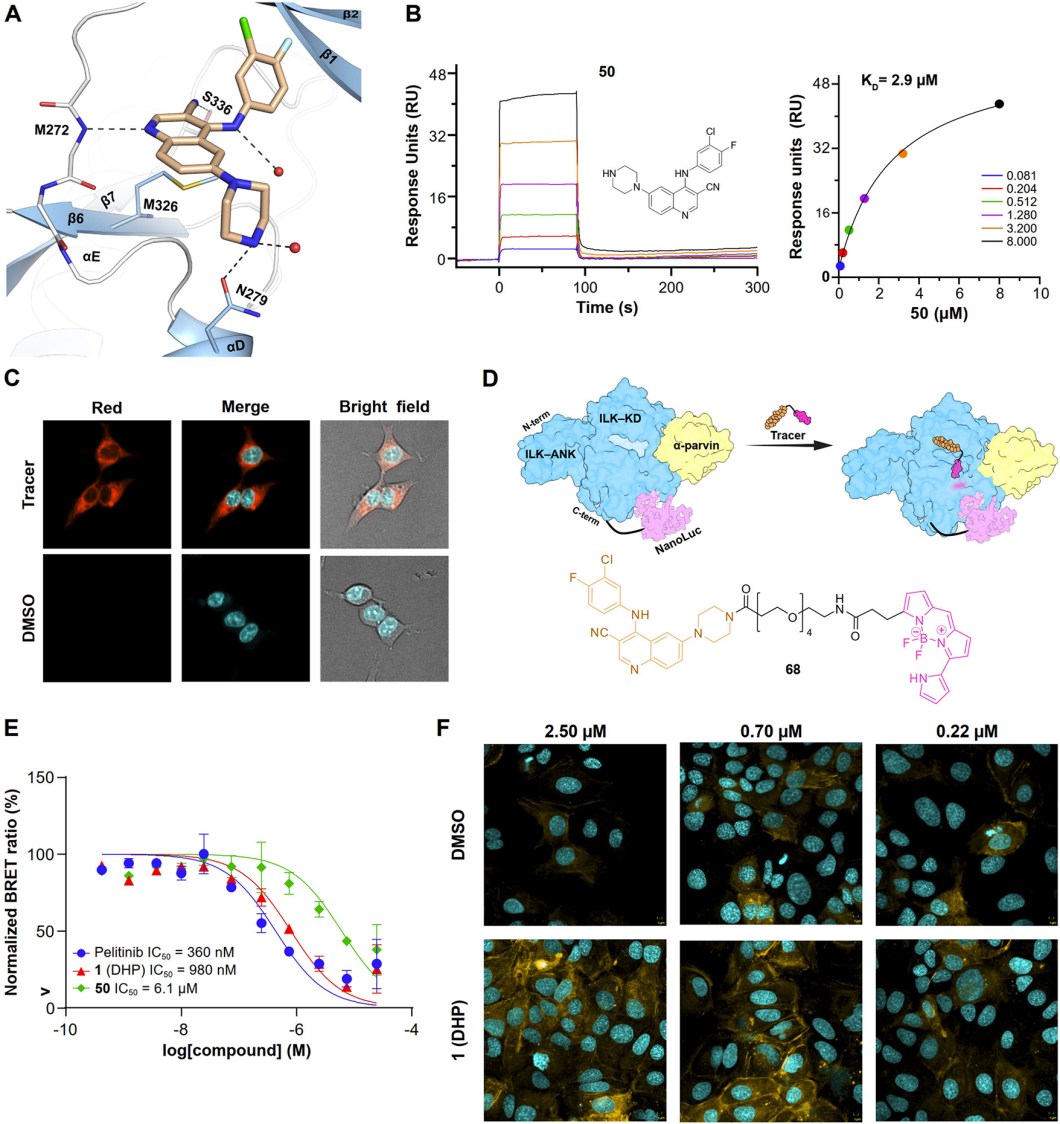
Cellular target engagement and effect on actin cytoskeleton organization (A) Co-crystal structure of the ILK:α-parvin complex bound to the warhead (**50**) used for NanoBRET tracer synthesis. Key molecular interactions and secondary structural elements are highlighted and labeled. (B) SPR analysis of **50**. Sensorgram responses and the corresponding 1:1 steady-state affinity model fits are shown, together with the fitted K_D_ value. (C) HEK293T cells were incubated with tracer **68** (2 μM, 2 min) and imaged by confocal microscopy. Red: ILK tracer (**68**); cyan: nuclei (Hoechst 33342); merged: overlay; brightfield: overlay with cell boundary. The tracer signal was detected in the cytoplasm, indicating cell permeability. Image is shown as a zoomed-in view; scale bar information is provided in Figure S3H. (D) Final optimized NanoBRET configuration using full-length, optimized α-parvin and ILK constructs. The optimal tag terminus and the tracer molecule employed in the assay are shown. The image was rendered using UCSF ChimeraX (version 1.8).^32^ (E) NanoBRET measurements in digitonin-permeabilized cells. Normalized BRET ratio (%) versus compound concentration plots showing fitted binding curves for Pelitinib, **1** (DHP), and **50**. Corresponding IC_50_ values were determined from dose–response analyses using GraphPad Prism.^33^ (F) Effect of **1** (DHP) on actin cytoskeleton organization. Cells were treated with DMSO or increasing concentrations of compound **1** (DHP), stained with CellMask Orange Actin Tracking Stain (F-actin, appearing yellow) and Hoechst (nuclei, blue), and imaged on a CQ1 system. DHP induces concentration-dependent changes in F-actin organization. Scale bar: 6 μm.

To assess the cell permeability of the tracer, we performed a cellular tracer uptake experiment using confocal microscopy (CQ1) in HEK293T cells (Figures 5C and S3H). Visual uptake of the tracer was observed in both the red and merged channels at 2 μM already after 2 min treatment with the tracer, indicating that the tracer was cell penetrant. The absence of a BRET signal in intact cells may be explained by potentially lower tracer affinity for ILK, competition with intracellular ATP, and possible steric hindrance of the NanoLuc–tracer interaction due to the dense assembly of the IPP complex at focal adhesions. In digitonin-permeabilized cells, at least some of these limitations were likely alleviated, allowing greater NanoLuc–tracer access. Notably, the substrate addition step diluted intracellular ATP concentrations to an average of approximately 1 μM, further reducing ATP competition and enabling robust BRET signal detection (Figure S3I).

We proceeded to validate selected key compounds in digitonin-permeabilized cells using the optimized system and the developed tracer (Figure 5D). Afatinib, Cpd 22, and EN-300, which showed no binding in both SPR and DSF, did not display any dose-dependent engagement in the NanoBRET assay. In contrast, pelitinib, **1** (DHP), and **50** all exhibited binding with affinities comparable to those measured by SPR (Figure 5E and Figure S3J). The establishment of the NanoBRET assay, albeit only in digitonin-permeabilized cells, thus provided a crucial orthogonal platform to validate on target activity.

Next, we evaluated the selectivity of **1** (DHP) and pelitinib using the NanoBRET based K192 kinase panel. At 1 μM, **1** (DHP) revealed tracer displacement above 50% for only three kinases of this panel, all located in different branches of the kinome phylogenetic tree (PLK4, FYN, and MAPK9 (JNK2)). Notably, DHP preferentially targeted the JNK2 isoform while the highly similar JNK1 (MAPK8) showed only 45.4% tracer displacement. In contrast, pelitinib engaged with 16 kinases above our 50% threshold under the same conditions, that indicating that our strategy of removing the reactive Michael acceptor substantially improved selectivity. Indeed, many of the inhibited kinases shared a cysteine residue at the same position as the primary target of pelitinib, the epidermal growth factor receptor (EGFR). Remarkably, FYN was the only tyrosine kinase significantly inhibited by DHP (Figure S3K, L and M).

### **1** (DHP) Affects Actin Cytoskeleton Organization Associated with Altered Actin Dynamics

We assessed the effect of compound **1** (DHP) on actin cytoskeleton organization. Given that ILK, through the IPP complex, plays a direct role in regulating the actin cytoskeleton, we investigated whether treatment of cells with DHP altered actin structure. Huh-7 (human hepatoma) cells were treated with increasing concentrations of DHP, with DMSO serving as the vehicle control. After 48 h, end point imaging was performed using CellMask Orange Actin Tracking Stain and Hoechst nuclear stain, and images were acquired using the CQ1 high-content imaging system.^31^

DHP treatment induced a dose-dependent reorganization of the actin cytoskeleton. At concentrations ≥700 nM, cells exhibited increased F-actin staining intensity accompanied by a characteristic punctate distribution (Figure 5F). Notably, pelitinib-treated cells displayed a more pronounced phenotype and greater cell death at an equivalent concentration to **1** (DHP), suggesting that the Michael acceptor moiety in pelitinib contributed to enhanced cytotoxicity, potentially through off-target engagement of cysteine-containing proteins (Figure 5F and Figure S4A). Collectively, these results demonstrate that **1** (DHP) perturbed actin cytoskeleton organization and actin dynamics in a concentration-dependent manner.

Finally, the cytotoxicity of **1** (DHP) was assessed using the CellTiter-Glo cell viability assay. The assay, conducted over 72 h, revealed that **1** (DHP) exhibited cytotoxicity in two tested cell lines, Huh-7 and lung adenocarcinoma cell line A549, at concentrations above 1 μM and 250 nM, respectively, after prolonged (48 h) exposure (Figure S4B).

## Discussion

In this study, we describe the identification and SAR of the first ATP competitive ligands targeting ILK. The pharmacological investigation of ILK has been limited so far due to the paucity of bona fide ligands target engagement assays. In our study, we found that Cpd 22 (ILK-IN-2),^18^ a widely used ILK binder was not a ligand of ILK:PARVA and likely acts upstream of ILK. We successfully validated binding of pelitinib (K_D_ = 221 nM) and its nonreactive dihydro form **1** (K_D_ = 252 nM) using biophysical and crystallographic methods. Furthermore, we established a functional NanoBRET assay, enabling the assessment of ILK ligands on full-length ILK:α-parvin which showed a good signal-to-noise assay window at least in permeabilized cells.

Our medicinal chemistry approach provided a streamlined strategy to systematically explore substitutions at the 4-position (back pocket), 6-position (front pocket), and 7-position (hinge region) of the central 3-cyano-quinoline scaffold in various combinations. Direct screening of compounds synthesized in small amounts in a parallelized workflow allowed us to drastically reduce the time from synthesis to evaluation, reagent costs and enabled the rapid assembly and testing of 39 potential ILK ligands, each featuring different substitutions on the pendant aniline ring. The reactions had high turnover rates (>90%) justifying direct screening by our *ΔT*_*m*_ assay.

Although the medicinal chemistry campaign on this scaffold did not yield further improvement in ligand affinity, it provided valuable insights into the chemical space of the ILK ATP-binding pocket. For instance, a relatively underexplored modification hotspot of 3-cyanoquinolines was the 7-position, which is commonly substituted with ether-containing functional groups, as seen in the FDA-approved kinase inhibitor neratinib or in the quinazoline-containing kinase inhibitors gefitinib or afatinib. In a previous study where we developed mutant-selective, macrocyclic quinazoline-based EGFR inhibitors, we found that a subtle modification, replacing the 7-position methoxy group with a hydrogen, unexpectedly abolished kinase binding.^34^ Similarly, in our ILK SAR series, we observed that this position was highly sensitive to modification (e.g., **23**, 4.0 μM vs **24**, 17.6 μM). Furthermore, kinome-wide sequence comparison revealed that the GK+2 tryptophan residue, located near the 7-position, is unique to ILK and steered the chemical modification space at this site. The K192 selectivity panel also highlighted that removing the reactive Michael acceptor electrophile to a saturated variant of the ligand improved selectivity by approximately 5-fold. Additionally, ILK assumed an αC-out helix conformation in all structures, in which the canonical β3 K220:αC E238 salt bridge, a hallmark of active kinases, was not conserved. This structural feature generated a large back pocket that could be exploited to improve both affinity and selectivity, as has been demonstrated for other kinases such as MER tyrosine kinase (MerTK) with αC-out conformations.^35^ Taken together, these findings highlight both the opportunities and challenges in enhancing ILK affinity and selectivity, and indeed emphasize the potential need to explore alternative scaffolds for effective targeting.

A key question arising from our study was whether the identified scaffold induced any conformational changes that may interfere with ILK scaffolding functions at focal adhesions via the IPP complex. Our structural analysis did not reveal significant conformational changes in both kinase lobes; however, this assessment may be also a constraint imposed by crystal packing. Molecular dynamics (MD) simulations of ILK,^16^ combined with direct cellular functional assays probing focal adhesions and actin cytoskeleton dynamics, could provide complementary approaches to address this question in the future.

A promising alternative strategy to modulate ILK function and the IPP complex could be the development of Proteolysis Targeting Chimeras (PROTACs), which would bypass the need for conformational changes to affect its scaffolding functions by directly inducing ILK degradation and, in turn disrupting the IPP complex. The 3-cyano-quinoline scaffold can provide a crucial starting point with two potential exit vectors for PROTAC design: the *N,N*-dimethylated tertiary amine at position 3 and the 7-position, as demonstrated by the synthesis of **46**. Additionally, given the critical role of α-parvin in ILK stability, future strategies could also consider targeting the ILK:α-parvin interface as an alternative to the ATP-binding pocket. Finally, we provide here the first validated ILK-binding ligand, offering an important chemical starting point for future therapeutic exploration, for instance by developing selective ILK degraders such as PROTACs.

## Conclusions

Despite advances in kinase inhibitor development, targeting the ATP pocket of the pseudokinase ILK has remained elusive. This study reports a well validated small-molecule ligand of ILK, supported by biophysical characterization and X-ray structural data. The cyano-quinoline scaffold identified here provides a well-defined starting point for further chemical space optimization and structure–activity relationship studies. Beyond serving as a foundation for future ligand development, the favorable selectivity of the developed ligand also provide a first tool to probe ILK biology, enabling future investigation of its scaffolding functions in focal adhesions and disease contexts. Additionally, the chemical scaffold can be leveraged as a starting point for targeted protein degradation strategies (PROTACs), expanding the therapeutic possibilities for this previously underexplored pseudokinase. Collectively, our work establishes the well-known ATP mimetic cyano-quinoline scaffold and provides a much-needed chemical foothold for probing ILK biology, laying the foundation for the development of next-generation selective and efficacious ILK modulators.

## Experimental Section

### Protein Expression and Purification

ILK (residues 182–502) and α-Parvin (residues 248–372) were cloned into the pDUET-1 vector using the MCS1 and MCS2 sites, respectively. α-Parvin contained an N-terminal His-tag followed by a TEV cleavage site, whereas ILK was expressed without an affinity tag. The expression plasmid was transformed into *E. coli* Rosetta cells, and transformants were selected using the appropriate antibiotics.

Overnight cultures grown at 37 °C were used to inoculate six 1 L TB cultures containing 100 μg/mL ampicillin. Cells were grown to an OD_600_ of 1.0, after which the temperature was reduced to 18 °C and protein expression was induced with 0.5 mM IPTG. Cultures were incubated with shaking overnight, and cells were harvested the following day by centrifugation and resuspended in binding buffer.

Cell pellets were lysed by sonication (8 s on/16 s off, total 8 min). Cell lysates were clarified by centrifugation at 15,000 rpm, and the supernatant was loaded onto a 5 mL Ni-NTA column pre-equilibrated with binding buffer. The column was washed with 10 column volumes of binding buffer, and the protein was eluted with 5 column volumes of buffer containing 250 mM imidazole. The His-tag was cleaved with TEV protease (1:50 w/w) during overnight dialysis (3 kDa cutoff) at 4 °C. The sample was then further purified by passing it onto a 1.5 mL Ni-NTA column pre-equilibrated in size exclusion buffer (20 mM HEPES, pH 7.5, 150 mM NaCl, 5% glycerol, and 0.5 mM TCEP). The column was eluted with size exclusion buffer containing increasing concentrations of imidazole (10 mM, 20 mM, 40 mM, and 250 mM). The samples analyzed by SDS–PAGE eluted in the flow-through, 10 mM, and 20 mM imidazole fractions. The pooled samples were concentrated to 2.5 mL and loaded onto a 120 mL Superdex 75 size-exclusion column. Eluted complex was checked for purity by SDS–PAGE and confirmed by mass spectrometry. The pooled peak fractions containing the complex was concentrated to 12.5 mg/mL and flash frozen in several aliquots.

For SPR experiments, an Avi-tag was installed at the C-terminus of ILK, separated by a G4S linker. Protein production was carried out by cotransforming with a compatible vector encoding untagged BirA ligase, and the biotinylated complex was purified in the same manner as described above.

The ILK W271L mutant was generated using a standard QuickChange site-directed mutagenesis protocol^36^ and purified as described above. Incorporation of the mutation was confirmed by both Sanger sequencing and mass spectrometry.

### DSF Assay

A 2 μM ILK:α-parvin complex solution in 20 μL assay buffer (20 mM HEPES, pH 7.4, 150 mM NaCl, 0.5 mM TCEP, 5% glycerol) was mixed 1:1000 with SYPRO Orange dye (Sigma) and compounds (10 μM final concentration). Fluorescence was monitored from 25–95 °C using an Mx3005P PCR instrument (excitation/emission: 465/590 nm). Data were analyzed with MxPro software.

### SPR Binding Assays

The SPR analysis was performed on a Biacore T200 (Cytiva Life Sciences) at 25 °C. Approximately 8000 RU of biotinylated ILK:α-parvin was loaded onto a Series S CM5 chip coated with streptavidin. All experiments were performed at 25 °C. The chip was equilibrated with a running buffer containing 20 mM HEPES, pH 7.4, 150 mM NaCl, 0.5 mM TCEP, and 0.05% Tween 20. A titration of serially diluted compounds was performed. The compounds were allowed to bind over the surface at a flow rate of 30 μL/min for 90 s, followed by a dissociation wash for 180 s. The sensorgrams were double-reference subtracted and analyzed using the Biacore evaluation software, and curves were fitted to a steady-state affinity fit model.

### Crystallization and X-Ray Data Processing

Purified ILK:α-parvin complex was concentrated to 12.5 mg/mL and crystallized using sitting-drop vapor diffusion at 4 °C. Initial small crystals grew in 0.1 M HEPES sodium pH 7.5, 10% (v/v) 2-propanol, and 20% (w/v) PEG 4000. Seeds from these crystals were used in a Microseed Matrix Screening (MMS), producing larger crystals in 0.1 M HEPES pH 7.5, 10% (w/v) PEG 8000, and 8% (v/v) ethylene glycol. Crystals were harvested, cryoprotected with reservoir solution containing 20% (v/v) ethylene glycol, and flash-frozen in liquid nitrogen for X-ray data collection.

Diffraction data were collected at the Diamond Light Source. Data were reduced (indexed and integrated) using Diamond’s automated pipelines, and subsequently merged and scaled with AIMLESS.^37^ The structure was solved by molecular replacement using PHASER,^38^ refined using PHENIX,^39^ and ligand density was confirmed with an omit map. The final refined coordinates were validated using COOT^40^ and deposited in the Protein Data Bank (PDB IDs: 9TP9 and 9TPD). Also see Table S1 for data collection and refinement statistics.

### NanoBRET Target Engagement Assays

Gene encoding full-length ILK, cloned in frame with NanoLuc fusion tag to the C terminus (cloned in-house), was transfected into HEK293T cells using FuGENE HD (Promega, E2312) following manufacturer’s protocol and proteins were allowed to express for 20 h at 37 °C and 5% CO_2_. Additionally, for coexpression experiments, plasmids encoding α-parvin and PINCH were cotransfected along with the ILK-C vector, to improve the overall assay quality. The 10 μL of transfected cells after trypsinization and resuspending in Opti-MEM (Life Technologies, 31985070) were dispensed into each well of the 384-well plate (Greiner 781207) at a cell density of 2 × 10^5^ cells/mL. For dose–response BRET measurements, compounds at various concentrations, immediately followed by Tracer at an optimum K_D_ concentration (FGMM175-2 μM), determined from tracer titration experiment, were pipetted using an Echo 550 acoustic dispenser (Labcyte). The system was allowed to equilibrate for 2 h at 37 °C and 5% CO_2_ prior to BRET measurements. To measure BRET signal, the NanoBRET NanoGlo Substrate (Promega, N1573) was added as per the manufacturer’s protocol, and filtered luminescence was measured on a PHERAstar plate reader (BMG Labtech) equipped with a luminescence filter pair (450 nm BP filter (donor) and 610 nm LP filter (acceptor)). For lysed NanoBRET measurement, 25 nL of Digitonin (0.05 μg/μL) was pipetted to each well using Labcyte and the plate was incubated for 5 min at 37 °C and 5% CO_2_. After incubation, the BRET signals were measured by PHERAstar plate reader following the same procedure as intact NanoBRET measurement. Competitive displacement data were then normalized to controls and was graphed using GraphPad Prism 9 software employing a normalized 3-parameter curve fit with the following equation:

1 $$Y=100/(1+10^{\left(X-{\log IC}_{50}\right)})$$

### K192 NanoBRET Selectivity Screening

To assess the selectivity of compound 10, the K192 Kinase Selectivity System (Promega, cat. no. NP4050) was used.^37^ For plate preparation, a transfection mix was prepared in white 384-well small-volume plates (Greiner, cat. no. 784080) by preplating 3 μL of 20 μL/mL FuGene HD (Promega, cat. no. E2311), diluted in an Opti-MEM medium (Gibco, cat. no. 11058-021). One μL of DNA from both DNA vector source plates of the K192 kit was added using an Echo 550 acoustic dispenser (Beckman Coulter). The mix was incubated for 30 min, and 6 μL of HEK293T cells in an Opti-MEM medium was added. The proteins were allowed to express for 20 h. After expression, Tracer K10 was added using the concentrations recommended in the K192 technical manual and a 1 μM inhibitor was added to every second well. After 2 h of equilibration, detection was carried out using substrate solution comprising Opti-MEM with a 1:166 dilution of a NanoBRET Nano-Glo substrate and a 1:500 dilution of the extracellular NanoLuc inhibitor. Five μL of substrate solution was added to every well, and filtered luminescence was measured on a PHERAstar plate reader (BMG Labtech) equipped with a luminescence filter pair (450 nm BP filter (donor) and a 610 nm LP filter (acceptor)). For every kinase, occupancy was calculated and plotted using GraphPad Prism 10 (Table S5).

### Permeability Studies via Confocal Microscopy

HEK293T were seeded at a density of 3000 cells per well in a 384-well plate (cell culture microplate, PS, f-bottom, μClear, 781091, Greiner) with a final volume of 40 μL and incubated overnight at 37 °C and 5% CO2 to allow for plate attachment. The following day, media was aspirated, cells were washed once with 1× Dulbecco’s Phosphate Buffered Saline (DPBS) and 40 μL of Opti-MEM mixed with Hoechst 33342 (Invitrogen) to a final concentration of 1.62 μM, was added to each well to minimize background fluorescence and to stain the nucleus, respectively. FGMM175 tracer was added at the concentration of 2 μM at 0.5% DMSO using an ECHO 550 acoustic dispenser (Labcyte). Fluorescent activity of tracer was recorded 5 min, after tracer addition while incubating the cells at 37 °C and 5% CO2. Images were acquired using a CQ1 high-content confocal microscope (Yokogawa, Musashino, Japan) with the following setup: Ex 405 nm/Em 447/60 nm (500 ms, 20%) for Hoechst 33342, Ex 561 nm/Em 617/73 nm (500 ms, 20%) for the Tracer and brightfield (50 ms, 20%). Data analysis was performed using CellPathfinder software (Yokogawa).

For the live cell actin staining experiment, Huh-7 were seeded at a density of 1500 cells per well in a 384-well plate (cell culture microplate, PS, f-bottom, μClear, 781091, Greiner) with a final volume of 40 μL and incubated overnight at 37 °C and 5% CO_2_ to allow for plate attachment. The following day, compounds at various concentrations were added using an ECHO 550 acoustic dispenser (Labcyte). After 48 h of treatment, fresh media containing 1× CellMask Orange Actin Tracking Stain (Thermo Fisher) and Hoechst 33342 was replaced and the images were recorded after the plate was incubated for 2 h at 37 °C and 5% CO_2_. Images were acquired using a CQ1 high-content confocal microscope (Yokogawa, Musashino, Japan) with the following setup: Ex 405 nm/Em 447/60 nm for Hoechst 33342, Ex 561 nm/Em 586/20 nm for the Actin Stain. Data analysis was performed using CellPathfinder software (Yokogawa).

### CellTiter-Glo (Promega)

Cell viability was determined using the CellTiter-Glo 2.0 Cell Viability Assay (Promega, G9241) following manufacture’s protocol. 40 μL of Huh-7 and A549 cells at a cell count of 2500 cells/well were seeded into individual wells of white 384-well plate (Corning 3570) and the cells were incubated overnight at 37 °C and 5% CO_2_. Following overnight incubation, serially diluted compounds were titrated on to the plate using an Echo acoustic dispenser (Labcyte) and was incubated for 48 h at 37 °C and 5% CO_2_. Cell viability assay reagent of 20 μL was added to each well and incubated for 10 min at room temperature (RT). Filtered luminescence was measured on a PHERAstar plate reader (BMG Labtech) and data was evaluated using GraphPad Prism 9 software employing a normalized curve fit with the following equation:

2 $$Y=\mathrm{Bottom}+(\mathrm{Top}‐\mathrm{Bottom})/(1+10^{\left((\mathrm{Log}{\mathrm{EC}}_{50}‐X)\times \mathrm{Hillslope}\right)})$$

### Chemistry

The synthetic routes of compounds will be outlined below, and the analytical data can be found in the Supporting Information. Synthetic routes leading to **S1–S16** and conversion analysis of the parallel synthesis approach (**2–40**) can be found in the Supporting Information (Scheme S2). All commercial chemicals were purchased from TCI, BLD Pharm, Enamine, abcr, MCE and Merck with a purity ≥ 95% and were used without further purification. The solvents with an analytical grade were obtained from VWR Chemicals, Honeywell and Merck and all dry solvents from Acros Organics. All reactions were carried out under an argon atmosphere. Parallel synthesis was performed using MonoBlock for 40 × 17 mm reagent vials (Heidolph) using a MR Hei-Tec magnetic stirrer (Heidolph) with attached thermometer without the need for purification. Due to the highly efficient SnAr reaction the reaction mixtures were used directly after solvent evaporation. Thin layer chromatography was carried out using silica gel on aluminum foils (60 Å pore diameter) obtained from Macherey-Nagel and visualized with ultraviolet light in a UV chamber (λ = 254 and 365 nm). The purification of the compounds was performed by flash chromatography using puriFlash XS 420 device with a UV–vis multiwave detector (200–400 nm) from Interchim. Prepacked normal-phase PF-SIHP and prepacked PF-C18HP reverse phase columns with particle sizes of 15 and 30 μm (Interchim) were used for purification. Preparative purification by HPLC was carried out on an Agilent 1260 Infinity II device using an Eclipse XDB-C18 (Agilent, 21.2 × 250 mm, 7 μm) reversed phase column. A gradient was used with or without 0.1% TFA in water (A) and 0.1% TFA in acetonitrile (B) (flow rate 21 mL/min), as a mobile phase. DMSO was evaporated using a SpeedVac Concentrator (ThermoFisher Scientific, Model#: SPD121P-230) coupled to a Savant RVT5105 Refrigerated Vapor Trap (ThermoFisher Scientific) and a VLP80 vacuum pump (ThermoScientific). Nuclear magnetic resonance spectroscopy (NMR) was performed with AV400, AV500, AV600 MHz spectrometers from Bruker. Chemical shifts (δ) are reported in parts per million (ppm). DMSO-d_6_ was used as solvent, and the spectra were calibrated to the solvent signal: 2.50 ppm (^1^H NMR) or 39.52 ppm (^13^C NMR). Coupling constants (*J*) were reported in hertz (Hz) and multiplicities were designated as followed: s (singlet), d (doublet), dd (doublet of doublets), t (triplet), dt (doublet of triplets), td (triplet of doublets), ddd (doublet of doublet of doublets), q (quartet), m (multiplet). Mass spectra were measured on a Surveyor MSQ device from ThermoFisher measuring in the positive- or negative-ion mode. Compounds **1, 54, 55, 68, S13 and S16** were additionally characterized by HRMS using a MALDI LTQ Orbitrap XL from ThermoScientific and a microOTOF-Q ESI source. The purity of the final compounds was determined by HPLC using an Agilent 1260 Infinity II device with a 1260 DAD HS detector (G7117C; 254 nm, 280 nm, 310 nm) and a LC/MSD device (G6125B, ESI pos. 100–1000). The compounds were analyzed on a Poroshell 120 EC-C18 (Agilent, 3 × 150 mm, 2.7 μm) reversed phase column using 0.1% formic acid in water (A) and 0.1% formic acid in acetonitrile (B) as a mobile phase. The following gradient was used: Method 1 (M1): 0 min: 5% B – 2 min: 80% B – 5 min: 95% B – 7 min: 95% B (flow rate of 0.6 mL/min). Method 2 (M2): 0 min: 5% B – 2.8 min: 75% B – 7.2 min: 100% B – 10 min: 100% B (flow rate of 0.6 mL/min). UV-detection was performed at 254 nm and all compounds used for further biological characterization showed a purity ≥95% unless stated otherwise.

### General Procedures

#### General Procedure 1 for SnAr Parallel Synthesis (**2–40**)

Three 50 mM stock solutions in THF containing 4-chloro-6-nitroquinoline-3-carbonitrile (**57**), 4-chloro-7-methoxy-6-nitroquinoline-3-carbonitrile (**58**), 4-chloro-7-ethoxy-6-nitroquinoline-3-carbonitrile (**59**) were prepared. 39 Stock solutions in EtOH containing anilines **AN1**–**AN13** (Figure S4) were prepared. 273 μL of quinoline stock solution in THF was transferred into 40 × 17 mm vials containing a 3 mm stirring bar. 152 μL of aniline stock solution was added to the vial alongside 1.8 eq of 4 N HCl in 1,4-dioxane. The vials were heated to 45 °C on the MonoBlock 16 h. Conversions were monitored using HPLC/MS. Lastly, all solvents were evaporated using a SpeedVac Concentrator.

#### General Procedure 2 for the Assembly of the Quinoline Core (**41–43**)

Step 1 (Cyclization): A solution of aniline derivative (1.0 equiv) and DMF-DMA (3.0 equiv) in 3 mL of DMF was stirred at 150 °C for 3 h. Afterward the solution was diluted with 10 mL DCM and the organic phase was washed with 10 mL H_2_O (3×). The remaining aqueous phase was extracted with 10 mL DCM (2×). The combined organic layers were dried over MgSO4, filtered and the solvent was removed in vacuo. The crude product was obtained as a purple liquid and used without further purification. Thereupon, dry ACN (3.0 equiv) was dissolved in 10 mL of dry THF. The reaction was cooled to −78 °C and *n*-BuLi 2.5 M in hexanes (2.5 equiv) was added dropwise over an hour. The suspension was stirred for 2 h at −78 °C. Afterward, a solution of the imine derivative (1.0 equiv) in 10 mL of dry THF was added dropwise over 30 min at −78 °C. The reaction mixture was stirred at room temperature overnight and quenched by addition of 4 M HCl in 1,4-dioxane (4.0 equiv) and stirred for 2 h. After addition of 25 mL H_2_O, the colorless precipitate was filtered and washed with ACN. The residue was dried in vacuo to give the quinoline as a gray solid.

Step 2 Chlorination: The precipitate formed in the previous step was solved in 8 eq. of POCl_3_ (min 3 mL) and stirred at 100 °C for 2 h to yield the chlorinated derivative.

Step 3 S_N_Ar: The crude reaction mixture from Step 2 (1 equiv) was solved in 10 mL of dry THF and cooled to 0 °C using an ice bath. Four M HCl in dioxane (2.5 equiv) were added dropwise and the solution was stirred for 15 min at 0 °C. Next, 3-chloro-4-fluoroaniline (1.05 equiv) was added to the reaction mixture. The solution was stirred for 16 h at 55 °C. The suspension was filtered, and the light-yellow precipitate was washed with ACN. The residue was dried in vacuo to give the 4-anilinoquinoline derivative.

#### General Procedure 3 for Sonogashira Coupling (**44–45**)

To a solution of iodo-derivative **43** (1.0 equiv), CuI (0.04 equiv) and PdCl_2_(PPh)_3_ (0.02 equiv) in 5 mL dry THF, triethylamine (3.5 equiv) was added and the solution was cooled to 0 °C. Afterward, alkyne derivative (1.1 equiv) was added dropwise and stirred at room temperature for 16 h. The reaction mixture was filtered through a pad of Celite. The filtrate was dried in vacuo and the crude was purified by reverse phase flash column chromatography (H_2_O/ACN 90/10 → 0/100).

#### General Procedure 4 for Ullmann Coupling (**47–53**)

A solution of iodo derivative **43** (1.0 equiv), in 2 mL of DMSO was treated with amine (**A1–A7**) (1.5 equiv), K_2_CO_3_ (3.0 equiv), CuI (0.1 equiv) and l-proline (0.2 equiv) in a sealed tube. The reaction mixture was heated to 80 °C in an oil bath and stirred for 48 h. The reaction mixture was filtered through a pad of Celite. The filtrate was dried in vacuo and the crude was purified by reverse phase flash column chromatography (H_2_O/ACN 90/10 → 0/100).

### N-(4-((3-Chloro-4-fluorophenyl)­amino)-3-cyano-7-ethoxyquinolin-6-yl)-4-(dimethylamino)­butanamide (**1**)

A conical reaction glass vial charged with 3 mm stirring bar and was dried with a heat gun 3× times prior to being used. Commercially available (E)-*N*-(4-((3-chloro-4-fluorophenyl)­amino)-3-cyano-7-ethoxyquinolin-6-yl)-4-(dimethylamino)­but-2-enamide (Pelitinib) (130 mg, 0.28 mmol, 1 equiv) was weighed into the vial and solved in 5 mL Toluene/DMF (1:3) (addition of DMF ensured good solubility). Cu­(OAc)_2_·H_2_O (5.5 mg, 0.02 mmol, 0.1 equiv), 1,2-bis­(diphenylphosphino)­benzene (o-BDPPB/BDP) (12.4 mg, 0.02, 0.1 equiv), Poly­(methylhydrosiloxane) *M*_n_ average 1,700–3,200 (CAS no. 63148-57-2, PMHS) (2 mL, 0.84 mmol, 3 equiv) and *t*-BuOH (27 μL, 0.28 mmol, 3 eq) were added under argon. The reaction vessel was sealed. The reaction turned from blue to yellow in the course of 5–10 min. The mixture was heated to 85 °C for 5 h until completion in an oil bath. The crude product was purified via reverse-phase flash chromatography (H_2_O/ACN: 90/10% → 0/100%) to give compound **1** as a yellow oil.^24^ Yield: 74%. ^1^H NMR (400 MHz, DMSO-d_6_) δ 10.43 (s, 1H), 9.80 (s, 1H), 9.58 (s, 1H), 8.98 (s, 1H), 7.61 (dd, J = 6.6, 2.5 Hz, 1H), 7.49 (t, J = 8.9 Hz, 2H), 7.35 (ddd, J = 8.8, 4.2, 2.6 Hz, 1H), 7.24 (t, J = 7.4 Hz, 1H), 7.17 (d, J = 7.7 Hz, 1H), 4.33 (q, J = 6.9 Hz, 2H), 3.11 (dt, J = 13.1, 5.0 Hz, 2H), 2.79 (d, J = 4.4 Hz, 6H), 2.59 (t, J = 7.3 Hz, 2H), 2.00–1.89 (m, 2H), 1.48 (t, J = 6.8 Hz, 3H) ppm. ^13^C NMR (101 MHz, DMSO-d_6_) δ 171.11, 154.85, 152.31, 150.75, 137.03, 129.38, 129.06, 128.68, 126.97, 125.80, 117.83, 117.61, 116.22, 115.84, 65.51, 56.66, 42.70, 33.05, 21.51, 20.24, 14.59 ppm. *m*/*z* = 470.72 [M + H]^+^. HRMS: *m*/*z* = 470.1768 [M + H]^+^.

### 6-Fluoro-4-hydroxyquinoline-3-carbonitrile (**63**)

The synthesis of compound **63** was performed according to General Procedure 2 (Step 1). Commercially available methyl 2-amino-5-fluorobenzoate **60** (2.00 g, 11.8 mmol, 1.0 equiv) and DMF-DMA (4.7 mL, 35.5 mmol, 3.0 equiv) were reacted. The *N*′-phenylformimidamide intermediate (2.65 g, 11.8 mmol, 1.0 equiv) was obtained as a dark-purple liquid and reacted with ACN (1.9 mL, 35.5 mmol, 3.0 equiv) and *n*-BuLi 2.5 M in hexanes (11.8 mL, 29.58 mmol, 2.5 equiv) to give compound **63**. Yield: 67% (1.47 g, 7.93 mmol). ^1^H NMR (400 MHz, DMSO-d_6_): δ 13.00 (s, 1H, H-11), 8.74 (s, 1H, H-8), 7.88–7.55 (m, 3H, H-2, H-3, H-6) ppm. ^13^C NMR (101 MHz, DMSO-d_6_): δ 173.77, 160.61, 158.18, 146.56, 135.96, 122.33, 116.66, 109.35, 92.92, 39.52 ppm. *m*/*z* = 189.05 [M + H]^+^.

### 4-((3-Chloro-4-fluorophenyl)­amino)-6-fluoroquinoline-3-carbonitrile (**41**)

The synthesis of compound **41** was performed according to General procedure 2 (Steps 2 and 3). **63** (0.82 g, 3.99 mmol, 1.0 equiv) was treated with 3 mL of POCl_3_ and the crude 4-chloro intermediate was mixed with 4 N HCl in dioxane (2.5 mL, 9.9 mmol, 2.5 equiv) and 3-chloro-4-fluoroaniline (578 mg, 3.99 mmol, 1.0 equiv) in 5 mL THF. Compound **41** was obtained as a light-gray solid. Yield: 96% (1.31 g, 3.83 mmol). ^1^H NMR (400 MHz, DMSO-d_6_): δ 11.16 (s, 1H, H-11), 9.00 (s, 1H, H-8), 8.71 (dd, J = 10.6, 2.7 Hz, 1H, H-6), 8.21–8.12 (m, 1H, H-3), 7.98 (ddd, J = 9.2, 7.9, 2.7 Hz, 1H, H-14), 7.77 (dd, J = 6.6, 2.6 Hz, 1H, H-2), 7.57 (t, J = 9.0 Hz, 1H, H-15), 7.52–7.47 (m, 1H, H-18) ppm. ^13^C NMR (101 MHz, DMSO-d_6_): δ 161.60, 159.14, 157.75, 155.29, 153.61, 150.48, 134.92, 128.41, 127.21, 123.69, 119.99, 117.52, 117.30, 114.72, 109.22, 86.91 ppm. *m*/*z* = 315.95 [M + H]^+^.

### 6-Bromo-4-hydroxyquinoline-3-carbonitrile (**64**)

The synthesis of compound **64** was performed according to General Procedure (Step 1). Commercially available methyl 2-amino-5-bromobenzoate **61** (2.00 g, 9.26 mmol, 1.0 equiv) and DMF-DMA (4.3 mL, 32.4 mmol, 3.5 equiv) were reacted. The *N*′-phenylformimidamide intermediate (2.64 g, 9.26 mmol, 1.0 equiv) was obtained as a dark-purple liquid and reacted with ACN (1.5 mL, 27.7 mmol, 3.0 equiv) and *n*-BuLi 2.5 M in hexanes (9.2 mL, 23.15 mmol, 2.5 equiv) to give compound **64** as a gray solid. Yield: 67% (1.54 g, 6.20 mmol). ^1^H NMR (400 MHz, DMSO-d_6_): δ 13.27 (s, 1H, H-11), 8.76 (s, 1H, H-8), 8.18 (d, J = 2.0 Hz, 1H, H-2), 7.93 (dd, J = 8.8, 2.5 Hz, 1H, H-6), 7.69 (dd, J = 8.8, 3.8, 1.8 Hz, 1H, H-3) ppm. ^13^C NMR (101 MHz, DMSO-d_6_): δ 173.29, 146.99, 138.18, 135.99, 127.10, 126.53, 121.89, 118.23, 116.49, 93.93, 39.52 ppm. *m*/*z* = 248.90 [M + H]^+^.

### Synthesis of 6-Bromo-4-((3-chloro-4-fluorophenyl)­amino)­quinoline-3-carbonitrile (**42**)

The synthesis of compound **42** was performed according to General Procedure 2 (Steps 2 and 3). **64** (0.44 g, 1.64 mmol, 1.0 equiv) was treated with 3 mL of POCl_3_ and the 4-chloro intermediate was mixed with 4 N HCl in dioxane (1.0 mL, 4.1 mmol, 2.5 equiv) and 3-chloro-4-fluoroaniline (238 mg, 1.64 mmol, 1.0 equiv) in 10 mL THF. Compound **42** was obtained as a light-gray solid. Yield: 64% (430 mg, 0.984 mmol). ^1^H NMR (400 MHz, DMSO-d_6_) δ 11.52 (s, 1H), 9.17 (s, 1H), 9.06 (s, 1H), 8.19 (s, 1H), 8.06 (d, J = 8.7 Hz, 1H), 7.78 (s, 1H), 7.57 (d, J = 8.9 Hz, 1H), 7.51 (s, 1H) ppm. ^13^C NMR (101 MHz, DMSO-d_6_) δ 153.92, 151.32, 137.72, 128.97, 127.77, 127.21, 125.72, 121.48, 120.52, 117.74, 40.36 ppm. *m*/*z* = 377.95 [M + H]^+^.

### 4-Hydroxy-6-iodoquinoline-3-carbonitrile (**65**)

The synthesis of compound **65** was performed according to General Procedure (Step 1). Commercially available methyl 2-amino-5-iodobenzoate **62** (2.00 g, 7.60 mmol, 1.0 equiv) and DMF-DMA (3.1 mL, 22.8 mmol, 3.0 equiv) were reacted. The *N*′-phenylformimidamide intermediate (2.52 g, 7.60 mmol, 1.0 equiv) was obtained as a dark-purple liquid and reacted with ACN (1.6 mL, 22.8 mmol, 3.0 equiv) and *n*-BuLi 2.5 M in hexanes (7.6 mL, 19.0 mmol, 2.5 equiv) to give compound **65** as a gray solid. Yield: 91% (2.04 g, 6.92 mmol). 1H NMR (400 MHz, DMSO-d_6_): δ 12.92 (s, 1H, H-11), 8.76 (s, 1H, H-8), 8.39 (d, J = 2.0 Hz, 1H, H-6), 8.06 (dd, J = 8.7, 2.1 Hz, 1H, H-2), 7.44 (d, J = 8.7 Hz, 1H, H-3) ppm. ^13^C NMR (101 MHz, DMSO-d_6_): δ 73.08, 147.04, 141.39, 138.41, 133.32, 126.69, 121.53, 116.50, 94.03, 90.60 ppm. *m*/*z* = 296.95 [M + H]^+^.

### 4-((3-Chloro-4-fluorophenyl)­amino)-6-iodoquinoline-3-carbonitrile (**43**)

The synthesis of compound **43** was performed according to General Procedure 2 (Steps 2 and 3). **65** (1.06 g, 3.38 mmol, 1.0 equiv) was treated with 3 mL of POCl_3_ and the crude 4-choloro intermediate was mixed with 4 N HCl in dioxane (2.1 mL, 8.5 mmol, 2.5 equiv) and 3-chloro-4-fluoroaniline (490 mg, 3.38 mmol, 1.0 equiv) in 10 mL THF. Compound **43** was obtained as a light-gray solid. Yield: 87% (1.35 g, 2.94 mmol). ^1^H NMR (400 MHz, DMSO-d_6_): δ 11.38 (s, 1H, H-11), 9.23 (s, 1H, H-8), 9.01 (s, 1H, H-6), 8.30 (dd, J = 8.8, 1.7 Hz, 1H, H-2), 7.86 (d, J = 8.8 Hz, 1H, H-3), 7.77 (dd, J = 6.7, 2.5 Hz, 1H, H-18), 7.56 (t, J = 9.0 Hz, 1H, H-14), 7.52–7.46 (m, 1H, H-15) ppm. ^13^C NMR (101 MHz, DMSO-d_6_): δ 158.22, 155.77, 153.44, 151.43, 142.94, 135.40, 133.00, 128.85, 127.64, 125.76, 120.79, 120.23, 117.96, 117.74, 115.09, 94.66, 87.64, 66.83, 40.21 ppm. *m*/*z* = 423.85 [M + H]^+^.

### 4-((3-Chloro-4-fluorophenyl)­amino)-6-((trimethylsilyl)­ethynyl)­quinoline-3-carbonitrile (**66**)

The synthesis of compound **66** was performed according to General Procedure 3. By utilizing compound **43** (250 mg, 0.545 mmol, 1.1 equiv), ethynyltrimethylsilane (69 μL, 0.49 mmol, 1.0 equiv), PdCl_2_(PPh_3_)_2_ (7 mg, 0.01 mmol, 0.02 equiv), CuI (2 mg, 0.020 mmol, 0.04 equiv) and triethylamine (241 μL, 1.73 mmol, 3.5 equiv) compound **66** was obtained a yellow oil. Yield: 51% (99 mg, 0.25 mmol). ^1^H NMR (400 MHz, DMSO-d_6_): δ 8.67 (s, 1H, H-8), 8.03 (s, 1H, H-6), 8.00–7.93 (m, 1H, H-2), 7.80 (d, J = 7.6 Hz, 1H, H-3), 7.30 (dd, J = 6.3, 2.6 Hz, 2H, H-18), 7.18 (t, J = 8.5 Hz, 1H, H-14), 7.14–7.07 (m, 1H, H-15), 0.24 (d, J = 1.3 Hz, 9H, H-24, H-25, H-26) ppm. ^13^C NMR (101 MHz, DMSO-d_6_): δ 158.27, 155.79, 152.99, 150.31, 148.71, 135.32, 135.09, 130.68, 127.27, 125.10, 122.35, 117.65, 117.43, 116.21, 103.64, 97.61, −0.10 ppm. *m*/*z* = 394.10 [M + H]^+^.

### 4-((3-Chloro-4-fluorophenyl)­amino)-6-ethynylquinoline-3-carbonitrile (**44**)

Compound **66** (70 mg, 0.18 mmol, 1.0 equiv) was solved in 2 mL of THF. Afterward, TBAF (0.3 mL, 0.3 mmol, 1.7 equiv) was added and the solution was stirred at room temperature for 1 h. The solution was diluted with 10 mL of EtOAc and the organic phase was washed with 10 mL of saturated NaHCO_3_ solution (2×). The remaining aqueous phase was extracted with 10 mL of EtOAc (2×). The combined organic layers were dried over MgSO_4_, filtered and the solvent was removed in vacuo to give compound **44** as a colorless solid. Yield: 84% (48 mg, 0.15 mmol). ^1^H NMR (400 MHz, DMSO-d_6_) δ 10.00 (s, 1H), 8.64 (d, J = 22.5 Hz, 2H), 7.89 (q, J = 8.5 Hz, 2H), 7.60 (d, J = 4.7 Hz, 1H), 7.47 (t, J = 9.0 Hz, 1H), 7.37 (s, 1H), 4.43 (s, 1H) ppm. ^13^C NMR (101 MHz, DMSO-d_6_) δ 150.72, 134.33, 126.97, 119.72, 119.53, 117.22, 117.00, 116.63, 82.35 ppm. *m*/*z* = 321.90 [M + H]^+^.

### Tert-Butyl (3-(4-((3-Chloro-4-fluorophenyl)­amino)-3-cyanoquinolin-6-yl)­prop-2-yn-1-yl)­carbamate (**67**)

The synthesis of compound **67** was performed according to General Procedure 3. By utilizing compound **43** (250 mg, 0.545 mmol, 1.1 equiv), *tert*-butyl prop-2-yn-1-ylcarbamate (77 mg, 0.49 mmol, 1.0 equiv), PdCl_2_(PPh3)_2_ (7 mg, 0.01 mmol, 0.02 equiv), CuI (2 mg, 0.020 mmol, 0.04 equiv) and triethylamine (241 μL, 1.73 mmol, 3.5 equiv) compound **67** was obtained with a yield of 83% (185 mg, 0.411 mmol). The compound was used as it is in the next step. *m*/*z* = 451.10 [M + H]^+^.

### 6-(3-Aminoprop-1-yn-1-yl)-4-((3-chloro-4-fluorophenyl)­amino)­quinoline-3-carbonitrile (**45**)

Compound **67** (163 mg, 0.363 mmol) was solved in 1 mL of DCM. Afterward, 0.5 mL of TFA were added and the solution was stirred at room temperature for 1 h. The reaction mixture was diluted with 2 mL of toluene and all solvents were removed in vacuo. The crude product was purified via reverse-phase flash chromatography ((H_2_O/ACN) + 0.1% TFA: 95/5% → 0/100%) to give compound **45** as a yellow solid. Yield: 99% (127 mg, 0.360 mmol). ^1^H NMR (400 MHz, DMSO-d_6_): δ 10.32 (s, 1H, H-11), 8.75–8.71 (m, 1H, H-8), 8.70–8.66 (m, 1H, H-6), 8.46 (s, 2H, H-24), 7.96 (d, J = 8.7 Hz, 1H, H-2), 7.88 (d, J = 8.7 Hz, 1H, H-3), 7.66–7.59 (m, 1H, H-18), 7.50 (t, J = 8.9 Hz, 1H, H-14), 7.40–7.34 (m, 1H, H-15), 4.10 (d, J = 4.9 Hz, 2H, H-23) ppm. ^13^C NMR (101 MHz, DMSO-d_6_): δ 135.11, 129.38, 128.69, 127.55, 127.47, 126.47, 125.80, 120.30, 120.11, 119.95, 117.82, 117.61, 116.59, 114.66, 88.54, 85.26, 84.89, 29.51, 21.51 ppm. *m*/*z* = 351.00 [M + H]^+^.

### 3-((2-Aminoethyl)­thio)-*N*-(4-((3-chloro-4-fluorophenyl)­amino)-3-cyano-7-ethoxyquinolin-6-yl)-4-(dimethylamino)­butanamide (**46**)

Commercially available (E)-*N*-(4-((3-chloro-4-fluorophenyl)­amino)-3-cyano-7-ethoxyquinolin-6-yl)-4-(dimethylamino)­but-2-enamide (Pelitinib) (20 mg, 43 μmol, 1 equiv), 2-(mercaptoethyl)­carbamate (18 mg ≙ 18.8 μL, 112 μmol, 2.6 equiv) and triethylamine (2.9 μL, 21 μmol, 0.5 equiv) were solved in 1 mL of dry DMSO and charged in a coned glass vial and sealed. The reaction mixture was transferred into a microwave reactor and heated to 65 °C for 16 h until completion of the reaction (monitored by HPLC/MS). Solvents were evaporated in a Speed Vac concentrator. The crude was solved in dry DCM. TFA (1 mL) and triethylsilane (TES) (1 mL) were added and let stir for 2 h until full conversion. Solvents were removed in vacuo and the crude was directly purified via purified via reverse-phase flash chromatography (H_2_O/ACN 98/2% → 0/100%) to give compound **46** as yellow oil. Yield: 52% over two steps (12 mg, 22 μmol). ^1^H NMR (400 MHz, DMSO-d_6_) δ 10.50 (s, 1H), 9.75 (s, 1H), 9.53 (s, 1H), 8.95 (s, 1H), 8.83 (s, 1H), 8.05 (s, 3H), 7.61 (dd, J = 6.7, 2.7 Hz, 1H), 7.51 (t, J = 4.4 Hz, 1H), 7.41–7.26 (m, 1H), 7.27–7.12 (m, 1H), 4.34 (q, J = 6.9 Hz, 2H), 3.47 (ddd, J = 40.0, 15.8, 7.7 Hz, 3H), 3.04 (dd, J = 16.9, 9.7 Hz, 3H), 2.99–2.90 (m, 2H), 2.86 (d, J = 2.9 Hz, 6H), 1.47 (t, J = 6.9 Hz, 3H), 1.22 (*n*-hexanes) ppm. ^13^C NMR (101 MHz, DMSO-d_6_) δ 170.30, 155.93, 152.34, 150.50, 148.20, 138.45, 128.71, 124.96, 123.72, 117.63, 117.41, 114.43, 109.11, 89.64, 78.14, 65.12, 45.71, 40.86, 28.67, 14.73, 1.63 ppm. *m*/*z* = 545.15 [M + H]^+^.

### *N*-(4-((3-Chloro-4-fluorophenyl)­amino)-3-cyanoquinolin-6-yl)­pent-4-ene-1-sulfonamide (**47**)

The synthesis of compound **47** was performed according to General Procedure 4. Compound **43** (100 mg, 0.21 mmol, 1.0 equiv) was reacted with pent-4-ene-1-sulfonamide (48 mg, 0.32 mmol, 1.5 equiv), l-proline (5 mg, 0.042 mmol, 0.2 equiv), CuI (5 mg, 0.02 mmol, 0.1 equiv) and potassium carbonate (90 mg, 0.65 mmol, 3.0 equiv). Compound **47** was obtained as a orange-yellow oil. Yield: 29% (27 mg, 0.06 mmol). ^1^H NMR (400 MHz, DMSO-d_6_) δ 10.87 (s, 1H), 8.86 (s, 1H), 8.50 (d, J = 9.2 Hz, 1H), 7.77–7.67 (m, 2H), 7.53 (ddd, J = 9.0, 5.4, 3.3 Hz, 2H), 7.44 (ddd, J = 8.8, 4.3, 2.6 Hz, 1H), 5.74 (ddt, J = 16.9, 10.2, 6.6 Hz, 1H), 5.03–4.88 (m, 2H), 3.33–3.29 (m, 2H), 2.12 (q, J = 7.2 Hz, 2H), 1.85–1.74 (m, 2H) ppm. ^13^C NMR (101 MHz, DMSO-d_6_) δ 152.82, 151.93, 144.44, 143.51, 137.12, 135.58, 128.00, 126.82, 125.39, 118.85, 117.46, 117.24, 115.94, 115.39, 114.09, 110.07, 86.50, 50.72, 40.43, 31.13, 22.34 ppm. *m*/*z* = 445.00 [M + H]^+^.

### *tert*-Butyl (1-(4-((3-Chloro-4-fluorophenyl)­amino)-3-cyanoquinolin-6-yl)-azetidin-3-yl)­carbamate (**48**)

The synthesis of compound **48** was performed according to General Procedure 4. Compound **43** (200 mg, 0.435 mmol, 1.0 equiv) was reacted with *tert*-butyl azetidin-3-ylcarbamate (113 mg, 0.653 mmol, 1.5 equiv), l-proline (10 mg, 0.087 mmol, 0.2 equiv), CuI (9 mg, 0.04 mmol, 0.1 equiv) and potassium carbonate (180 mg, 1.30 mmol, 3.0 equiv). Compound **48** was obtained as a yellow solid yield of 55% (111 mg, 0.249 mmol). ^1^H NMR (400 MHz, DMSO-d_6_) δ 10.36 (s, 1H), 8.72 (s, 1H), 7.79 (d, J = 9.0 Hz, 1H), 7.74 (dd, J = 6.5, 2.0 Hz, 1H), 7.55 (t, J = 8.9 Hz, 1H), 7.46 (d, J = 11.2 Hz, 1H), 7.41 (d, J = 10.2 Hz, 2H), 7.05 (s, 1H), 4.30 (s, 2H), 3.94 (d, J = 36.0 Hz, 1H), 3.69 (s, 2H), 1.38 (s, 9H) ppm. ^13^C NMR (101 MHz, DMSO-d_6_) δ 146.92, 145.59, 124.93, 120.32, 117.47, 117.25, 97.58, 78.78, 42.14, 39.52, 28.03 ppm. *m*/*z* = 468.05 [M + H]^+^.

### *tert*-Butyl (*S*)-(1-(4-((3-Chloro-4-fluorophenyl)­amino)-3-cyanoquinolin-6-yl)-pyrrolidin-3 yl)­carbamate (**49**)

The synthesis of compound **49** was performed according to General Procedure 4. Compound **43** (200 mg, 0.435 mmol, 1.0 equiv) was reacted with *tert*-butyl (*S*)-pyrrolidin-3-ylcarbamate (121 mg, 0.653 mmol, 1.5 equiv), l-proline (10 mg, 0.087 mmol, 0.2 equiv), CuI (9 mg, 0.04 mmol, 0.1 equiv) and potassium carbonate (180 mg, 1.30 mmol, 3.0 equiv). Compound **49** was obtained as a yellow oil. Yield: 27% (54 mg, 0.12 mmol). ^1^H NMR (400 MHz, DMSO-d_6_) δ 10.71 (s, 1H), 8.79 (s, 1H), 7.85 (d, J = 9.1 Hz, 1H), 7.80 (d, J = 6.4 Hz, 1H), 7.58 (t, J = 8.9 Hz, 1H), 7.52 (d, J = 3.9 Hz, 1H), 7.46 (d, J = 10.6 Hz, 1H), 7.30 (s, 2H), 4.22 (d, J = 6.6 Hz, 1H), 3.70–3.59 (m, 1H), 3.55 (d, J = 6.0 Hz, 1H), 3.46–3.34 (m, 1H), 3.22 (d, J = 6.0 Hz, 1H), 2.30–2.16 (m, 1H), 2.06–1.90 (m, 1H), 1.40 (s, 9H) ppm. ^13^C NMR (101 MHz, DMSO-d_6_) δ 155.32, 152.40, 147.08, 144.50, 128.72, 128.71, 122.21, 117.53, 99.81, 77.99, 53.94, 49.92, 46.31, 39.52, 30.29, 28.24 ppm. *m*/*z* = 482.10 [M + H]^+^.

### 4-((3-Chloro-4-fluorophenyl)­amino)-6-(piperazin-1-yl)­quinoline-3-carbo-nitrile (**50**)

Compound **S1** (10 mg, 0.021 mmol) was solved in 1 mL of DCM. Afterward, 0.5 mL of TFA were added and the solution was stirred at room temperature for 1 h. After characterization via LC-MS, the reaction mixture was diluted with 2 mL of toluene, and the solvent was removed in vacuo. The crude product was purified using reverse phase chromatography (H_2_O/ACN) 98/2% → 0/100%) to yield **50** as yellow oil. Yield: 85%. ^1^H NMR (300 MHz, DMSO-d_6_): δ 8.25 (s, 1H, H-8), 7.69 (d, J = 9.1 Hz, 1H, H-3), 7.62 (d, J = 2.6 Hz, 1H, H-6), 7.59–7.53 (m, 1H, H-14), 7.41– 7.33 (m, J = 10.7, 7.2 Hz, 2H, H-2, H-15), 7.22–7.14 (m, 1H, H-18), 3.21 (t, J = 5.0 Hz, 4H, H-23, H-25), 2.86 (t, J = 5.1 Hz, 4H, H-22, H-26) ppm. ^13^C NMR (101 MHz, DMSO-d_6_): δ 159.13, 154.53, 151.06, 139.73, 136.78, 134.29, 133.19, 133.14, 130.80, 127.22, 124.88, 122.48, 119.26, 111.35, 91.04, 50.14, 47.34 ppm. *m*/*z* = 382.10 [M + H]^+^.

### 4-((3-Chloro-4-fluorophenyl)­amino)-6-(piperazin-1-yl)­quinoline-3-carbo-nitrile (**51**)

Propiolic acid (1.2 μL, 0.019 mmol, 1.2 equiv) and HATU (7.4 mg, 0.02 mmol, 1.3 equiv) were dissolved in 1 mL of DMF. DIPEA (7.8 μL, 0.045 mmol, 3.0 equiv) was added and the reaction mixture was stirred at room temperature for 15 min. 4-((3-chloro-4-fluorophenyl)­amino)-6-(piperazin-1-yl)­quinoline-3-carbonitrile (**50**) (6 mg, 0.015 mmol, 1.0 equiv) was added to the solution and the reaction mixture was stirred for 1 h. The solvent was removed in vacuo and the crude product was purified via reverse-phase flash chromatography ((H_2_O/ACN + 0.1% TFA): 90/10 → 0/100). Yield: 60% (3.7 mg, 3.9 mg). ^1^H NMR (300 MHz, DMSO-d_6_) δ 9.75 (s, 1H), 8.43 (s, 1H), 7.85 (d, J = 9.0 Hz, 1H), 7.72 (d, J = 11.2 Hz, 2H), 7.58 (dd, J = 6.7, 2.7 Hz, 1H), 7.48 (t, J = 9.0 Hz, 1H), 7.40–7.28 (m, 2H), 4.65 (s, 1H), 3.90 (s, 2H), 3.71 (s, 2H), 3.45 (s, 4H). *m*/*z* = 434.10 [M + H]^+^.

### Tert-Butyl ((1*S*,4*S*)-4-((4-((3-Chloro-4-fluorophenyl)­amino)-3-cyanoquinolin-6-yl)­amino)­cyclohexyl)­carbamate (**52**)

The synthesis of compound **52** was performed according to General Procedure 4. Compound **43** (200 mg, 0.435 mmol, 1.0 equiv) was reacted with *tert*-butyl ((1*S*,4*S*)-4-aminocyclohexyl)­carbamate (140 mg, 0.653 mmol, 1.5 equiv), l-proline (10 mg, 0.087 mmol, 0.2 equiv), CuI (9 mg, 0.04 mmol, 0.1 equiv) and potassium carbonate (180 mg, 1.301 mmol, 3.0 equiv). Compound **52** was obtained as a yellow solid. Yield: 54% (111 mg, 0.244 mmol). ^1^H NMR (400 MHz, DMSO-d_6_) δ 10.62 (s, 1H), 8.79 (s, 1H), 7.84 (s, 1H), 7.81 (s, 1H), 7.73 (d, J = 9.3 Hz, 1H), 7.55 (d, J = 23.0 Hz, 3H), 7.21 (s, 1H), 6.74 (d, J = 8.8 Hz, 1H), 3.56 (s, 1H), 3.42 (s, 1H), 1.78–1.54 (m, 8H), 1.39 (s, 9H) ppm. ^13^C NMR (101 MHz, DMSO-d_6_) δ 158.49, 158.15, 125.79, 120.50, 117.30, 115.04, 97.11, 48.04, 39.52, 28.30, 27.06, 26.48, 25.45 ppm. *m*/*z* = 510.15 [M + H]^+^.

### N-(4-((3-Chloro-4-fluorophenyl)­amino)-3-cyanoquinolin-6-yl)-4-nitrobenzamide (**53**)

The synthesis of compound **53** was performed according to General Procedure 4. Compound **43** (100 mg, 0.21 mmol, 1.0 equiv) was reacted with 4-nitrobenzamide (52 mg, 0.32 mmol, 1.5 equiv), l-proline (5 mg, 0.042 mmol, 0.2 equiv), CuI (5 mg, 0.02 mmol, 0.1 equiv) and potassium carbonate (90 mg, 0.65 mmol, 3.0 equiv). Compound **53** was obtained as a yellow oil. Yield: 34% (33 mg, 0.07 mmol). ^1^H NMR (400 MHz, DMSO-d_6_) δ 11.09 (s, 1H), 10.28–9.96 (m, 1H), 8.69 (s, 1H), 8.52 (d, J = 2.1 Hz, 1H), 8.47 (d, J = 9.2 Hz, 1H), 8.44–8.37 (m, 2H), 8.29–8.19 (m, 2H), 8.04 (dd, J = 9.2, 2.2 Hz, 1H), 7.67–7.59 (m, 1H), 7.49 (t, J = 9.0 Hz, 1H), 7.38 (dd, J = 8.0, 4.1 Hz, 1H) ppm. ^13^C NMR (101 MHz, DMSO-d_6_) δ 165.19, 129.96, 124.15 ppm. *m*/*z* = 462.05 [M + H]^+^.

### 4-((3,5-Dibromo-4-fluorophenyl)­amino)-6-(piperazin-1-yl)­quinoline-3-carbonitrile (**54**)

Compound **S2** (250 mg, 0.710 mmol, 1.0 equiv) and piperazine (185 mg, 2.13 mmol, 3.0 equiv) were solved in 2 mL of ethanol in a microwave vial. DIPEA (495 μL, 2.82 mmol, 4.0 equiv) was added and the vial was sealed. The microwave assisted reaction was stirred for 10 h at 120 °C. Afterward, the solvent was removed in vacuo and the crude was purified via reverse-phase flash chromatography ((H_2_O/ACN 98/2%) + 0.1% TFA → 0/100%) to give compound **54** as a yellow solid. Yield: 30% (105 mg, 0.213 mmol). ^1^H NMR (500 MHz, DMSO-d_6_) δ 10.55 (s, 1H), 9.17 (s, 2H), 8.81 (d, J = 2.5 Hz, 1H), 8.42–8.33 (m, 1H), 7.77 (dd, J = 5.8, 2.3 Hz, 2H), 7.59 (dd, J = 9.5, 2.6 Hz, 1H), 7.26 (q, J = 2.3 Hz, 1H), 3.71 (t, J = 5.3 Hz, 4H), 3.29 (s, 4H) ppm. ^13^C NMR (126 MHz, DMSO-d_6_) δ 158.73, 154.66, 153.49, 152.28, 151.73, 137.67, 129.45, 129.38, 128.68, 125.49, 118.03, 116.30, 116.10, 111.75, 109.50, 109.31, 86.94, 44.34, 42.77 ppm. *m*/*z* = 506.05 [M + H]^+^ (Br isotope). HRMS: *m*/*z* = 503.9832 [M + H]^+^.

### 4-((3-Chloro-4-fluorophenyl)­amino)-7-(piperazin-1-yl)­quinoline-3-carbonitrile (**55**)

Compound **S3** (200 mg, 0.435 mmol, 1.0 equiv) was solved in 3 mL of DMSO and reacted with *tert*-butyl *tert*-butyl piperazine-1-carboxylate (121 mg, 0.653 mmol, 1.5 equiv), l-proline (10 mg, 0.087 mmol, 0.2 equiv), CuI (9 mg, 0.04 mmol, 0.1 equiv) and potassium carbonate (180 mg, 1.30 mmol, 3.0 equiv) in a sealed conic glass vial for 48 h at 80 °C. The crude was solved in DCM and filtered through a pad of Celite. The filtrate was concentrated and washed 3× with 10 mL of water. The organic phase was collected and dried over MgSO_4_ and solvents were removed in vacuo. The crude was solved in DCM (5 mL) and TFA (3 mL) was added. The mixture was stirred for 4 h until completion. All solvents were removed in vacuo (coevaporation with toluene). The crude compound was purified using reverse phase column chromatography (H_2_O/ACN 98/2% → 0/100%). Compound **55** was obtained as a yellow oil. Yield: 20% (33 mg, 0.09 mmol). ^1^H NMR (500 MHz, DMSO-d_6_) δ 10.80 (s, 1H), 9.33 (s, 2H), 8.77 (s, 1H), 7.92 (d, J = 9.1 Hz, 1H), 7.85 (d, J = 9.7 Hz, 2H), 7.74 (dd, J = 6.8, 2.5 Hz, 1H), 7.54 (t, J = 8.9 Hz, 1H), 7.47 (dt, J = 8.2, 3.4 Hz, 1H), 3.63 (t, J = 5.1 Hz, 4H), 3.31 (s, 4H). ^13^C NMR (126 MHz, DMSO-d_6_) δ 158.90, 158.64, 157.21, 155.25, 152.41, 149.16, 147.74, 136.44, 135.57, 135.55, 128.13, 126.92, 126.86, 125.43, 125.05, 120.00, 119.85, 119.70, 117.83, 117.44, 117.27, 115.48, 115.32, 105.09, 86.87, 45.13, 42.38. *m*/*z* = 382.10 [M + H]^+^. HRMS: *m*/*z* = 382.1222 [M + H]^+^.

### Synthesis of 5-Azido-N-(4-((3-chloro-4-fluorophenyl)­amino)-3-cyanoquinolin-7-yl)-pentanamide (**56**)

Compound **S7** (10 mg, 0.023 mmol, 1.0 equiv) and sodium azide (7.5 mg, 0.12 mmol, 5.0 equiv) were solved in 0.5 mL of DMF and heated to 80 °C for 24 h in a round-bottom flask. The solution was diluted with 10 mL of H_2_O and the product was extracted with 10 mL of DCM (3×). The combined organic layers were washed with 10 mL of H_2_O (2×), dried over MgSO_4_, filtered and the solvent was removed in vacuo. The crude product was purified via reverse-phase flash chromatography (H_2_O/ACN: 90/10% → 0/100%) to give compound **56** as a brown solid with a yield of 30% (3.0 mg, 0.006 mmol). ^1^H NMR (300 MHz, DMSO-d_6_) δ 10.47 (s, 1H), 9.86 (s, 1H), 8.55 (s, 1H), 8.45–8.34 (m, 1H), 8.30 (d, J = 2.1 Hz, 1H), 7.78 (d, J = 9.3 Hz, 1H), 7.54 (d, J = 6.5 Hz, 1H), 7.44 (t, J = 8.9 Hz, 1H), 2.44 (d, J = 7.2 Hz, 2H), 1.86–1.76 (m, 2H), 1.75–1.57 (m, 4H) ppm. *m*/*z* = 438.10 [M + H]^+^.

### *N*-(15-(4-(4-((3-Chloro-4-fluorophenyl)­amino)-3-cyanoquinolin-6-yl)-piperazin-1-yl)-15-oxo-3,6,9,12-tetraoxapentadecyl)-3-(5,5-difluoro-7-(1H-pyrrol-2-yl)-5H-5λ4,6λ4-dipyrrolo­[1,2-c:2″,1″-F]­[1,3,2]­diazaborinin-3-yl)­propenamide (**68**)

2,5-Dioxopyrrolidin-1-yl-3-(5,5-difluoro-7-(1H-pyrrol-2-yl)-5H-5λ4,6λ4-dipyrrolo­[1,2-c:2″,1″-f]­[1,3,2]­diazaborinin-3-yl)­propanoate (5.2 mg, 0.012 mmol, 1.0 equiv) and compound **S9** (8.6 mg, 0.014 mmol, 1.1 equiv) were dissolved in 1 mL of DMF. DIPEA (6.5 μL, 0.037 mmol, 3.0 equiv) was added and the reaction mixture was stirred for 1 h. The solvent was removed in vacuo and the crude product was purified via reverse-phase flash chromatography ((H_2_O/ACN + 0.2% TFA): 98/2% → 0/100%) to give compound **68** as a dark-purple solid. Yield: 32% (3.7 mg, 0.0038 mmol). ^1^H NMR (500 MHz, DMSO-d_6_): δ 11.42 (s, 1H), 10.64 (s, 1H), 8.78 (s, 1H), 8.01 (t, J = 5.6 Hz, 1H), 7.95 (s, 0H), 7.88–7.79 (m, 2H), 7.76–7.70 (m, 2H), 7.55 (td, J = 8.9, 1.3 Hz, 1H), 7.49–7.44 (m, 1H), 7.42 (s, 1H), 7.36 (s, 1H), 7.32 (d, J = 4.5 Hz, 1H), 7.26 (s, 1H), 7.16 (d, J = 4.5 Hz, 1H), 7.00 (d, J = 3.9 Hz, 1H), 6.39–6.24 (m, 2H), 3.65 (t, J = 6.4 Hz, 7H), 3.52–3.46 (m, 11H), 3.45–3.43 (m, 1H), 3.41 (t, J = 5.9 Hz, 1H), 3.39–3.37 (m, 2H), 3.22 (q, J = 5.8 Hz, 2H), 3.13 (t, J = 7.8 Hz, 2H), 2.89 (s, 1H), 2.73 (s, 1H), 2.64 (t, J = 6.5 Hz, 2H), 2.55 (t, J = 5.5 Hz, 1H) ppm. ^13^C NMR (126 MHz, DMSO-d_6_): δ 171.08, 169.10, 162.34, 157.27, 155.89, 155.30, 152.38, 150.23, 149.75, 146.95, 136.94, 135.36, 133.00, 132.41, 128.26, 127.07, 126.74, 126.11, 124.85, 124.55, 124.37, 122.89, 119.70, 119.35, 117.41, 116.13, 115.16, 111.52, 104.04, 86.60, 69.76, 69.57, 69.09, 66.77, 47.80, 47.34, 44.42, 40.47, 38.62, 35.78, 34.31, 33.79, 32.78, 30.77, 24.02 ppm. ^19^F NMR (471 MHz, DMSO-d_6_): δ −74.14, −117.66, −142.15.*m*/*z* = 940.30 [M + H]^+^, HRMS: *m*/*z* = 962.35050 [M + Na]^+^.

## Supplementary Material











## Notes

Figure S1: altered sequence features of the ILK pseudokinase domain (zoomed-in view of ILK); Figure S2: structural and biophysical evaluation and selectivity mechanisms; Figure S3: development of NanoBRET tracer system and NanoBRET based selectivity data; Figure S4: effect on actin cytoskeleton organization and cell titer glow viability assay; Figure S5: anilines used in the study; Table S1: data collection and refinement statistics; Scheme S1: failed reaction and synthesis limitations; and Scheme S2: supplementary procedures (PDF)
K192 NanoBRET selectivity screening (CSV)
Molecular formula strings file (CSV)
The DSF screening data (XLSX)
The spectral data (PDF)

## Abbreviations

*ΔT*_*m*_: change in melting temperature
CH2: Calponin Homology 2
EN300: EN300-6740186
FAK: focal adhesion kinase
GSK-3β: glycogen synthase kinase-3β
ILK: integrin-linked kinase
MD: molecular dynamics
MerTK: MER tyrosine kinase
MMS: Matrix Screening
parvin: actin-linked protein associated with focal adhesion
PINCH: particularly interesting new cysteine-histidine-rich protein
ANK: ankyrin repeat
PKD: pseudo kinase domain
PROTAC: proteolysis-targeting chimera
RMSD: root-mean-square deviation
SPR: surface plasmon resonance
SRC: sarcoma (proto-oncogene tyrosine-protein kinase Src)
WH2: winged helix 2
*T*_*m*_: melting temperature
