# Supplementary material for: Identification and Validation of 3‑Cyano-Quinoline Ligands Targeting Integrin-Linked Kinase (ILK)
Source: J Med Chem. 2026 May 25;69(11):12982–3001. doi: 10.1021/acs.jmedchem.5c03773 (PMC13266989; doi:10.1021/acs.jmedchem.5c03773)
Supplement: Supplementary file 1 [file jm5c03773_si_001.pdf]

Supplementary information (SI)

Identification and Validation of 3-Cyano-quinoline Ligands Targeting Integrin-Linked Kinase (ILK)

Francesco Aleksy Greco,[1,2,3,†] Kamal Rayees Abdul Azeez,[1,2,†] Marko Mitrović,[1,2] Saran Aswathaman Sivashanmugam,[1,2] Martin Peter Schwalm,[1,2,3] Franziska Preuss,[1,2] Deep Chatterjee,[1,2] Viktoria Morasch,[1,2] Sebastian Mathea,[1,2] Thomas Hanke, [1,2] Susanne Müller,[1,2] and Stefan Knapp[1,2,3,*]

[1]Institute of Pharmaceutical Chemistry, Goethe University Frankfurt, Max-von-Laue-Str. 9, 60438 Frankfurt am Main, Germany

[2]Structural Genomics Consortium (SGC), Buchmann Institute for Molecular Life Sciences (BMLS), Max-von-Laue-Str. 15, 60438 Frankfurt am Main, Germany

[3]German Cancer Research Center (DKFZ), Im Neuenheimer Feld 280, 69120 Heidelberg, Germany

[†] These authors contributed equally

*Correspondence: knapp@pharmchem.uni-frankfurt.de

TABLE OF CONTENTS

SECTIONS		PAGE
**SCHEMES AND FIGURES**		
**Figures**	**Title / Description**	
Figure S1	Altered sequence features of the ILK pseudokinase domain.	S2
Figure S2	Structural and Biophysical Evaluation, and Selectivity Mechanisms	S3
Figure S3	Development and Validation of a NanoBRET Tracer System for ILK	S6
Figure S4	Effect on actin cytoskeleton organization and cell titer glow viability assay	S11
**Tables**		
Table1	Data Collection and Refinement Statistics	S12
**Chemistry schemes, figures, and procedures**		
Scheme S1	Failed Reactions and Synthesis Limitations	S13
Scheme S2	Supplementary Procedures	S17
Figure S5	Anilines used in the study	S26
**REFERENCES**		S26

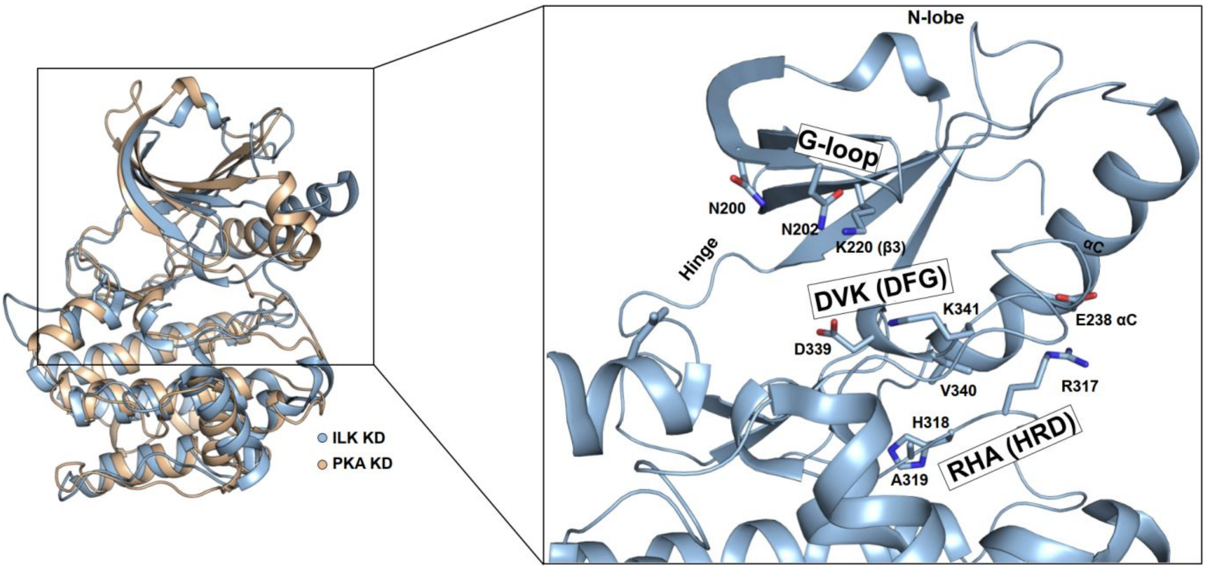

Figure S1. Altered sequence features of the ILK pseudokinase domain (zoomed-in view of ILK). Key altered motifs in ILK are highlighted, and the corresponding conserved canonical kinase motifs are shown in parentheses.

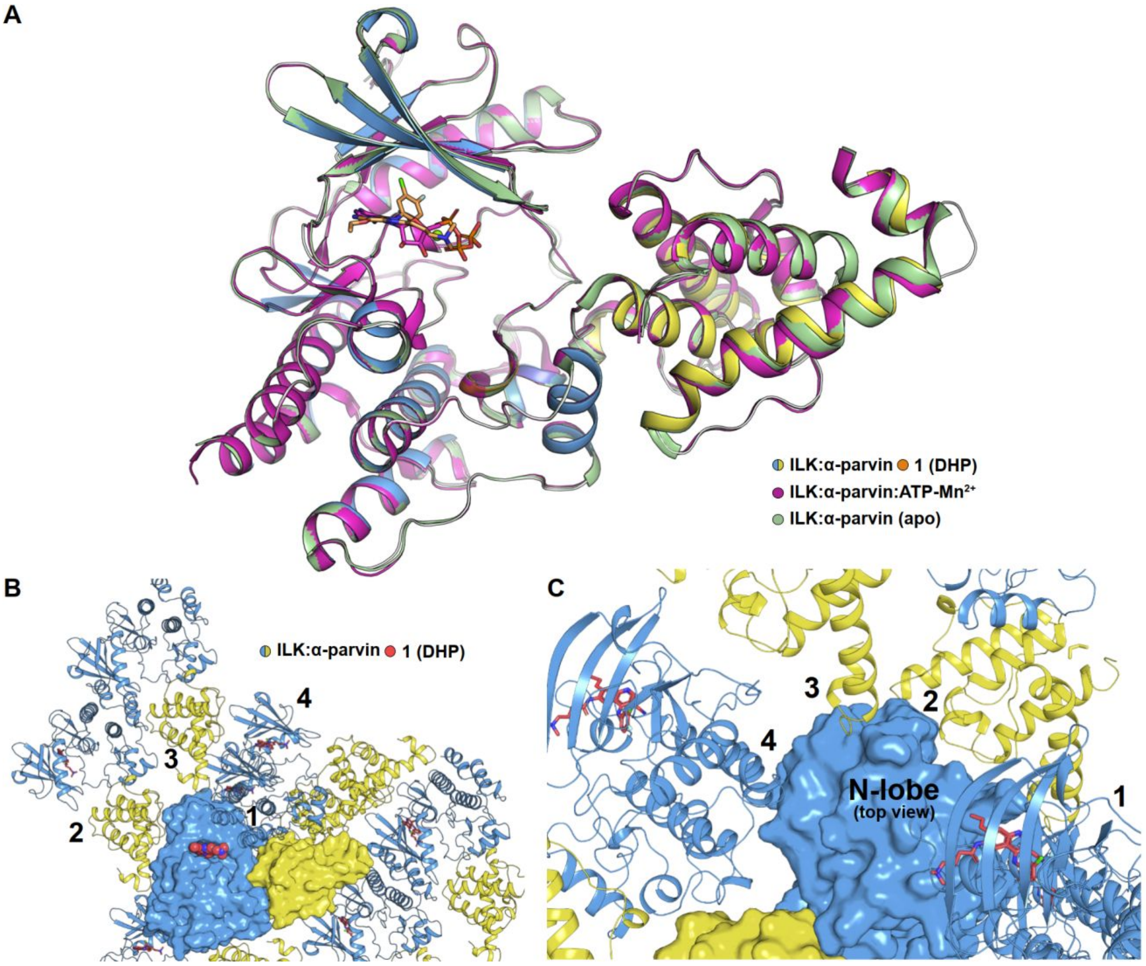
A
ILK:α-parvin
1 (DHP)
ILK:α-parvin:ATP-Mn2+
ILK:α-parvin (apo)
B
ILK:α-parvin
1 (DHP)
1
2
3
4
C
N-lobe
(top view)
1
2
3
4

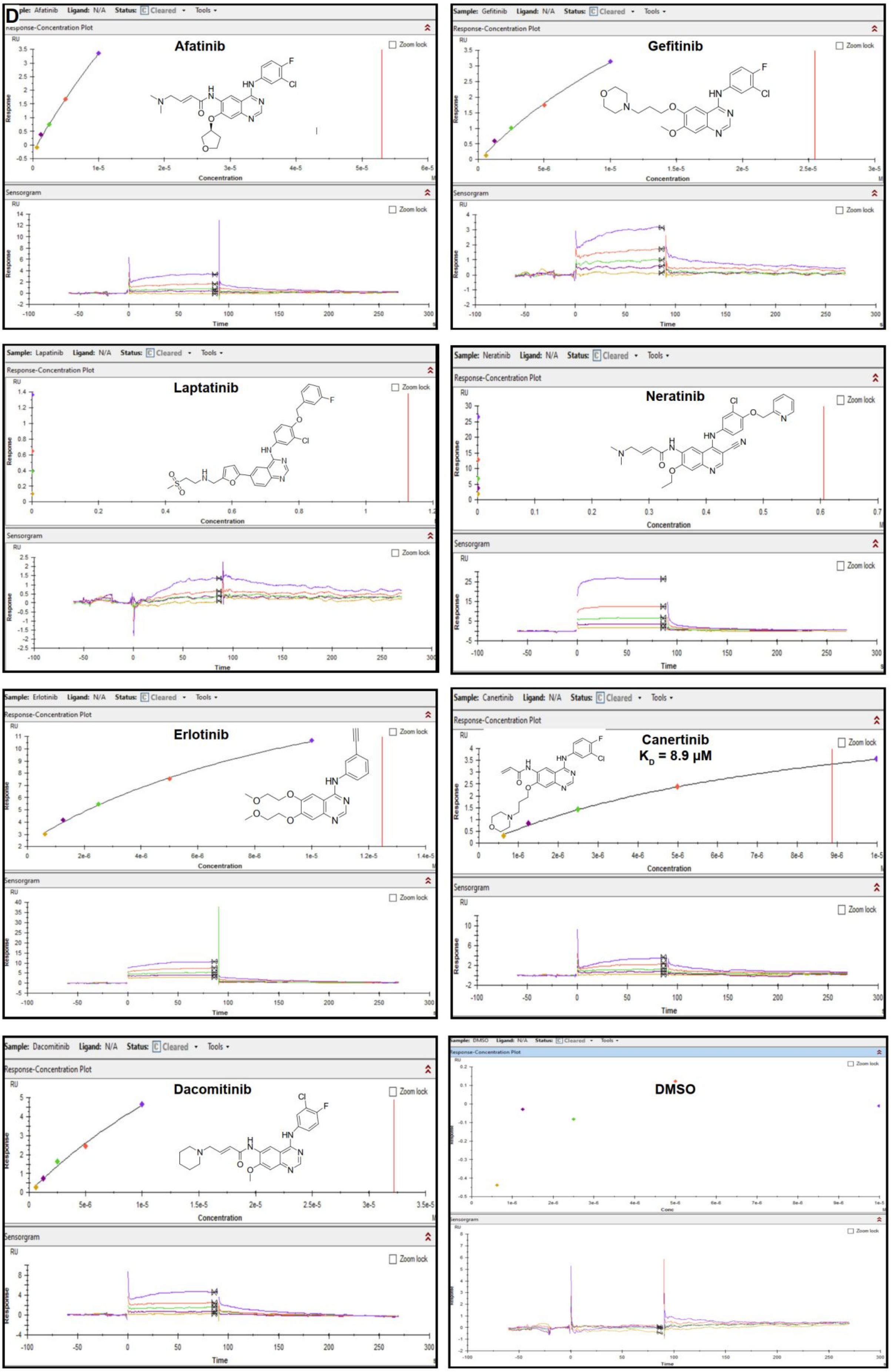
D
Sample: Afatinib Ligand: N/A Status: Cleared Tools
Response-Concentration Plot
Afatinib
Sensorgram
Sample: Gefitinib Ligand: N/A Status: Cleared Tools
Gefitinib
Sample: Lapatinib Ligand: N/A Status: Cleared Tools
Laptatinib
Sample: Neratinib Ligand: N/A Status: Cleared Tools
Neratinib
Sample: Erlotinib Ligand: N/A Status: Cleared Tools
Erlotinib
Sample: Canertinib Ligand: N/A Status: Cleared Tools
Canertinib
K_D = 8.9 μM
Sample: Dacomitinib Ligand: N/A Status: Cleared Tools
Dacomitinib
Sample: DMSO Ligand: N/A Status: Cleared Tools
DMSO
Concentration
Time
Response
Zoom lock

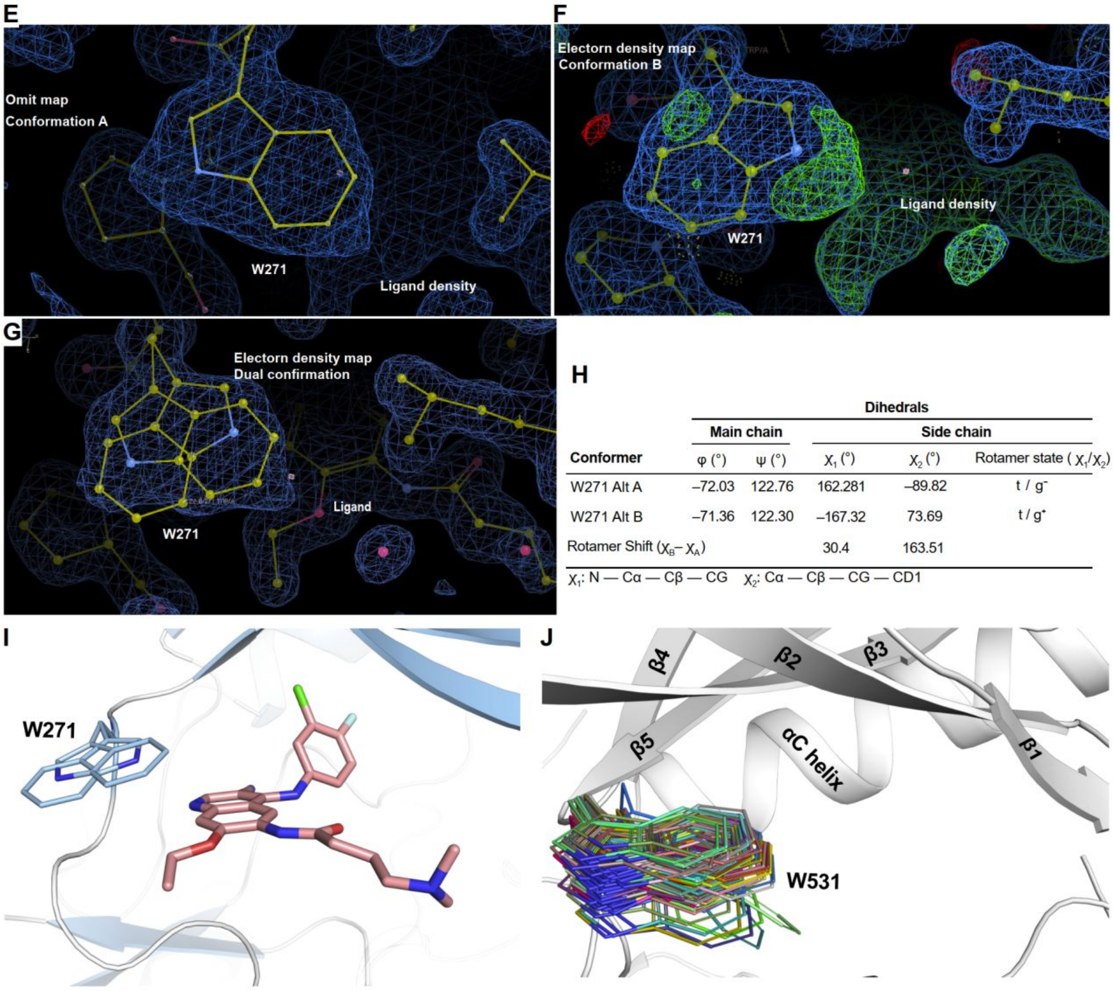

	Dihedrals				
	Main chain		Side chain		
Conformer	φ (°)	ψ (°)	χ_1 (°)	χ_2 (°)	Rotamer state (χ_1/χ_2)
W271 Alt A	–72.03	122.76	162.281	–89.82	t / g^-
W271 Alt B	–71.36	122.30	–167.32	73.69	t / g^+
Rotamer Shift ($\chi_B - \chi_A$)			30.4	163.51	

χ_1: N — Cα — Cβ — CG χ_2: Cα — Cβ — CG — CD1

Figure S2. Structural and Biophysical Evaluation and Selectivity Mechanisms.

(A–C) Structural alignment of ILK:α-parvin complexes and stabilization by symmetry-related molecules.

(A) ILK:α-parvin structures of the ATP-Mn^{2+} bound form (PDB: 3KMW) and the 1 (DHP)–bound form, aligned to the apo structure (PDB: 3KMU), with RMSD values of 0.231 and 0.304 Å, respectively.

(B) Symmetry-related molecules within 4 Å of the asymmetric unit. The symmetry mates stabilizing the kinase N-lobe are labeled 1–4.

(C) Zoomed-in top view of the kinase N-lobe showing its stabilization by two molecules of α-parvin (2 and 3) and two molecules of ILK–KD. Symmetry mates and structural alignments were generated using PyMOL.

(D) Assessment of pelitinib selectivity over related EGFR inhibitors. Steady-state affinity fits and the corresponding double-reference–subtracted sensorgrams generated using the T200 Biacore software suite, are shown alongside the Lewis structures of the tested quinoline scaffolds. Canertinib was the only compound to exhibit a plottable K_D, whereas the remaining compounds showed weak or no detectable binding at the tested concentrations. DMSO served as a control.

(E–I) Structural basis for selectivity conferred by the GK+2 W271 residue.

(E) W271 Fo–Fc omit map showing electron density for alternate conformation A, generated using Phenix.

(F) W271 Fo–Fc difference map (green) refined with alternate conformation B.

(G) 2Fo–Fc map showing both W271 conformations fitted into the electron density.

(H) Calculated dihedral angles for both W271 alternate conformations, confirming a rotamer shift and defining the residue's rotameric state. Dihedrals were calculated using PyMOL[1].

(I) W271 alternate conformations are displayed in the presence of the ligand **1** (DHP).

(J) A total of 108 BRAF structures retrieved from the KLIFS database[2] were aligned to the reference structure (PDB: 1UWH), with W531 shown as sticks. The orientation of W531 remained largely unchanged, and no side-chain rotamer flips were observed.

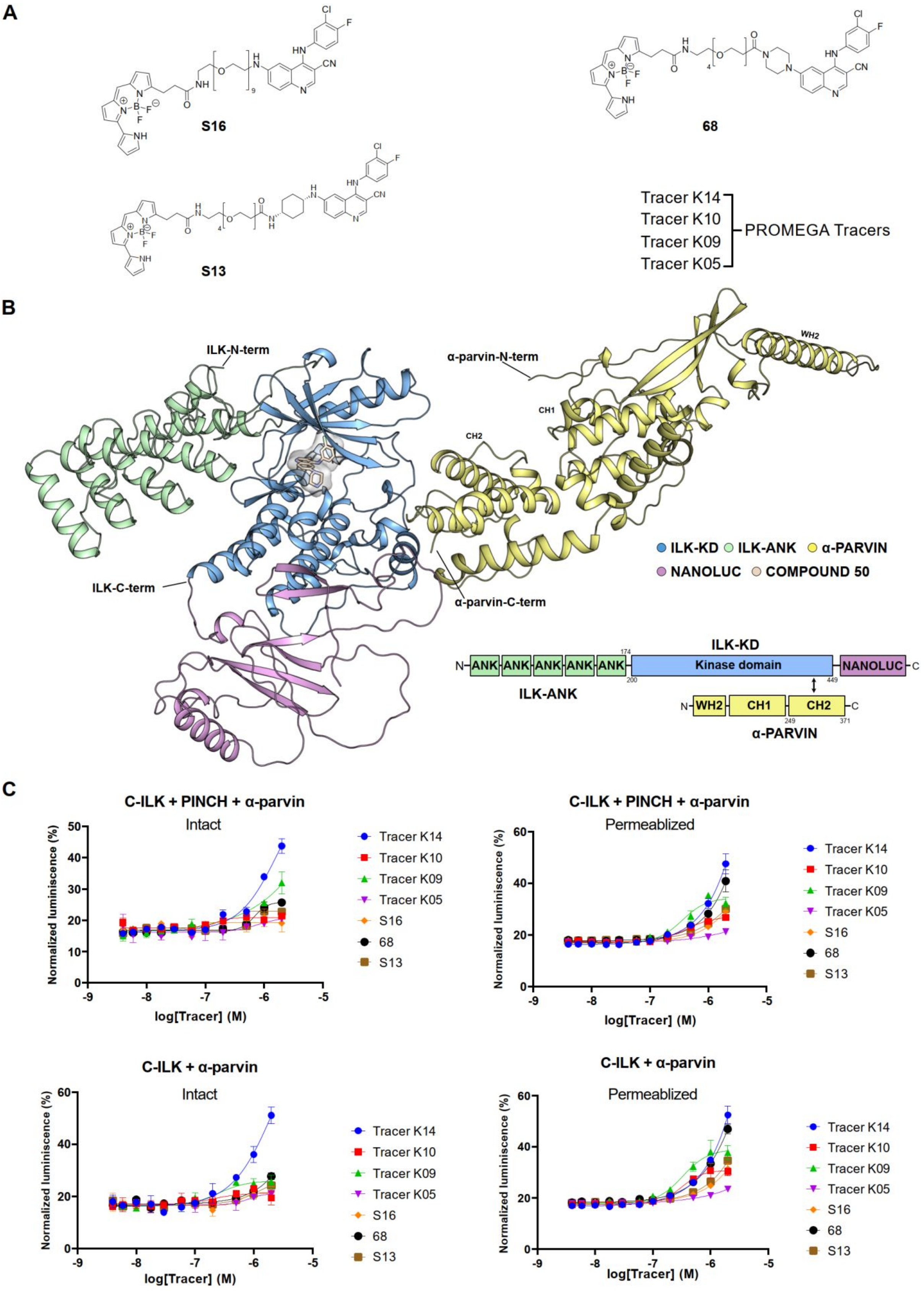

A
Cl
F
HN
CN
N
NH
O
9
B
F
S16
68
4
S13
Tracer K14
Tracer K10
Tracer K09
Tracer K05
PROMEGA Tracers
B
ILK-N-term
α-parvin-N-term
WH2
CH1
CH2
ILK-C-term
α-parvin-C-term
ILK-KD
ILK-ANK
α-PARVIN
NANOLUC
COMPOUND 50
ANK
174
Kinase domain
200
449
NANOLUC
CH1
249
371
C
C-ILK + PINCH + α-parvin
Intact
Permeablized
C-ILK + α-parvin
Normalized luminiscence (%)
log[Tracer] (M)
S16
68
S13

D

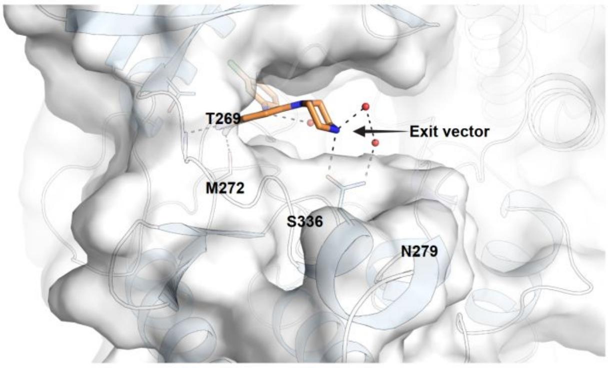

E

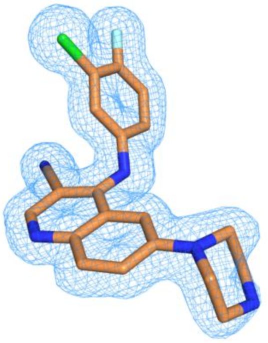

F

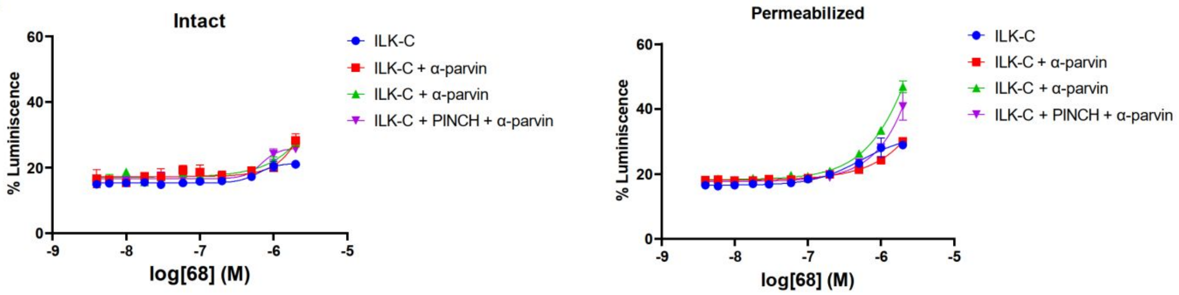

G

NanoBRET setup	NanoLuc terminal	Coexpression system	NanoBRET signal
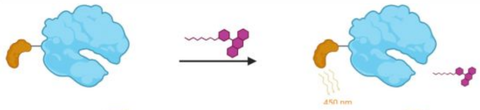	ILK - C-term	ILK alone	No
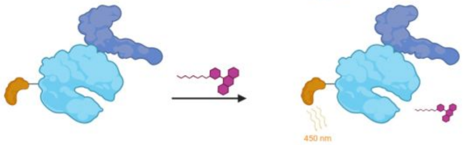	ILK - C-term	ILK + PINCH	No
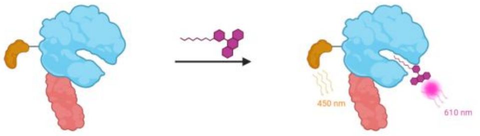	ILK - C-term	ILK + α-parvin	Yes
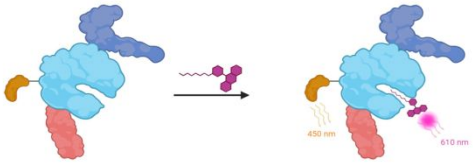	ILK - C-term	ILK + α-parvin + PINCH	Yes

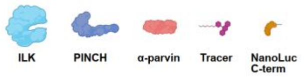

H

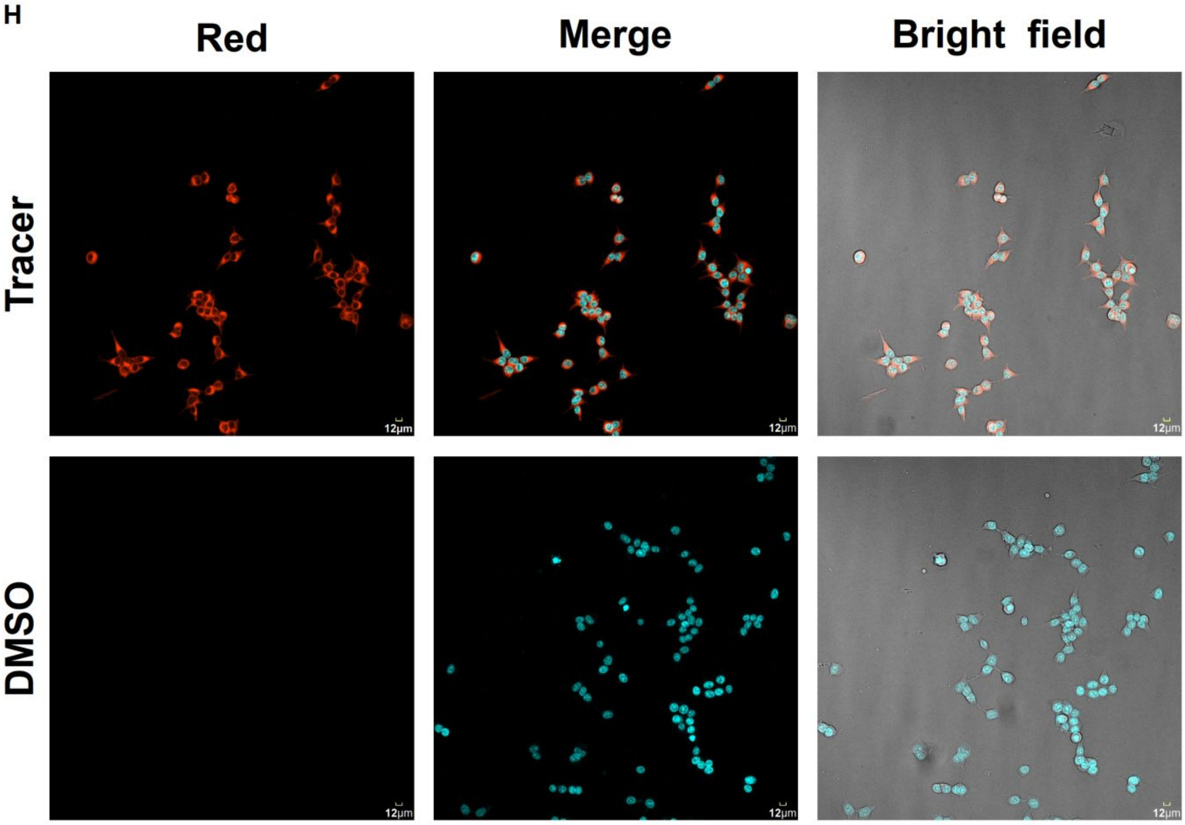

I

Cell characteristics	
cell radius	~6.5 µm
cell volume	~1.15 × 10^{-6} µL
~3000 cells (per well)	~3.45 × 10^{-3} µL
ATP concentration range	~1000 – 10,000 µM
ATP concentration average	~5000 µM
**Digitonin-permeabilized mode**	
Final assay volume / well	~15 µL
Dilution factor	~4300 (15 / 0.00345)
ATP concentration (average)	~1 µM (5000 / 4300)

J

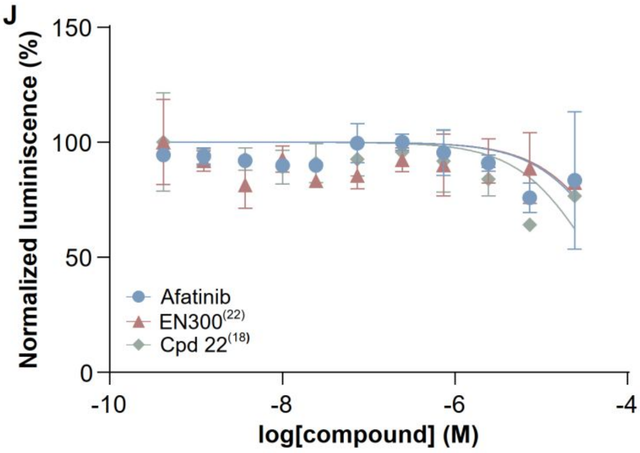

K

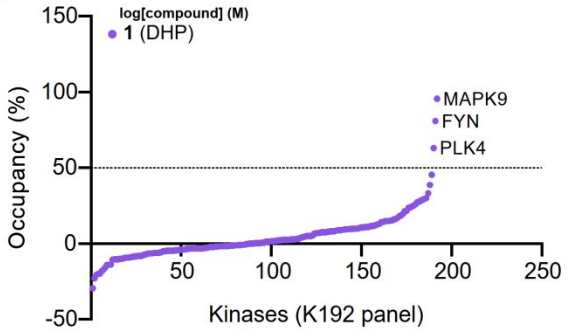

L

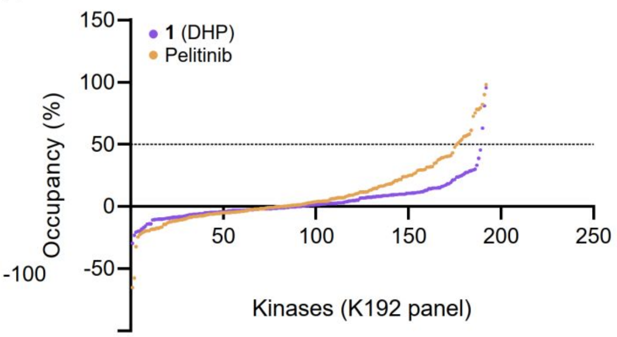

L

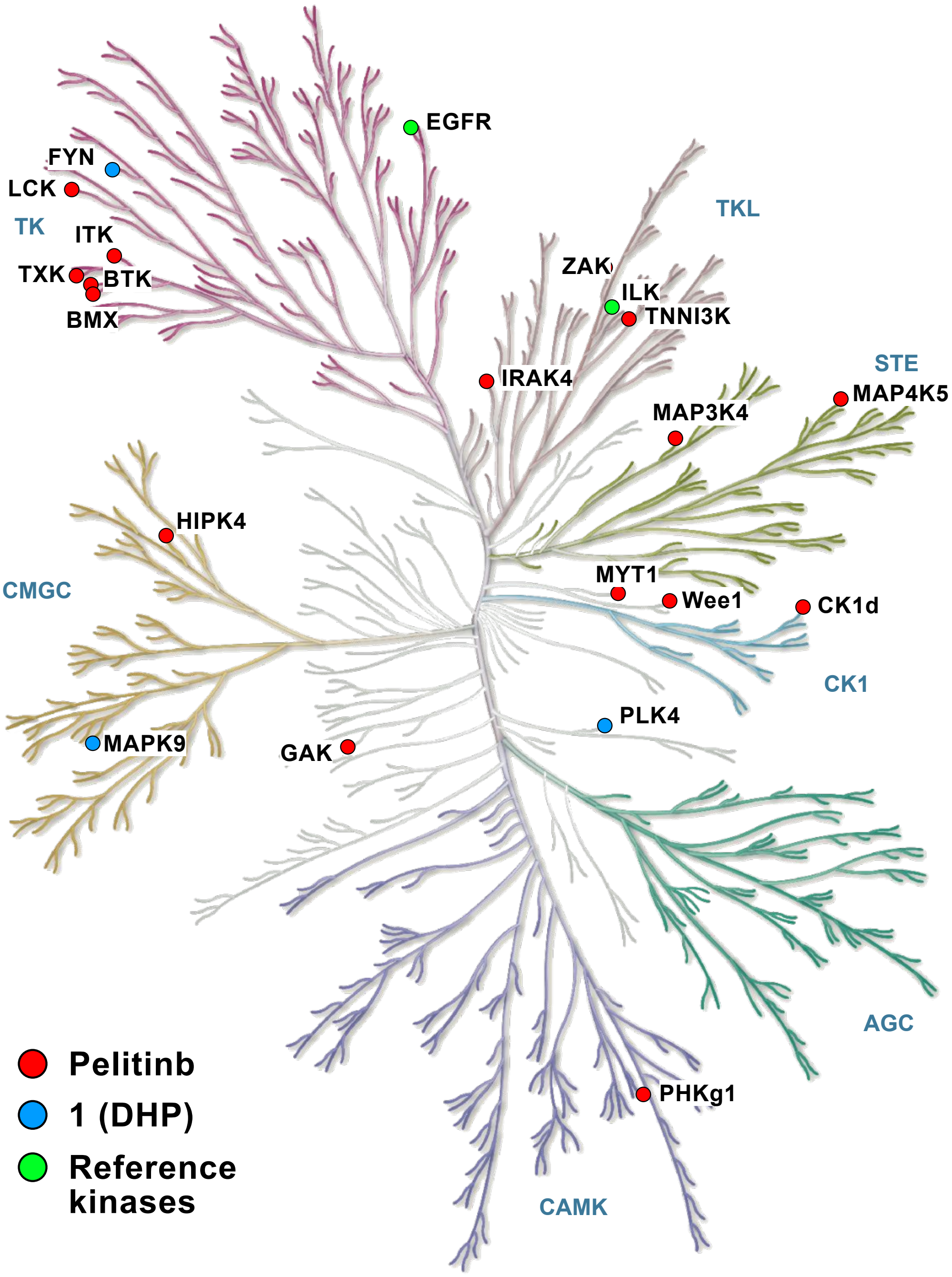

Figure S3. Development of NanoBRET tracer system and NanoBRET based selectivity data

(A) Lewis structures of the tracers tested for the NanoBRET assay. **S13**, **68**, and **S13** were synthesized in-house based on the cyano-quinoline scaffold. Four additional proprietary Promega tracers were also tested, selected using structural and phylogenetic analyses from the tracer database.

(B) Assembled full-length ILK:α-parvin complex with NanoLuc modeled onto the C-terminus of the ILK kinase domain, together with the tracer warhead used for tracer development. The model showed that NanoLuc at the ILK C-terminus did not interfere with α-parvin or PINCH binding while placing it in close proximity to the tracer. NanoLuc was modeled using AlphaFold, and the figure was generated using PyMOL. Domain architectures are indicated (bottom right).

(C) NanoBRET assay results for all tested tracers using C-terminal ILK–NanoLuc in the presence of PINCH and/or α-parvin, in both intact and digitonin-permeabilized cells.
(D) Co-crystal structure of ILK bound to the warhead **50** (orange), used for NanoBRET assay development, highlighting the ligand binding mode. The piperazine exit vector used for tracer attachment is indicated.
(E) Fo–Fc omit map (contoured at 1.0 σ) showing unbiased electron density confirming the position of **50**.
(F) Evaluation of the selected tracer **68**, assessing binding and BRET signal for C-terminal ILK–NanoLuc with different combinations of the IPP complex proteins in both intact and digitonin-permeabilized mode.
(G) Schematic of the NanoBRET configurations tested to identify a functional assay setup. Tag orientations and co-expression arrangements are shown, along with whether a BRET signal was observed.
(H) Zoomed-out CQ1 confocal image demonstrating tracer permeability, shown here to display the 12 µm scale bar.
(I) ATP dilution in digitonin-permeabilized cells. Intracellular ATP decreased to about 1 µM upon addition of substrate solution, calculated from estimated average relative cellular volumes.[3,4]
(J) NanoBRET measurements in digitonin-permeabilized cells. (Left): Normalized luminescence versus compound concentration plots showing fitted binding curves. Afatinib, EN300, and Cpd 22 did not show any measurable BRET signal, as expected.
(K) Kinome selectivity profiling of compound **1** (DHP) using the NanoBRET K192 kinase panel in live cells. Target occupancy values were determined at 1 µM in duplicates. Kinases exhibiting ≥50% occupancy are highlighted.
(L) Comparison of **1** (DHP) and pelitinib under the same conditions.
(M) Kinases exceeding the 50% target occupancy cutoff were mapped onto the human kinome phylogenetic tree using kinmap[5]. Kinases engaged by **1** (DHP) and pelitinib are highlighted in blue and red, respectively. The reference kinases ILK and EGFR are highlighted in green.

A

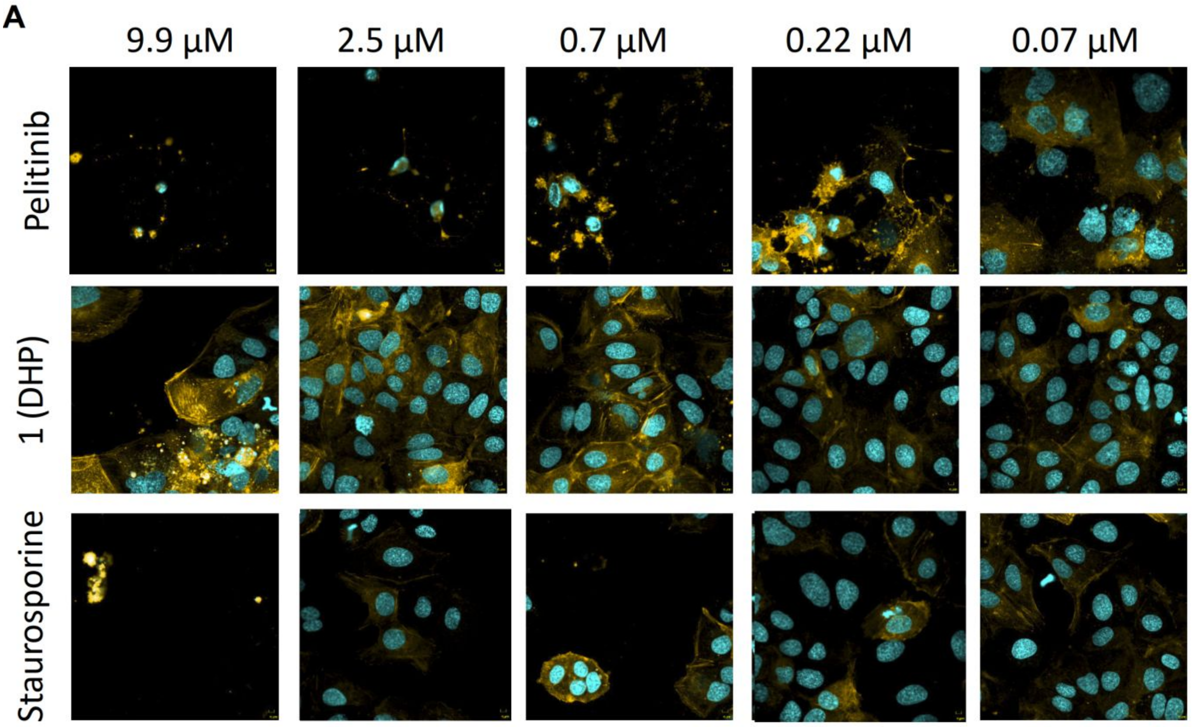

B

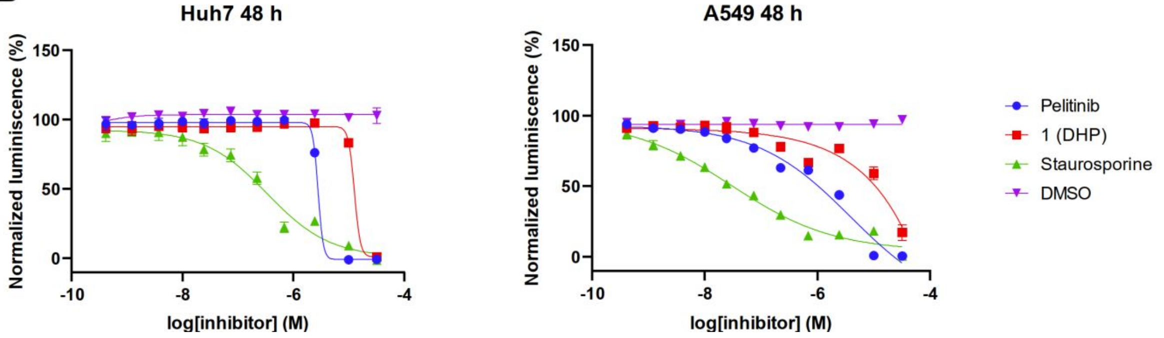

Figure S4. effect on actin cytoskeleton organization and cell titer glow viability assay

(A) Effect of **1** (DHP) on actin cytoskeleton organization. Cells were treated with DMSO or increasing concentrations of compound **1** (DHP) and pelitinib. stained with CellMask Orange Actin Tracking Stain (F-actin, appearing yellow) and Hoechst (nuclei, blue), and imaged on a CQ1 system. DHP induces concentration-dependent changes in F-actin organization. Staurosporine was used to validate the assay. Scale bar: 6 µm.

(B) CellTiter-Glo luminescent cell viability assay in Huh7 and A549 cell lines treated with 1 (DHP) and pelitinib at varying concentrations. Staurosporine and DMSO were used as positive and negative controls, respectively.

Table 1. Data Collection and Refinement Statistics.

PDB code	**9TP9**	**9TPD**
Protein complex	ILK:α-parvin	ILK:α-parvin
Ligand	1 (DHP)	Compound 50
Wavelength	0.976254	0.976269
Resolution range	40.52 - 1.45 (1.502 - 1.45)	43.07 - 1.5 (1.554 - 1.5)
Space group	P 1 21 1	P 1 21 1
Unit cell	43.8359 118.522 47.4938 90 100.384 90	43.86 118.37 47.6 90 100.91 90
Total reflections	581060 (54676)	531254 (53404)
Unique reflections	84138 (8418)	74633 (7352)
Multiplicity	6.9 (6.5)	7.1 (7.3)
Completeness (%)	99.96 (99.96)	98.20 (97.29)
Mean I/sigma(I)	23.47 (1.21)	26.87 (6.23)
Wilson B-factor	18.50	19.73
R-merge	0.04658 (0.7628)	0.03332 (0.2733)
R-meas	0.05037 (0.8303)	0.03597 (0.294)
R-pim	0.01902 (0.3232)	0.01341 (0.1079)
CC1/2	0.999 (0.792)	0.999 (0.965)
CC*	1 (0.94)	1 (0.991)
Reflections used in refinement	84113 (8416)	74630 (7352)
Reflections used for R-free	4180 (401)	7392 (732)
R-work	0.1985 (0.2711)	0.1888 (0.2342)
R-free	0.2195 (0.3037)	0.2148 (0.2699)
CC(work)	0.954 (0.840)	0.959 (0.873)
CC(free)	0.948 (0.806)	0.959 (0.826)
Number of non-hydrogen atoms	3456	3472

macromolecules	3104	3148
ligands	122	128
solvent	292	261
Protein residues	392	395
RMS(bonds)	0.007	0.010
RMS(angles)	0.97	1.13
Ramachandran favored (%)	98.97	99.23
Ramachandran allowed (%)	1.03	0.77
Ramachandran outliers (%)	0.00	0.00
Rotamer outliers (%)	0.00	0.00
Clashscore	2.24	3.46
Average B-factor	23.91	30.54
macromolecules	23.23	30.11
ligands	25.83	32.30
solvent	30.78	35.31

Statistics for the highest-resolution shell are shown in parentheses.

Scheme S1. Failed Reaction and Synthesis Limitations.

Buchwald Hartwig coupling:

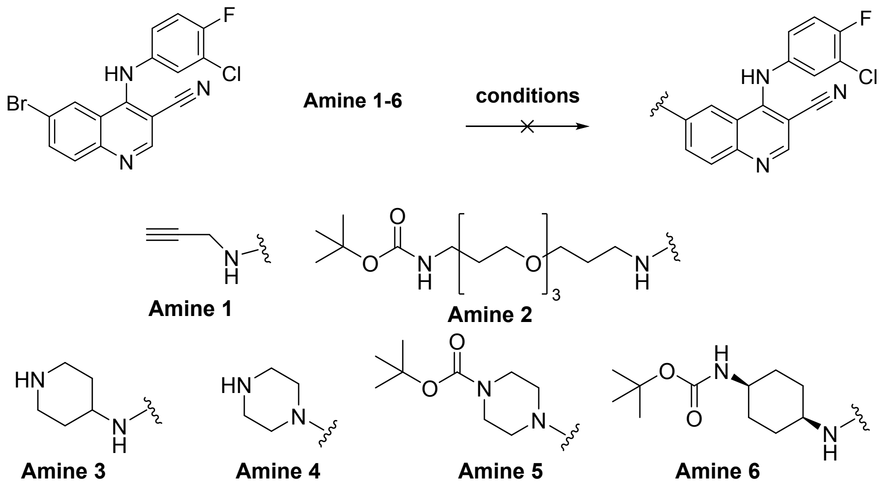

6-bromo-4-((3-chloro-4-fluorophenyl)amino)quinoline-3-carbonitrile (50 mg, 0.14 mmol, 1 eq.) the corresponding amine (2 eq.), Pd catalyst (0.1 eq.), phosphine ligand (0.1 eq.) were solved in the solvents stated below. The mixture was subjected to heating in an oil bath. No product could be isolated using this procedure.

Amine	Base	Cat./Ligand	Solvent	Conditions	Yield
1	KOtBu	Pd(dba)$_2$/BINAP	1,4-Dioxane	90 °C, 18 h	-
1	KOtBu	Pd$_2$(dba)$_3$/Xantphos	1,4-Dioxane	90 °C, 24 h	-
1	NaOtBu	Pd(OAc)$_2$/XPhos	Toluene	100 °C, 16 h	-
1	LiHMDS	XPhos Pd G2	THF	70 °C, 24 h	-
1	LiHMDS	BrettPhos Gen3	THF	70 °C, 24 h	-
2	KOtBu	Pd$_2$(dba)$_3$/BINAP	1,4-Dioxane	90 °C, 16 h	-
2	NaOtBu	Pd(dba)$_2$/BINAP	Toluene	100 °C, 36 h	-
3	Cs_2CO_3	Pd(dba)$_2$/XPhos	*t*-BuOH	100 °C, 24 h	-
3	NaOtBu	Pd(OAc)$_2$/Josiphos	DME	100 °C, 36 h	-
4	KOtBu	Pd$_2$(dba)$_3$/BINAP	1,4-Dioxane	100 °C, 18 h	-
4	NaOtBu	Pd(OAc)$_2$/Josiphos	DME	100 °C, 48 h	-
5	NaOtBu	Pd(OAc)$_2$/Josiphos	DME	100 °C, 48 h	-
8	NaOtBu	Pd(OAc)$_2$/Josiphos	DME	100 °C, 48 h	-

S$_N$Ar with Pyridin-4-amine

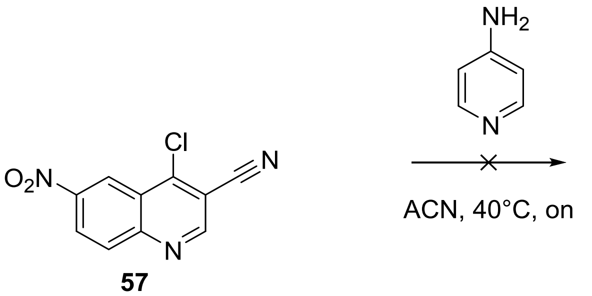

57 (30 mg, 0.13 mmol, 1 eq.) was solved in dry ACN (5 mL). Pyridin-4-amine (18 mg, 0.19 mmol, 1.5 eq.) was added to the mixture and heated overnight. No product could be isolated.

S$_N$Ar using 4-((3-chloro-4-fluorophenyl)amino)-6-fluoroquinoline-3-carbonitrile

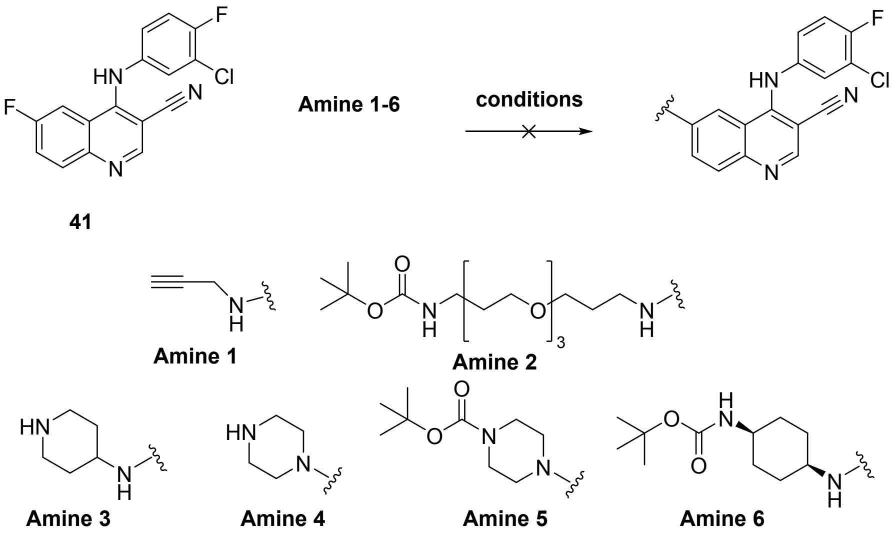

Compound **41** (125 mg, 0.35 mmol, 1.0 eq) and the corresponding amine (3.0 eq) were solved in 2 ml of ethanol the solvent stated below in a microwave vial. The corresponding base was added and the vial was sealed. The mixture was reacted using the condition stated below. No product could be isolated using this procedure.

**Amine**	**Base**	**Solvent**	**Conditions**	**Product**	**Yield**
1	DIPEA	DMF	90 °C, 24 h	44	-
1	LiHMDS	THF	60 °C, 48 h	44	-
2	DIPEA	DMSO	130 °C, 48 h	45	-

3	K_2CO_3	DMF	80 °C, 24 h	46	-
3	DIPEA	DMSO	130 °C, 48 h	46	-
4	DIPEA	DMSO	130 °C, 48 h	47	-
4	LiHMDS	THF	100 °C, 48 h	47	-
4	DIPEA	Ethanol	120 °C, 10 h, *mw*	47	30 %
4	Cs_2CO_3	DMSO	120 °C, 10 h, *mw*	47	-
5	DIPEA	Ethanol	120 °C, 10 h, *mw*	48	-
5	Cs_2CO_3	DMSO	130 °C, 12 h, *mw*	48	-
6	DIPEA	Ethanol	125 °C, 8 h, *mw*	49	-

S_NAr using organic photoredox catalysis[6]

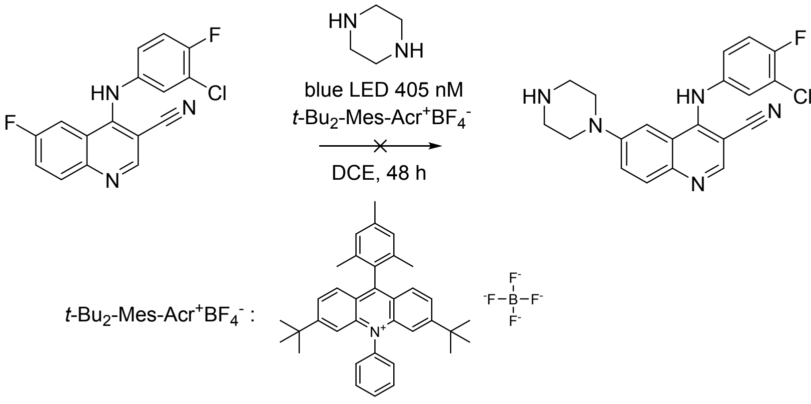

4-((3-chloro-4-fluorophenyl)amino)-6-fluoroquinoline-3-carbonitrile (30 mg, 0.1 mmol, 1 eq.), piperazine (17 mg, 0.2 mmol, 2 eq.), 9-Mesityl-3,6-di-tert-butyl-10-phenylacridinium tetrafluoroborate (12 mg, 0.02 mmol, 0.2 eq.) were loaded in a heat gun dried glass vial with a magnetic stirring bar. Dry 1,2- dichloroethane (2 mL) was added and the vial and was irradiated with a 405 nM UV lamp (Hepatochem) for 48 h. Product formation could not be observed using this procedure.

Ullmann coupling using tert-butyl (4-aminobicyclo[1.1.1]pentan-2-yl)carbamate

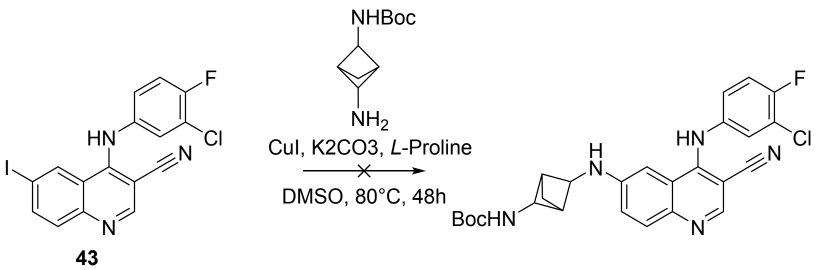

43

The synthesis was performed according to General Procedure 4. Compound **43** (100 mg, 0.21 mmol, 1.0 eq) was reacted with *tert-butyl (4-aminobicyclo[1.1.1]pentan-2-yl)carbamate* (63 mg, 0.32 mmol,

1.5 eq), *L*-proline (5 mg, 0.042 mmol, 0.2 eq), CuI (5 mg, 0.02 mmol, 0.1 eq) and potassium carbonate (90 mg, 0.65 mmol, 3.0 eq) in DMSO. The reaction was stirred at 80°C for 48h. Product formation could not be observed using this procedure.

Michael system reduction using hydroboration/protodeboronation[7]

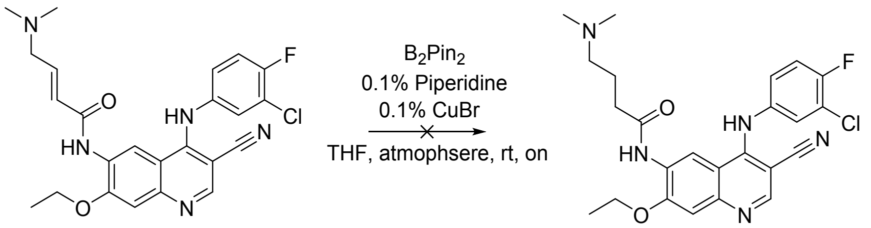

(E)-*N*-(4-((3-chloro-4-fluorophenyl)amino)-3-cyano-7-ethoxyquinolin-6-yl)-4-(dimethylamino)but-2-enamide (Pelitinib) (40 mg, 0.09 mmol, 1 eq.) was charged in a 25 mL flask. Bis(pinacolato)diboron (51 mg, 0.2 mmol, 2 eq.), CuBr (2 mg , 0.009 mmol, 0.1 eq.) and piperidine (1 µL, 0.009 mmol, 0.1 eq.) were added to the mixture and solved in 2 ml THF. The reaction was stirred over night in an open flask exposed to atmosphere. Product formation could not be observed using this procedure.

Michael system reduction with o-Nitrobenzenesulfonylhydrazide (NBSH) (Diimide reduction)[8]

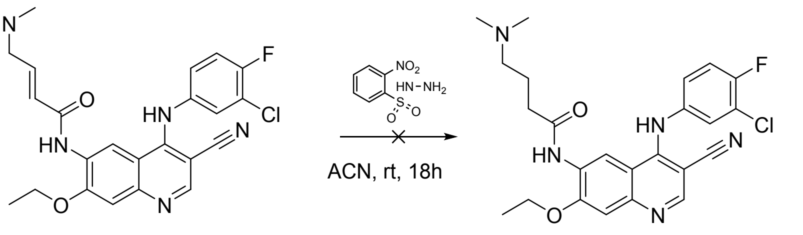

(E)-*N*-(4-((3-chloro-4-fluorophenyl)amino)-3-cyano-7-ethoxyquinolin-6-yl)-4-(dimethylamino)but-2-enamide (Pelitinib) (40 mg, 0.09 mmol, 1 eq.) was charged in a sealed glass vial. o-Nitrobenzenesulfonylhydrazide (44 mg, 0.2 mmol, 2 eq.) and KOAc (10 mg, 0.2 mmol, 1 eq.) were added and solved in dry ACN (3 mL). The mixture was stirred at room temperature for 18h. Product formation could not be observed using this procedure.

Acylation of 6-position amino moiety

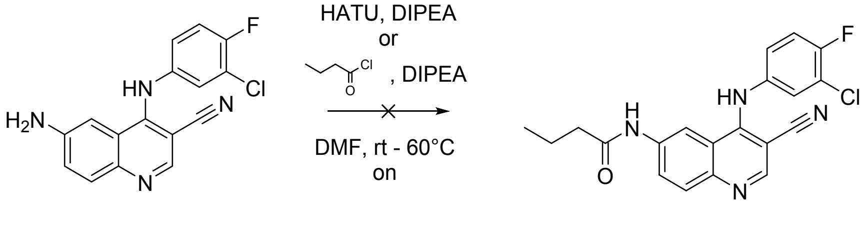

Scheme S2. Supplementary Procedures.

***tert*-butyl 4-(4-((3-chloro-4-fluorophenyl)amino)-3-cyanoquinolin-6-yl)-piperazine-1-carboxylate (S1)**

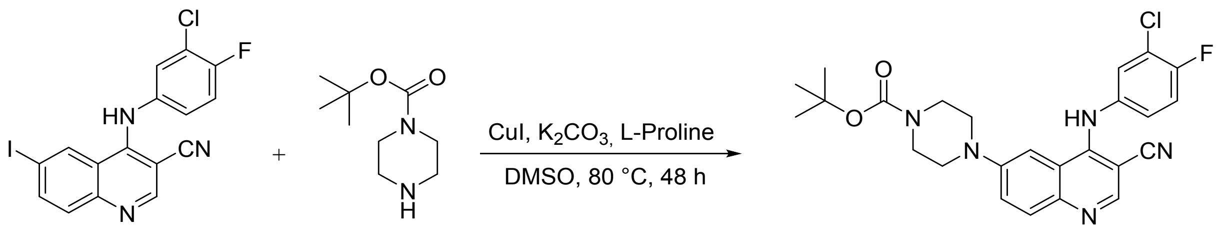

43 **S1**

The synthesis of compound **S1** was performed according to General Procedure 4. Compound **43** (200 mg, 0.435 mmol, 1.0 eq) was reacted with 1-boc-piperazine (122 mg, 0.653 mmol, 1.5 eq), *L*-proline (10 mg, 0.087 mmol, 0.2 eq), CuI (9 mg, 0.04 mmol, 0.1 eq) and potassium carbonate (180 mg, 1.301 mmol, 3.0 eq). Compound **S1** was obtained as a pale-yellow oil. Yield: 48 % (108 mg, 0.217 mmol). ^{1}H NMR (400 MHz, CDCl3): δ 8.55 (s, 1H, H-8), 7.94 (d, J = 9.3 Hz, 1H, H-18), 7.50 (dd, J = 9.3, 2.6 Hz, 1H, H-3), 7.20 (dd, J = 6.2, 2.7 Hz, 1H, H-14), 7.14 (t, J = 8.6 Hz, 1H, H-15), 7.06 – 6.99 (m, 1H, H-6), 6.93 (d, J = 2.6 Hz, 1H, H-2), 3.55 (t, 4H, H23, H-25), 3.12 (t, 4H, H-22, H-26), 1.47 (s, 10H, H-31, H-32, H-33) ppm. ^{13}C NMR (101 MHz, DMSO-d6): δ 153.92, 153.78, 149.93, 149.26, 148.43, 137.26, 129.81, 126.35, 125.14, 123.83, 119.77, 119.58, 117.24, 117.02, 105.04, 104.47, 79.15, 48.12, 45.47, 28.09 ppm. m/z = 482.10 $[M+H]^+$

Synthesis of 4-((3,5-dibromo-4-fluorophenyl)amino)-6-fluoroquinoline-3-carbonitrile (S2)

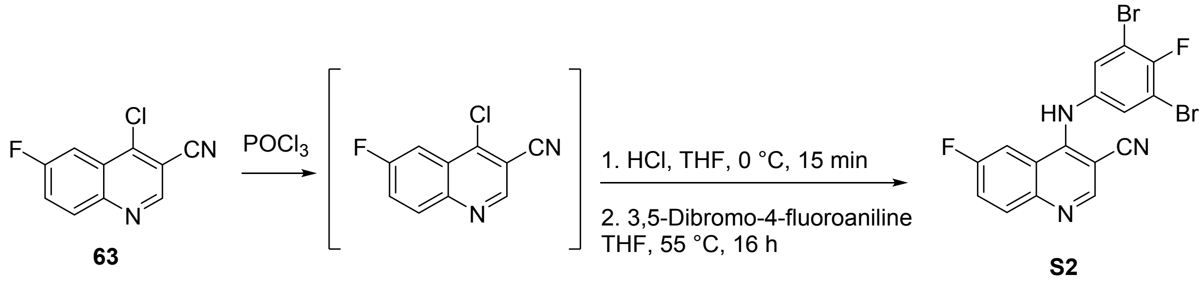

63 **S2**

The synthesis of compound **S2** was performed according to General procedure 2 (Steps 2 & 3). **63** (0.82 g, 3.99 mmol, 1.0 eq) was treated with 3 mL of $POCl_3$ and the formed crude 4-chloro intermediate was mixed with 4 N HCl in Dioxane (2.5 ml, 9.9 mmol, 2.5 eq) and 3,5-dibromo-4-fluoroaniline (578 mg, 3.99 mmol, 1.0 eq) in 10 mL THF. Compound **S2** was obtained as a light-grey solid. Yield: 73 % (0.57 mg, 1.29 mmol). ^1H NMR (400 MHz, DMSO-d6): 9.12 (s, 1H, H-11), 8.91 (d, J = 10.3 Hz, 1H, H-8), 8.24 (dd, J = 9.9, 5.2 Hz, 1H, H-3), 8.03 (t, J = 8.5 Hz, 1H, H-2), 7.94 (s, 1H, H-18), 7.93 (s, 1H, H-14), 7.09 – 7.03 (m, 1H, H-6) ppm. ^{13}C NMR (101 MHz, DMSO-d6): δ 159.23, 154.14, 149.88, 135.77, 130.51, 125.64, 124.19, 123.94, 119.26, 114.45, 111.95, 109.89, 108.89, 87.20 ppm. m/z = 439.85 $[M+H]^+$

4-((3-chloro-4-fluorophenyl)amino)-7-iodoquinoline-3-carbonitrile (S3)

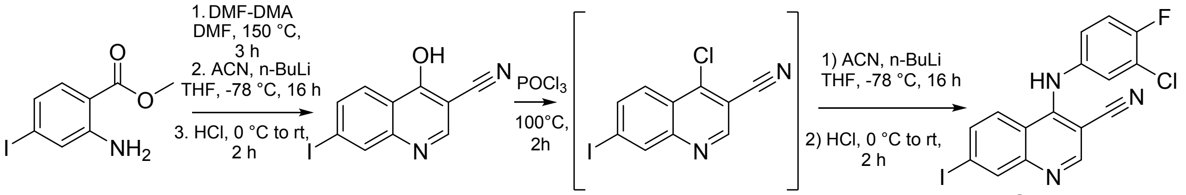

A solution of aniline derivative (2 g, 7.2 mmol, 1.0 eq) and DMF-DMA (2.89 mL, 21.66 mmol 3.0 eq) in 10 ml of DMF was stirred at 150 °C for 3 h. Afterwards the solution was diluted with 20 ml DCM and the organic phase was washed with 10 ml H_2O (3x). The remaining aqueous phase was extracted with 10 ml DCM (2x). The combined organic layers were dried with $MgSO_4$, filtered and the solvent was removed in vacuo. The crude product was obtained as a purple liquid and used without further purification. Thereupon, dry ACN (0.72 mL, 21.7 mmol, 3.0 eq) was dissolved in 10 ml of dry THF. The reaction was cooled to -78 °C and *n*-BuLi 2.5 M in hexanes (7.22 mL, 18.1 mmol, 2.5 eq) was added dropwise over an hour. The suspension was stirred for two hours at -78 °C. Afterwards, a solution of the crude imine derivative (2.4 g, 7.2 mmol, 1.0 eq) in 10 ml of dry THF was added dropwise over 30 minutes at -78 °C. The reaction mixture was stirred at room temperature overnight and quenched by addition of 4 M HCl in dioxane (7.2 mL, 28.0 mmol, 4.0 eq) and stirred for 2 h. After addition of 25 ml H_2O, the colourless precipitate was filtered and washed with ACN. The residue was dried in vacuo to give the 4-hydroxy-quinoline a as a grey solid. The precipitate formed in the previous step was solved in 8 eq. of $POCl_3$ (5 mL) and stirred at 100°C for 2h to yield the chlorinated derivative which was directly used in the next step. The crude chlorinated derivative was solved in 10 mL of dry THF and cooled to 0°C using an ice bath. 4 M HCl in dioxane (3.6 mL, 14.4 mmol, 2.5 eq) were added dropwise and the solution was stirred for 15 minutes at 0 °C. Next, 3-Chloro-4-fluoroaniline (1.1 g, 7.6 mmol, 1.05 eq)

was added to the reaction mixture. The solution was stirred for 2 hours at room temperature. The suspension was filtered, and the light-yellow precipitate was washed with ACN. The residue was dried in vacuo to give **S3** derivative as a light yellow solid. Yield: 65% after four steps (2.1 g, 5 mmol). ^{1}H NMR (400 MHz, DMSO-d6) δ 9.27 (d, J = 1.8 Hz, 1H), 8.96 (s, 1H), 8.28 (dd, J = 8.8, 1.7 Hz, 1H), 7.87 (d, J = 8.8 Hz, 1H), 7.75 (dd, J = 6.6, 2.5 Hz, 1H), 7.54 (d, J = 9.0 Hz, 1H), 7.49 – 7.46 (m, 1H), 7.33 (d, J = 5.1 Hz, 1H), 7.21 (s, 1H), 3.68-3.58 (m, 1,4-dioxane), 1.75-1.69 (m, grease) ppm. ^{13}C NMR (101 MHz, DMSO) δ 155.14, 152.73, 151.06, 142.27, 132.57, 128.19, 127.06, 125.71, 120.43, 119.89, 119.70, 117.42, 117.20, 114.80, 94.06, 87.25, 65.29 (1,4-dioxane), 45.00, 28.43 (*n*-hexanes), 27.26 (grease) ppm. m/z = 423.90 $[M+H]^+$

Synthesis of 4-Hydroxy-7-nitroquinoline-3-carbonitrile (S4)

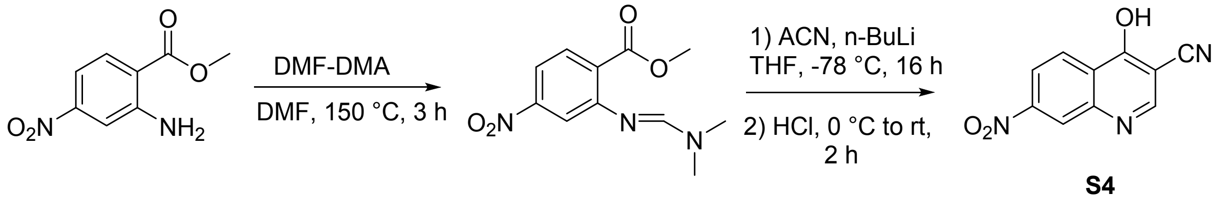

S4

The synthesis of compound **12** was performed according to General Procedure 1. By utilizing Methyl 2-amino-4-nitrobenzoate (1.00 g, 5.49 mmol, 1.0 eq) and DMF-DMA (2.2 ml, 16.5 mmol, 3.0 eq) compound **7** was obtained as a dark-purple liquid in the first step. Thereupon, compound **7** (1.37 g, 5.49 mmol, 1.0 eq) was used without further purification and reacted with ACN (0.9 ml, 16.5 mmol, 3.0 eq) and *n*-BuLi 2.5 M in hexanes (5.5 ml, 13.7 mmol, 2.5 eq) to give compound **S4**. Yield: 40 % over two steps (473 mg, 2.19 mmol). ^{1}H NMR (400 MHz, DMSO-d6): δ 8.87 (s, 1H, H-8), 8.80 (d, J = 2.6 Hz, 1H, H-6), 8.52 (dd, J = 9.1, 2.7 Hz, 1H, H-1), 7.83 (d, J = 9.1 Hz, 1H, H-3). ^{13}C NMR (101 MHz, DMSO-d6): δ 173.88, 148.42, 144.14, 143.00, 127.33, 124.62, 121.39, 121.29, 115.93, 95.23. m/z = 256.95 $[M+H]^+$

4-((3-chloro-4-fluorophenyl)amino)-7-nitroquinoline-3-carbonitrile (S5)

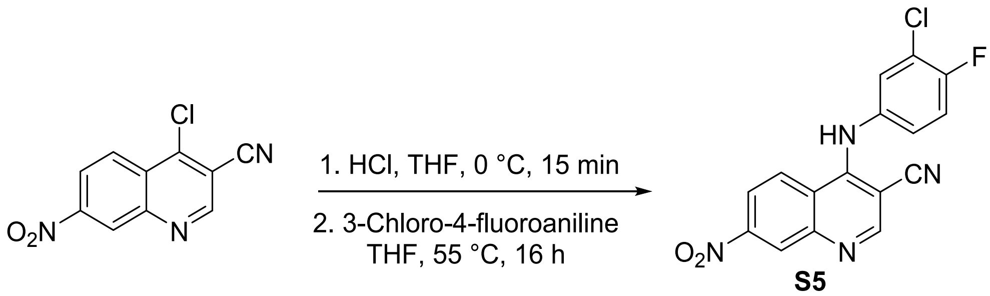

S5

The synthesis of compound S4 was performed according to General procedure step 2 and step 3. By utilizing crude 4-choloro intermediate (487 mg, 2.09 mmol, 1.0 eq), 4M HCl in Dioxane (1.3 ml, 5.2

mmol, 2.5 eq) and 3-Chloro-4-fluoroaniline (318 mg, 2.19 mmol, 1.05 eq) compound **S5** was obtained as a yellow solid. Yield: 63 % (456 mg, 1.33 mmol). ^{1}H NMR (300 MHz, DMSO-d6): δ 10.54 (s, 1H), 9.57 (s, 1H), 8.78 (s, 1H), 8.55 (d, J = 9.5 Hz, 1H), 8.12 (d, J = 9.3 Hz, 1H), 7.71 (s, 1H), 7.57 – 7.34 (m, 2H) ppm. ^{13}C NMR (101 MHz, DMSO-d6): δ 149.69, 128.54, 126.85, 126.10, 124.84, 122.76, 120.13, 120.11, 117.79, 116.68, 115.55, 113.07, 107.94, 90.38 ppm. m/z = 342.95 $[M+H]^+$

7-amino-4-((4-chloro-3-fluorophenyl)amino)quinoline-3-carbonitrile (S6)

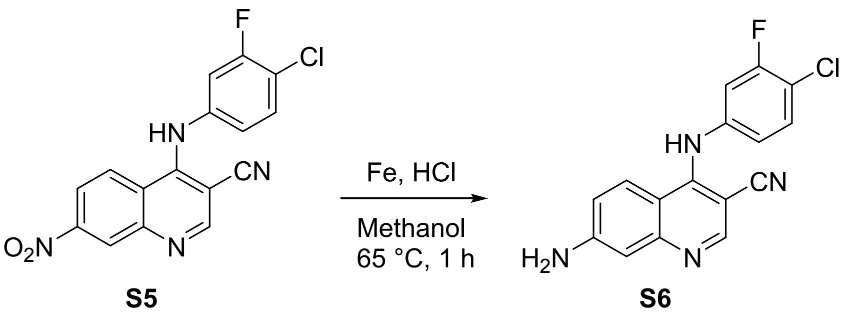

Compound **S5** (285 mg, 0.831 mmol, 1.0 eq) and iron powder (371 mg, 6.65 mmol, 8.0 eq) were solved in 5 ml of methanol. The solution was diluted with 4M HCl in Dioxane (0.5 ml, 2.1 mmol, 2.5 eq) and refluxed at 65 °C for one hour. The reaction mixture was filtered through a pad of celite and the filter cake rinsed with 10 ml of methanol (2x). The solvent was removed in vacuo to give compound **S6** as a light-brown solid. Purification of **S6** was performed using chromatography (normal phase n-Hexanes/EtOAc 100/0% → 0/100% + reverse phase H_2O/ACN: 90/10 → 0/100). Compound stability was a limiting factor. Thus, the compound was used as a crude in the next coupling steps. Conversion: 99% (257 mg, 0.827 mmol). m/z = 313.05 $[M+H]^+$

5-Chloro-N-(4-((3-chloro-4-fluorophenyl)amino)-3-cyanoquinolin-7-yl)-pentanamide (S7)

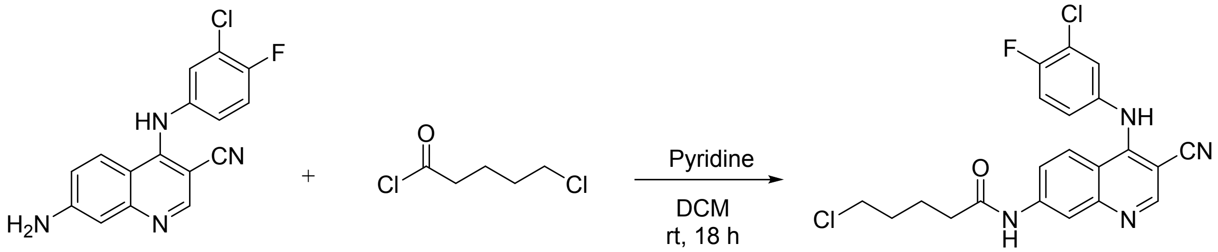

Compound **S6** (44 mg, 0.141 mmol, 1.0 eq), 5-Chloropentanoyl chloride (60 µl, 0.423 mmol, 3.0 eq) and pyridine (28 µl, 0.353 mmol, 2.5 eq) were solved in 2 ml of DCM and stirred at room temperature for 18 h. The reaction was quenched with 10 ml of H_2O and the product was extracted with 10 ml of DCM (3x). The combined organic layers were washed with 10 ml of H_2O (2x), dried over $MgSO_4$, filtered and the solvent was removed in vacuo. The crude product was purified via reverse-phase flash

chromatography (H_2O/ACN: 90/10 → 0/100) to give compound 35 as a dark-red solid. Yield: 17 % (10.3 mg, 0.024 mmol). ^{1}H NMR (400 MHz, DMSO-d6): δ 10.91 (s, 1H, H-21), 10.78 (s, 1H, H-12), 8.90 (s, 1H, H-8), 8.57 (d, J = 9.2 Hz, 1H, H-6), 8.47 (d, J = 2.1 Hz, 1H, H-3), 7.83 (dd, J = 9.3, 2.1 Hz, 1H, H-14), 7.74 (dd, J = 6.6, 2.6 Hz, 1H, H-1), 7.54 (t, J = 8.9 Hz, 1H, H-17), 7.50 – 7.40 (m, 1H, H-18), 3.69 (t, J = 6.0 Hz, 2H, H-26), 1.85 – 1.71 (m, 4H, H-24, H-25), 1.23 (m, 2H, H-23) ppm. ^{13}C NMR (101 MHz, DMSO-d_6) δ 172.31, 144.09, 128.14, 126.92, 124.85, 119.94, 117.45, 117.23, 113.98, 86.28, 45.10, 39.52, 35.62, 31.52, 22.18 ppm. m/z = 431.05 [M+H]$^+$.

***tert*-butyl(15-(4-(4-((3-chloro-4-fluorophenyl)amino)-3-cyanoquinolin-6-yl)piperazin-1-yl)-15-oxo-3,6,9,12-tetraoxapentadecyl)carbamate (S8)**

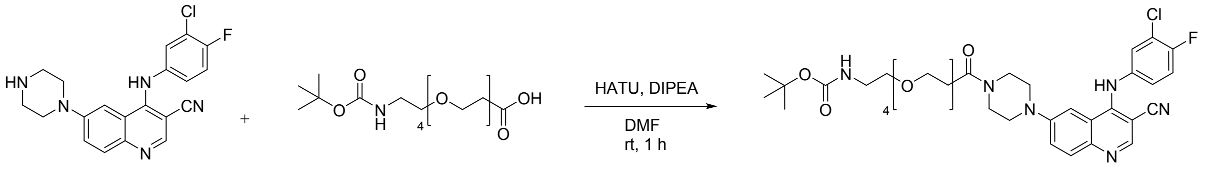

50

S8

2,2-Dimethyl-4-oxo-3,8,11,14,17-pentaoxa-5-azaicosan-20-oic acid (8.2 mg, 0.023 mmol, 1.2 eq) and HATU (9.6 mg, 0.025 mmol, 1.3 eq) were dissolved in 1 ml of DMF. DIPEA (10.2 µl, 0.058 mmol, 3.0 eq) was added and the reaction mixture was stirred at room temperature for 15 minutes. Compound **50** (7.9 mg, 0.020 mmol, 1.0 eq) was added to the solution and the reaction mixture was stirred for 1 h. The solvent was removed in vacuo and the crude product was purified via reverse-phase flash chromatography ((H2O/ACN + 0.1 % TFA): 90/10 → 0/100) to give compound **S8** as a colourless resin with a yield of 71 % (10.7 mg, 0.014 mmol). The required purity of ≥ 95% could not be reached thus the compound was used as it is in the next step. m/z = 729.70 [M+H]$^+$

6-(4-(1-amino-3,6,9,12-tetraoxapentadecan-15-oyl)piperazin-1-yl)-4-((3-chloro-4-fluorophenyl)amino)quinoline-3-carbonitrile (S9)

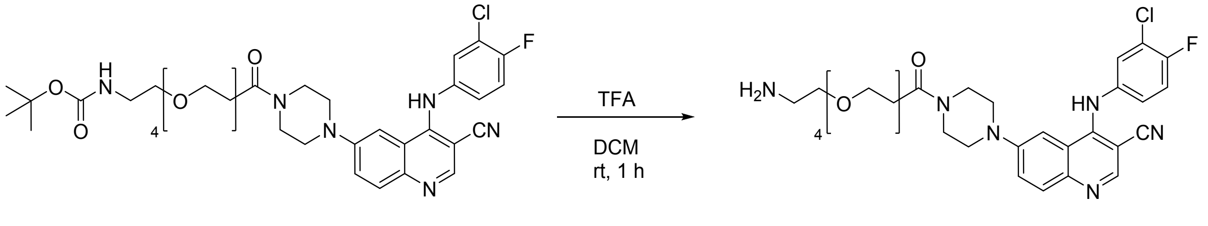

S8

S9

Compound **S8** (10 mg, 0.014 mmol) was solved in 1 ml of DCM. Afterwards, 0.5 ml of TFA were added and the solution was stirred at room temperature for 1 h. After characterization via LC-MS, the

reaction mixture was diluted with 2 ml of toluene and the solvent was removed in vacuo. The crude product **S9** was utilized without further purification. m/z = 629.25 [M+H]+

6-(((1*s*,4*s*)-4-aminocyclohexyl)amino)-4-((3-chloro-4-fluorophenyl)amino)-quinoline-3-carbonitrile (S10)

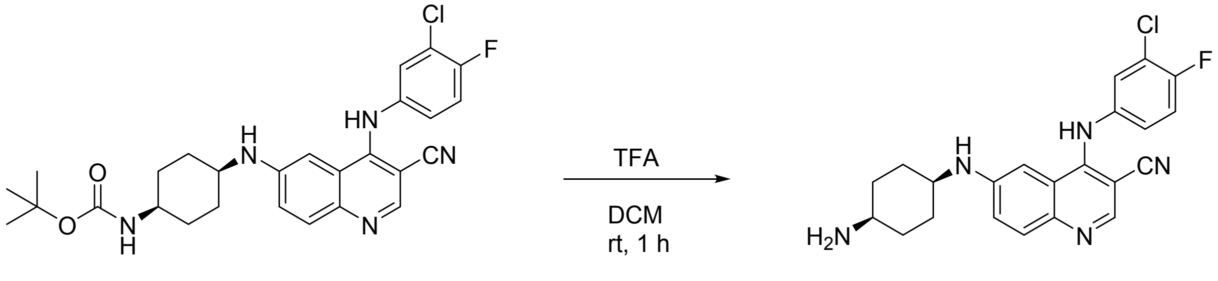

Compound **52** (10 mg, 0.019 mmol) was solved in 1 ml of DCM. Afterwards, 0.5 ml of TFA were added and the solution was stirred at room temperature for one hour. After characterization via LC-MS, the reaction mixture was diluted with 2 ml of toluene and the solvent was removed in vacuo. The crude product **S10** was utilized without further purification. m/z = 410.10 [M+H]+

***tert*-butyl (15-(((1s,4s)-4-((4-((3-chloro-4-fluorophenyl)amino)-3-cyano-quinolin-6-yl)amino)cyclohexyl)amino)-15-oxo-3,6,9,12-tetraoxapentadecyl)carbamate (S11)**

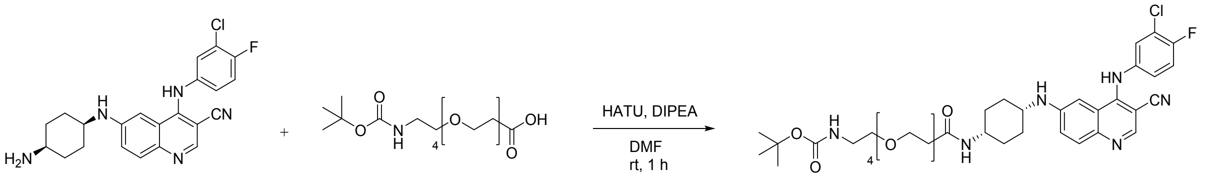

2,2-Dimethyl-4-oxo-3,8,11,14,17-pentaoxa-5-azaicosan-20-oic acid (8.2 mg, 0.023 mmol, 1.2 eq) and HATU (9.6 mg, 0.025 mmol, 1.3 eq) were dissolved in 1 ml of DMF. DIPEA (10.2 µl, 0.058 mmol, 3.0 eq) was added and the reaction mixture was stirred at room temperature for 15 minutes. Compound **S10** (8 mg, 0.02 mmol, 1.0 eq) was added to the solution and the reaction mixture was stirred for 1 h. The solvent was removed in vacuo and the crude product was purified via reverse-phase flash chromatography (H_2O/CAN 90/10% → 0/100%) to give compound **S11** as a colourless resin with a yield of 68 % (10 mg, 0.013 mmol). The required purity of ≥ 95% could not be reached thus the compound was used as it is in the next step. m/z = 757.80 [M+H]+

1-amino-*N*-((1s,4s)-4-((4-((3-chloro-4-fluorophenyl)amino)-3-cyanoquinolin-6-yl)amino)cyclohexyl)-3,6,9,12-tetraoxapentadecan-15-amide (S12)

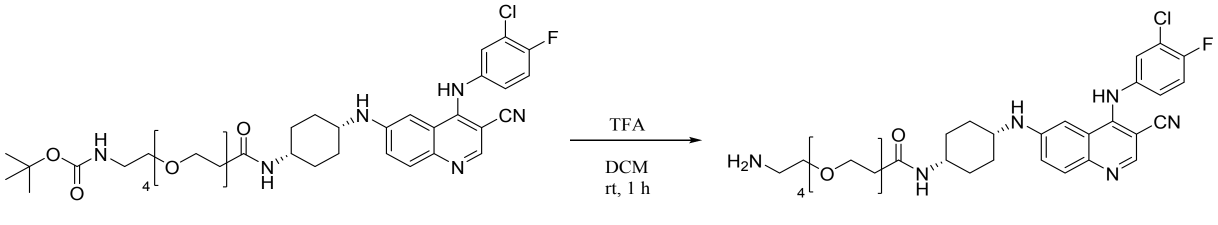

S11 **S12**

Compound **S11** (10 mg, 0.013 mmol) was solved in 1 ml of DCM. Afterwards, 0.5 ml of TFA were added and the solution was stirred at room temperature for 1 h. After characterization via TLC, the reaction mixture was diluted with 2 ml of toluene and the solvent was removed in vacuo. The crude product **S12** was utilized without further purification. Rf : 0.18 (DCM/MeOH; 9:1).

3-(19-(((1s,4s)-4-((4-((3-chloro-4-fluorophenyl)amino)-3-cyanoquinolin-6-yl)-amino)cyclohexyl)amino)-3,19-dioxo-7,10,13,16-tetraoxa-4-azanonadecyl)-5,5-difluoro-7-(1H-pyrrol-2-yl)-5H-6λ4-dipyrrolo[1,2-c:2',1'-f][1,3,2]diazaborinin-5-uide (S13)

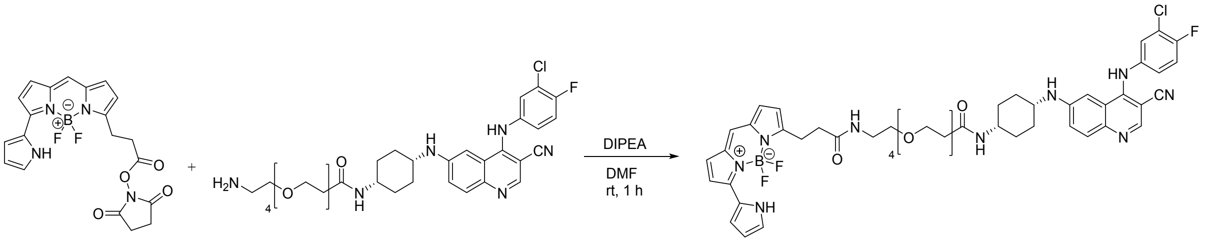

S12 **S13**

2,5-Dioxopyrrolidin-1-yl-3-(5,5-difluoro-7-(1H-pyrrol-2-yl)-5H-5λ4,6λ4-dipyrrolo[1,2-c:2',1'-f][1,3,2]diazaborinin-3-yl)propanoate (5.1 mg, 0.012 mmol, 1.0 eq) and compound **S12** (8.6 mg, 0.013 mmol, 1.1 eq) were dissolved in 1 ml of DMF. DIPEA (6.2 µl, 0.036 mmol, 3.0 eq) was added and the reaction mixture was stirred for one hour. The solvent was removed in vacuo and the crude product was purified via reverse-phase flash chromatography ((H2O/ACN + 0.2 % TFA): 98/2% → 0/100%) to give compound **S13** as a dark-purple solid. Yield: 30 % (3.4 mg, 0.004 mmol). ^{1}H NMR (500 MHz, DMSO-d6): δ 11.43 (s, 1H), 10.43 (s, 1H), 8.68 (s, 1H), 8.02 (t, J = 5.6 Hz, 1H), 7.95 (s, 1H), 7.78 – 7.68 (m, 3H), 7.56 – 7.49 (m, 1H), 7.47 – 7.41 (m, 2H), 7.36 (s, 1H), 7.33 (d, J = 4.5 Hz, 1H), 7.26 (s, 1H), 7.21 – 7.13 (m, 2H), 7.00 (d, J = 4.0 Hz, 1H), 6.40 – 6.29 (m, 2H), 3.62 – 3.56 (m, 2H), 3.52 – 3.46 (m, 13H), 3.41 (t, J = 5.9 Hz, 2H), 3.22 (q, J = 5.7 Hz, 1H), 3.13 (t, 3H), 2.89 (s, 2H), 2.73 (s, 2H), 2.33 (t, J = 6.5 Hz, 2H), 1.75 – 1.55 (m, 8H) ppm. ^{13}C NMR (126 MHz, DMSO-d6): δ 171.11, 170.24, 169.48, 162.37, 155.90, 150.24, 136.95, 133.02, 132.43, 126.76, 126.12, 124.40, 122.90, 119.36, 117.40, 116.14, 111.53, 96.81, 69.80, 69.58, 69.10, 66.96, 53.54, 47.80, 45.58, 38.63, 36.11, 35.79, 33.80, 30.78, 27.64, 27.23, 25.47,

24.03 ppm. ^{19}F NMR (471 MHz, DMSO-d6): δ -73.85, -117.97, -142.21. m/z = 968.45 $[M+H]^+$, HRMS: m/z = 990.38480 $[M+Na]^+$

***tert*-butyl (29-((4-((3-chloro-4-fluorophenyl)amino)-3-cyanoquinolin-6-yl)-amino)-3,6,9,12,15,18,21,24,27-nonaoxanonacosyl)carbamate (S14)**

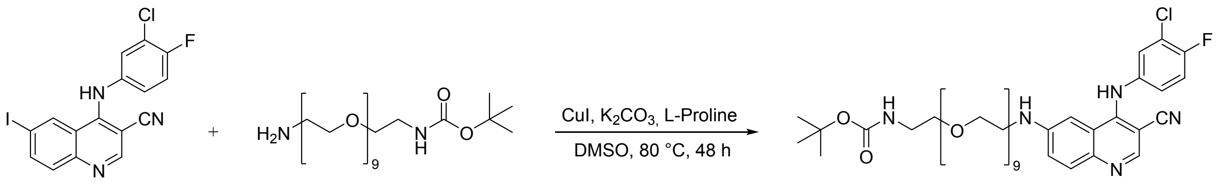

43 **S14**

The synthesis of compound **S14** was performed according to General Procedure 4. Compound **43** (75 mg, 0.16 mmol, 1.0 eq) was reacted with tert-butyl (29-amino-3,6,9,12,15,18,21,24,27-nonaoxanon-acosyl)carbamate (115 mg, 0.245 mmol, 1.5 eq), *L*-proline (4 mg, 0.03 mmol, 0.2 eq), CuI (3 mg, 0.02 mmol, 0.1 eq) and potassium carbonate (67 mg, 0.49 mmol, 3.0 eq) compound **S14** was obtained as a pale-yellow oil and used as it is in the next step. Yield: 35 % (49 mg, 0.57 mmol). ^{1}H NMR (400 MHz, DMSO-d6): δ 10.51 (s, 1H, H-11), 8.74 (d, J = 9.4 Hz, 1H, H-8), 7.81 – 7.69 (m, 3H, H-2, H-3, H-6), 7.57 (td, J = 9.0, 3.1 Hz, 1H, H, H-18), 7.51 – 7.44 (m, 2H, H-14, H-17), 7.31 (s, 1H, H-21), 6.73 (t, J = 6.2 Hz, 1H, H-51), 3.65 (t, J = 5.7 Hz, 2H), 3.61 – 3.54 (m, 7H), 3.53 – 3.48 (m, 36H), 3.43 – 3.34 (m, 4H), 3.11 (q, J = 5.9 Hz, 0H), 3.06 (q, J = 6.0 Hz, 1H), 2.98 (q, J = 5.5 Hz, 2H), 1.37 (s, J = 2.2 Hz, 9H, H-56, H-57, H-58) ppm. ^{13}C NMR (101 MHz, DMSO-d6): δ 155.58, 148.87, 148.79, 144.53, 144.39, 135.46, 128.43, 128.25, 126.98, 125.20, 125.09, 120.52, 120.02, 119.83, 117.49, 117.28, 117.26, 96.81, 77.58, 69.76, 69.16, 68.42, 66.67, 42.63, 38.62, 28.22 ppm. m/z = 853.05 $[M+H]^+$

Synthesis of 6-((29-amino-3,6,9,12,15,18,21,24,27-nonaoxanonacosyl)amino)-4-((3-chloro-4-fluorophenyl)amino)quinoline-3-carbonitrile (S15)

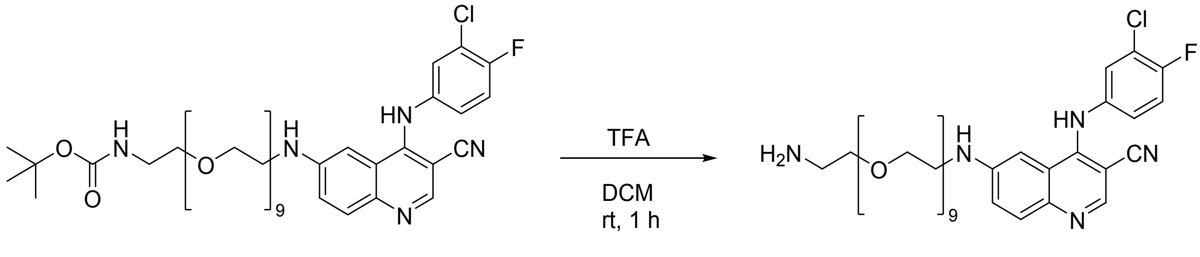

S14 **S15**

Compound **S14** (18 mg, 0.021 mmol) was solved in 1 ml of DCM. Afterwards, 0.5 ml of TFA were added and the solution was stirred at room temperature for one hour. The reaction mixture was diluted with 2 ml of toluene and the solvent was removed in vacuo. The crude product **S15** was utilized without further purification. m/z = 753.13 $[M+H]^+$

***N*-(29-((4-((3-chloro-4-fluorophenyl)amino)-3-cyanoquinolin-6-yl)amino)-3,6,9,12,15,18,21,24,27-nonaoxanonacosyl)-3-(5,5-difluoro-7-(1H-pyrrol-2-yl)-5H-5λ4,6λ4-dipyrrolo[1,2-c:2',1'-f][1,3,2]diazaborinin-3-yl)propenamide (S16)**

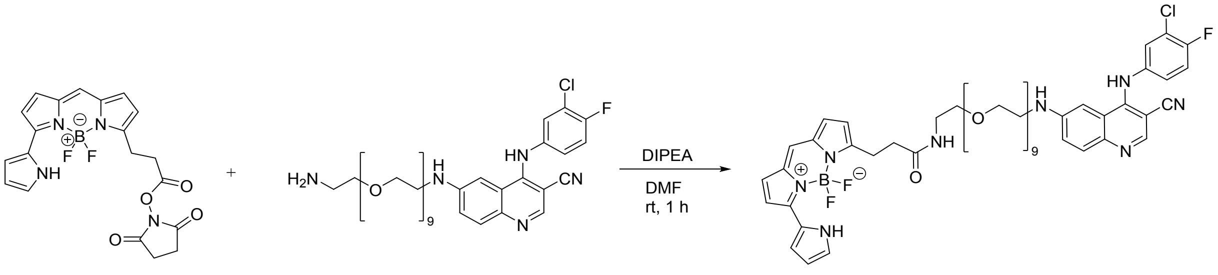

S15 S16

2,5-Dioxopyrrolidin-1-yl-3-(5,5-difluoro-7-(1H-pyrrol-2-yl)-5H-5λ4,6λ4-dipyrrolo[1,2-c:2',1'-f][1,3,2]diazaborinin-3-yl)propanoate (3.8 mg, 0.009 mmol, 1.0 eq) and compound **S15** (7.5 mg, 0.010 mmol, 1.1 eq) were dissolved in 300 µl of DMF. DIPEA (6.3 µl, 0.036 mmol, 4.0 eq) was added and the reaction mixture was stirred for one hour. After characterization via LC-MS, the reaction mixture was purified via preparative HPLC ((H_2O/ACN) + 0.1% TFA). Compound **S16** was obtained as a dark-purple solid with a yield of 94 % (11 mg, 0.008 mmol). ^{1}H NMR (400 MHz, DMSO-d6): δ 11.42 (s, 1H), 10.70 (s, 1H), 8.81 (s, 1H), 8.02 (s, 1H), 7.81 (d, J = 6.8 Hz, 1H), 7.74 (d, J = 9.1 Hz, 1H), 7.58 (t, J = 8.8 Hz, 1H), 7.54 – 7.46 (m, 2H), 7.43 (s, 1H), 7.39 – 7.31 (m, 3H), 7.27 (s, 1H), 7.16 (d, J = 4.6 Hz, 1H), 7.01 (d, J = 4.0 Hz, 1H), 6.33 (s, 2H), 3.65 (t, J = 5.7 Hz, 2H), 3.59 – 3.36 (m, 39H), 3.23 (q, J = 6.0 Hz, 2H), 3.13 (t, J = 7.7 Hz, 3H), 2.83 (s, 1H) ppm. ^{13}C NMR (101 MHz, DMSO-d6): δ 171.05, 159.29, 155.94, 150.22, 149.06, 143.94, 136.92, 132.99, 132.40, 128.91, 126.75, 126.11, 125.47, 124.39, 122.89, 119.32, 117.33, 116.13, 111.52, 96.84, 69.75, 69.58, 69.11, 68.37, 42.62, 38.61, 33.78, 25.46, 24.01 ppm. m/z = 522.35 [M+H, *z* = 2, Fluorine fragmentation]$^+$ HRMS: m/z = 1085.42975 $[M+Na]^+$

Figure S5. Anilines used in the study.

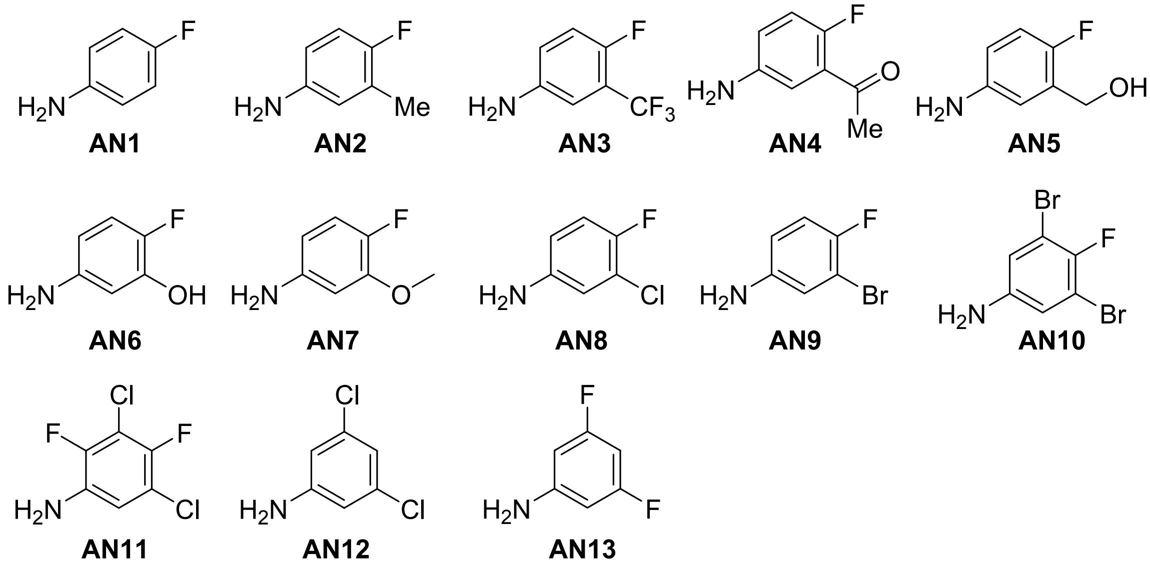

REFERENCES

(1) Schrödinger, LLC. The PyMOL Molecular Graphics System, 2024. https://pymol.org.

(2) *KLIFS: a structural kinase-ligand interaction database | Nucleic Acids Research | Oxford Academic*. https://academic.oup.com/nar/article/44/D1/D365/2502606?login=true (accessed 2025-10-14).

(3) Seidel, S.; Maschke, R. W.; Mozaffari, F.; Eibl-Schindler, R.; Eibl, D. Improvement of HEK293 Cell Growth by Adapting Hydrodynamic Stress and Predicting Cell Aggregate Size Distribution. *Bioengineering (Basel)* **2023**, *10* (4), 478. https://doi.org/10.3390/bioengineering10040478.

(4) *Diameter of HEK-293 cell - Human Homo sapiens - BNID 108893*. https://bionumbers.hms.harvard.edu/bionumber.aspx?id=108893&utm (accessed 2025-11-21).

(5) Eid, S.; Turk, S.; Volkamer, A.; Rippmann, F.; Fulle, S. KinMap: A Web-Based Tool for Interactive Navigation through Human Kinome Data. *BMC Bioinformatics* **2017**, *18* (1), 16. https://doi.org/10.1186/s12859-016-1433-7.

(6) Shi, W.; Zhang, J.; Zhao, F.; Wei, W.; Liang, F.; Zhang, Y.; Zhou, S. Nucleophilic Aromatic Substitution of Unactivated Aryl Fluorides with Primary Aliphatic Amines by Organic Photoredox Catalysis. *Chemistry – A European Journal* **2020**, *26* (65), 14823–14827. https://doi.org/10.1002/chem.202002315.

(7) Ding, W.; Song, Q. Chemoselective Catalytic Reduction of Conjugated α,β-Unsaturated Ketones to Saturated Ketones via a Hydroboration/Protodeboronation Strategy. *Org. Chem. Front.* **2015**, *3* (1), 14–18. https://doi.org/10.1039/C5QO00289C.

(8) Marsh, B. J.; Carbery, D. R. One-Pot o-Nitrobenzenesulfonylhydrazide (NBSH) Formation−Diimide Alkene Reduction Protocol. *J. Org. Chem.* **2009**, *74* (8), 3186–3188. https://doi.org/10.1021/jo900237y.
