# Supplementary material for: Identification and Validation of 3‑Cyano-Quinoline Ligands Targeting Integrin-Linked Kinase (ILK)
Source: J Med Chem. 2026 May 25;69(11):12982–3001. doi: 10.1021/acs.jmedchem.5c03773 (PMC13266989; doi:10.1021/acs.jmedchem.5c03773)
Supplement: Supplementary file 5 [file jm5c03773_si_005.pdf]

Parellel Synthesis Conversion Spectra

Compound 2

```
Data File W:\analyti...S_DATA\Natalia_HTS\FGMHTS2_13+14_1.5micro (1) 2021-03-10 06-53-31.D
Sample Name: FGMHTS2_13+14_1.5micro

    =====================================================================
    Acq. Operator   : SYSTEM
    Sample Operator : SYSTEM
    Acq. Instrument : LCMS test                    Location :   D1F-C1
    Injection Date  : 3/10/2021 7:51:07 AM              Inj :   1
                                                 Inj Volume : 1.000 µl
    Different Inj Volume from Sample Entry!   Actual Inj Volume : 1.500 µl
    Acq. Method     : C:\Users\Public\Documents\ChemStation\1\Methods\0-Std_mth\Gradient\FAST_
                      Nonpolar_General_Method_MS-PosScan-100-1000.M
    Last changed    : 2/18/2021 11:58:42 AM by SYSTEM
    Analysis Method : C:\Users\Public\Documents\ChemStation\1\Methods\DEF_LC.M
    Last changed    : 9/20/2021 11:46:17 AM by SYSTEM
                      (modified after loading)
    Additional Info : Peak(s) manually integrated
```

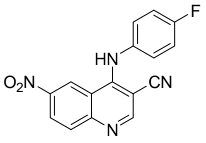

2

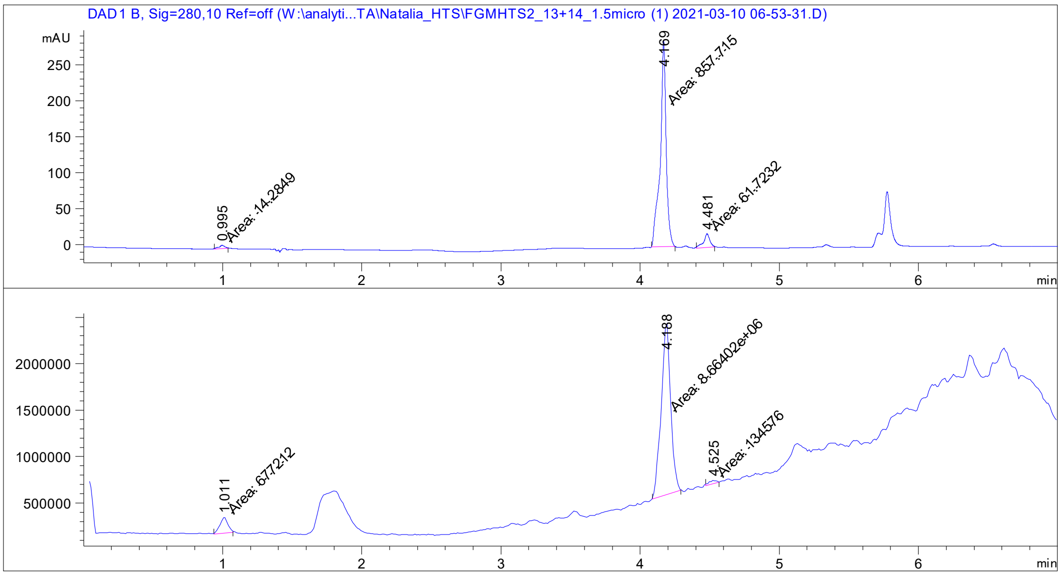

MS Signal: MSD1 TIC, MS File, ES-API, Pos, Scan, Frag: 70, "PosScan"
Spectra averaged over upper half of peaks.
Noise Cutoff: 1000 counts.
Reportable Ion Abundance: > 10%.

Retention Time (MS)	MS Area	Mol. Weight or Ion
1.011	677212	330.95 I
		329.00 I
		283.20 I
		282.25 I
		280.10 I
		279.00 I
		256.10 I
		227.95 I
		223.95 I
		200.95 I
		199.95 I
		186.85 I
		184.95 I
		183.90 I
		182.90 I
		177.90 I
		175.95 I
		174.00 I
		172.00 I
		158.95 I
		150.05 I
		146.05 I
		144.00 I
		142.00 I
		137.95 I
		130.95 I
		128.95 I
		124.10 I
		123.05 I
		112.00 I
		110.90 I
		110.05 I
		104.05 I
		102.10 I
		100.95 I
4.188	8664020	310.00 I
		309.05 I
4.525	134576	316.25 I
		298.25 I
		296.20 I
		283.25 I
		282.25 I
		280.15 I
		279.05 I
		268.20 I
		256.20 I

172.10 I

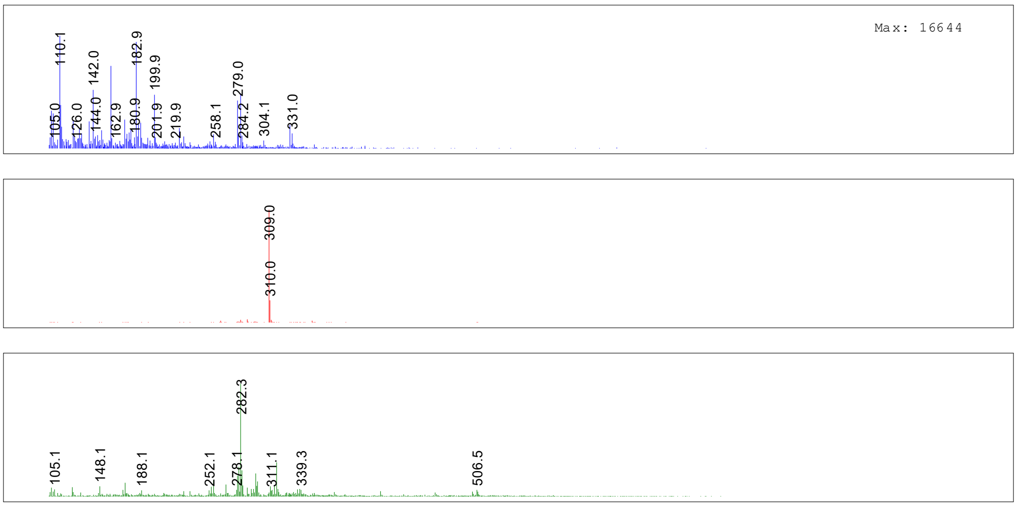

*** End of Report ***

Compound 3

Data File W:\analytical_LCMS_DATA\Natalia_HTS\FGHTS2_vial13 (1) 2021-03-09 10-41-47.D
Sample Name: FGHTS2_vial13

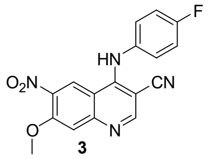

3

===

Acq. Operator : SYSTEM
Sample Operator : SYSTEM
Acq. Instrument : LCMS test Location : D1F-F5
Injection Date : 3/9/2021 1:44:54 PM Inj : 1
Inj Volume : 1.000 µl
Different Inj Volume from Sample Entry! Actual Inj Volume : 1.200 µl
Acq. Method : C:\Users\Public\Documents\ChemStation\1\Methods\0-Std_mth\Gradient\FAST_Nonpolar_General_Method_MS-PosScan-100-1000.M
Last changed : 2/18/2021 11:58:42 AM by SYSTEM
Analysis Method : C:\Users\Public\Documents\ChemStation\1\Methods\DEF_LC.M
Last changed : 9/20/2021 3:18:40 PM by SYSTEM
(modified after loading)
Additional Info : Peak(s) manually integrated

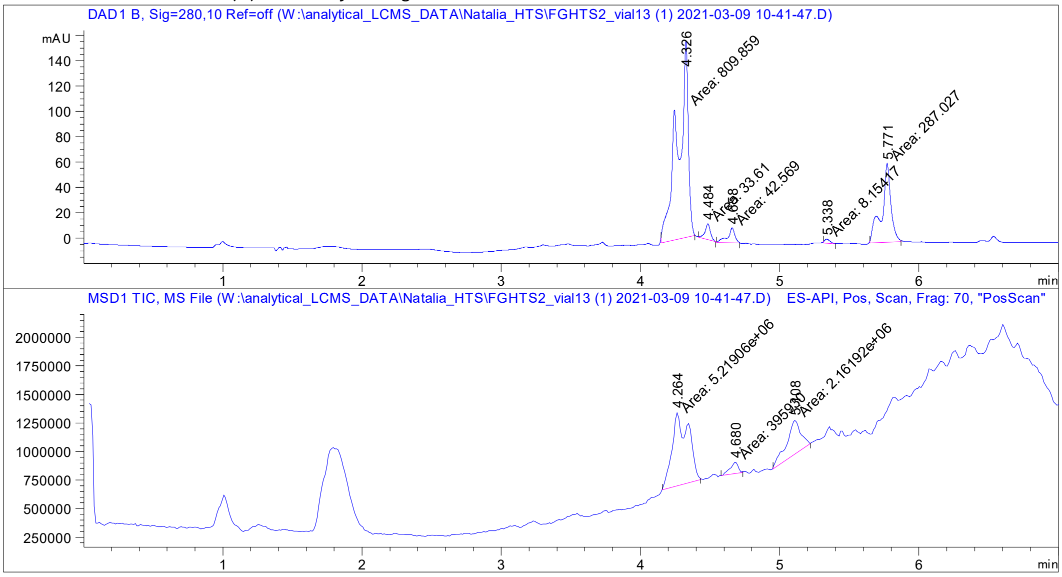

Data File W:\analytical_LCMS_DATA\Natalia_HTS\FGHTS2_vial13 (1) 2021-03-09 10-41-47.D
Sample Name: FGHTS2_vial13

MS Signal: MSD1 TIC, MS File, ES-API, Pos, Scan, Frag: 70, "PosScan"
Spectra averaged over upper half of peaks.
Noise Cutoff: 1000 counts.
Reportable Ion Abundance: > 10%.

Retention Time (MS)	MS Area	Mol. Weight or Ion
4.264	5219058	340.00 I
		339.00 I
		282.20 I
		265.95 I
		264.00 I
4.680	395930	506.50 I
		451.15 I
		331.05 I
		329.00 I
		316.25 I
		313.10 I
		310.15 I
		309.05 I
		296.20 I
		283.25 I
		282.25 I
		280.20 I
		279.05 I
		268.15 I
		263.05 I
		256.20 I
		254.20 I
		172.15 I
5.108	2161918	327.35 I
		326.35 I
		296.20 I
		283.25 I
		282.20 I
		280.20 I

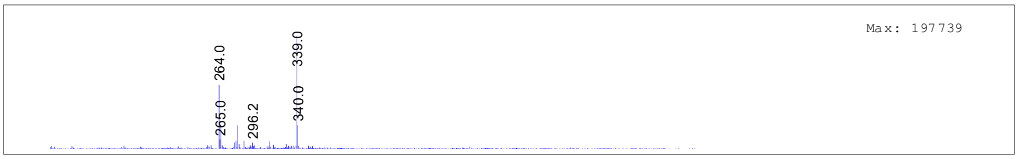

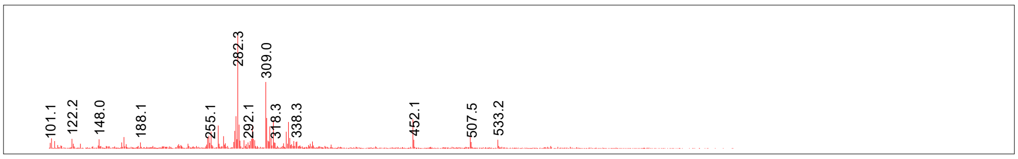

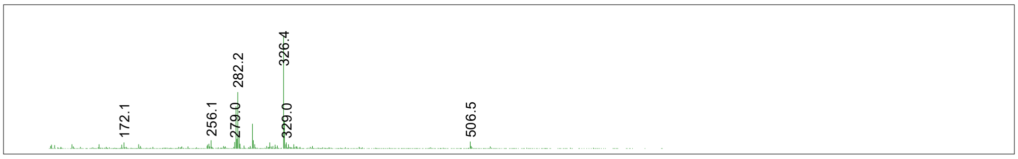

*** End of Report ***

Compound 4

Data File W:\analytical_LCMS_DATA\Natalia_HTS\FGMHTS2_13+11 (1) 2021-03-23 07-41-57.D
Sample Name: FGMHTS2_13+11

===

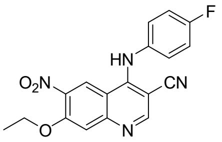

4

Acq. Operator : SYSTEM
Sample Operator : SYSTEM
Acq. Instrument : LCMS test   Location : D1B-B1
Injection Date : 3/23/2021 8:25:03 AM   Inj : 1
Inj Volume : 1.000 µl
Different Inj Volume from Sample Entry!   Actual Inj Volume : 1.500 µl
Acq. Method : C:\Users\Public\Documents\ChemStation\1\Methods\0-Std_mth\Gradient\FAST_Nonpolar_General_Method_MS-PosScan-100-1000.M
Last changed : 2/18/2021 11:58:42 AM by SYSTEM
Analysis Method : C:\Users\Public\Documents\ChemStation\1\Methods\0-Std_mth\Gradient\SLOW_Nonpolar_General_Method_MS-PosScan-100-1000.M
Last changed : 4/21/2021 11:43:02 AM by SYSTEM
(modified after loading)
Method Info : General method-use for RX monitoring

Additional Info : Peak(s) manually integrated

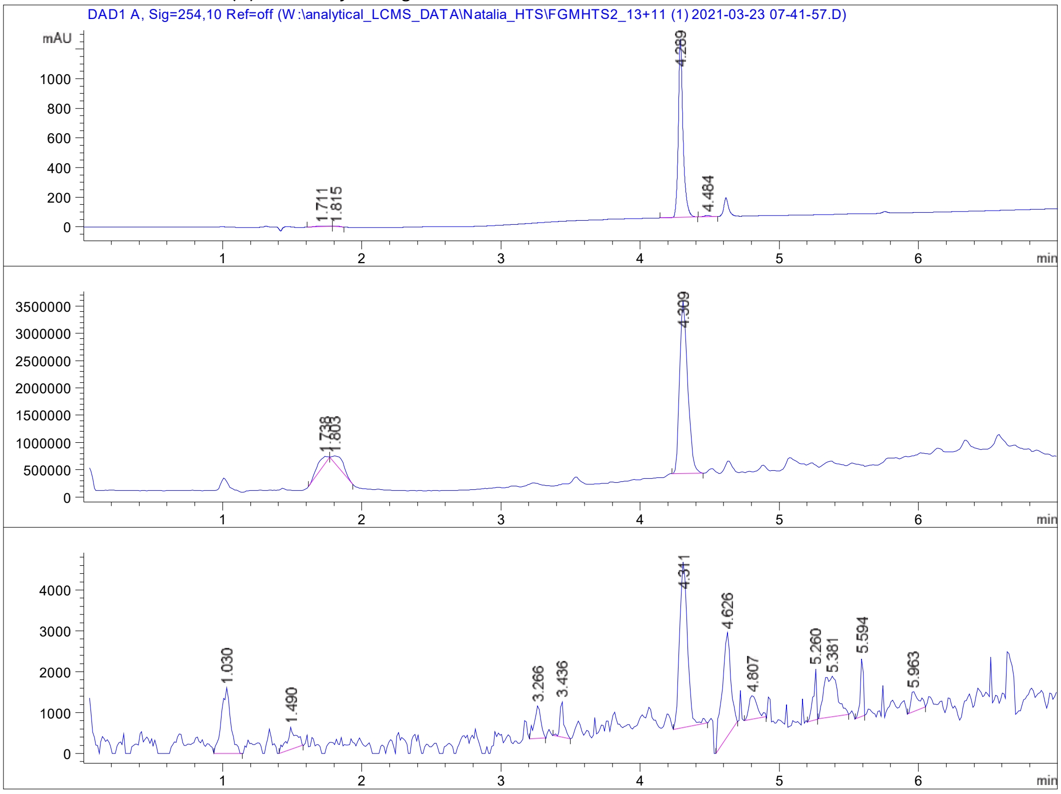

Data File W:\analytical_LCMS_DATA\Natalia_HTS\FGMHTS2_13+11 (1) 2021-03-23 07-41-57.D
Sample Name: FGMHTS2_13+11

MS Signal: MSD1 TIC, MS File, ES-API, Pos, Scan, Frag: 70, "PosScan"
Spectra averaged over upper half of peaks.
Noise Cutoff: 1000 counts.
Reportable Ion Abundance: > 10%.

Retention Time (MS)	MS Area	Mol. Weight or Ion
1.738	912064	112.10 I
1.803	1048126	112.10 I
4.309	12871998	354.05 I<br>353.05 I

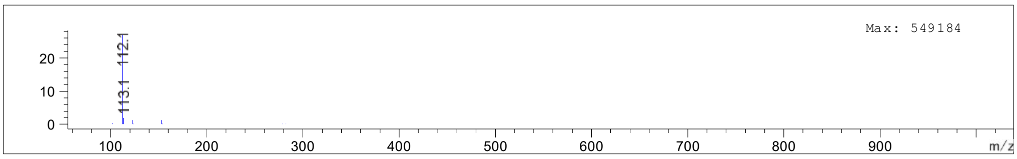

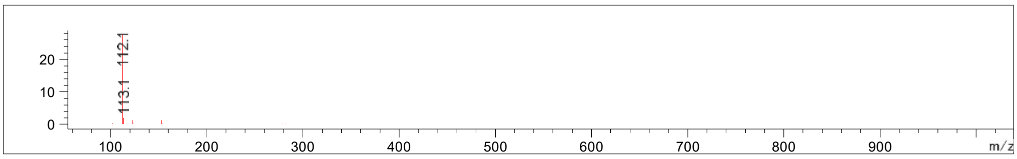

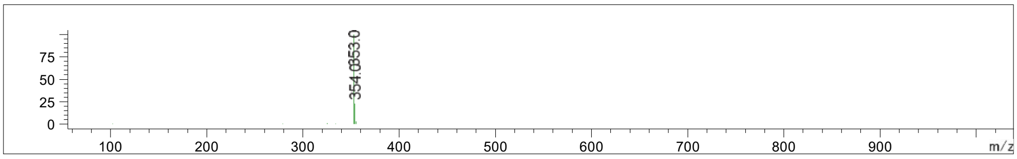

*** End of Report ***

Compound 5

```
Data File W:\analyti...MS_DATA\Natalia_HTS\FGMHTS2_6+14_1.5micro (1) 2021-03-10 06-43-09.D
Sample Name: FGMHTS2_6+14_1.5micro

    ====================================================================
    Acq. Operator   : SYSTEM
    Sample Operator : SYSTEM
    Acq. Instrument : LCMS test                      Location :   D1F-B6
    Injection Date  : 3/10/2021 7:09:20 AM                Inj :   1
                                                   Inj Volume : 1.000 µl
    Different Inj Volume from Sample Entry!   Actual Inj Volume : 1.500 µl
    Acq. Method     : C:\Users\Public\Documents\ChemStation\1\Methods\0-Std_mth\Gradient\FAST_
                      Nonpolar_General_Method_MS-PosScan-100-1000.M
    Last changed    : 2/18/2021 11:58:42 AM by SYSTEM
    Analysis Method : C:\Users\Public\Documents\ChemStation\1\Methods\DEF_LC.M
    Last changed    : 9/20/2021 11:46:17 AM by SYSTEM
                      (modified after loading)
    Additional Info : Peak(s) manually integrated
```

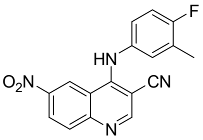

5

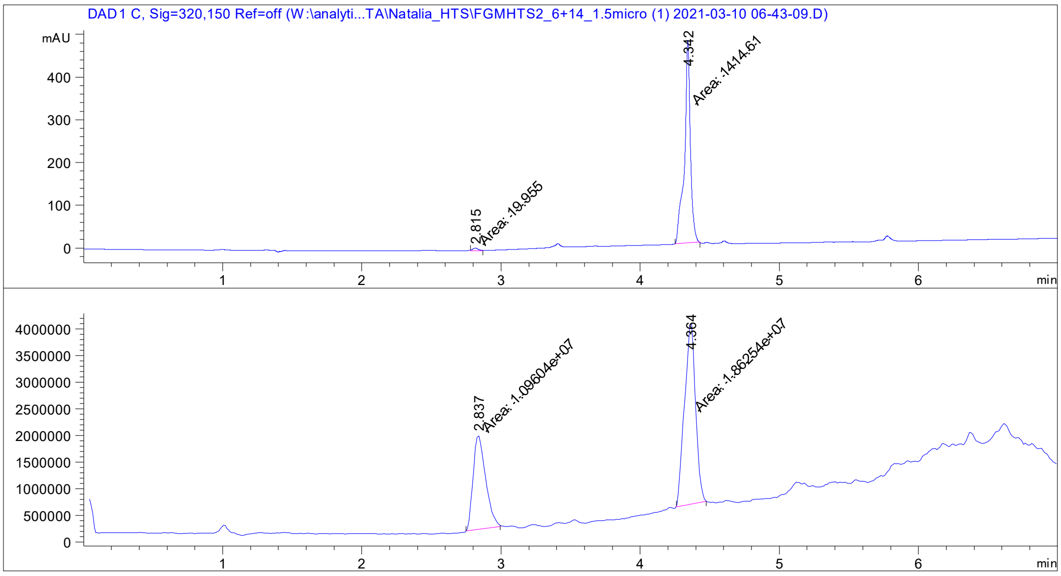

MS Signal: MSD1 TIC, MS File, ES-API, Pos, Scan, Frag: 70, "PosScan"
Spectra averaged over upper half of peaks.
Noise Cutoff: 1000 counts.
Reportable Ion Abundance: > 10%.

Retention Time (MS)	MS Area	Mol. Weight or Ion
2.837	10960398	126.10 I
4.364	18625436	324.05 I
		323.05 I

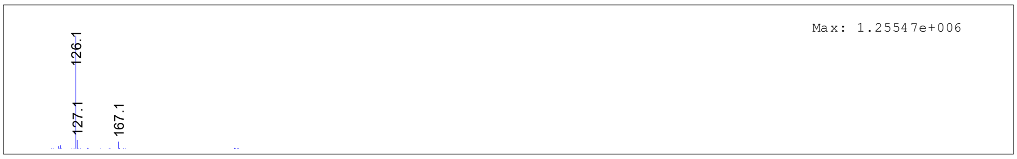

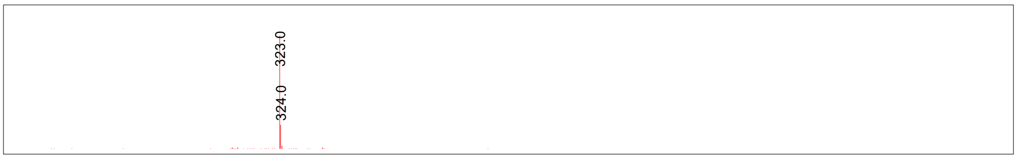

*** End of Report ***

Compound 6

```
Data File W:\analytical_LCMS_DATA\Natalia_HTS\FGHTS2_vial6slow (1) 2021-03-09 10-40-23.D
Sample Name: FGHTS2_vial6slow

    =====================================================================
    Acq. Operator   : SYSTEM
    Sample Operator : SYSTEM
    Acq. Instrument : LCMS test                    Location :   D1F-F1
    Injection Date  : 3/9/2021 11:54:38 AM              Inj :   1
                                                 Inj Volume : 1.000 µl
    Different Inj Volume from Sample Entry!   Actual Inj Volume : 1.200 µl
    Acq. Method     : C:\Users\Public\Documents\ChemStation\1\Methods\0-Std_mth\Gradient\SLOW_
                      Nonpolar_General_Method_MS-PosScan-100-1000.M
    Last changed    : 2/18/2021 12:01:57 PM by SYSTEM
    Analysis Method : C:\Users\Public\Documents\ChemStation\1\Methods\DEF_LC.M
    Last changed    : 9/20/2021 3:18:40 PM by SYSTEM
                      (modified after loading)
    Additional Info : Peak(s) manually integrated
```

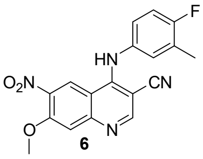

6

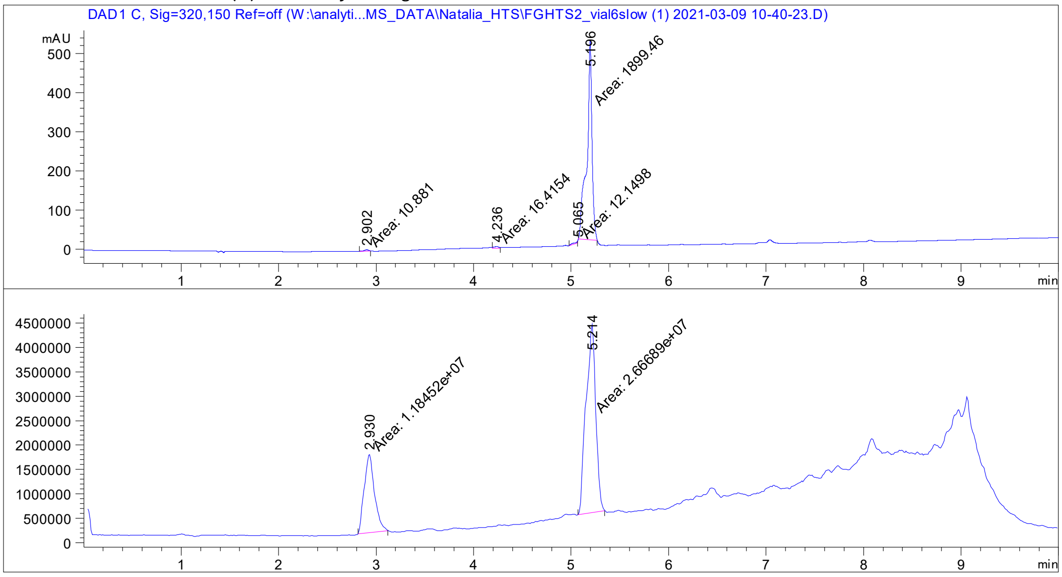

Data File W:\analytical_LCMS_DATA\Natalia_HTS\FGHTS2_vial6slow (1) 2021-03-09 10-40-23.D
Sample Name: FGHTS2_vial6slow

MS Signal: MSD1 TIC, MS File, ES-API, Pos, Scan, Frag: 70, "PosScan"
Spectra averaged over upper half of peaks.
Noise Cutoff: 1000 counts.
Reportable Ion Abundance: > 10%.

Retention Time (MS)	MS Area	Mol. Weight or Ion
2.930	11845172	126.15 I
5.214	26668852	354.05 I
		353.05 I

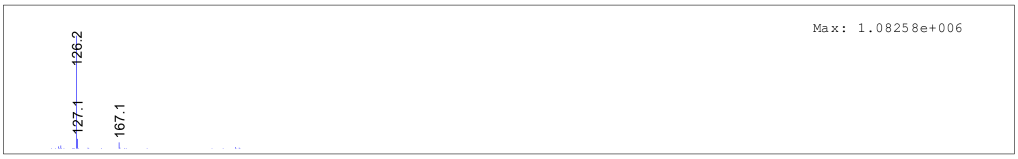

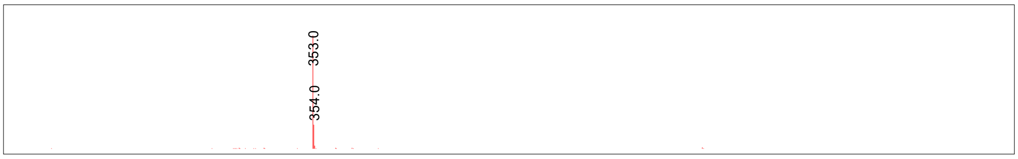

*** End of Report ***

Compound 7

```
Data File W:\analytical_LCMS_DATA\Natalia_HTS\FGMHTS2_11+6_2 (1) 2021-03-23 08-27-29.D
Sample Name: FGMHTS2_11+6_2

    =====================================================================
    Acq. Operator    : SYSTEM
    Sample Operator  : SYSTEM
    Acq. Instrument  : LCMS test                       Location :   D1B-B6
    Injection Date   : 3/23/2021 9:41:34 AM                 Inj :   1
                                                     Inj Volume : 1.000 µl
    Different Inj Volume from Sample Entry!   Actual Inj Volume : 1.500 µl
    Acq. Method      : C:\Users\Public\Documents\ChemStation\1\Methods\0-Std_mth\Gradient\FAST_
                       Nonpolar_General_Method_MS-PosScan-100-1000.M
    Last changed     : 2/18/2021 11:58:42 AM by SYSTEM
    Analysis Method  : C:\Users\Public\Documents\ChemStation\1\Methods\DEF_LC.M
    Last changed     : 9/20/2021 11:46:17 AM by SYSTEM
                       (modified after loading)
    Additional Info  : Peak(s) manually integrated
```

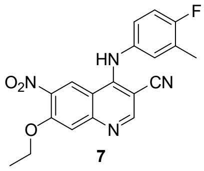

7

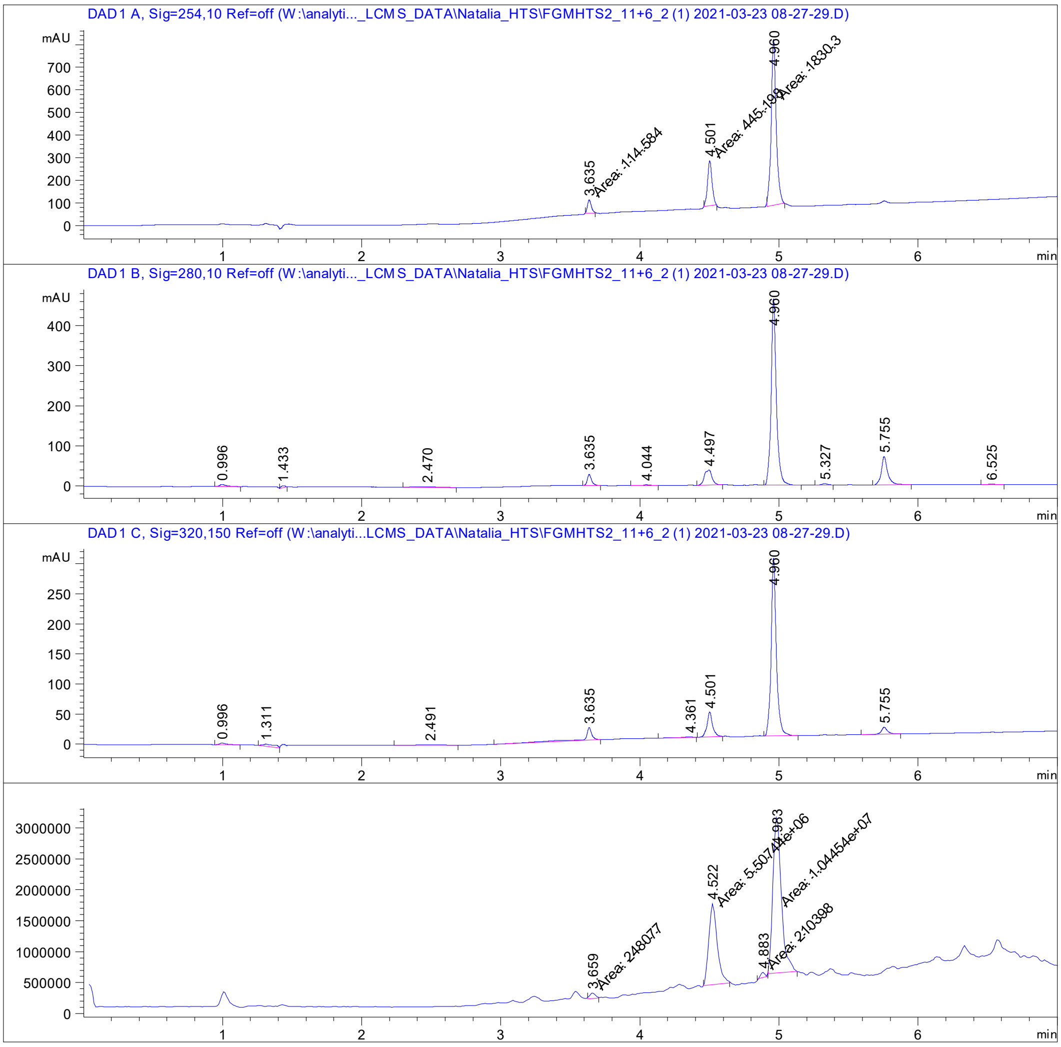

Data File W:\analytical_LCMS_DATA\Natalia_HTS\FGMHTS2_11+6_2 (1) 2021-03-23 08-27-29.D
Sample Name: FGMHTS2_11+6_2

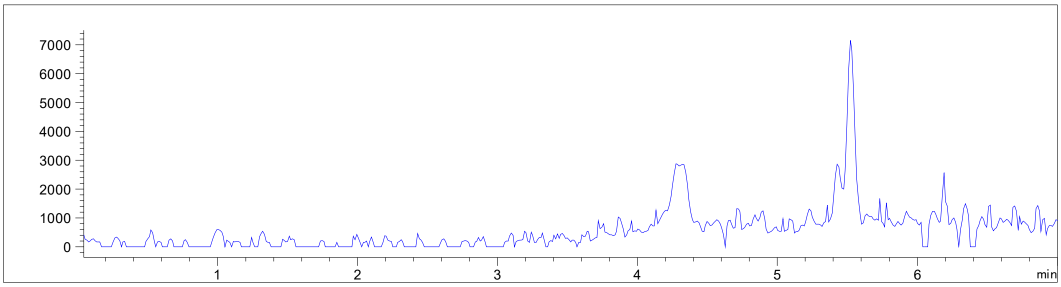

Data File W:\analytical_LCMS_DATA\Natalia_HTS\FGMHTS2_11+6_2 (1) 2021-03-23 08-27-29.D
Sample Name: FGMHTS2_11+6_2

MS Signal: MSD1 TIC, MS File, ES-API, Pos, Scan, Frag: 70, "PosScan"
Spectra averaged over upper half of peaks.
Noise Cutoff: 1000 counts.
Reportable Ion Abundance: > 10%.

Retention Time (MS)	MS Area	Mol. Weight or Ion
3.659	248077	282.15 I
		260.95 I
		260.00 I
		200.15 I
		102.20 I
4.522	5507438	205.00 I
		202.95 I
		165.95 I
		164.00 I
		161.95 I
4.883	210398	403.00 I
		282.20 I
		280.15 I
		217.00 I
		216.05 I
		102.25 I
4.983	10445387	407.00 I
		406.05 I
		405.00 I
		404.00 I
		403.05 I

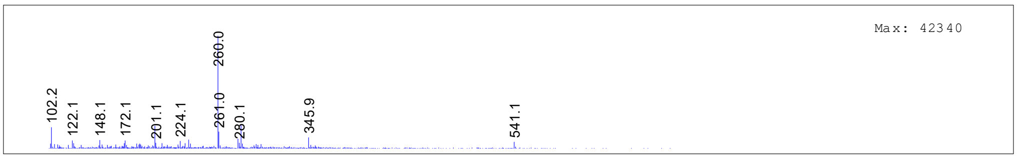

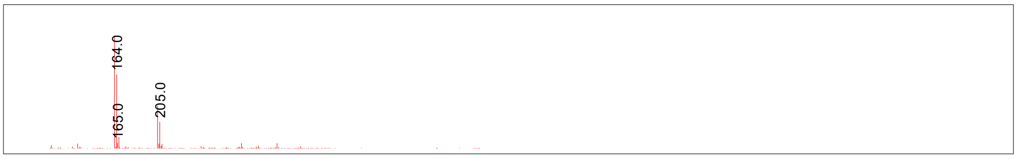

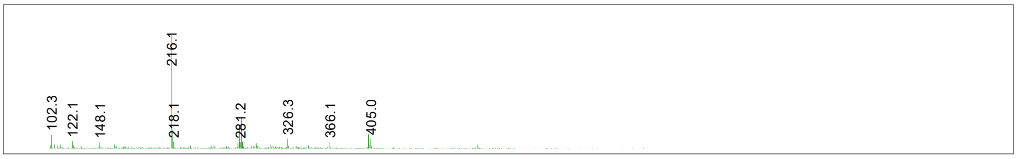

Data File W:\analytical_LCMS_DATA\Natalia_HTS\FGMHTS2_11+6_2 (1) 2021-03-23 08-27-29.D
Sample Name: FGMHTS2_11+6_2

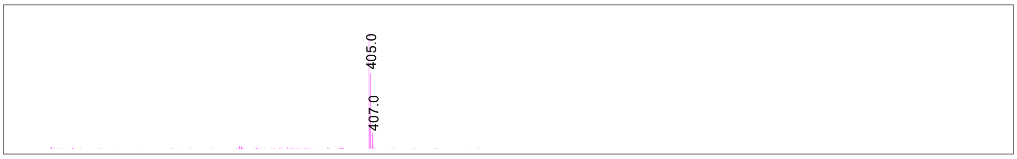

*** End of Report ***

Compound 8

```
Data File W:\analyti...MS_DATA\Natalia_HTS\FGMHTS2_7+14_1.5micro (1) 2021-03-10 06-42-55.D
Sample Name: FGMHTS2_7+14_1.5micro

    =====================================================================
    Acq. Operator   : SYSTEM
    Sample Operator : SYSTEM
    Acq. Instrument : LCMS test                      Location :   D1F-B7
    Injection Date  : 3/10/2021 7:19:48 AM                Inj :   1
                                                   Inj Volume : 1.000 µl
    Different Inj Volume from Sample Entry!  Actual Inj Volume : 1.500 µl
    Acq. Method     : C:\Users\Public\Documents\ChemStation\1\Methods\0-Std_mth\Gradient\FAST_
                      Nonpolar_General_Method_MS-PosScan-100-1000.M
    Last changed    : 2/18/2021 11:58:42 AM by SYSTEM
    Analysis Method : C:\Users\Public\Documents\ChemStation\1\Methods\DEF_LC.M
    Last changed    : 9/20/2021 11:46:17 AM by SYSTEM
                      (modified after loading)
    Additional Info : Peak(s) manually integrated
```

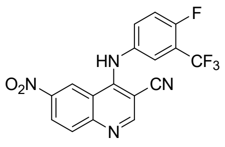

8

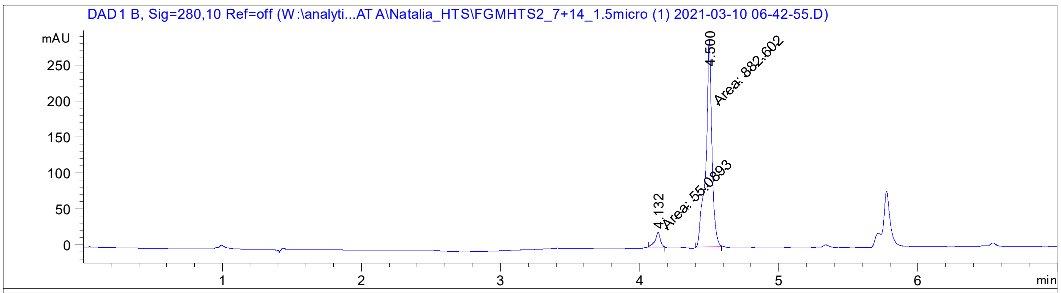

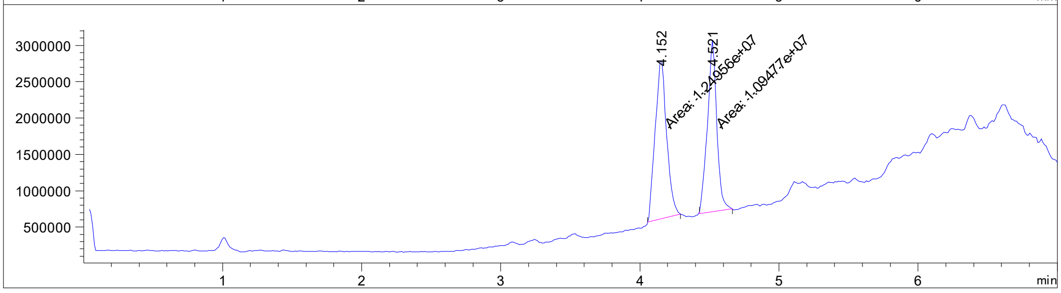

Data File W:\analyti...MS_DATA\Natalia_HTS\FGMHTS2_7+14_1.5micro (1) 2021-03-10 06-42-55.D
Sample Name: FGMHTS2_7+14_1.5micro

MS Signal: MSD1 TIC, MS File, ES-API, Pos, Scan, Frag: 70, "PosScan"
Spectra averaged over upper half of peaks.
Noise Cutoff: 1000 counts.
Reportable Ion Abundance: > 10%.

Retention Time (MS)	MS Area	Mol. Weight or Ion
4.152	12495627	221.05 I
		180.05 I
4.521	10947695	378.00 I
		377.05 I

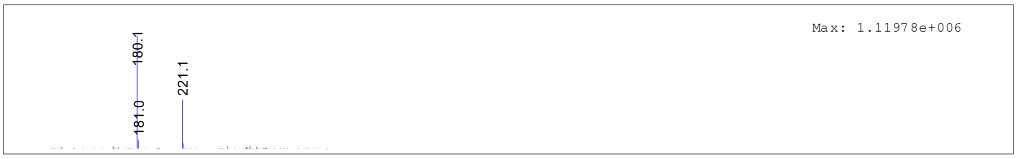

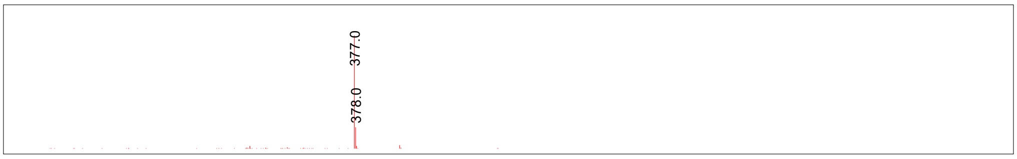

*** End of Report ***

Compound 9

```
Data File W:\analytical_LCMS_DATA\Natalia_HTS\FGHTS2_vial7 (1) 2021-03-09 10-40-50.D
Sample Name: FGHTS2_vial7

    =====================================================================
    Acq. Operator   : SYSTEM
    Sample Operator : SYSTEM
    Acq. Instrument : LCMS test                       Location :   D1F-F2
    Injection Date  : 3/9/2021 12:18:32 PM                 Inj :   1
                                                    Inj Volume : 1.000 µl
    Different Inj Volume from Sample Entry!   Actual Inj Volume : 1.200 µl
    Acq. Method     : C:\Users\Public\Documents\ChemStation\1\Methods\0-Std_mth\Gradient\FAST_
                      Nonpolar_General_Method_MS-PosScan-100-1000.M
    Last changed    : 2/18/2021 11:58:42 AM by SYSTEM
    Analysis Method : C:\Users\Public\Documents\ChemStation\1\Methods\DEF_LC.M
    Last changed    : 9/20/2021 3:18:40 PM by SYSTEM
                      (modified after loading)
    Additional Info : Peak(s) manually integrated
```

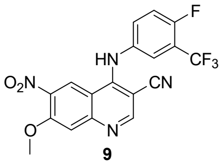

9

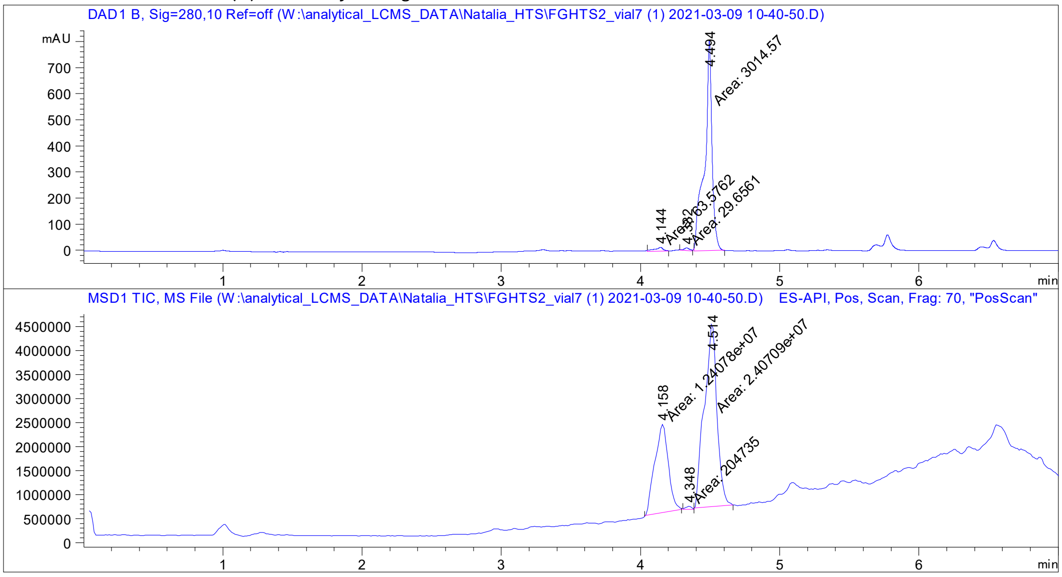

Data File W:\analytical_LCMS_DATA\Natalia_HTS\FGHTS2_vial7 (1) 2021-03-09 10-40-50.D
Sample Name: FGHTS2_vial7

MS Signal: MSD1 TIC, MS File, ES-API, Pos, Scan, Frag: 70, "PosScan"
Spectra averaged over upper half of peaks.
Noise Cutoff: 1000 counts.
Reportable Ion Abundance: > 10%.

Retention Time (MS)	MS Area	Mol. Weight or Ion
4.158	12407832	221.00 I
		179.95 I
4.348	204735	426.00 I
		298.20 I
		297.20 I
		296.20 I
		288.20 I
		283.25 I
		282.25 I
		280.15 I
		279.10 I
		266.00 I
		264.00 I
		256.20 I
		221.05 I
		180.00 I
		172.15 I
		102.15 I
4.514	24070850	408.00 I
		407.05 I

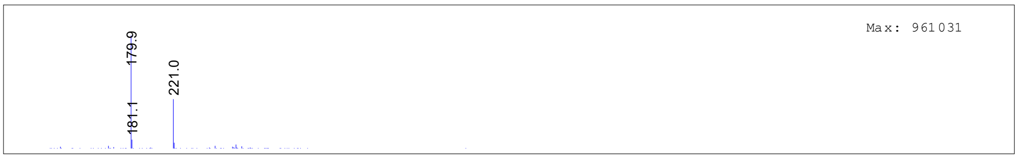

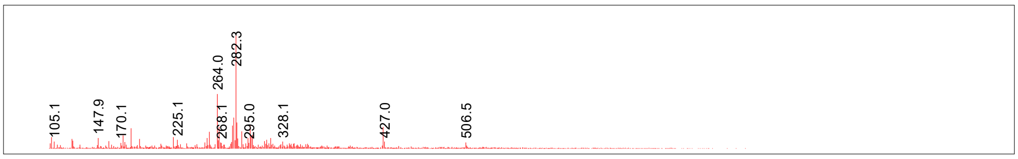

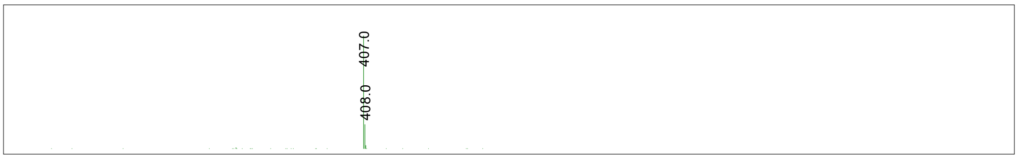

*** End of Report ***

Compound 10

```
Data File W:\analytical_LCMS_DATA\Natalia_HTS\FGMHTS2_7+11 (1) 2021-03-23 07-25-01.D
Sample Name: FGMHTS2_7+11

=====================================================================
Acq. Operator   : SYSTEM
Sample Operator : SYSTEM
Acq. Instrument : LCMS test                    Location :   D1B-A7
Injection Date  : 3/23/2021 7:53:44 AM              Inj :   1
                                             Inj Volume : 1.000 µl
Different Inj Volume from Sample Entry!   Actual Inj Volume : 1.500 µl
Acq. Method     : C:\Users\Public\Documents\ChemStation\1\Methods\0-Std_mth\Gradient\FAST_
                  Nonpolar_General_Method_MS-PosScan-100-1000.M
Last changed    : 2/18/2021 11:58:42 AM by SYSTEM
Analysis Method : C:\Users\Public\Documents\ChemStation\1\Methods\0-Std_mth\Gradient\SLOW_
                  Nonpolar_General_Method_MS-PosScan-100-1000.M
Last changed    : 4/22/2021 3:11:23 PM by SYSTEM
                  (modified after loading)
Method Info     : General method-use for RX monitoring

Additional Info : Peak(s) manually integrated
```

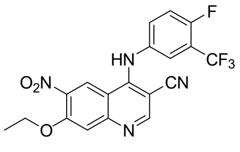

10

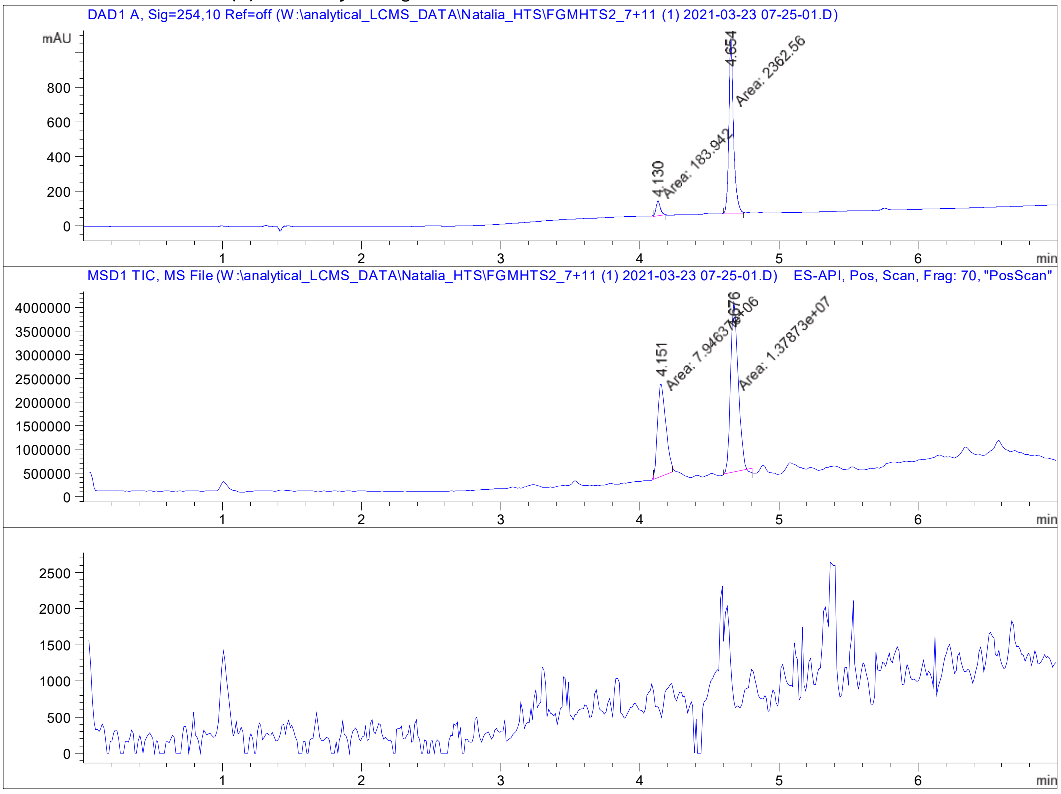

Data File W:\analytical_LCMS_DATA\Natalia_HTS\FGMHTS2_7+11 (1) 2021-03-23 07-25-01.D
Sample Name: FGMHTS2_7+11

MS Signal: MSD1 TIC, MS File, ES-API, Pos, Scan, Frag: 70, "PosScan"
Spectra averaged over upper half of peaks.
Noise Cutoff: 1000 counts.
Reportable Ion Abundance: > 10%.

Retention Time (MS)	MS Area	Mol. Weight or Ion
4.151	7946371	221.05 I
		180.00 I
4.676	13787323	422.05 I
		421.05 I

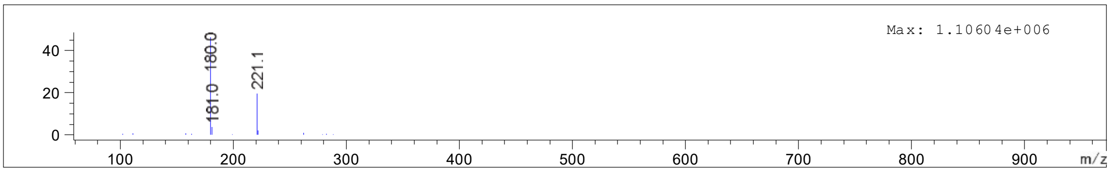

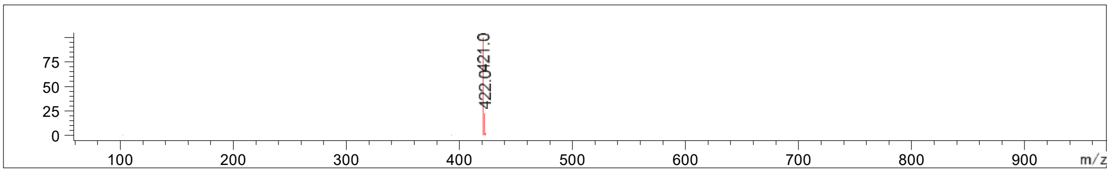

*** End of Report ***

Compound 11

```
Data File W:\analyti...S_DATA\Natalia_HTS\FGMHTS2_15+14_1.5micro (1) 2021-03-10 06-53-42.D
Sample Name: FGMHTS2_15+14_1.5micro

    =====================================================================
    Acq. Operator   : SYSTEM
    Sample Operator : SYSTEM
    Acq. Instrument : LCMS test                     Location :   D1F-C2
    Injection Date  : 3/10/2021 8:01:32 AM               Inj :   1
                                                  Inj Volume : 1.000 µl
    Different Inj Volume from Sample Entry!   Actual Inj Volume : 1.500 µl
    Acq. Method     : C:\Users\Public\Documents\ChemStation\1\Methods\0-Std_mth\Gradient\FAST_
                      Nonpolar_General_Method_MS-PosScan-100-1000.M
    Last changed    : 2/18/2021 11:58:42 AM by SYSTEM
    Analysis Method : C:\Users\Public\Documents\ChemStation\1\Methods\DEF_LC.M
    Last changed    : 9/20/2021 11:46:17 AM by SYSTEM
                      (modified after loading)
    Additional Info : Peak(s) manually integrated
```

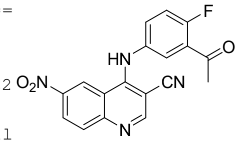

11

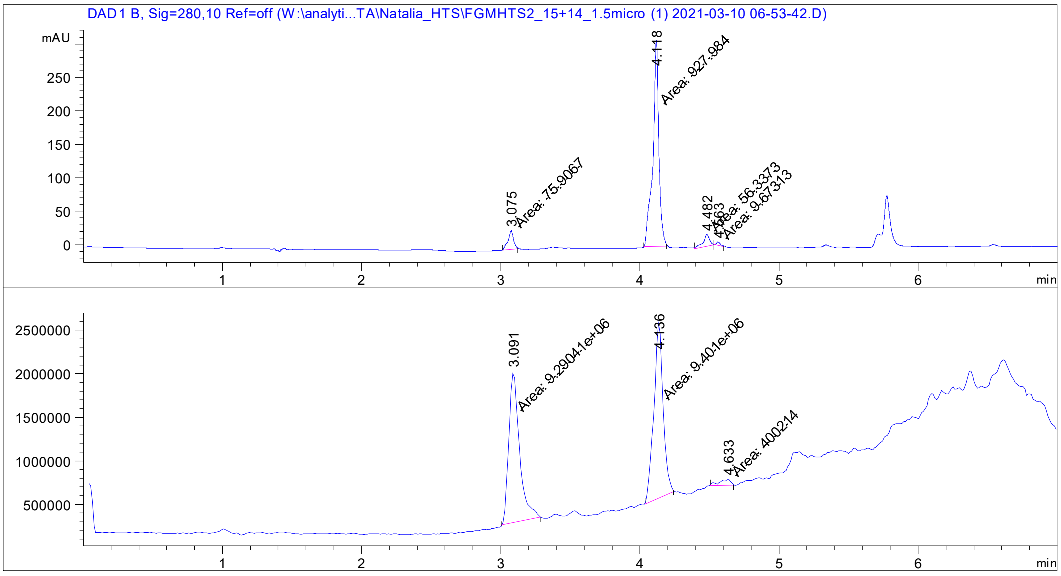

Data File W:\analyti...S_DATA\Natalia_HTS\FGMHTS2_15+14_1.5micro (1) 2021-03-10 06-53-42.D
Sample Name: FGMHTS2_15+14_1.5micro

MS Signal: MSD1 TIC, MS File, ES-API, Pos, Scan, Frag: 70, "PosScan"
Spectra averaged over upper half of peaks.
Noise Cutoff: 1000 counts.
Reportable Ion Abundance: > 10%.

Retention Time (MS)	MS Area	Mol. Weight or Ion
3.091	9290406	195.05 I
		154.05 I
4.136	9400996	352.00 I
		351.00 I
4.633	400214	484.10 I
		316.30 I
		310.15 I
		297.20 I
		296.25 I
		295.10 I
		284.15 I
		283.20 I
		282.25 I
		280.20 I
		279.05 I
		268.15 I
		256.20 I
		254.20 I
		172.10 I

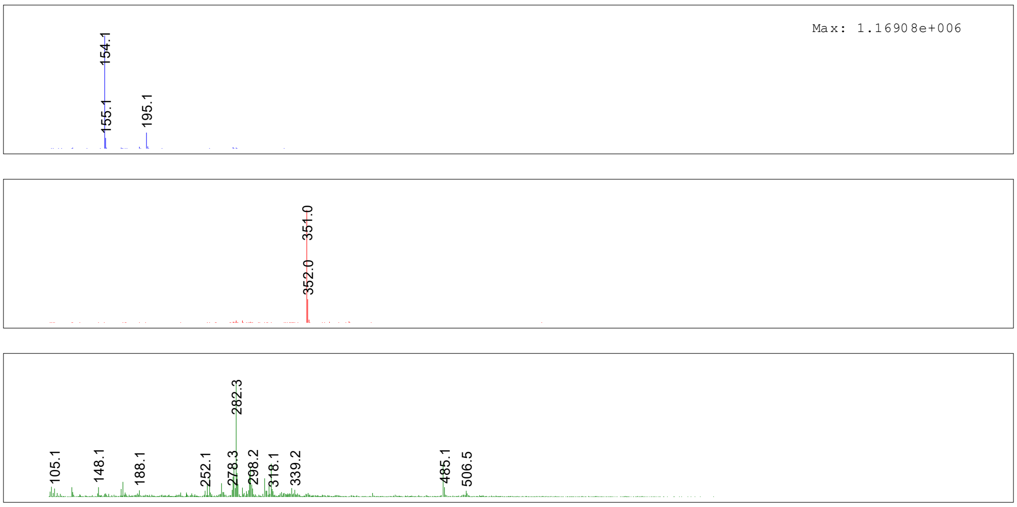

*** End of Report ***

Compound 12

```
Data File W:\analytical_LCMS_DATA\Natalia_HTS\FGHTS2_vial12+15 (1) 2021-03-16 07-51-02.D
Sample Name: FGHTS2_vial12+15

    =================================================================
    Acq. Operator   : SYSTEM
    Sample Operator : SYSTEM
    Acq. Instrument : LCMS test                        Location :   D1B-A5
    Injection Date  : 3/16/2021 9:58:47 AM                  Inj :   1
                                                     Inj Volume : 1.000 µl
    Different Inj Volume from Sample Entry!   Actual Inj Volume : 1.500 µl
    Acq. Method     : C:\Users\Public\Documents\ChemStation\1\Methods\0-Std_mth\Gradient\FAST_
                      Nonpolar_General_Method_MS-PosScan-100-1000.M
    Last changed    : 2/18/2021 11:58:42 AM by SYSTEM
    Analysis Method : C:\Users\Public\Documents\ChemStation\1\Methods\DEF_LC.M
    Last changed    : 9/20/2021 3:18:40 PM by SYSTEM
                      (modified after loading)
    Additional Info : Peak(s) manually integrated
```

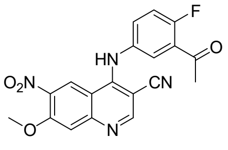

12

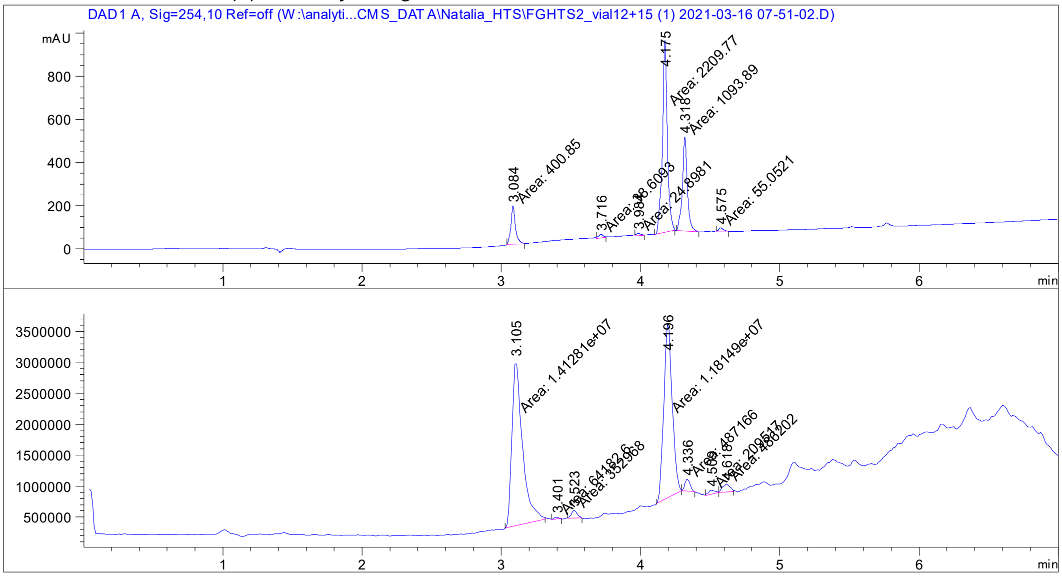

Data File W:\analytical_LCMS_DATA\Natalia_HTS\FGHTS2_vial12+15 (1) 2021-03-16 07-51-02.D
Sample Name: FGHTS2_vial12+15

MS Signal: MSD1 TIC, MS File, ES-API, Pos, Scan, Frag: 70, "PosScan"
Spectra averaged over upper half of peaks.
Noise Cutoff: 1000 counts.
Reportable Ion Abundance: > 10%.

Retention Time (MS)	MS Area	Mol. Weight or Ion
3.105	14128111	195.05 I
		154.10 I
3.401	64183	287.05 I
		282.20 I
		279.05 I
		195.00 I
		163.10 I
		155.10 I
		154.05 I
		148.10 I
		102.20 I
3.523	352968	284.20 I
		282.20 I
		279.05 I
		230.15 I
		229.10 I
		224.15 I
		208.10 I
		207.10 I
		195.00 I
		163.15 I
		154.10 I
		102.20 I
4.196	11814914	382.10 I
		381.10 I
4.336	487166	381.05 I
		296.20 I
		284.25 I
		282.25 I
		280.20 I
		279.05 I
		265.95 I
		265.10 I
		264.00 I
		102.20 I
4.509	209517	326.20 I
		316.25 I
		314.25 I
		312.25 I
		311.15 I
		310.25 I
		298.20 I
		297.20 I

		296.20	I
		294.20	I
		284.30	I
		283.20	I
		282.20	I
		281.25	I
		280.20	I
		279.05	I
		278.20	I
		268.20	I
		256.25	I
		254.10	I
		252.05	I
		172.05	I
		163.20	I
		148.15	I
		122.10	I
		102.20	I
4.618	486202	515.15	I
		514.15	I
		414.15	I
		326.20	I
		316.20	I
		314.20	I
		312.25	I
		311.30	I
		310.20	I
		298.15	I
		297.20	I
		296.25	I
		295.20	I
		294.25	I
		284.25	I
		283.20	I
		282.25	I
		280.20	I
		279.10	I
		268.20	I
		256.20	I
		254.15	I
		251.95	I
		172.05	I
		148.15	I
		122.15	I
		102.20	I

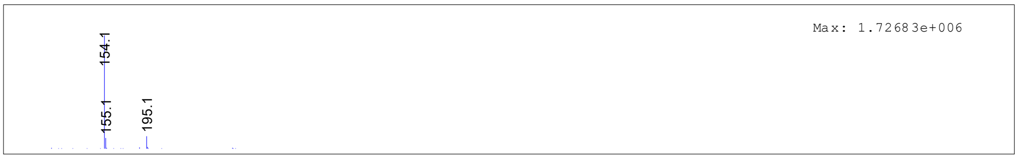

Data File W:\analytical_LCMS_DATA\Natalia_HTS\FGHTS2_vial12+15 (1) 2021-03-16 07-51-02.D
Sample Name: FGHTS2_vial12+15

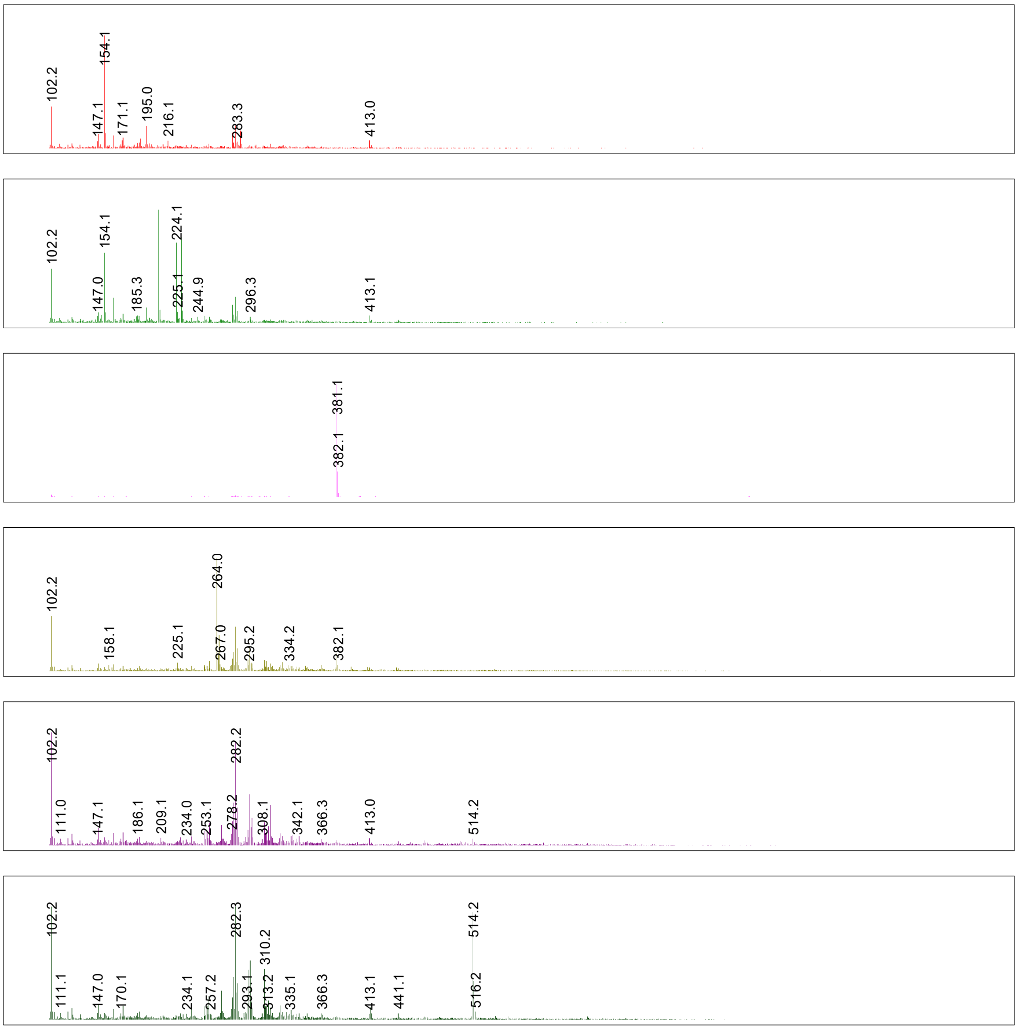

*** End of Report ***

Compound 13

Data File W:\analytical_LCMS_DATA\Natalia_HTS\FGMHTS2_15+11 (1) 2021-03-23 08-27-58.D
Sample Name: FGMHTS2_15+11

===
Acq. Operator : SYSTEM
Sample Operator : SYSTEM
Acq. Instrument : LCMS test   Location : D1B-B2
Injection Date : 3/23/2021 8:35:29 AM   Inj : 1
                Inj Volume : 1.000 µl
Different Inj Volume from Sample Entry!   Actual Inj Volume : 1.500 µl
Acq. Method : C:\Users\Public\Documents\ChemStation\1\Methods\0-Std_mth\Gradient\FAST_Nonpolar_General_Method_MS-PosScan-100-1000.M
Last changed : 2/18/2021 11:58:42 AM by SYSTEM
Analysis Method : C:\Users\Public\Documents\ChemStation\1\Methods\0-Std_mth\Gradient\SLOW_Nonpolar_General_Method_MS-PosScan-100-1000.M
Last changed : 4/22/2021 2:41:27 PM by SYSTEM
(modified after loading)
Method Info : General method-use for RX monitoring

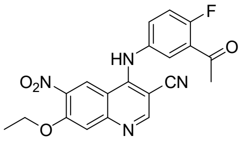

13

Additional Info : Peak(s) manually integrated

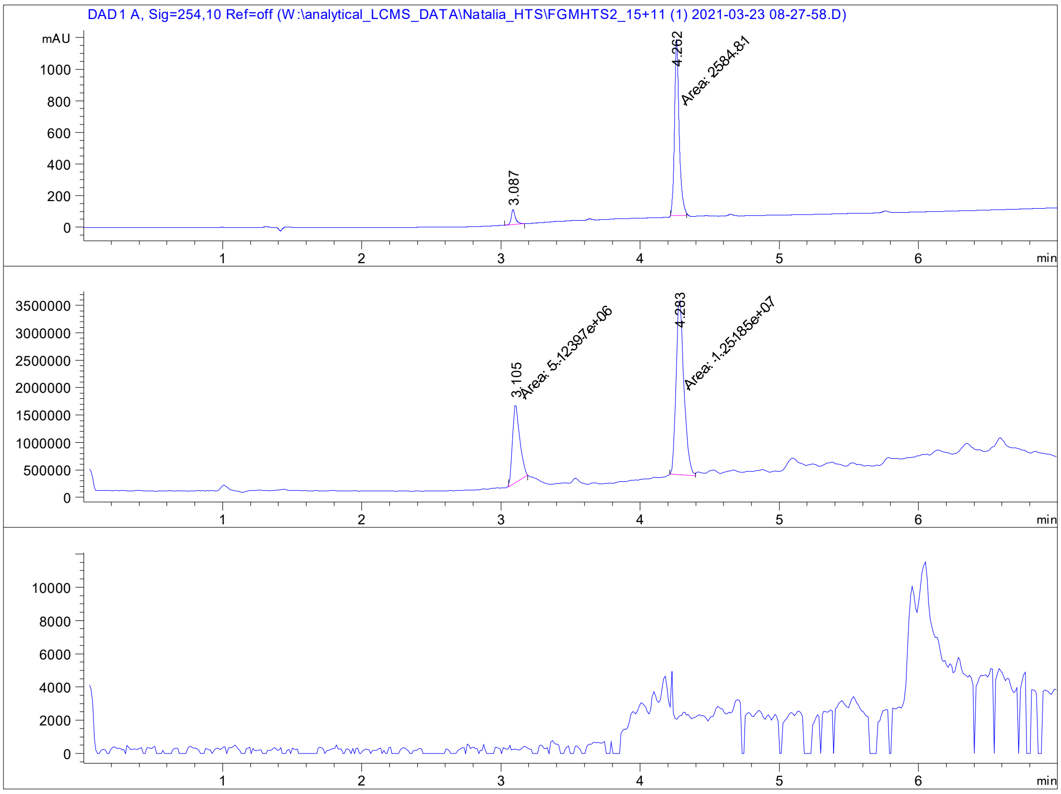

Data File W:\analytical_LCMS_DATA\Natalia_HTS\FGMHTS2_15+11 (1) 2021-03-23 08-27-58.D
Sample Name: FGMHTS2_15+11

MS Signal: MSD1 TIC, MS File, ES-API, Pos, Scan, Frag: 70, "PosScan"
Spectra averaged over upper half of peaks.
Noise Cutoff: 1000 counts.
Reportable Ion Abundance: > 10%.

Retention Time (MS)	MS Area	Mol. Weight or Ion
3.105	5123971	195.05 I
		154.05 I
4.283	12518521	396.10 I
		395.05 I

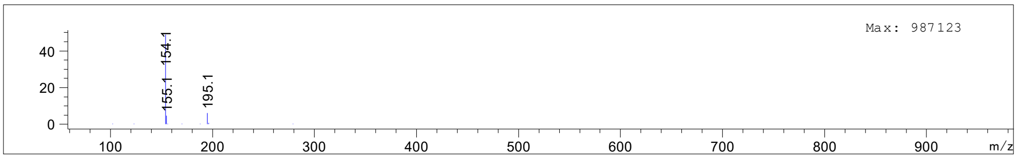

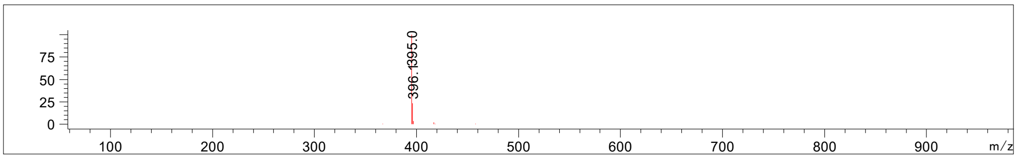

*** End of Report ***

Compound 14

Data File W:\analyti...S_DATA\Natalia_HTS\FGMHTS2_17+14_1.5micro (1) 2021-03-10 06-54-02.D
Sample Name: FGMHTS2_17+14_1.5micro

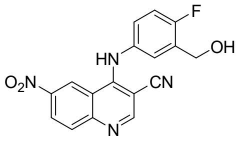

14

===

Acq. Operator : SYSTEM
Sample Operator : SYSTEM
Acq. Instrument : LCMS test   Location : D1F-C4
Injection Date : 3/10/2021 8:22:24 AM   Inj : 1
Inj Volume : 1.000 µl
Different Inj Volume from Sample Entry!   Actual Inj Volume : 1.500 µl
Acq. Method : C:\Users\Public\Documents\ChemStation\1\Methods\0-Std_mth\Gradient\FAST_Nonpolar_General_Method_MS-PosScan-100-1000.M
Last changed : 2/18/2021 11:58:42 AM by SYSTEM
Analysis Method : C:\Users\Public\Documents\ChemStation\1\Methods\DEF_LC.M
Last changed : 9/20/2021 11:46:17 AM by SYSTEM
(modified after loading)
Additional Info : Peak(s) manually integrated

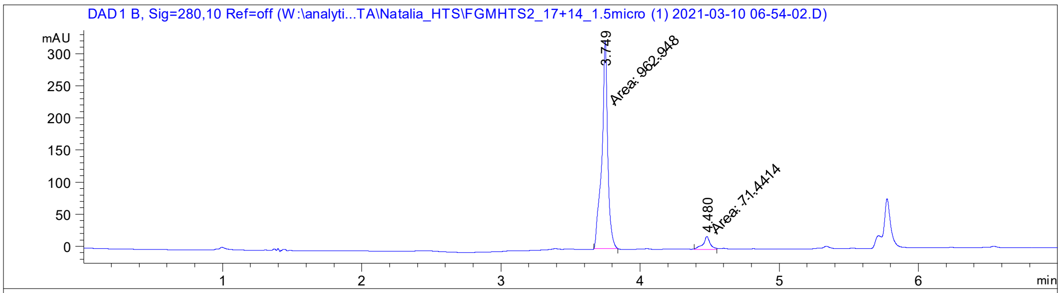

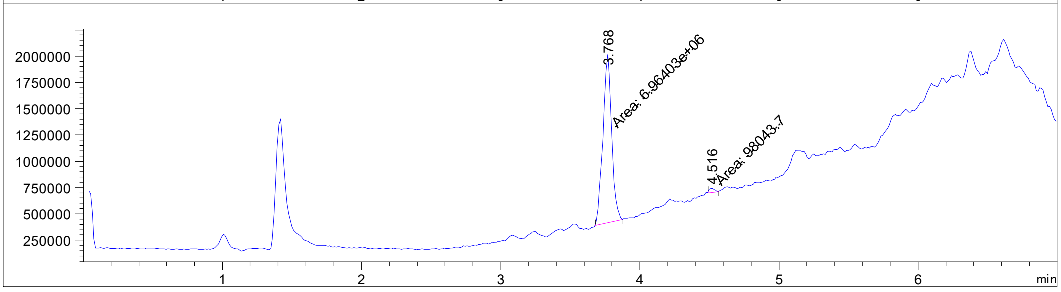

Data File W:\analyti...S_DATA\Natalia_HTS\FGMHTS2_17+14_1.5micro (1) 2021-03-10 06-54-02.D
Sample Name: FGMHTS2_17+14_1.5micro

MS Signal: MSD1 TIC, MS File, ES-API, Pos, Scan, Frag: 70, "PosScan"
Spectra averaged over upper half of peaks.
Noise Cutoff: 1000 counts.
Reportable Ion Abundance: > 10%.

Retention Time (MS)	MS Area	Mol. Weight or Ion
3.768	6964027	340.05 I
		339.05 I
4.516	98044	316.25 I
		298.25 I
		296.20 I
		283.25 I
		282.20 I
		280.25 I
		279.05 I
		268.15 I
		256.20 I
		172.10 I

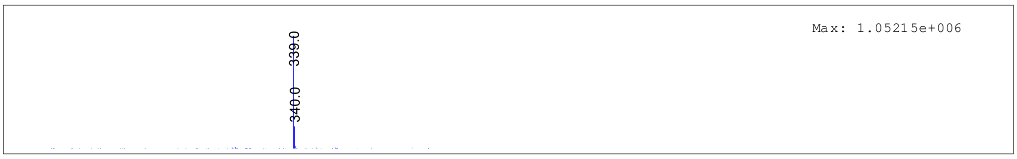

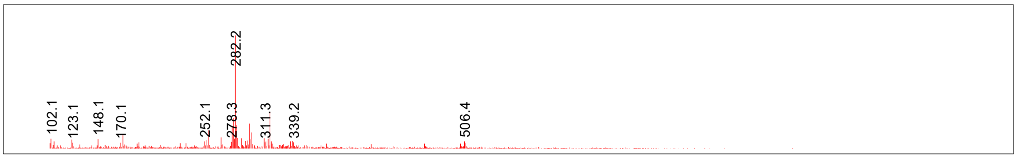

*** End of Report ***

Compound 15

```
Data File W:\analytical_LCMS_DATA\Natalia_HTS\FGHTS2_vial12+17 (1) 2021-03-16 07-51-31.D
Sample Name: FGHTS2_vial12+17

      =================================================================
      Acq. Operator   : SYSTEM
      Sample Operator : SYSTEM
      Acq. Instrument : LCMS test                    Location :   D1B-A7
      Injection Date  : 3/16/2021 10:40:59 AM             Inj :   1
                                                   Inj Volume : 1.000 µl
      Different Inj Volume from Sample Entry!   Actual Inj Volume : 1.500 µl
      Acq. Method     : C:\Users\Public\Documents\ChemStation\1\Methods\0-Std_mth\Gradient\FAST_
                        Nonpolar_General_Method_MS-PosScan-100-1000.M
      Last changed    : 2/18/2021 11:58:42 AM by SYSTEM
      Analysis Method : C:\Users\Public\Documents\ChemStation\1\Methods\DEF_LC.M
      Last changed    : 9/20/2021 3:18:40 PM by SYSTEM
                        (modified after loading)
      Additional Info : Peak(s) manually integrated
```

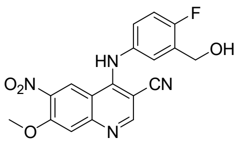

15

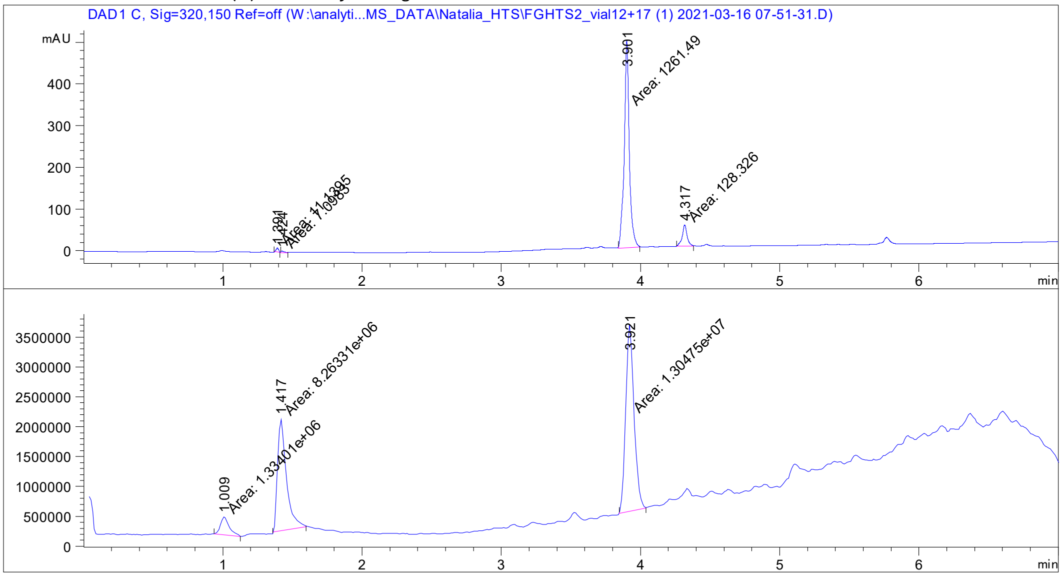

Data File W:\analytical_LCMS_DATA\Natalia_HTS\FGHTS2_vial12+17 (1) 2021-03-16 07-51-31.D
Sample Name: FGHTS2_vial12+17

MS Signal: MSD1 TIC, MS File, ES-API, Pos, Scan, Frag: 70, "PosScan"
Spectra averaged over upper half of peaks.
Noise Cutoff: 1000 counts.
Reportable Ion Abundance: > 10%.

Retention Time (MS)	MS Area	Mol. Weight or Ion
1.009	1334011	282.20 I
		279.10 I
		223.95 I
		200.00 I
		187.00 I
		184.95 I
		183.95 I
		182.95 I
		171.95 I
		163.15 I
		159.00 I
		142.00 I
		138.00 I
		129.00 I
		123.05 I
		112.10 I
		111.10 I
		110.05 I
		102.10 I
1.417	8263314	142.05 I
3.921	13047504	370.05 I
		369.10 I

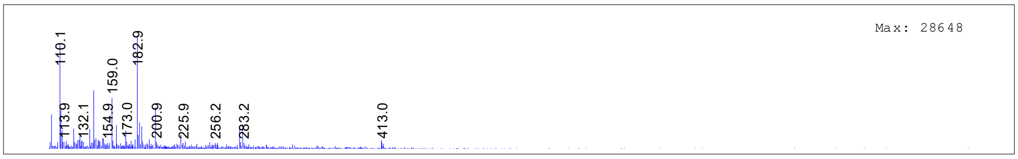

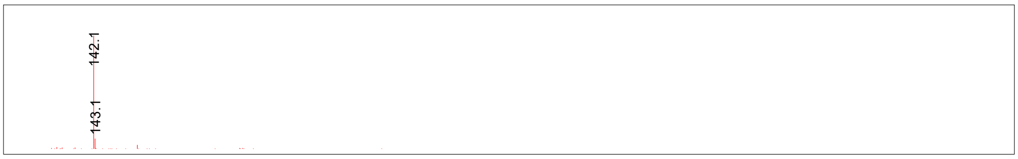

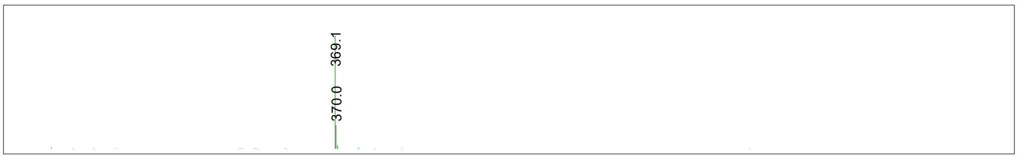

*** End of Report ***

Compound 16

```
Data File W:\analytical_LCMS_DATA\Natalia_HTS\FGMHTS2_17+11 (1) 2021-03-23 08-27-17.D
Sample Name: FGMHTS2_17+11

=====================================================================
Acq. Operator   : SYSTEM
Sample Operator : SYSTEM
Acq. Instrument : LCMS test                    Location :   D1B-B4
Injection Date  : 3/23/2021 9:10:01 AM              Inj :   1
                                             Inj Volume : 1.000 µl
Different Inj Volume from Sample Entry!   Actual Inj Volume : 1.500 µl
Acq. Method     : C:\Users\Public\Documents\ChemStation\1\Methods\0-Std_mth\Gradient\FAST_
                  Nonpolar_General_Method_MS-PosScan-100-1000.M
Last changed    : 2/18/2021 11:58:42 AM by SYSTEM
Analysis Method : C:\Users\Public\Documents\ChemStation\1\Methods\0-Std_mth\Gradient\SLOW_
                  Nonpolar_General_Method_MS-PosScan-100-1000.M
Last changed    : 4/22/2021 2:41:27 PM by SYSTEM
                  (modified after loading)
Method Info     : General method-use for RX monitoring

Additional Info : Peak(s) manually integrated
```

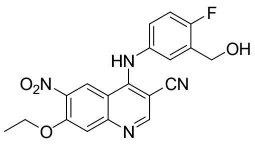

16

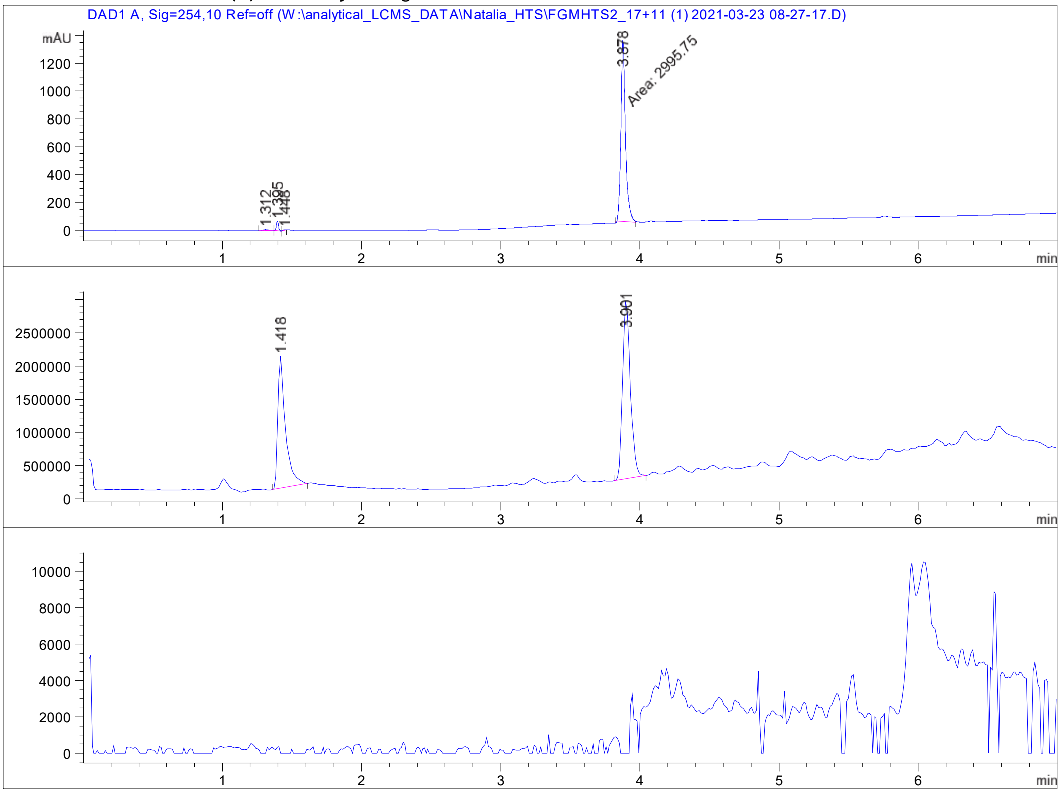

Data File W:\analytical_LCMS_DATA\Natalia_HTS\FGMHTS2_17+11 (1) 2021-03-23 08-27-17.D
Sample Name: FGMHTS2_17+11

MS Signal: MSD1 TIC, MS File, ES-API, Pos, Scan, Frag: 70, "PosScan"
Spectra averaged over upper half of peaks.
Noise Cutoff: 1000 counts.
Reportable Ion Abundance: > 10%.

Retention Time (MS)	MS Area	Mol. Weight or Ion
1.418	7632144	142.10 I
3.901	10477314	384.10 I
		383.15 I

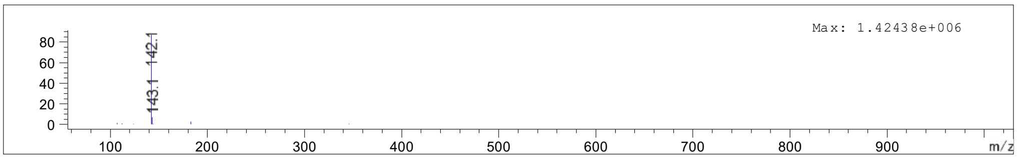

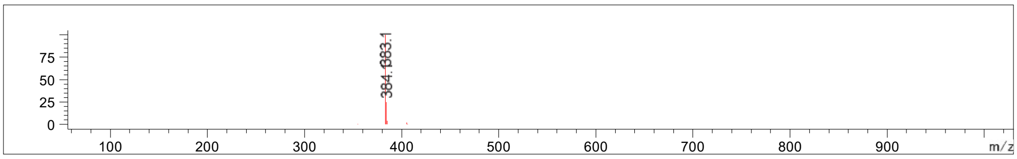

*** End of Report ***

Compound 17

```
Data File W:\analytical_LCMS_DATA\Natalia_HTS\FGHTS2_vial14+18 (1) 2021-03-16 07-50-36.D
Sample Name: FGHTS2_vial14+18

     =====================================================================
     Acq. Operator   : SYSTEM
     Sample Operator : SYSTEM
     Acq. Instrument : LCMS test                       Location :   D1B-A3
     Injection Date  : 3/16/2021 9:10:35 AM                 Inj :   1
                                                     Inj Volume : 1.000 µl
     Different Inj Volume from Sample Entry!   Actual Inj Volume : 1.500 µl
     Acq. Method     : C:\Users\Public\Documents\ChemStation\1\Methods\0-Std_mth\Gradient\FAST_
                       Nonpolar_General_Method_MS-PosScan-100-1000.M
     Last changed    : 2/18/2021 11:58:42 AM by SYSTEM
     Analysis Method : C:\Users\Public\Documents\ChemStation\1\Methods\DEF_LC.M
     Last changed    : 9/20/2021 11:46:17 AM by SYSTEM
                       (modified after loading)
     Additional Info : Peak(s) manually integrated
```

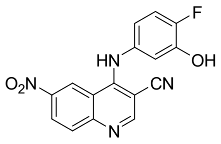

17

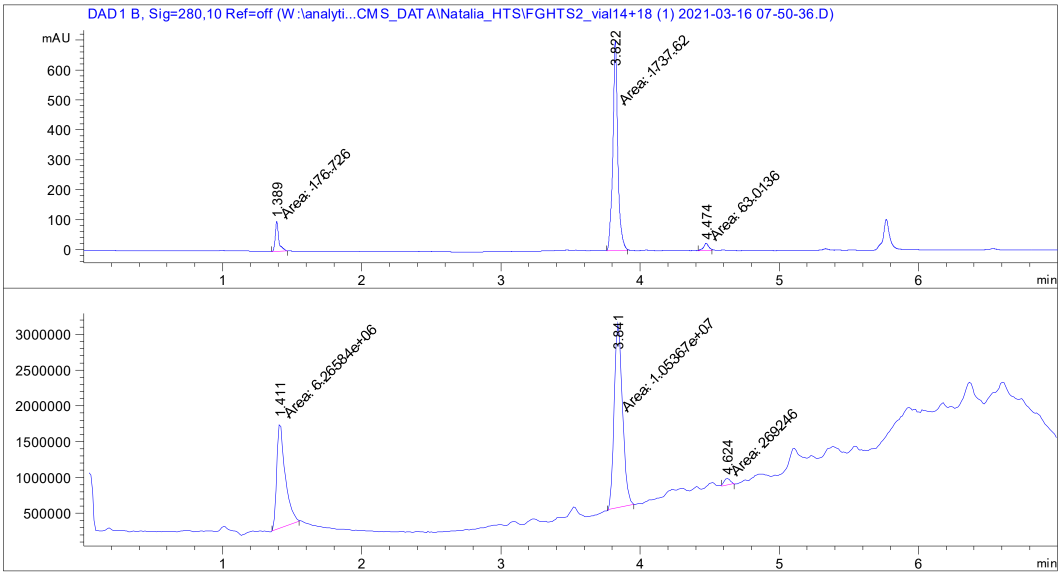

Data File W:\analytical_LCMS_DATA\Natalia_HTS\FGHTS2_vial14+18 (1) 2021-03-16 07-50-36.D
Sample Name: FGHTS2_vial14+18

MS Signal: MSD1 TIC, MS File, ES-API, Pos, Scan, Frag: 70, "PosScan"
Spectra averaged over upper half of peaks.
Noise Cutoff: 1000 counts.
Reportable Ion Abundance: > 10%.

Retention Time (MS)	MS Area	Mol. Weight or Ion
1.411	6265840	128.05 I
3.841	10536701	326.00 I
		325.05 I
4.624	269246	326.25 I
		314.20 I
		312.20 I
		311.15 I
		310.20 I
		298.25 I
		297.25 I
		296.20 I
		295.15 I
		294.25 I
		284.25 I
		283.25 I
		282.25 I
		281.20 I
		280.15 I
		279.10 I
		268.20 I
		256.25 I
		254.15 I
		251.95 I
		239.15 I
		172.15 I
		163.15 I
		148.05 I
		102.20 I

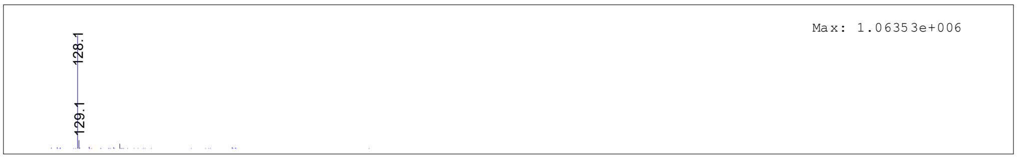

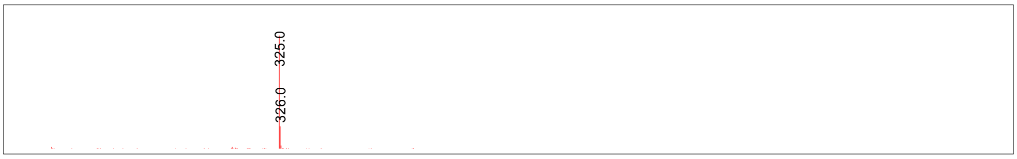

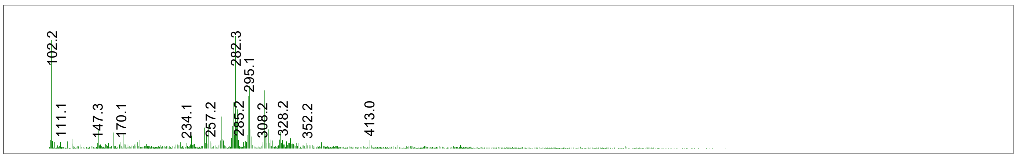

*** End of Report ***

Compound 18

```
Data File W:\analytical_LCMS_DATA\Natalia_HTS\FGHTS2_vial12+18 (1) 2021-03-16 07-51-51.D
Sample Name: FGHTS2_vial12+18

    =================================================================
    Acq. Operator   : SYSTEM
    Sample Operator : SYSTEM
    Acq. Instrument : LCMS test                    Location :   D1B-A8
    Injection Date  : 3/16/2021 11:05:05 AM             Inj :   1
                                                 Inj Volume : 1.000 µl
    Different Inj Volume from Sample Entry!   Actual Inj Volume : 1.500 µl
    Acq. Method     : C:\Users\Public\Documents\ChemStation\1\Methods\0-Std_mth\Gradient\FAST_
                      Nonpolar_General_Method_MS-PosScan-100-1000.M
    Last changed    : 2/18/2021 11:58:42 AM by SYSTEM
    Analysis Method : C:\Users\Public\Documents\ChemStation\1\Methods\DEF_LC.M
    Last changed    : 9/20/2021 3:18:40 PM by SYSTEM
                      (modified after loading)
    Additional Info : Peak(s) manually integrated
```

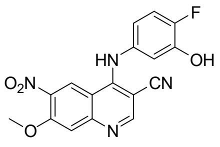

18

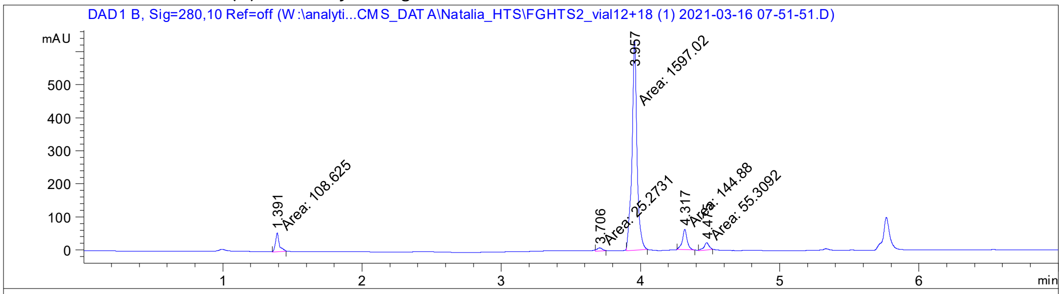

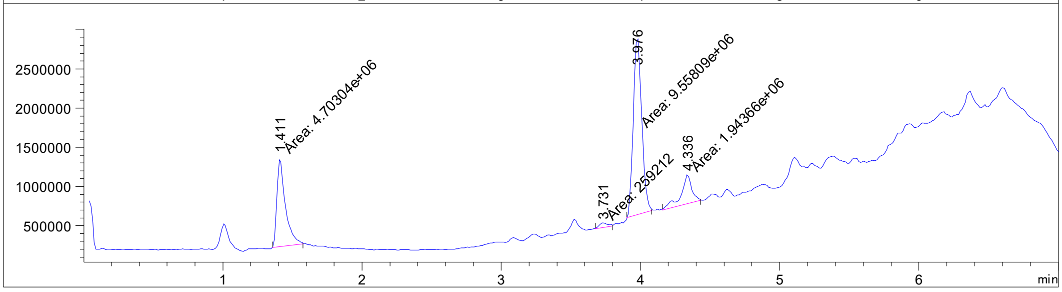

Data File W:\analytical_LCMS_DATA\Natalia_HTS\FGHTS2_vial12+18 (1) 2021-03-16 07-51-51.D
Sample Name: FGHTS2_vial12+18

MS Signal: MSD1 TIC, MS File, ES-API, Pos, Scan, Frag: 70, "PosScan"
Spectra averaged over upper half of peaks.
Noise Cutoff: 1000 counts.
Reportable Ion Abundance: > 10%.

Retention Time (MS)	MS Area	Mol. Weight or Ion
1.411	4703043	128.10 I
3.731	259212	415.10 I
		413.10 I
		375.00 I
		374.10 I
		358.05 I
		310.25 I
		296.25 I
		284.30 I
		283.15 I
		282.25 I
		280.10 I
		279.10 I
		260.05 I
		256.20 I
		244.20 I
		188.10 I
		172.00 I
		163.20 I
		148.00 I
		122.05 I
		102.15 I
3.976	9558088	356.05 I
		355.05 I
4.336	1943662	312.20 I
		296.25 I
		294.20 I
		284.30 I
		282.20 I
		280.20 I
		265.95 I
		265.05 I
		264.00 I
		102.15 I

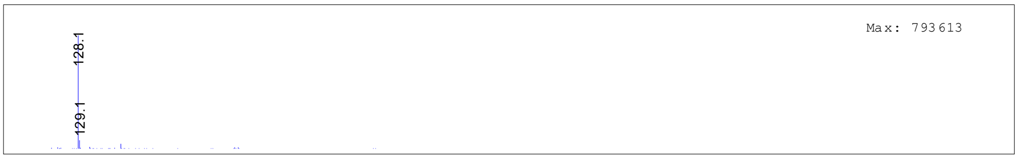

Data File W:\analytical_LCMS_DATA\Natalia_HTS\FGHTS2_vial12+18 (1) 2021-03-16 07-51-51.D
Sample Name: FGHTS2_vial12+18

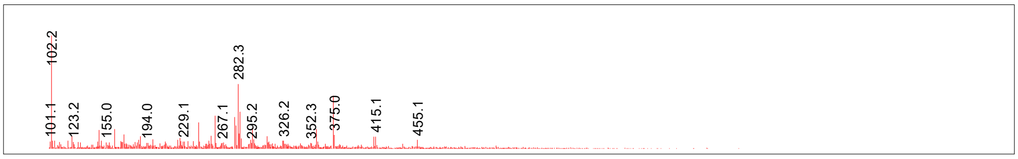

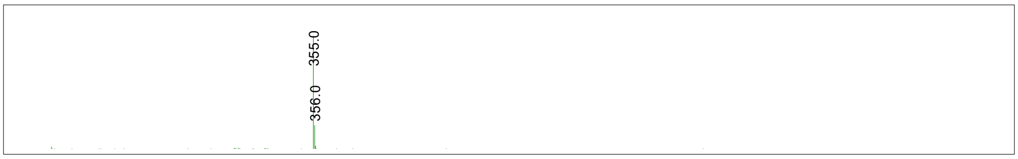

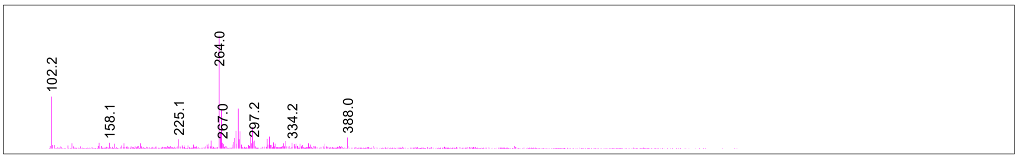

*** End of Report ***

Compound 19

```
Data File W:\analytical_LCMS_DATA\Natalia_HTS\FGMHTS2_18+11 (1) 2021-03-23 08-27-23.D
Sample Name: FGMHTS2_18+11

      =====================================================================
      Acq. Operator   : SYSTEM
      Sample Operator : SYSTEM
      Acq. Instrument : LCMS test                     Location :   D1B-B5
      Injection Date  : 3/23/2021 9:20:28 AM               Inj :   1
                                                    Inj Volume : 1.000 µl
      Different Inj Volume from Sample Entry!   Actual Inj Volume : 1.500 µl
      Acq. Method     : C:\Users\Public\Documents\ChemStation\1\Methods\0-Std_mth\Gradient\FAST_
                        Nonpolar_General_Method_MS-PosScan-100-1000.M
      Last changed    : 2/18/2021 11:58:42 AM by SYSTEM
      Analysis Method : C:\Users\Public\Documents\ChemStation\1\Methods\0-Std_mth\Gradient\SLOW_
                        Nonpolar_General_Method_MS-PosScan-100-1000.M
      Last changed    : 4/22/2021 2:41:27 PM by SYSTEM
                        (modified after loading)
      Method Info     : General method-use for RX monitoring

      Additional Info : Peak(s) manually integrated
```

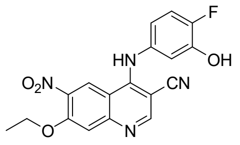

19

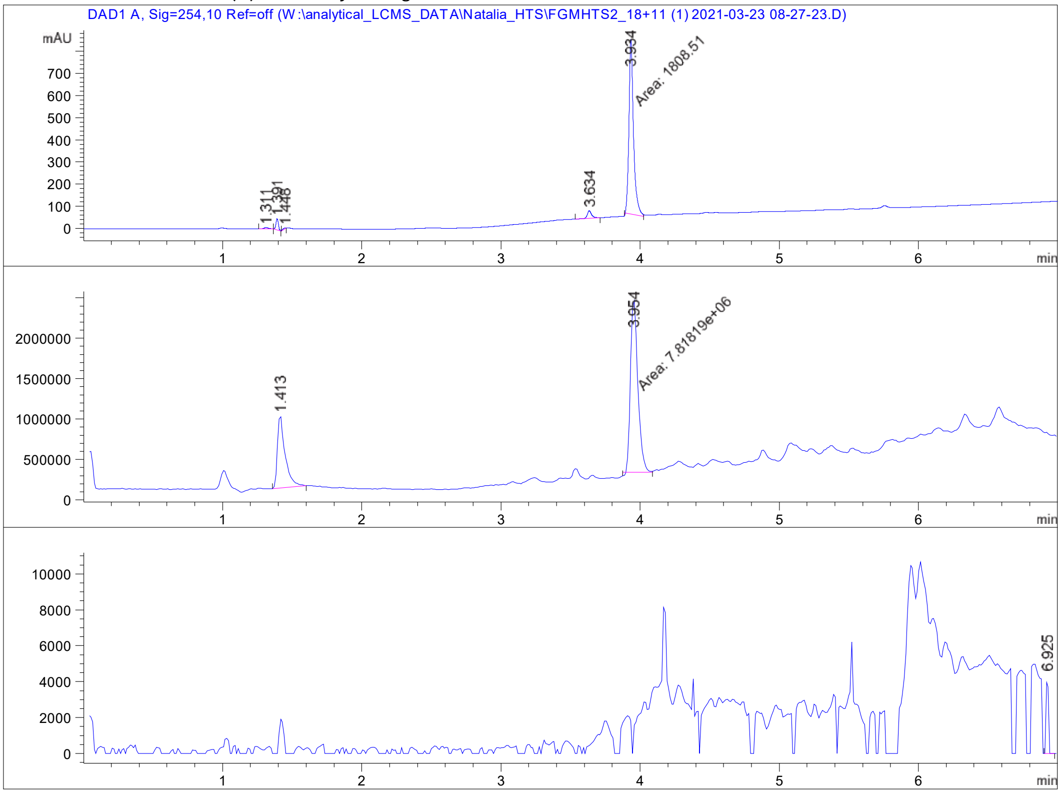

Data File W:\analytical_LCMS_DATA\Natalia_HTS\FGMHTS2_18+11 (1) 2021-03-23 08-27-23.D
Sample Name: FGMHTS2_18+11

MS Signal: MSD1 TIC, MS File, ES-API, Pos, Scan, Frag: 70, "PosScan"
Spectra averaged over upper half of peaks.
Noise Cutoff: 1000 counts.
Reportable Ion Abundance: > 10%.

Retention Time (MS)	MS Area	Mol. Weight or Ion
1.413	3454586	128.10 I
3.954	7818189	370.00 I
		369.05 I

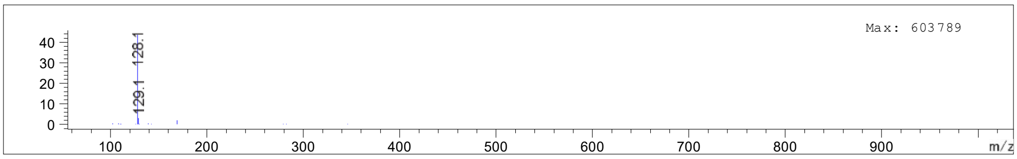

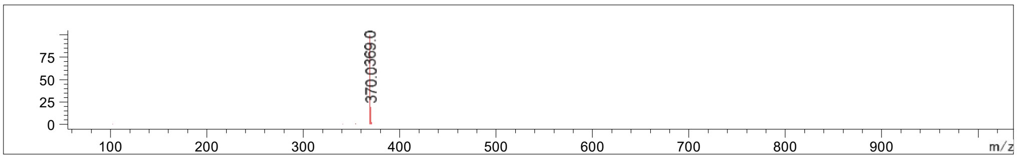

*** End of Report ***

Compound 20

Data File W:\analytical_LCMS_DATA\Natalia_HTS\FGMHTS2_4+14 (1) 2021-03-25 10-38-35.D
Sample Name: FGMHTS2_4+14

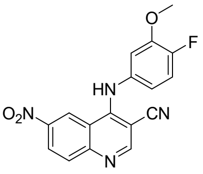

20

===

Acq. Operator : SYSTEM
Sample Operator : SYSTEM
Acq. Instrument : LCMS test Location : D1F-C1
Injection Date : 3/25/2021 11:17:33 AM Inj : 1
Inj Volume : 1.000 µl
Different Inj Volume from Sample Entry! Actual Inj Volume : 1.500 µl
Acq. Method : C:\Users\Public\Documents\ChemStation\1\Methods\0-Std_mth\Gradient\FAST_Nonpolar_General_Method_MS-PosScan-100-1000.M
Last changed : 2/18/2021 11:58:42 AM by SYSTEM
Analysis Method : C:\Users\Public\Documents\ChemStation\1\Methods\0-Std_mth\Gradient\SLOW_Nonpolar_General_Method_MS-PosScan-100-1000.M
Last changed : 4/21/2021 11:43:02 AM by SYSTEM
(modified after loading)
Method Info : General method-use for RX monitoring

Additional Info : Peak(s) manually integrated

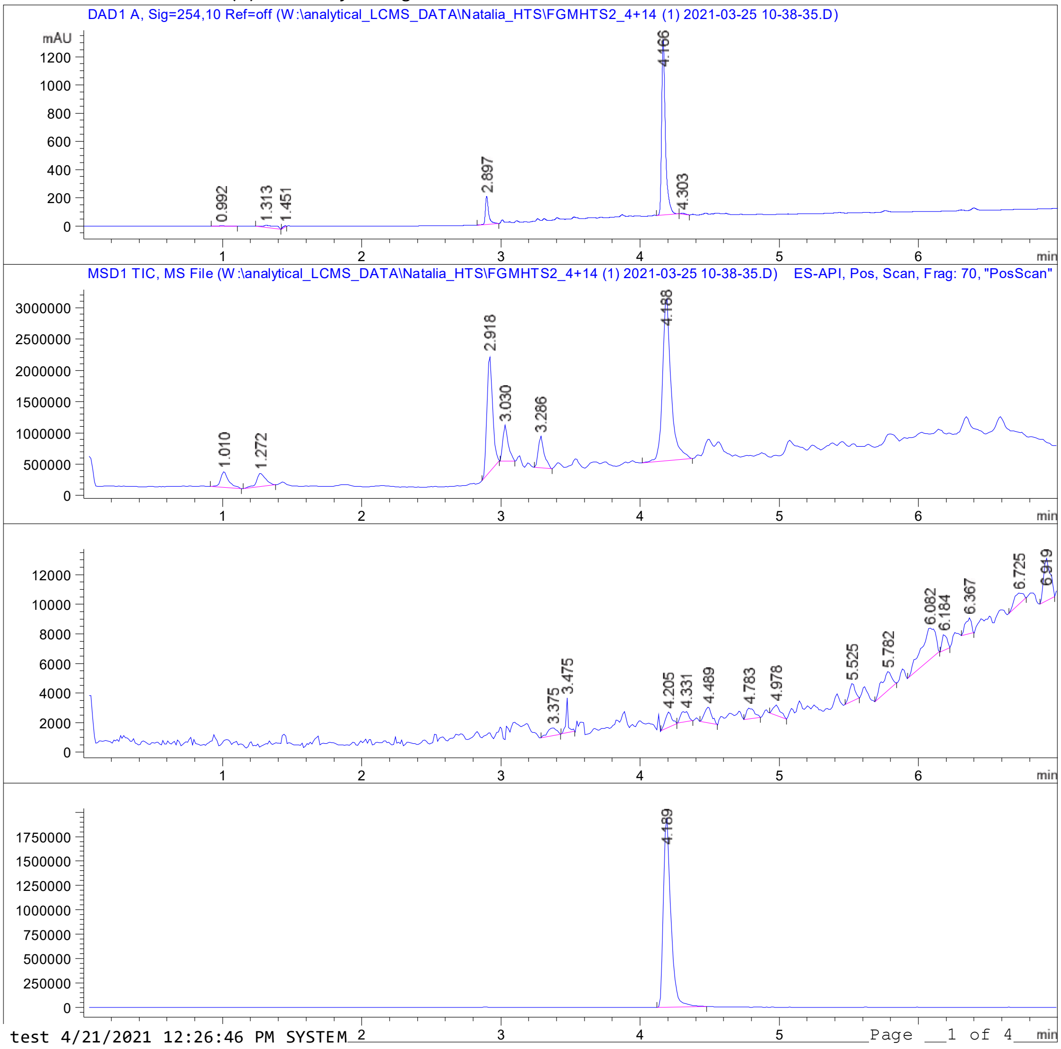

Data File W:\analytical_LCMS_DATA\Natalia_HTS\FGMHTS2_4+14 (1) 2021-03-25 10-38-35.D
Sample Name: FGMHTS2_4+14

MS Signal: MSD1 TIC, MS File, ES-API, Pos, Scan, Frag: 70, "PosScan"
Spectra averaged over upper half of peaks.
Noise Cutoff: 1000 counts.
Reportable Ion Abundance: > 10%.

Retention Time (MS)	MS Area	Mol. Weight or Ion
1.010	1047114	407.10 I
		282.20 I
		278.95 I
		223.95 I
		213.10 I
		199.90 I
		186.90 I
		184.95 I
		183.95 I
		183.00 I
		159.00 I
		141.95 I
		137.95 I
		127.90 I
		122.95 I
		111.95 I
		111.00 I
		110.10 I
		102.15 I
1.272	975491	171.10 I
		169.10 I
		154.05 I
		102.20 I
2.918	5303136	281.10 I
		280.10 I
3.030	1534265	179.10 I
		178.05 I
3.286	1463181	351.10 I
		350.10 I
4.188	11048289	340.05 I
		339.05 I

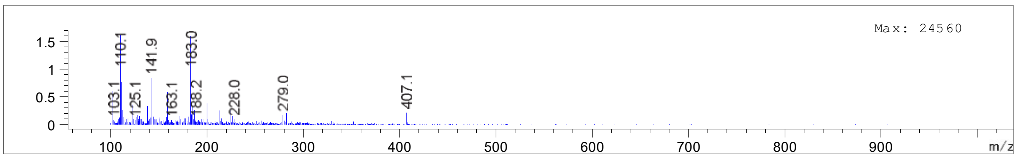

Data File W:\analytical_LCMS_DATA\Natalia_HTS\FGMHTS2_4+14 (1) 2021-03-25 10-38-35.D
Sample Name: FGMHTS2_4+14

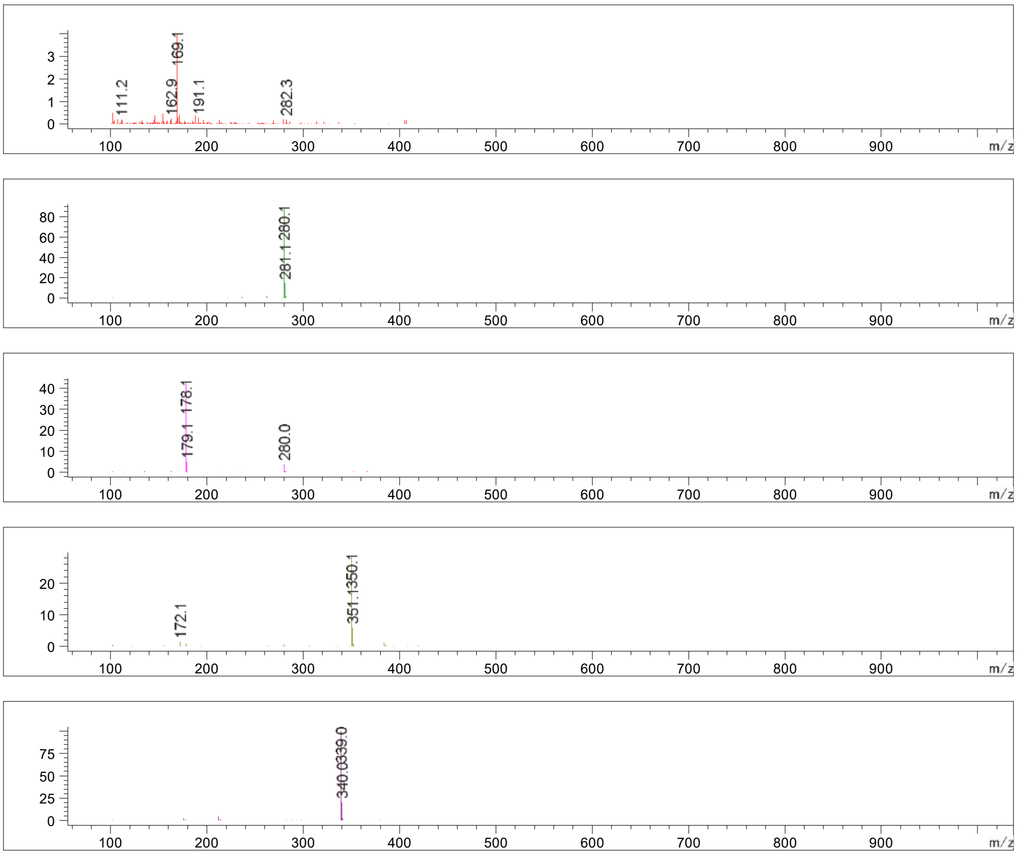

*** End of Report ***

Compound 22

```
Data File W:\analytical_LCMS_DATA\Natalia_HTS\FGHTS 4+11 (1) 2021-03-30 17-52-19.D
Sample Name: FGHTS 4+11

      =====================================================================
      Acq. Operator   : SYSTEM
      Sample Operator : SYSTEM
      Acq. Instrument : LCMS test                       Location :   D1F-A9
      Injection Date  : 3/30/2021 8:07:32 PM                 Inj :   1
                                                      Inj Volume : 1.000 µl
      Different Inj Volume from Sample Entry!   Actual Inj Volume : 3.000 µl
      Acq. Method     : C:\Users\Public\Documents\ChemStation\1\Methods\0-Std_mth\Gradient\FAST_
                        Nonpolar_General_Method_MS-PosScan-100-1000.M
      Last changed    : 2/18/2021 11:58:42 AM by SYSTEM
      Analysis Method : C:\Users\Public\Documents\ChemStation\1\Methods\0-Std_mth\Gradient\SLOW_
                        Nonpolar_General_Method_MS-PosScan-100-1000.M
      Last changed    : 4/21/2021 11:43:02 AM by SYSTEM
                        (modified after loading)
      Method Info     : General method-use for RX monitoring

      Additional Info : Peak(s) manually integrated
```

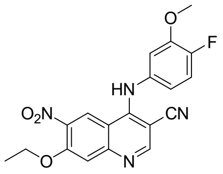

22

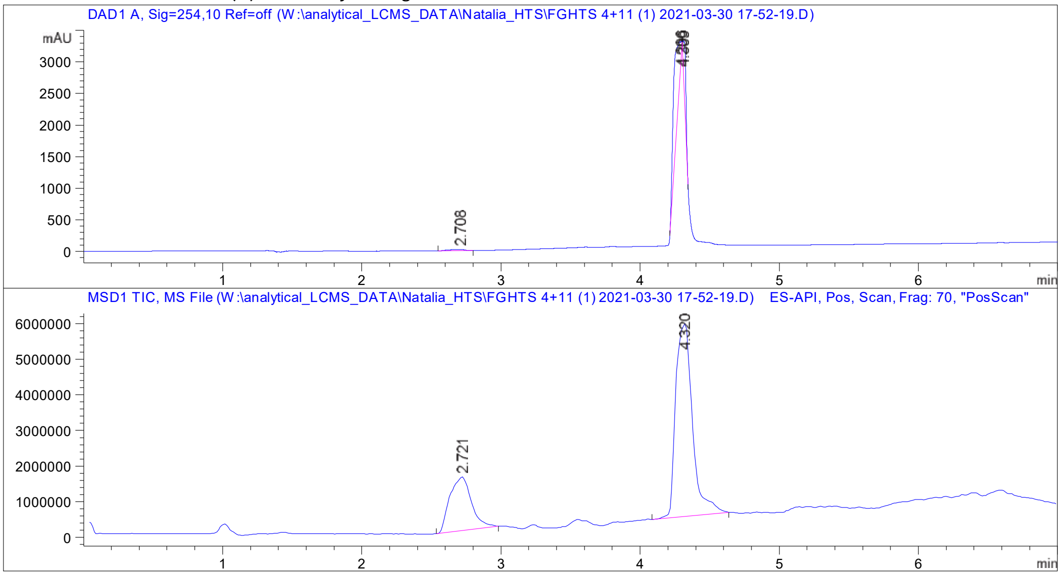

Data File W:\analytical_LCMS_DATA\Natalia_HTS\FGHTS 4+11 (1) 2021-03-30 17-52-19.D
Sample Name: FGHTS 4+11

MS Signal: MSD1 TIC, MS File, ES-API, Pos, Scan, Frag: 70, "PosScan"
Spectra averaged over upper half of peaks.
Noise Cutoff: 1000 counts.
Reportable Ion Abundance: > 10%.

Retention Time (MS)	MS Area	Mol. Weight or Ion
2.721	16338628	142.10 I
4.320	45279924	384.10 I
		383.10 I

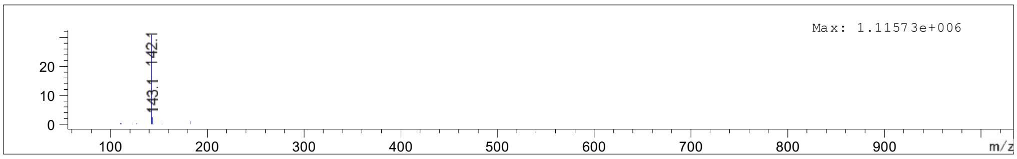

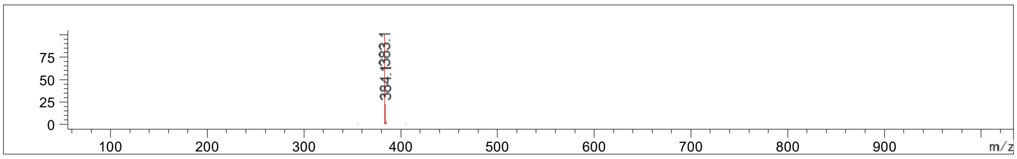

*** End of Report ***

Compound 23

```
Data File W:\analyti...MS_DATA\Natalia_HTS\FGMHTS2_2+14_1.5micro (1) 2021-03-10 06-24-26.D
Sample Name: FGMHTS2_2+14_1.5micro

=====================================================================
Acq. Operator   : SYSTEM
Sample Operator : SYSTEM
Acq. Instrument : LCMS test                    Location :   D1F-B2
Injection Date  : 3/10/2021 6:27:34 AM              Inj :   1
                                             Inj Volume : 1.000 µl
Different Inj Volume from Sample Entry!   Actual Inj Volume : 1.500 µl
Acq. Method     : C:\Users\Public\Documents\ChemStation\1\Methods\0-Std_mth\Gradient\FAST_
                  Nonpolar_General_Method_MS-PosScan-100-1000.M
Last changed    : 2/18/2021 11:58:42 AM by SYSTEM
Analysis Method : C:\Users\Public\Documents\ChemStation\1\Methods\DEF_LC.M
Last changed    : 9/20/2021 11:46:17 AM by SYSTEM
                  (modified after loading)
Additional Info : Peak(s) manually integrated
```

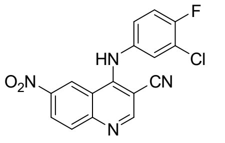

23

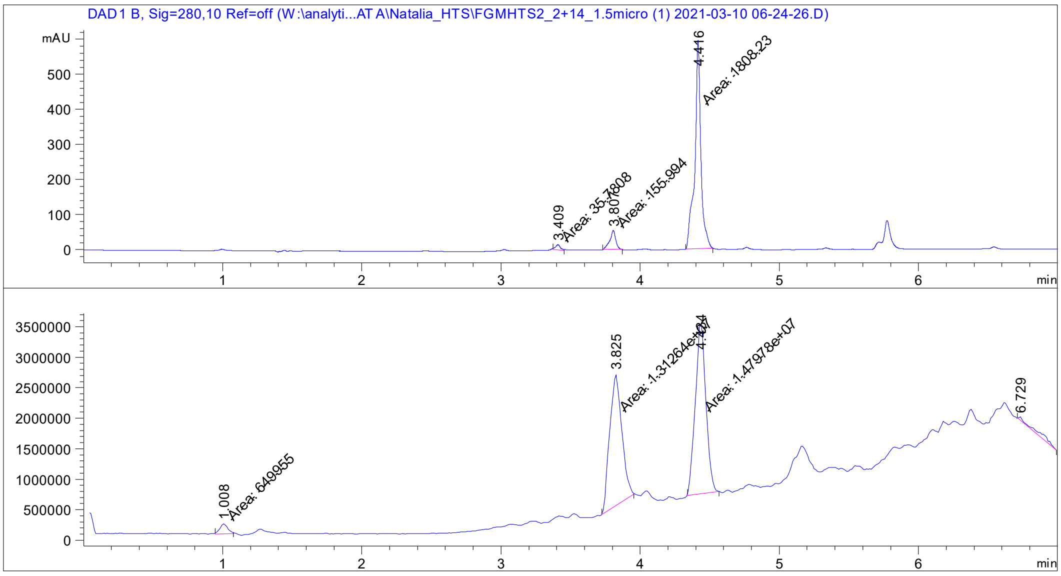

Data File W:\analyti...MS_DATA\Natalia_HTS\FGMHTS2_2+14_1.5micro (1) 2021-03-10 06-24-26.D
Sample Name: FGMHTS2_2+14_1.5micro

MS Signal: MSD1 TIC, MS File, ES-API, Pos, Scan, Frag: 70, "PosScan"
Spectra averaged over upper half of peaks.
Noise Cutoff: 1000 counts.
Reportable Ion Abundance: > 10%.

Retention Time (MS)	MS Area	Mol. Weight or Ion
1.008	649955	282.35 I
		279.05 I
		224.05 I
		199.95 I
		186.85 I
		184.90 I
		182.95 I
		178.00 I
		176.00 I
		172.00 I
		158.95 I
		158.10 I
		156.95 I
		150.15 I
		141.95 I
		137.90 I
		128.95 I
		122.90 I
		112.10 I
		111.00 I
		110.10 I
		109.05 I
		104.10 I
		102.20 I
		100.95 I
3.825	13126363	189.00 I
		187.00 I
		148.00 I
		146.00 I
4.434	14797831	345.00 I
		344.00 I
		343.00 I
6.729	646017	296.25 I
		283.20 I
		282.25 I
		281.20 I
		280.20 I
		256.25 I
		254.20 I
		172.10 I

Data File W:\analyti...MS_DATA\Natalia_HTS\FGMHTS2_2+14_1.5micro (1) 2021-03-10 06-24-26.D
Sample Name: FGMHTS2_2+14_1.5micro

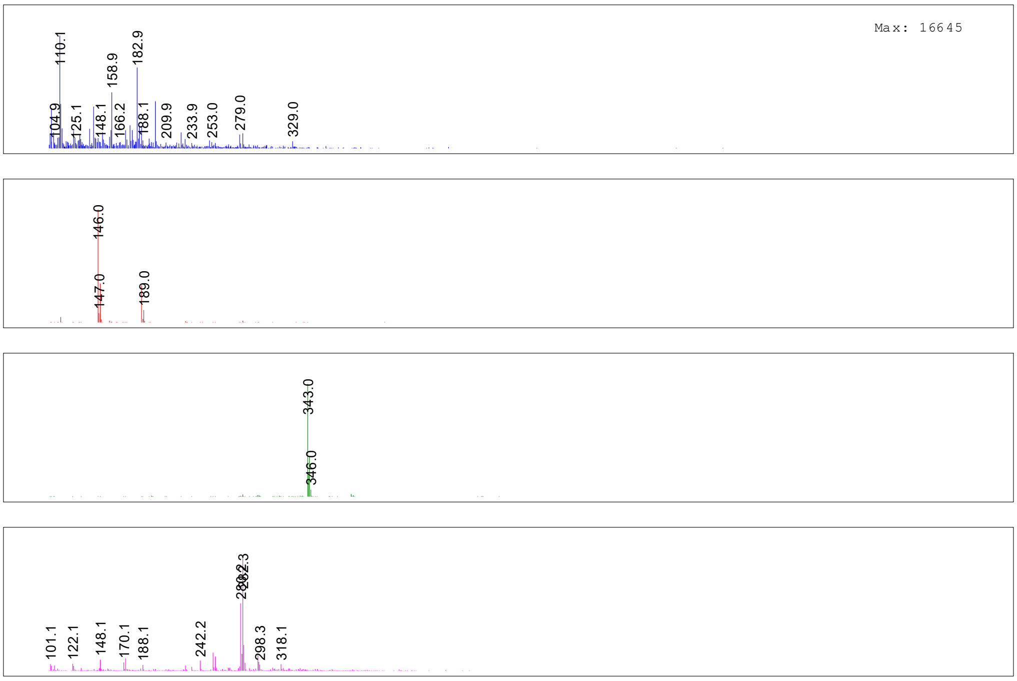

*** End of Report ***

Compound 24

```
Data File W:\analytical_LCMS_DATA\Francesco\FGHTS2_vial2T (1) 2021-03-08 07-04-39.D
Sample Name: FGHTS2_vial2T

    =====================================================================
    Acq. Operator   : SYSTEM
    Sample Operator : SYSTEM
    Acq. Instrument : LCMS test                    Location :  D1F-E1
    Injection Date  : 3/8/2021 7:07:19 AM               Inj :  1
                                                 Inj Volume : 1.000 µl
    Acq. Method     : C:\Users\Public\Documents\ChemStation\1\Methods\0-Std_mth\Gradient\FAST_
                      Nonpolar_General_Method_MS-PosScan-100-1000.M
    Last changed    : 2/18/2021 11:58:42 AM by SYSTEM
    Analysis Method : C:\Users\Public\Documents\ChemStation\1\Methods\DEF_LC.M
    Last changed    : 9/20/2021 3:18:40 PM by SYSTEM
                      (modified after loading)
    Additional Info : Peak(s) manually integrated
```

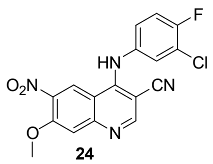

24

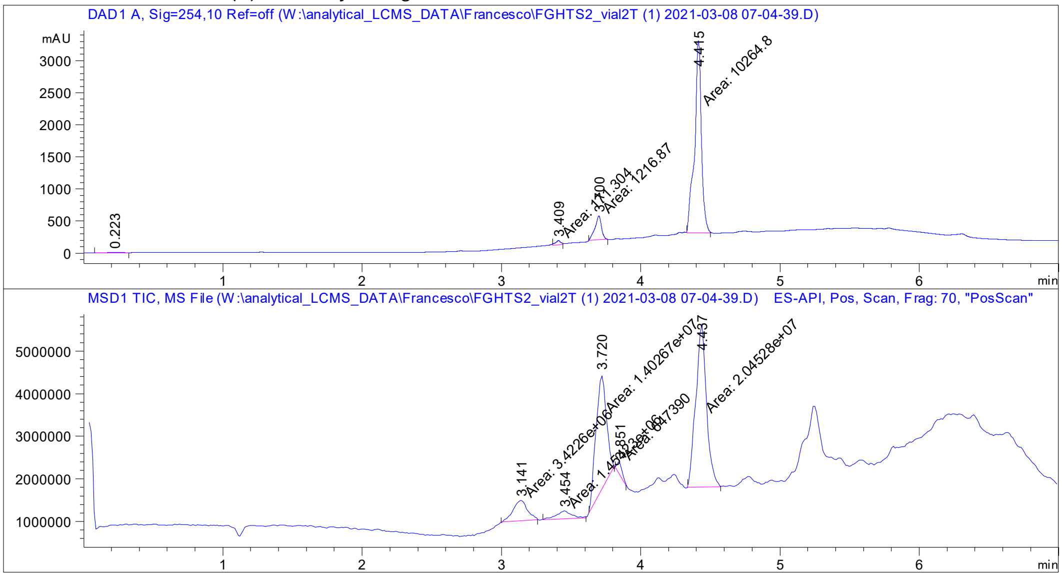

Data File W:\analytical_LCMS_DATA\Francesco\FGHTS2_vial2T (1) 2021-03-08 07-04-39.D
Sample Name: FGHTS2_vial2T

MS Signal: MSD1 TIC, MS File, ES-API, Pos, Scan, Frag: 70, "PosScan"
Spectra averaged over upper half of peaks.
Noise Cutoff: 1000 counts.
Reportable Ion Abundance: > 10%.

Retention Time (MS)	MS Area	Mol. Weight or Ion
3.141	3422600	687.55 I
		665.60 I
		643.55 I
		621.55 I
		599.50 I
		577.45 I
		523.10 I
		508.50 I
		493.75 I
		479.10 I
		464.35 I
		449.85 I
		435.00 I
		276.00 I
		267.10 I
		207.95 I
		207.10 I
		173.10 I
		172.15 I
		171.05 I
		167.00 I
		158.05 I
		154.75 I
		145.05 I
		130.05 I
		126.00 I
		122.40 I
		102.10 I
3.454	1454226	394.10 I
		393.15 I
		345.05 I
		343.05 I
		325.10 I
		307.10 I
		305.05 I
		282.20 I
		279.10 I
		278.05 I
		275.95 I
		246.05 I
		240.00 I
		226.05 I
		224.10 I
		208.00 I
		207.15 I
		198.95 I
		192.15 I

		186.15 I
		185.05 I
		180.05 I
		173.05 I
		172.15 I
		171.00 I
		167.05 I
		163.05 I
		158.00 I
		145.00 I
		130.10 I
		127.95 I
		122.25 I
		102.10 I
3.720	14026717	189.00 I
		187.00 I
		148.00 I
		146.00 I
3.851	647390	198.00 I
		197.00 I
		196.00 I
		187.00 I
		148.00 I
		146.05 I
4.437	20452760	375.05 I
		374.05 I
		373.00 I

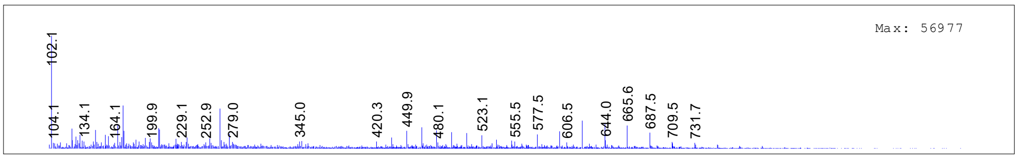

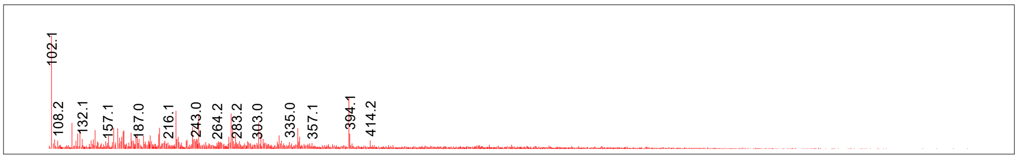

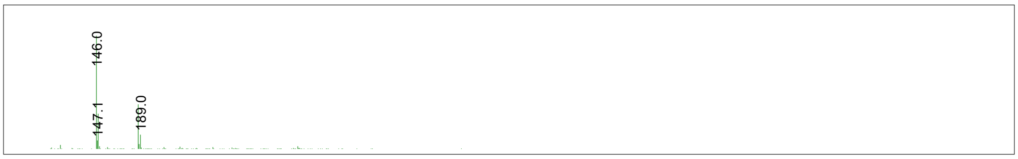

Data File W:\analytical_LCMS_DATA\Francesco\FGHTS2_vial2T (1) 2021-03-08 07-04-39.D
Sample Name: FGHTS2_vial2T

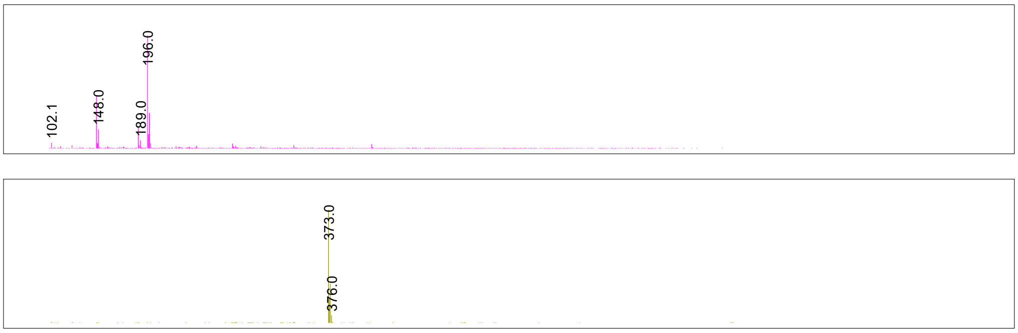

*** End of Report ***

Compound 25

```
Data File W:\analytical_LCMS_DATA\Natalia_HTS\FGMHTS2_2+11 (1) 2021-03-23 07-09-40.D
Sample Name: FGMHTS2_2+11

    =====================================================================
    Acq. Operator    : SYSTEM
    Sample Operator  : SYSTEM
    Acq. Instrument  : LCMS test                       Location :   D1B-A2
    Injection Date   : 3/23/2021 7:22:24 AM                 Inj :   1
                                                    Inj Volume : 1.000 µl
    Different Inj Volume from Sample Entry!   Actual Inj Volume : 1.500 µl
    Acq. Method      : C:\Users\Public\Documents\ChemStation\1\Methods\0-Std_mth\Gradient\FAST_
                       Nonpolar_General_Method_MS-PosScan-100-1000.M
    Last changed     : 2/18/2021 11:58:42 AM by SYSTEM
    Analysis Method  : C:\Users\Public\Documents\ChemStation\1\Methods\0-Std_mth\Gradient\SLOW_
                       Nonpolar_General_Method_MS-PosScan-100-1000.M
    Last changed     : 4/22/2021 3:11:23 PM by SYSTEM
                       (modified after loading)
    Method Info      : General method-use for RX monitoring

    Additional Info : Peak(s) manually integrated
```

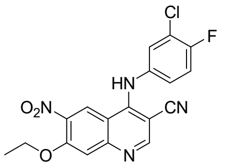

25

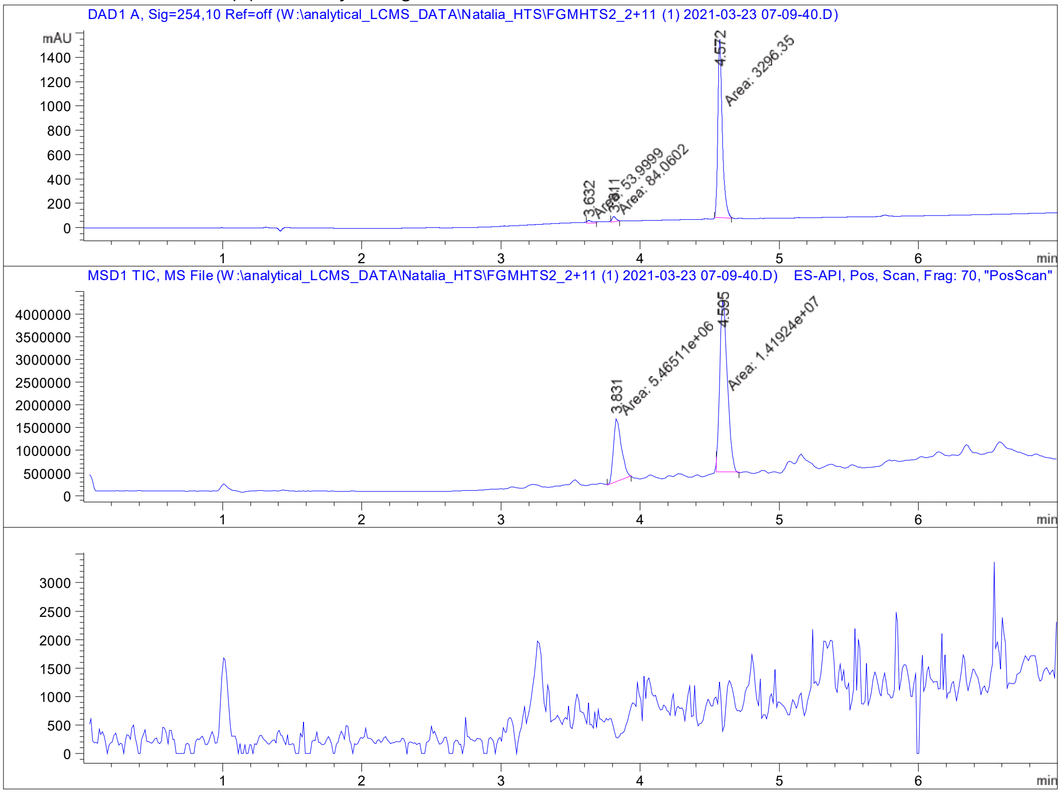

Data File W:\analytical_LCMS_DATA\Natalia_HTS\FGMHTS2_2+11 (1) 2021-03-23 07-09-40.D
Sample Name: FGMHTS2_2+11

MS Signal: MSD1 TIC, MS File, ES-API, Pos, Scan, Frag: 70, "PosScan"
Spectra averaged over upper half of peaks.
Noise Cutoff: 1000 counts.
Reportable Ion Abundance: > 10%.

Retention Time (MS)	MS Area	Mol. Weight or Ion
3.831	5465109	187.05 I
		148.00 I
		146.00 I
4.595	14192433	389.05 I
		388.00 I
		387.00 I

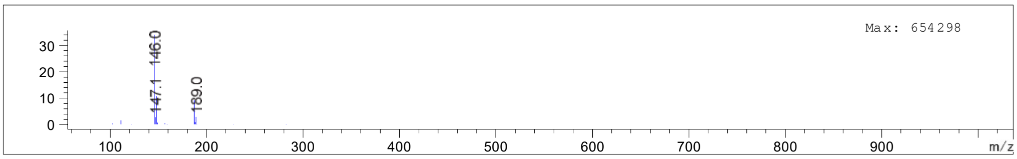

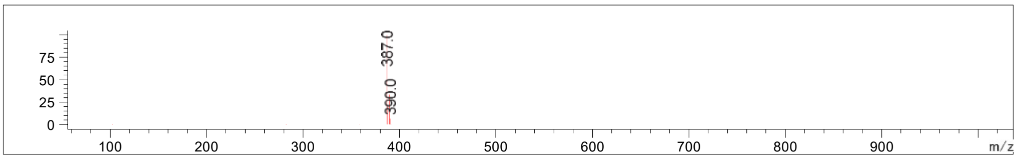

*** End of Report ***

Compound 26

```
Data File W:\analytical_LCMS_DATA\Natalia_HTS\FGMHTS2_1+14 (1) 2021-03-10 06-11-30.D
Sample Name: FGMHTS2_1+14

    =====================================================================
    Acq. Operator   : SYSTEM
    Sample Operator : SYSTEM
    Acq. Instrument : LCMS test                        Location :   D1F-B1
    Injection Date  : 3/10/2021 6:14:14 AM                  Inj :   1
                                                     Inj Volume : 1.000 µl
    Acq. Method     : C:\Users\Public\Documents\ChemStation\1\Methods\0-Std_mth\Gradient\FAST_
                      Nonpolar_General_Method_MS-PosScan-100-1000.M
    Last changed    : 2/18/2021 11:58:42 AM by SYSTEM
    Analysis Method : C:\Users\Public\Documents\ChemStation\1\Methods\DEF_LC.M
    Last changed    : 9/20/2021 11:46:17 AM by SYSTEM
                      (modified after loading)
    Additional Info : Peak(s) manually integrated
```

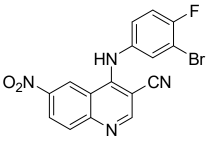

26

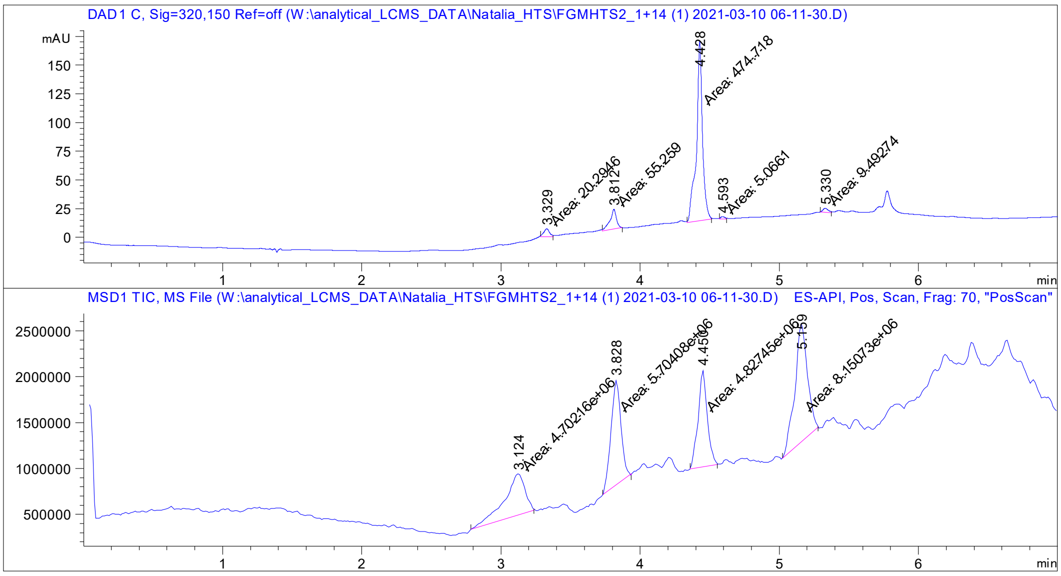

Data File W:\analytical_LCMS_DATA\Natalia_HTS\FGMHTS2_1+14 (1) 2021-03-10 06-11-30.D
Sample Name: FGMHTS2_1+14

MS Signal: MSD1 TIC, MS File, ES-API, Pos, Scan, Frag: 70, "PosScan"
Spectra averaged over upper half of peaks.
Noise Cutoff: 1000 counts.
Reportable Ion Abundance: > 10%.

Retention Time (MS)	MS Area	Mol. Weight or Ion
3.124	4702164	687.65 I
		665.60 I
		643.55 I
		621.55 I
		599.50 I
		577.55 I
		555.55 I
		508.35 I
		479.10 I
		464.30 I
		449.75 I
		267.05 I
		198.95 I
		172.10 I
		171.05 I
		167.00 I
		158.05 I
		130.05 I
		122.40 I
		102.15 I
3.828	5704076	232.95 I
		230.95 I
		191.90 I
		189.95 I
4.450	4827448	389.95 I
		388.95 I
		387.95 I
		386.90 I
5.159	8150725	327.35 I
		326.30 I
		280.20 I

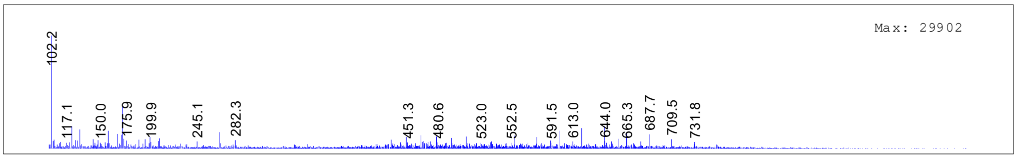

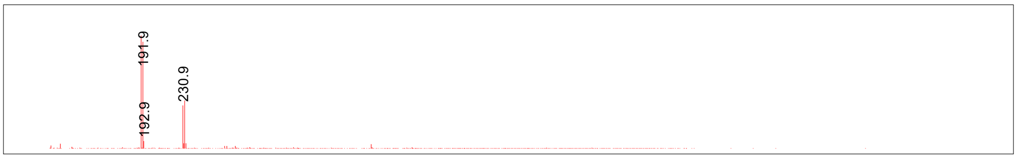

Data File W:\analytical_LCMS_DATA\Natalia_HTS\FGMHTS2_1+14 (1) 2021-03-10 06-11-30.D
Sample Name: FGMHTS2_1+14

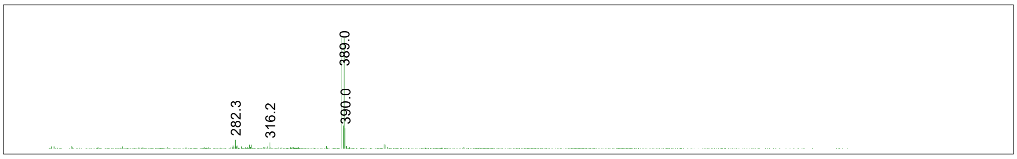

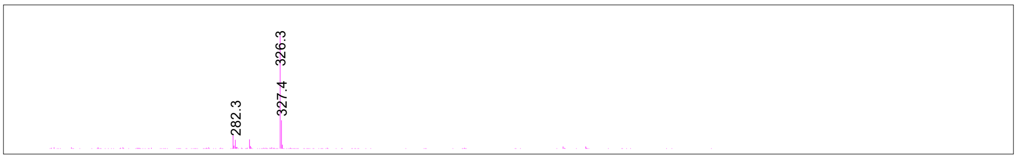

*** End of Report ***

Compound 27

Sample Report

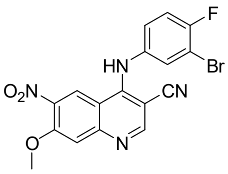

27

Column Information

**Column name:**	Poroshell 120 EC-C18, 1000 bar	**Length:**	150.0 mm
**Serial #:**	USJYZ02256	**Void Time:**	min
**Diameter:**	3.00 mm		
**Dead volume:**	0.640 ml		

**Data file:**	W:\analytical_LCMS_DATA\Natalia_HTS\FGHTS2_vial1 (1) 2021-03-09 07-32-02.D		
**Sample name:**	FGHTS2_vial1		
**Description:**			
**Sample amount:**	0.000	**Sample type:**	Sample
**Instrument:**	LCMS test	**Location:**	D1F-A1
**Injection date:**	2021-03-09 08:32:08+01:00	**Injection:**	1 of 1
		**Injection volume:**	0.700
**Acq. method:**	FAST_Nonpolar_General_Method_MS-PosScan-100-1000.M		
**Analysis method:**	DEF_LC.M		

Module	Type	Part. No.	Serial No.	Firmware
Agilent G6125B MSD	Detector	G6125B	<undef>	3.02.50
Quat. Pump	Pump	G7104C	DEAGZ01778	B.07.30 [0005]
Column Comp.	Column compartment	G7116A	DEAEM06531	D.07.30 [0005]
DAD	Detector	G7117C	DEAEK07274	D.07.30 [0005]
Multisampler	Auto sampler	G7167A	DEAGY01736	D.07.32 [0001]

Results

Sample Name FGHTS2_vial1

Chromatograms

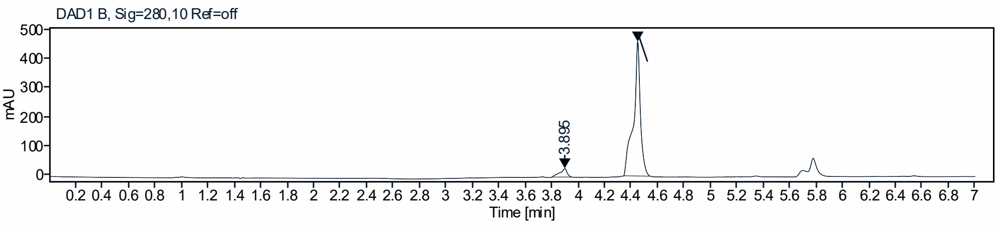

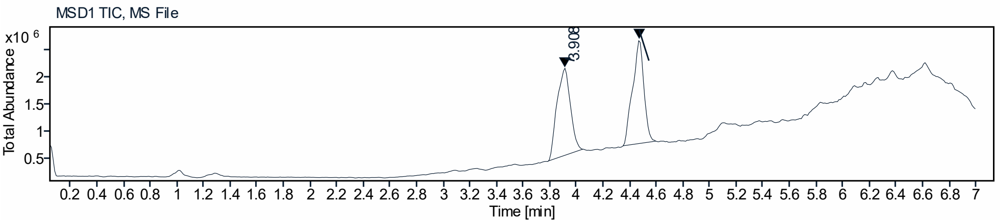

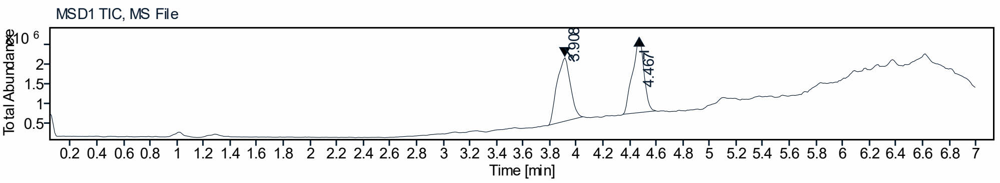

Sample Purity

Signal Description DAD1 B, Sig=280,10 Ref=off

Sample Name	Name	RT	Width	Area	Area%	Height
FGHTS2_vial1		3.895	0.055	110.5527	6.19	29.3784
FGHTS2_vial1		4.446	0.038	1674.9293	93.81	461.1955

Max Area% 93.808

UV Signal Purity>95% Fail

UV Apex Spectra

GOETHE UNIVERSITÄT FRANKFURT AM MAIN

RT:

3.895

Sample Name:

FGHTS2_vial1

Signal Name:

DAD1B

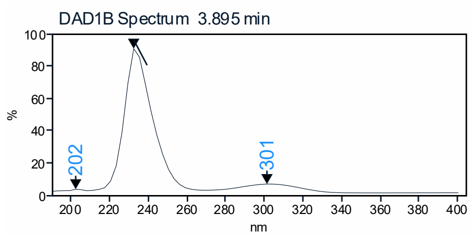

RT:

4.446

Sample Name:

FGHTS2_vial1

Signal Name:

DAD1B

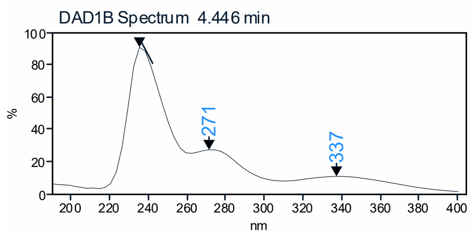

MSD Apex Spectra

RT:

3.908

Sample Name:

FGHTS2_vial1

Signal Name:

MSD1TIC

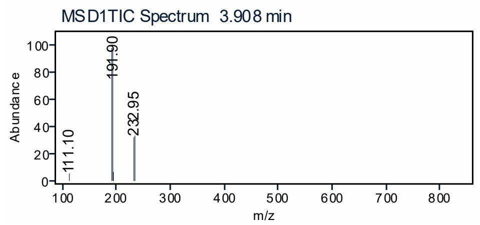

RT:

4.467

Sample Name:

FGHTS2_vial1

Signal Name:

MSD1TIC

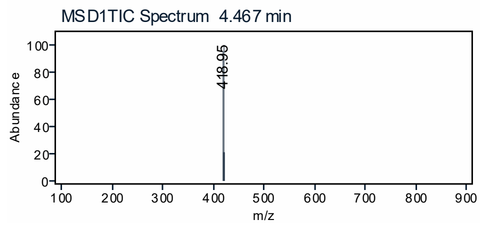

Compound 28

Data File W:\analytical_LCMS_DATA\Natalia_HTS\FGMHTS2_1+11 (1) 2021-03-23 07-09-15.D
Sample Name: FGMHTS2_1+11

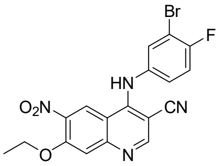

28

```
=====================================================================
Acq. Operator   : SYSTEM
Sample Operator : SYSTEM
Acq. Instrument : LCMS test                    Location :   D1B-A1
Injection Date  : 3/23/2021 7:11:59 AM              Inj :   1
                                             Inj Volume : 1.000 µl
Different Inj Volume from Sample Entry!   Actual Inj Volume : 1.500 µl
Acq. Method     : C:\Users\Public\Documents\ChemStation\1\Methods\0-Std_mth\Gradient\FAST_
                  Nonpolar_General_Method_MS-PosScan-100-1000.M
Last changed    : 2/18/2021 11:58:42 AM by SYSTEM
Analysis Method : C:\Users\Public\Documents\ChemStation\1\Methods\0-Std_mth\Gradient\SLOW_
                  Nonpolar_General_Method_MS-PosScan-100-1000.M
Last changed    : 4/22/2021 3:11:23 PM by SYSTEM
                  (modified after loading)
Method Info     : General method-use for RX monitoring

Additional Info : Peak(s) manually integrated
```

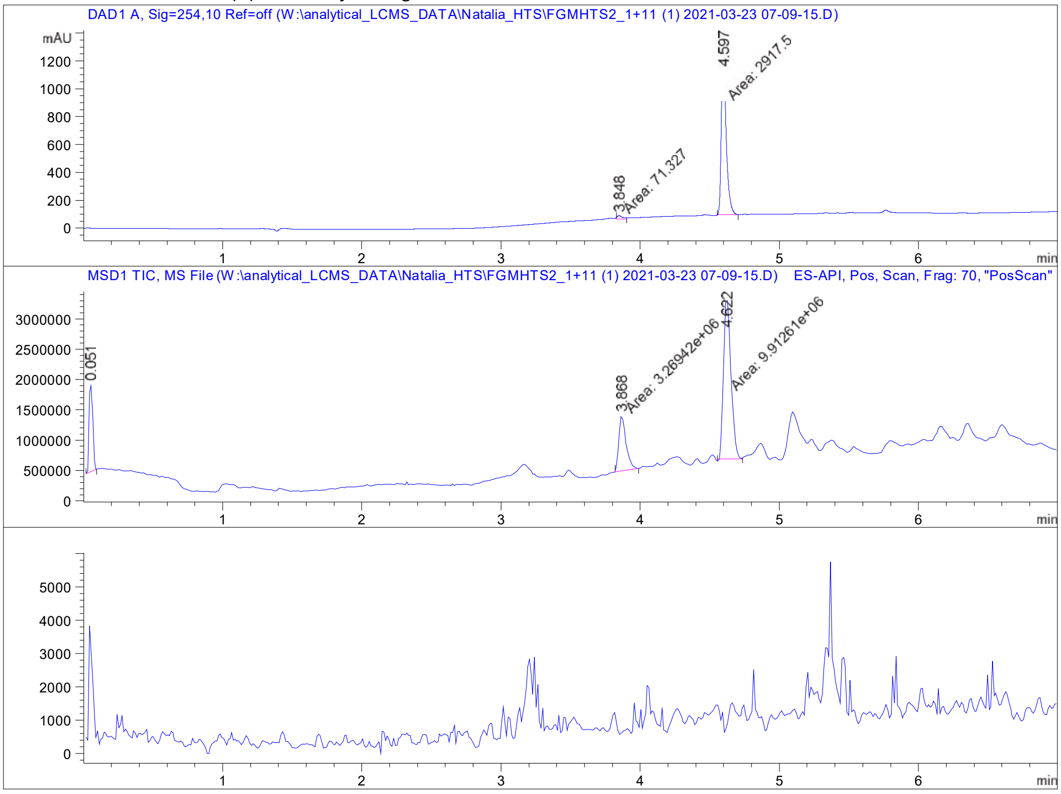

Data File W:\analytical_LCMS_DATA\Natalia_HTS\FGMHTS2_1+11 (1) 2021-03-23 07-09-15.D
Sample Name: FGMHTS2_1+11

MS Signal: MSD1 TIC, MS File, ES-API, Pos, Scan, Frag: 70, "PosScan"
Spectra averaged over upper half of peaks.
Noise Cutoff: 1000 counts.
Reportable Ion Abundance: > 10%.

Retention Time (MS)	MS Area	Mol. Weight or Ion
0.051	2730536	198.90 I
		198.00 I
		185.00 I
		176.15 I
		175.95 I
		175.80 I
		171.05 I
		170.90 I
		167.10 I
		166.90 I
		166.60 I
		158.10 I
		157.90 I
		157.75 I
		143.20 I
		143.00 I
		130.10 I
		129.95 I
		129.80 I
		122.65 I
		122.50 I
		120.65 I
		120.40 I
		109.95 I
		102.30 I
		102.10 I
		101.95 I
		101.45 I
3.868	3269422	232.95 I
		230.95 I
		191.95 I
		189.95 I
4.622	9912608	433.95 I
		432.95 I
		432.05 I
		431.00 I

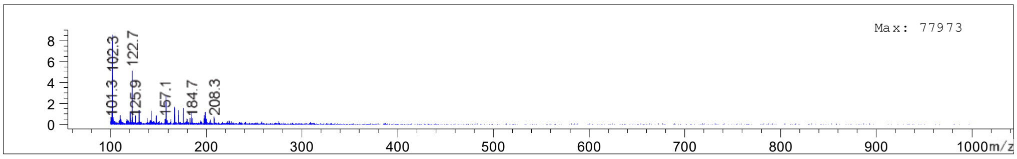

Data File W:\analytical_LCMS_DATA\Natalia_HTS\FGMHTS2_1+11 (1) 2021-03-23 07-09-15.D
Sample Name: FGMHTS2_1+11

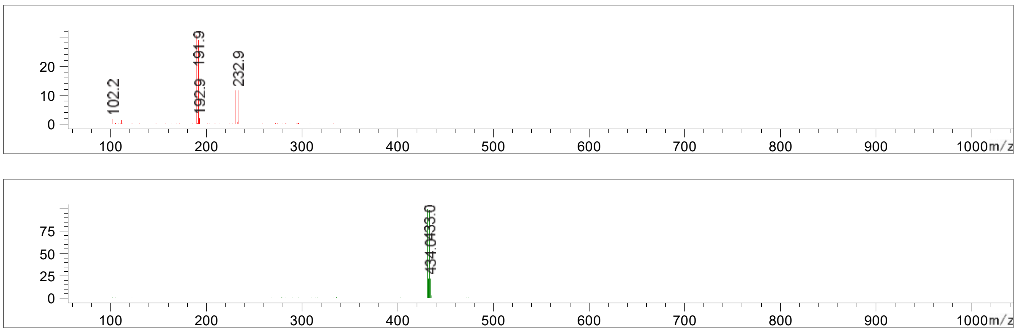

*** End of Report ***

Compound 29

```
Data File W:\analytical_LCMS_DATA\Natalia_HTS\FGHTS2_vial14+19 (1) 2021-03-16 07-50-47.D
Sample Name: FGHTS2_vial14+19

    =====================================================================
    Acq. Operator   : SYSTEM
    Sample Operator : SYSTEM
    Acq. Instrument : LCMS test                    Location :   D1B-A4
    Injection Date  : 3/16/2021 9:34:42 AM              Inj :   1
                                                 Inj Volume : 1.000 µl
    Different Inj Volume from Sample Entry!   Actual Inj Volume : 1.500 µl
    Acq. Method     : C:\Users\Public\Documents\ChemStation\1\Methods\0-Std_mth\Gradient\FAST_
                      Nonpolar_General_Method_MS-PosScan-100-1000.M
    Last changed    : 2/18/2021 11:58:42 AM by SYSTEM
    Analysis Method : C:\Users\Public\Documents\ChemStation\1\Methods\DEF_LC.M
    Last changed    : 9/20/2021 11:46:17 AM by SYSTEM
                      (modified after loading)
    Additional Info : Peak(s) manually integrated
```

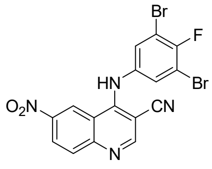

29

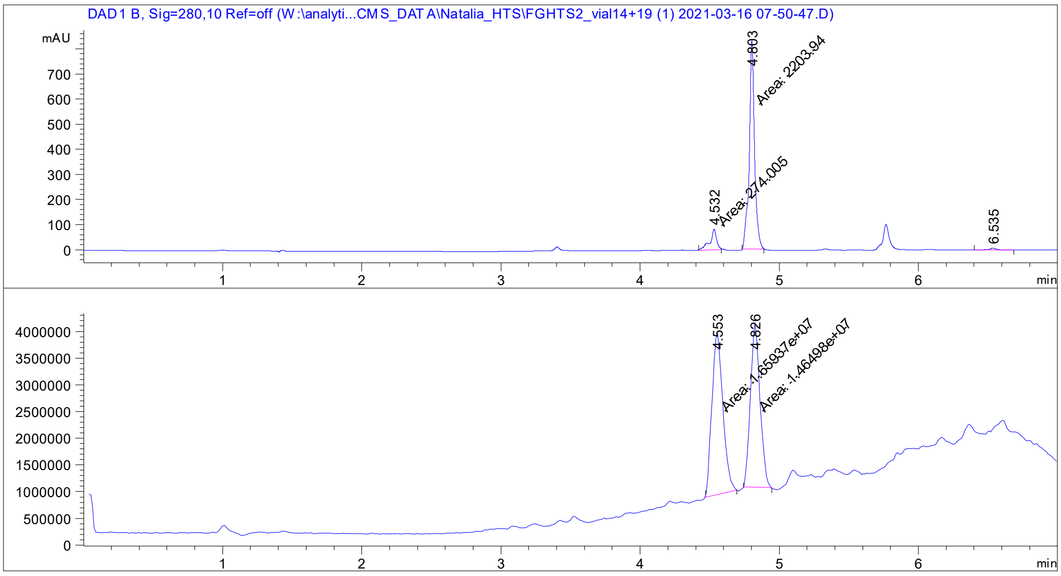

Data File W:\analytical_LCMS_DATA\Natalia_HTS\FGHTS2_vial14+19 (1) 2021-03-16 07-50-47.D
Sample Name: FGHTS2_vial14+19

MS Signal: MSD1 TIC, MS File, ES-API, Pos, Scan, Frag: 70, "PosScan"
Spectra averaged over upper half of peaks.
Noise Cutoff: 1000 counts.
Reportable Ion Abundance: > 10%.

Retention Time (MS)	MS Area	Mol. Weight or Ion
4.553	16593693	312.85 I
		310.85 I
		308.85 I
		271.85 I
		269.85 I
		267.80 I
4.826	14649811	468.90 I
		467.90 I
		466.95 I
		465.90 I
		464.90 I

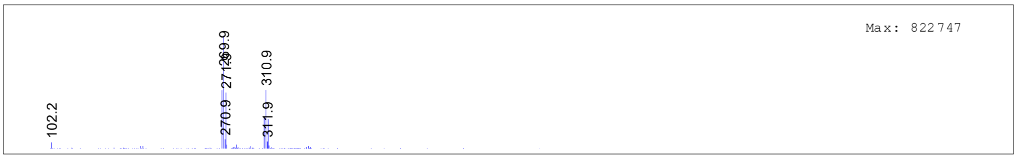

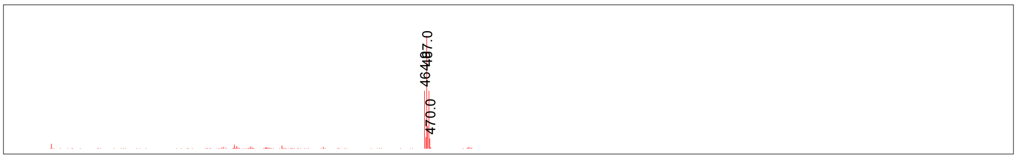

*** End of Report ***

Compound 30

```
Data File W:\analytical_LCMS_DATA\Natalia_HTS\FGHTS2_vial12+19 (1) 2021-03-16 07-52-01.D
Sample Name: FGHTS2_vial12+19

    =====================================================================
    Acq. Operator   : SYSTEM
    Sample Operator : SYSTEM
    Acq. Instrument : LCMS test                      Location :   D1B-A9
    Injection Date  : 3/16/2021 11:29:11 AM               Inj :   1
                                                   Inj Volume : 1.000 µl
    Different Inj Volume from Sample Entry!   Actual Inj Volume : 1.500 µl
    Acq. Method     : C:\Users\Public\Documents\ChemStation\1\Methods\0-Std_mth\Gradient\FAST_
                      Nonpolar_General_Method_MS-PosScan-100-1000.M
    Last changed    : 2/18/2021 11:58:42 AM by SYSTEM
    Analysis Method : C:\Users\Public\Documents\ChemStation\1\Methods\DEF_LC.M
    Last changed    : 9/20/2021 3:18:40 PM by SYSTEM
                      (modified after loading)
    Additional Info : Peak(s) manually integrated
```

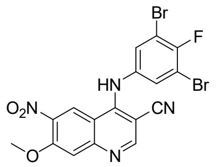

30

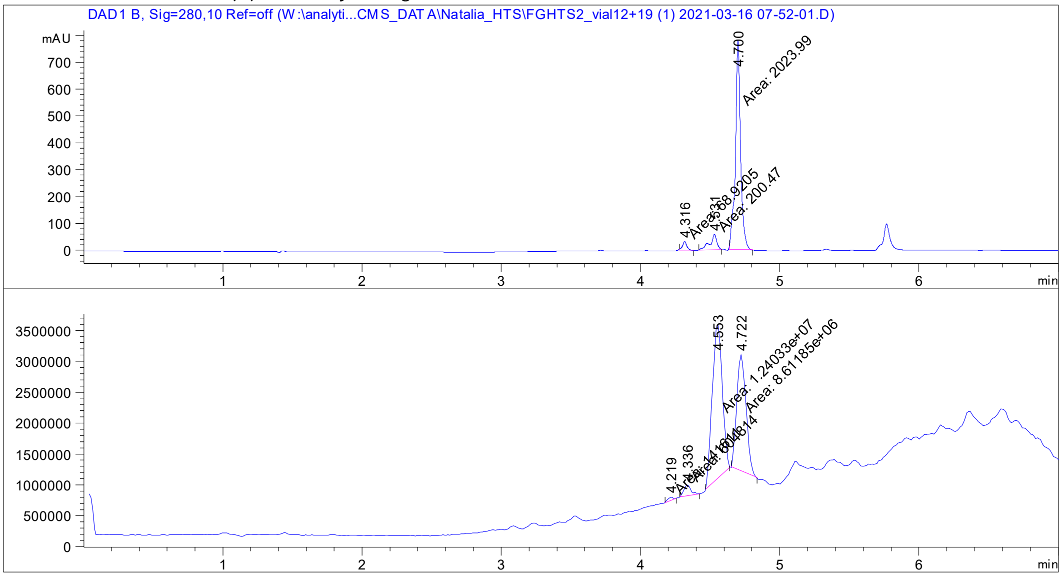

Data File W:\analytical_LCMS_DATA\Natalia_HTS\FGHTS2_vial12+19 (1) 2021-03-16 07-52-01.D
Sample Name: FGHTS2_vial12+19

MS Signal: MSD1 TIC, MS File, ES-API, Pos, Scan, Frag: 70, "PosScan"
Spectra averaged over upper half of peaks.
Noise Cutoff: 1000 counts.
Reportable Ion Abundance: > 10%.

Retention Time (MS)	MS Area	Mol. Weight or Ion
4.219	141611	316.25 I
		312.25 I
		310.25 I
		298.25 I
		297.20 I
		296.15 I
		295.20 I
		294.20 I
		284.25 I
		283.20 I
		282.25 I
		281.20 I
		280.20 I
		279.10 I
		278.25 I
		256.20 I
		214.15 I
		172.10 I
		148.10 I
		122.15 I
		102.20 I
4.336	604814	312.20 I
		310.20 I
		298.25 I
		296.20 I
		294.15 I
		284.25 I
		283.30 I
		282.30 I
		280.15 I
		279.10 I
		265.95 I
		265.05 I
		264.05 I
		256.20 I
		102.20 I
4.553	12403333	312.85 I
		310.85 I
		308.90 I
		271.85 I
		269.85 I
		267.85 I
4.722	8611846	498.90 I
		497.90 I
		496.90 I

Data File W:\analytical_LCMS_DATA\Natalia_HTS\FGHTS2_vial12+19 (1) 2021-03-16 07-52-01.D
Sample Name: FGHTS2_vial12+19

495.95 I
494.90 I

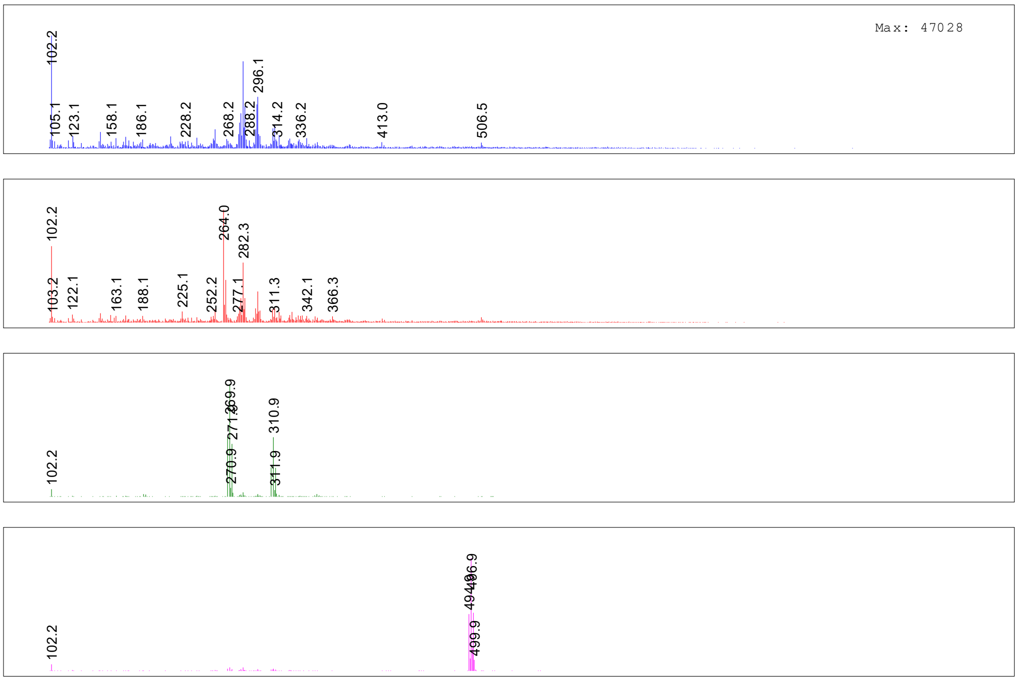

*** End of Report ***

Compound 31

```
Data File W:\analytical_LCMS_DATA\Natalia_HTS\FGMHTS2_19+11 (1) 2021-03-23 08-28-01.D
Sample Name: FGMHTS2_19+11

=====================================================================
Acq. Operator   : SYSTEM
Sample Operator : SYSTEM
Acq. Instrument : LCMS test                    Location :   D1B-B7
Injection Date  : 3/23/2021 10:02:40 AM             Inj :   1
                                             Inj Volume : 1.000 µl
Different Inj Volume from Sample Entry!   Actual Inj Volume : 1.500 µl
Acq. Method     : C:\Users\Public\Documents\ChemStation\1\Methods\0-Std_mth\Gradient\FAST_
                  Nonpolar_General_Method_MS-PosScan-100-1000.M
Last changed    : 2/18/2021 11:58:42 AM by SYSTEM
Analysis Method : C:\Users\Public\Documents\ChemStation\1\Methods\0-Std_mth\Gradient\SLOW_
                  Nonpolar_General_Method_MS-PosScan-100-1000.M
Last changed    : 4/21/2021 11:43:02 AM by SYSTEM
                  (modified after loading)
Method Info     : General method-use for RX monitoring

Additional Info : Peak(s) manually integrated
```

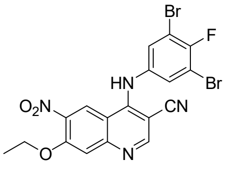

31

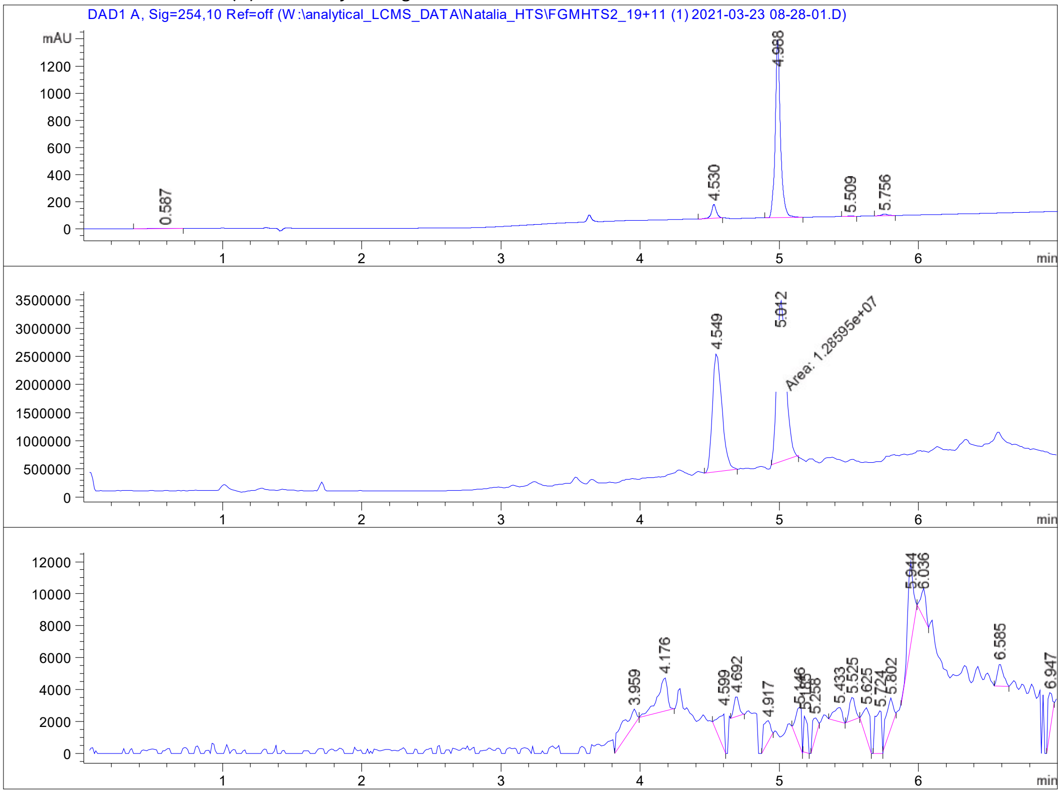

Data File W:\analytical_LCMS_DATA\Natalia_HTS\FGMHTS2_19+11 (1) 2021-03-23 08-28-01.D
Sample Name: FGMHTS2_19+11

MS Signal: MSD1 TIC, MS File, ES-API, Pos, Scan, Frag: 70, "PosScan"
Spectra averaged over upper half of peaks.
Noise Cutoff: 1000 counts.
Reportable Ion Abundance: > 10%.

Retention Time (MS)	MS Area	Mol. Weight or Ion
4.549	10091944	312.90 I
		310.90 I
		308.85 I
		271.85 I
		269.85 I
		267.85 I
5.012	12859451	513.90 I
		512.90 I
		511.95 I
		510.95 I
		509.90 I
		508.90 I

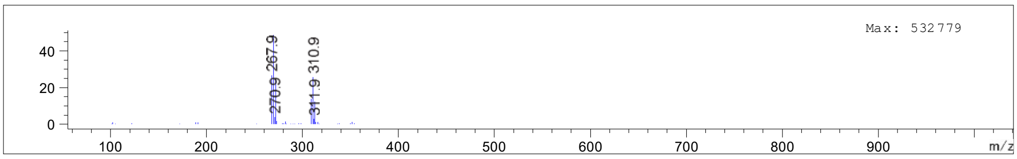

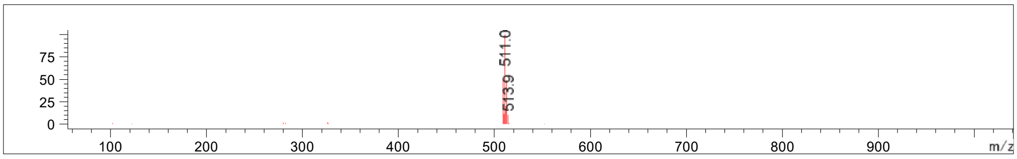

*** End of Report ***

Compound 32

Data File W:\analyti...S_DATA\Natalia_HTS\FGMHTS2_16+14_1.5micro (1) 2021-03-10 06-53-52.D
Sample Name: FGMHTS2_16+14_1.5micro

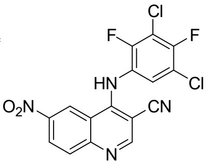

32

===
Acq. Operator : SYSTEM
Sample Operator : SYSTEM
Acq. Instrument : LCMS test   Location : D1F-C3
Injection Date : 3/10/2021 8:11:58 AM   Inj : 1
Inj Volume : 1.000 µl
Different Inj Volume from Sample Entry!   Actual Inj Volume : 1.500 µl
Acq. Method : C:\Users\Public\Documents\ChemStation\1\Methods\0-Std_mth\Gradient\FAST_Nonpolar_General_Method_MS-PosScan-100-1000.M
Last changed : 2/18/2021 11:58:42 AM by SYSTEM
Analysis Method : C:\Users\Public\Documents\ChemStation\1\Methods\DEF_LC.M
Last changed : 9/20/2021 11:46:17 AM by SYSTEM
(modified after loading)
Additional Info : Peak(s) manually integrated

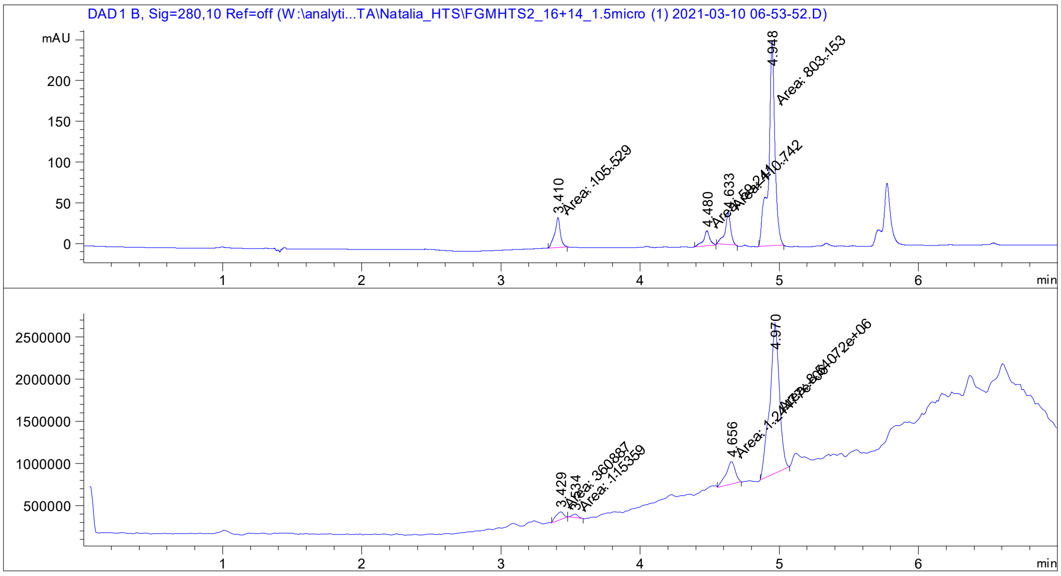

Data File W:\analyti...S_DATA\Natalia_HTS\FGMHTS2_16+14_1.5micro (1) 2021-03-10 06-53-52.D
Sample Name: FGMHTS2_16+14_1.5micro

MS Signal: MSD1 TIC, MS File, ES-API, Pos, Scan, Frag: 70, "PosScan"
Spectra averaged over upper half of peaks.
Noise Cutoff: 1000 counts.
Reportable Ion Abundance: > 10%.

Retention Time (MS)	MS Area	Mol. Weight or Ion
3.429	360887	282.20 I
		279.00 I
		217.05 I
		215.95 I
		148.05 I
3.534	115359	329.05 I
		283.35 I
		282.20 I
		280.05 I
		279.05 I
		256.25 I
		252.15 I
		230.15 I
		229.10 I
		224.20 I
		208.10 I
		207.15 I
		186.00 I
		172.10 I
		163.15 I
		148.05 I
		104.10 I
		102.15 I
4.656	1244767	316.25 I
		310.20 I
		296.10 I
		295.15 I
		283.30 I
		282.25 I
		280.15 I
		279.10 I
		256.25 I
		240.95 I
		238.95 I
		201.90 I
		200.00 I
		197.95 I
4.970	8540719	398.90 I
		397.90 I
		396.95 I
		395.95 I
		394.95 I

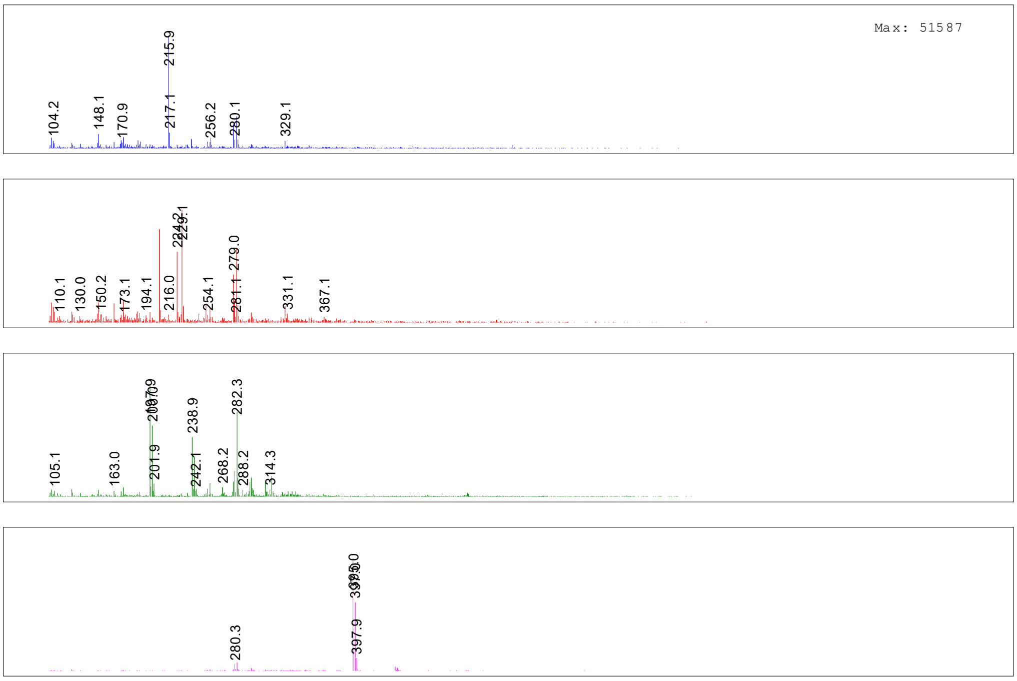

*** End of Report ***

Compound 33

```
Data File W:\analytical_LCMS_DATA\Natalia_HTS\FGHTS2_vial12+16 (1) 2021-03-16 07-51-17.D
Sample Name: FGHTS2_vial12+16

    =================================================================
    Acq. Operator   : SYSTEM
    Sample Operator : SYSTEM
    Acq. Instrument : LCMS test                     Location :   D1B-A6
    Injection Date  : 3/16/2021 10:19:53 AM              Inj :   1
                                                  Inj Volume : 1.000 µl
    Different Inj Volume from Sample Entry!   Actual Inj Volume : 1.500 µl
    Acq. Method     : C:\Users\Public\Documents\ChemStation\1\Methods\0-Std_mth\Gradient\FAST_
                      Nonpolar_General_Method_MS-PosScan-100-1000.M
    Last changed    : 2/18/2021 11:58:42 AM by SYSTEM
    Analysis Method : C:\Users\Public\Documents\ChemStation\1\Methods\DEF_LC.M
    Last changed    : 9/20/2021 3:18:40 PM by SYSTEM
                      (modified after loading)
    Additional Info : Peak(s) manually integrated
```

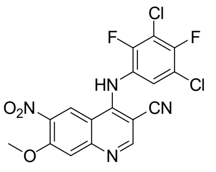

33

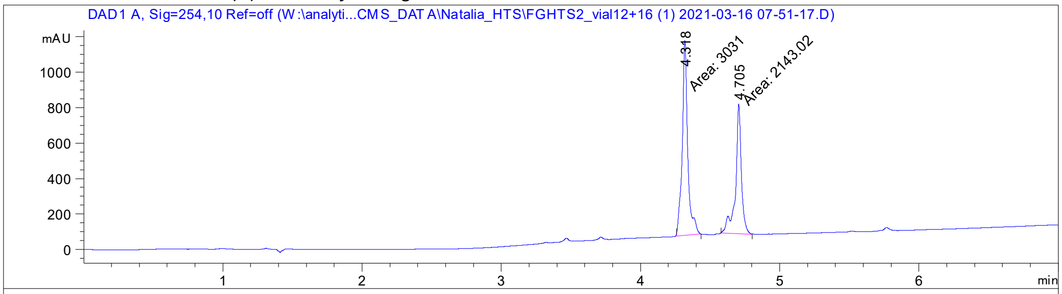

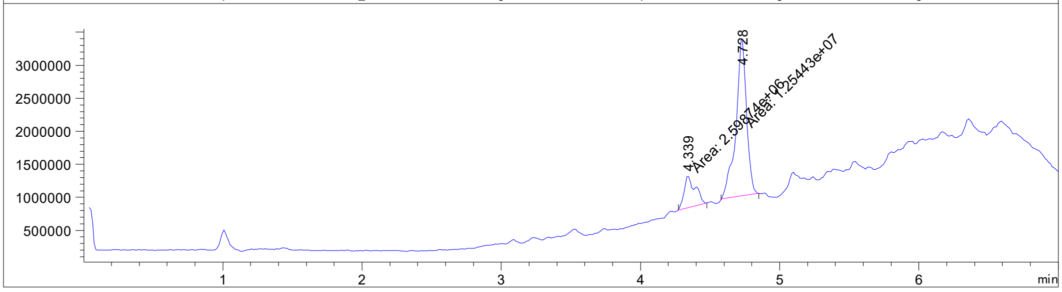

Data File W:\analytical_LCMS_DATA\Natalia_HTS\FGHTS2_vial12+16 (1) 2021-03-16 07-51-17.D
Sample Name: FGHTS2_vial12+16

MS Signal: MSD1 TIC, MS File, ES-API, Pos, Scan, Frag: 70, "PosScan"
Spectra averaged over upper half of peaks.
Noise Cutoff: 1000 counts.
Reportable Ion Abundance: > 10%.

Retention Time (MS)	MS Area	Mol. Weight or Ion
4.339	2598745	298.25 I
		296.25 I
		282.25 I
		280.25 I
		275.00 I
		274.05 I
		265.95 I
		265.00 I
		264.00 I
		102.15 I
4.728	12544332	429.00 I
		428.00 I
		426.95 I
		426.00 I
		425.00 I

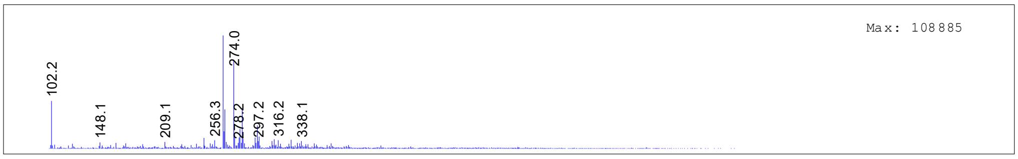

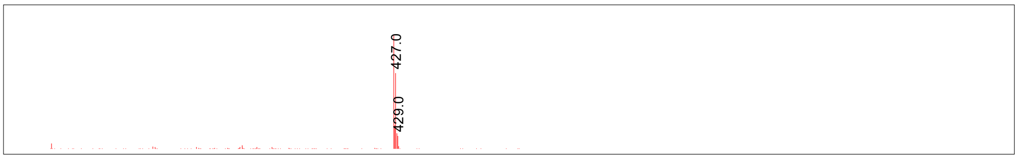

*** End of Report ***

Compound 34

Data File W:\analytical_LCMS_DATA\Natalia_HTS\FGMHTS2_16+11 (1) 2021-03-23 08-27-11.D
Sample Name: FGMHTS2_16+11

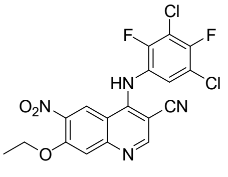

34

===

Acq. Operator : SYSTEM
Sample Operator : SYSTEM
Acq. Instrument : LCMS test   Location : D1B-B3
Injection Date : 3/23/2021 8:59:35 AM   Inj : 1
                                        Inj Volume : 1.000 µl
Different Inj Volume from Sample Entry!   Actual Inj Volume : 1.500 µl
Acq. Method : C:\Users\Public\Documents\ChemStation\1\Methods\0-Std_mth\Gradient\FAST_Nonpolar_General_Method_MS-PosScan-100-1000.M
Last changed : 2/18/2021 11:58:42 AM by SYSTEM
Analysis Method : C:\Users\Public\Documents\ChemStation\1\Methods\0-Std_mth\Gradient\SLOW_Nonpolar_General_Method_MS-PosScan-100-1000.M
Last changed : 4/22/2021 2:41:27 PM by SYSTEM
(modified after loading)
Method Info : General method-use for RX monitoring

Additional Info : Peak(s) manually integrated

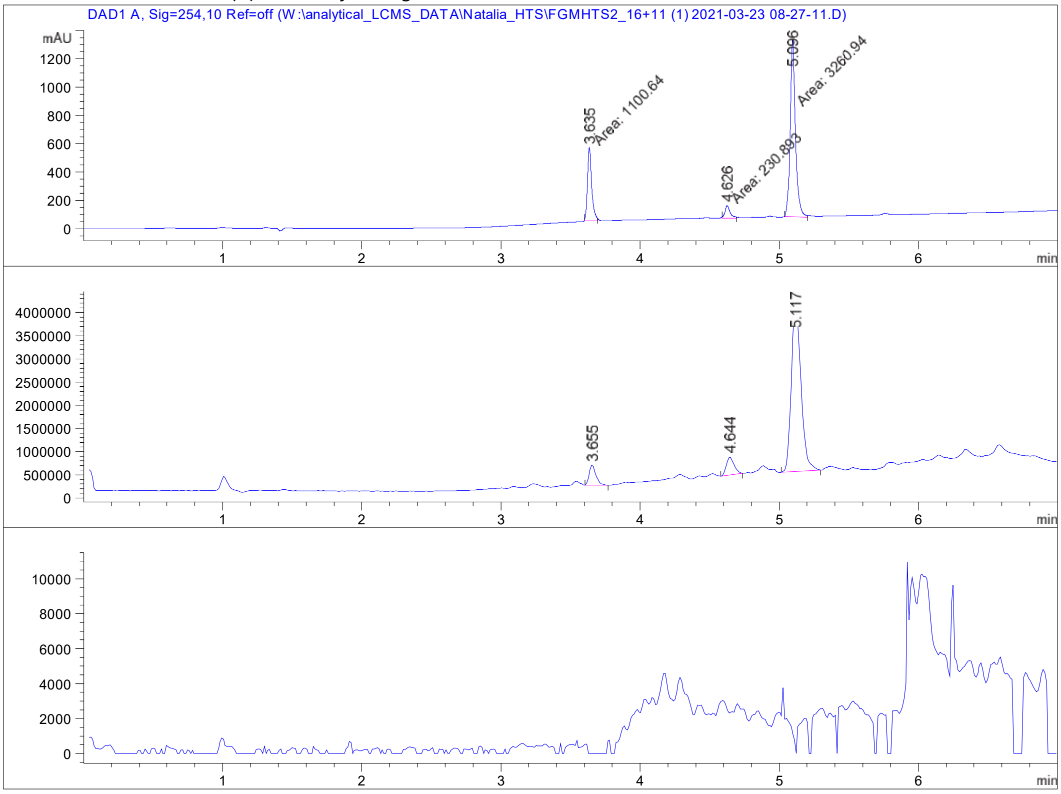

Data File W:\analytical_LCMS_DATA\Natalia_HTS\FGMHTS2_16+11 (1) 2021-03-23 08-27-11.D
Sample Name: FGMHTS2_16+11

MS Signal: MSD1 TIC, MS File, ES-API, Pos, Scan, Frag: 70, "PosScan"
Spectra averaged over upper half of peaks.
Noise Cutoff: 1000 counts.
Reportable Ion Abundance: > 10%.

Retention Time (MS)	MS Area	Mol. Weight or Ion
3.655	1440661	261.05 I
		260.05 I
4.644	1479829	282.20 I
		240.95 I
		238.95 I
		201.90 I
		199.90 I
		197.90 I
		102.20 I
5.117	17666308	442.95 I
		441.95 I
		440.95 I
		440.05 I
		439.00 I

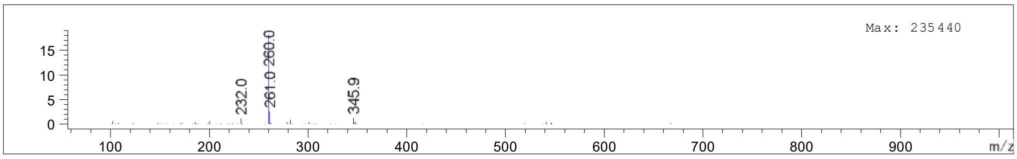

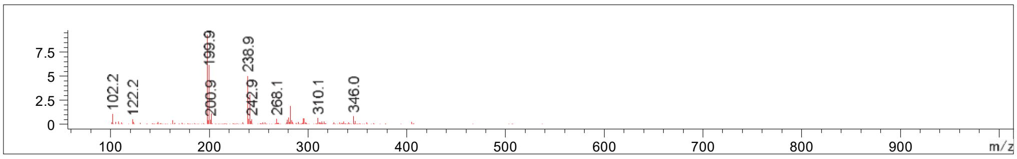

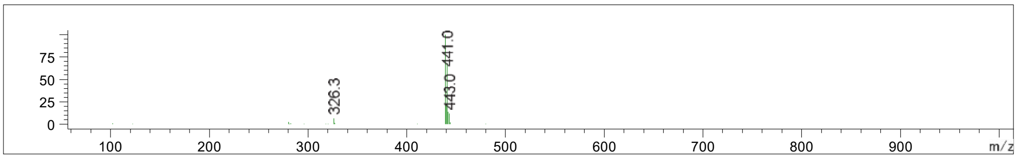

*** End of Report ***

Compound 35

```
Data File W:\analyti...MS_DATA\Natalia_HTS\FGMHTS2_9+14_1.5micro (1) 2021-03-10 06-53-19.D
Sample Name: FGMHTS2_9+14_1.5micro

     =====================================================================
     Acq. Operator   : SYSTEM
     Sample Operator : SYSTEM
     Acq. Instrument : LCMS test                      Location :   D1F-B9
     Injection Date  : 3/10/2021 7:40:40 AM                Inj :   1
                                                   Inj Volume : 1.000 µl
     Different Inj Volume from Sample Entry!   Actual Inj Volume : 1.500 µl
     Acq. Method     : C:\Users\Public\Documents\ChemStation\1\Methods\0-Std_mth\Gradient\FAST_
                       Nonpolar_General_Method_MS-PosScan-100-1000.M
     Last changed    : 2/18/2021 11:58:42 AM by SYSTEM
     Analysis Method : C:\Users\Public\Documents\ChemStation\1\Methods\DEF_LC.M
     Last changed    : 9/20/2021 11:46:17 AM by SYSTEM
                       (modified after loading)
     Additional Info : Peak(s) manually integrated
```

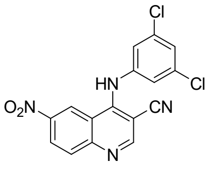

35

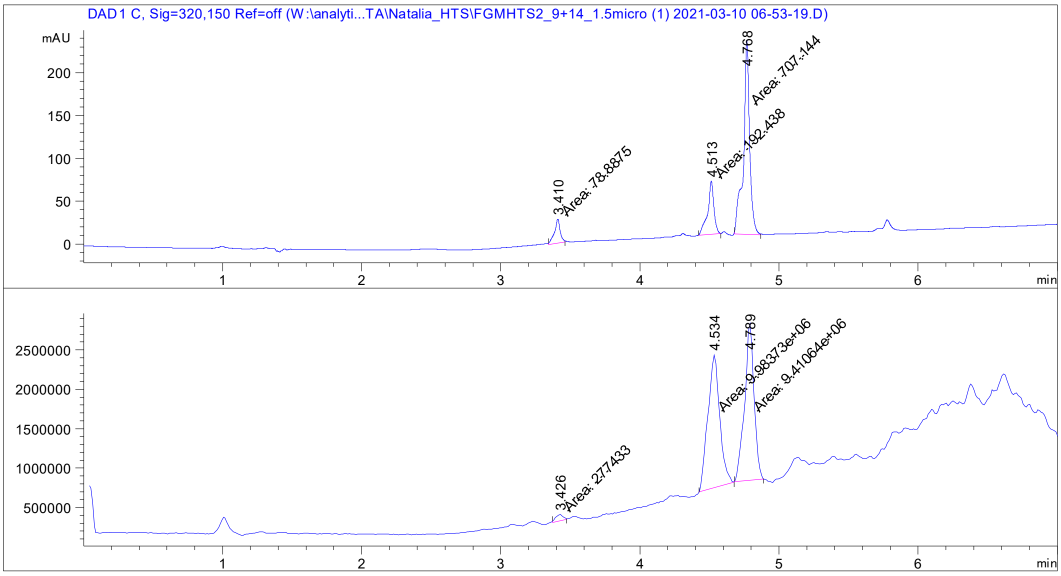

Data File W:\analyti...MS_DATA\Natalia_HTS\FGMHTS2_9+14_1.5micro (1) 2021-03-10 06-53-19.D
Sample Name: FGMHTS2_9+14_1.5micro

MS Signal: MSD1 TIC, MS File, ES-API, Pos, Scan, Frag: 70, "PosScan"
Spectra averaged over upper half of peaks.
Noise Cutoff: 1000 counts.
Reportable Ion Abundance: > 10%.

Retention Time (MS)	MS Area	Mol. Weight or Ion
3.426	277433	282.20 I
		280.10 I
		279.05 I
		256.30 I
		217.05 I
		216.00 I
		186.05 I
		172.15 I
		148.10 I
		104.10 I
		102.20 I
4.534	9983731	205.00 I
		203.00 I
		165.95 I
		163.95 I
		162.00 I
4.789	9410639	362.95 I
		361.95 I
		360.95 I
		360.00 I
		358.95 I

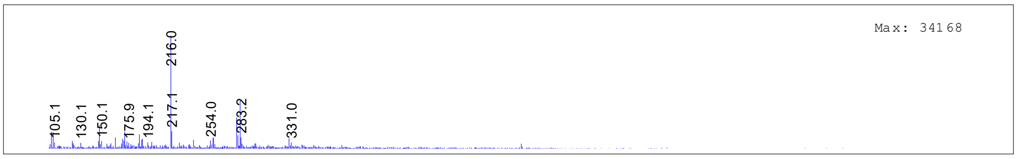

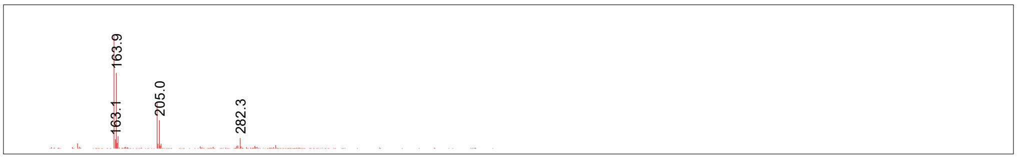

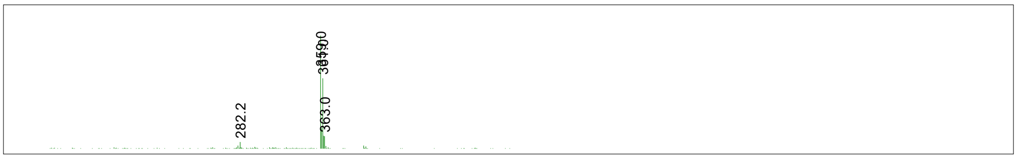

*** End of Report ***

Compound 36

```
Data File W:\analytical_LCMS_DATA\Natalia_HTS\FGHTS2_vial9 (1) 2021-03-09 10-41-24.D
Sample Name: FGHTS2_vial9

    =====================================================================
    Acq. Operator    : SYSTEM
    Sample Operator  : SYSTEM
    Acq. Instrument  : LCMS test                        Location :   D1F-F4
    Injection Date   : 3/9/2021 1:20:59 PM                   Inj :   1
                                                      Inj Volume : 1.000 µl
    Different Inj Volume from Sample Entry!   Actual Inj Volume : 1.200 µl
    Acq. Method      : C:\Users\Public\Documents\ChemStation\1\Methods\0-Std_mth\Gradient\FAST_
                       Nonpolar_General_Method_MS-PosScan-100-1000.M
    Last changed     : 2/18/2021 11:58:42 AM by SYSTEM
    Analysis Method  : C:\Users\Public\Documents\ChemStation\1\Methods\DEF_LC.M
    Last changed     : 9/20/2021 3:18:40 PM by SYSTEM
                       (modified after loading)
    Additional Info  : Peak(s) manually integrated
```

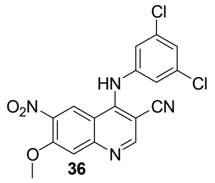

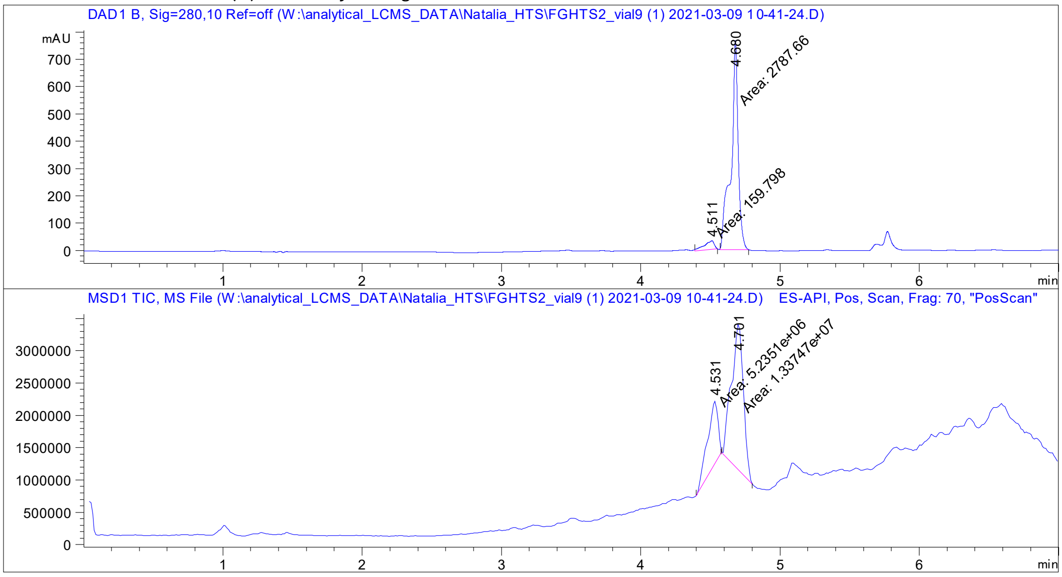

Data File W:\analytical_LCMS_DATA\Natalia_HTS\FGHTS2_vial9 (1) 2021-03-09 10-41-24.D
Sample Name: FGHTS2_vial9

MS Signal: MSD1 TIC, MS File, ES-API, Pos, Scan, Frag: 70, "PosScan"
Spectra averaged over upper half of peaks.
Noise Cutoff: 1000 counts.
Reportable Ion Abundance: > 10%.

Retention Time (MS)	MS Area	Mol. Weight or Ion
4.531	5235095	282.20 I
		205.05 I
		203.00 I
		165.95 I
		163.95 I
		161.95 I
4.701	13374704	392.95 I
		392.00 I
		391.00 I
		390.00 I
		389.00 I

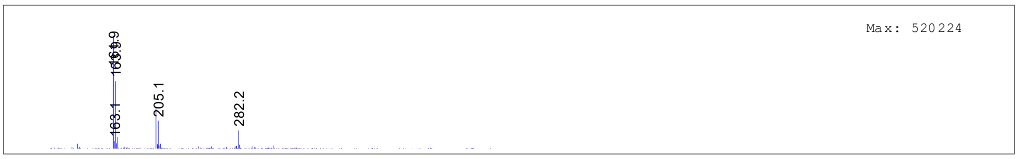

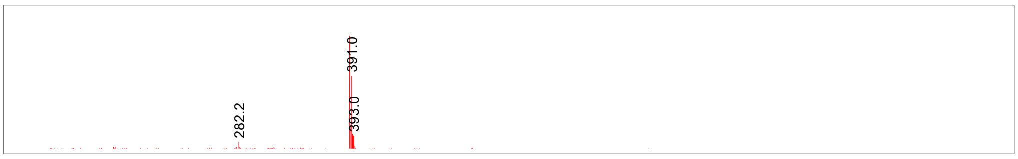

*** End of Report ***

Compound 37

```
Data File W:\analytical_LCMS_DATA\Natalia_HTS\FGMHTS2_9+11 (1) 2021-03-23 07-41-39.D
Sample Name: FGMHTS2_9+11

    =====================================================================
    Acq. Operator   : SYSTEM
    Sample Operator : SYSTEM
    Acq. Instrument : LCMS test                    Location :   D1B-A9
    Injection Date  : 3/23/2021 8:14:37 AM              Inj :   1
                                                 Inj Volume : 1.000 µl
    Different Inj Volume from Sample Entry!   Actual Inj Volume : 1.500 µl
    Acq. Method     : C:\Users\Public\Documents\ChemStation\1\Methods\0-Std_mth\Gradient\FAST_
                      Nonpolar_General_Method_MS-PosScan-100-1000.M
    Last changed    : 2/18/2021 11:58:42 AM by SYSTEM
    Analysis Method : C:\Users\Public\Documents\ChemStation\1\Methods\0-Std_mth\Gradient\SLOW_
                      Nonpolar_General_Method_MS-PosScan-100-1000.M
    Last changed    : 4/22/2021 2:41:27 PM by SYSTEM
                      (modified after loading)
    Method Info     : General method-use for RX monitoring

    Additional Info : Peak(s) manually integrated
```

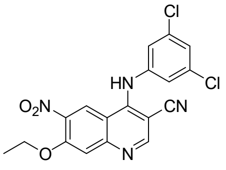

37

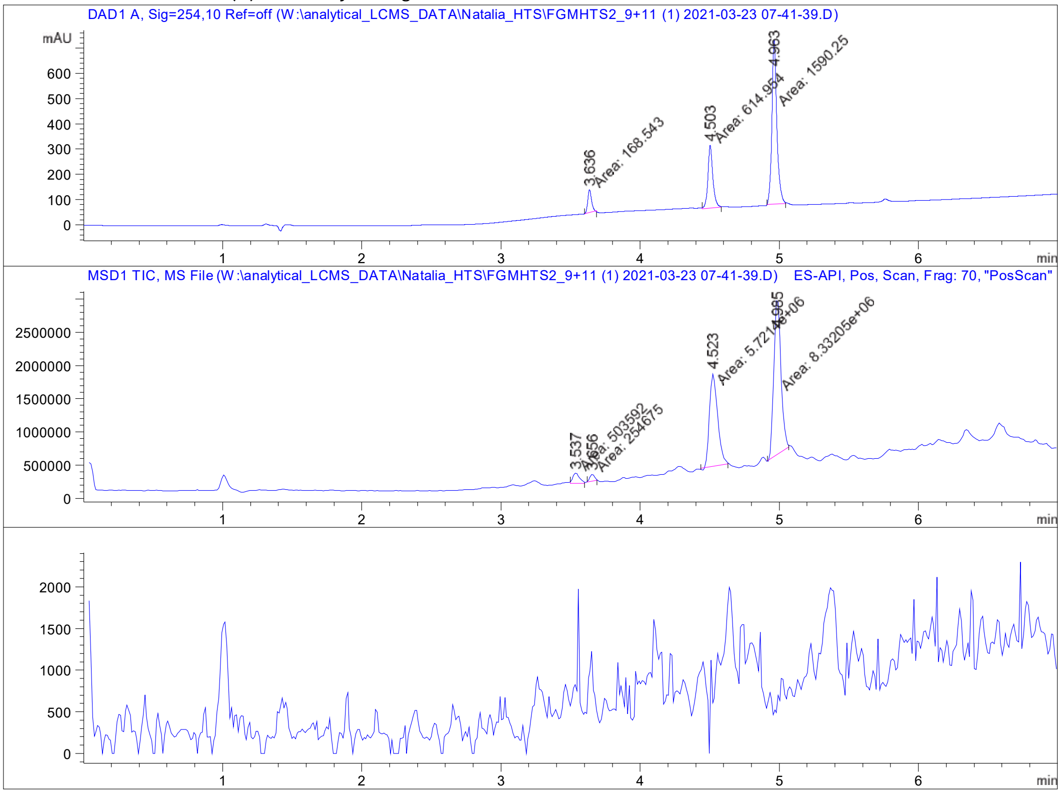

Data File W:\analytical_LCMS_DATA\Natalia_HTS\FGMHTS2_9+11 (1) 2021-03-23 07-41-39.D
Sample Name: FGMHTS2_9+11

MS Signal: MSD1 TIC, MS File, ES-API, Pos, Scan, Frag: 70, "PosScan"
Spectra averaged over upper half of peaks.
Noise Cutoff: 1000 counts.
Reportable Ion Abundance: > 10%.

Retention Time (MS)	MS Area	Mol. Weight or Ion
3.537	503592	282.20 I
		252.20 I
		229.05 I
		224.15 I
		208.15 I
		207.10 I
		102.20 I
3.656	254675	282.15 I
		261.00 I
		260.05 I
		200.15 I
		102.15 I
4.523	5721404	205.05 I
		203.00 I
		166.00 I
		163.95 I
		161.95 I
4.985	8332047	407.00 I
		406.05 I
		405.00 I
		404.05 I
		402.95 I

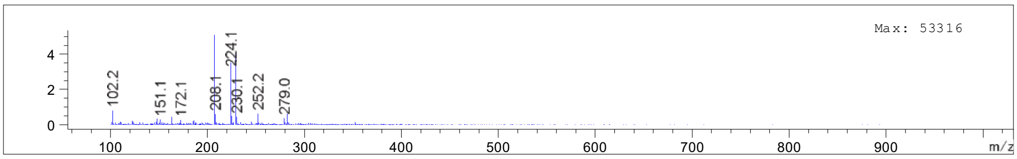

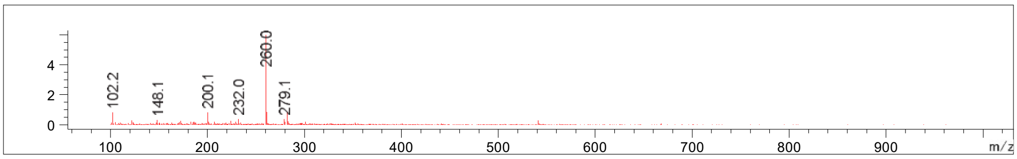

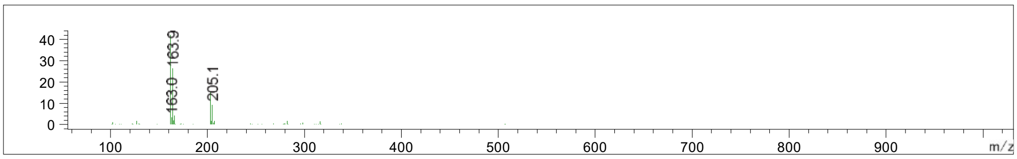

Data File W:\analytical_LCMS_DATA\Natalia_HTS\FGMHTS2_9+11 (1) 2021-03-23 07-41-39.D
Sample Name: FGMHTS2_9+11

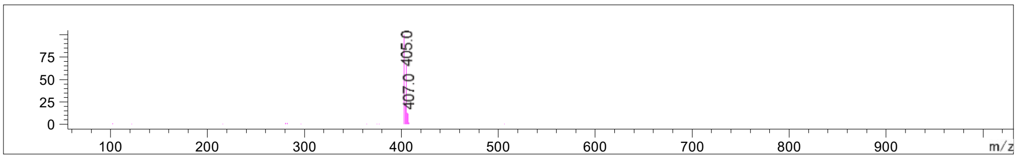

*** End of Report ***

Compound 38

```
Data File W:\analyti...MS_DATA\Natalia_HTS\FGMHTS2_8+14_1.5micro (1) 2021-03-10 06-53-09.D
Sample Name: FGMHTS2_8+14_1.5micro

    =====================================================================
    Acq. Operator   : SYSTEM
    Sample Operator : SYSTEM
    Acq. Instrument : LCMS test                   Location :   D1F-B8
    Injection Date  : 3/10/2021 7:30:13 AM             Inj :   1
                                                Inj Volume : 1.000 µl
    Different Inj Volume from Sample Entry!  Actual Inj Volume : 1.500 µl
    Acq. Method     : C:\Users\Public\Documents\ChemStation\1\Methods\0-Std_mth\Gradient\FAST_
                      Nonpolar_General_Method_MS-PosScan-100-1000.M
    Last changed    : 2/18/2021 11:58:42 AM by SYSTEM
    Analysis Method : C:\Users\Public\Documents\ChemStation\1\Methods\DEF_LC.M
    Last changed    : 9/20/2021 11:46:17 AM by SYSTEM
                      (modified after loading)
    Additional Info : Peak(s) manually integrated
```

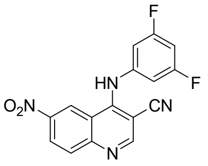

38

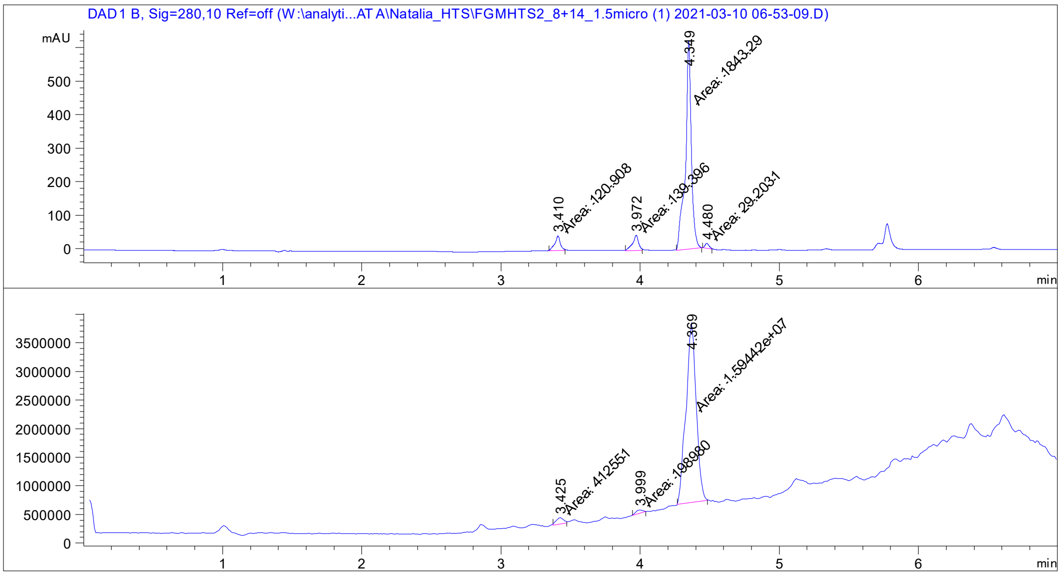

Data File W:\analyti...MS_DATA\Natalia_HTS\FGMHTS2_8+14_1.5micro (1) 2021-03-10 06-53-09.D
Sample Name: FGMHTS2_8+14_1.5micro

MS Signal: MSD1 TIC, MS File, ES-API, Pos, Scan, Frag: 70, "PosScan"
Spectra averaged over upper half of peaks.
Noise Cutoff: 1000 counts.
Reportable Ion Abundance: > 10%.

Retention Time (MS)	MS Area	Mol. Weight or Ion
3.425	412551	282.25 I
		279.05 I
		257.00 I
		216.90 I
		216.05 I
		172.05 I
		148.10 I
		104.10 I
		102.15 I
3.999	198980	343.20 I
		296.15 I
		288.20 I
		283.25 I
		282.20 I
		280.20 I
		279.00 I
		256.20 I
		172.10 I
		171.05 I
		130.10 I
4.369	15944161	328.00 I
		327.05 I

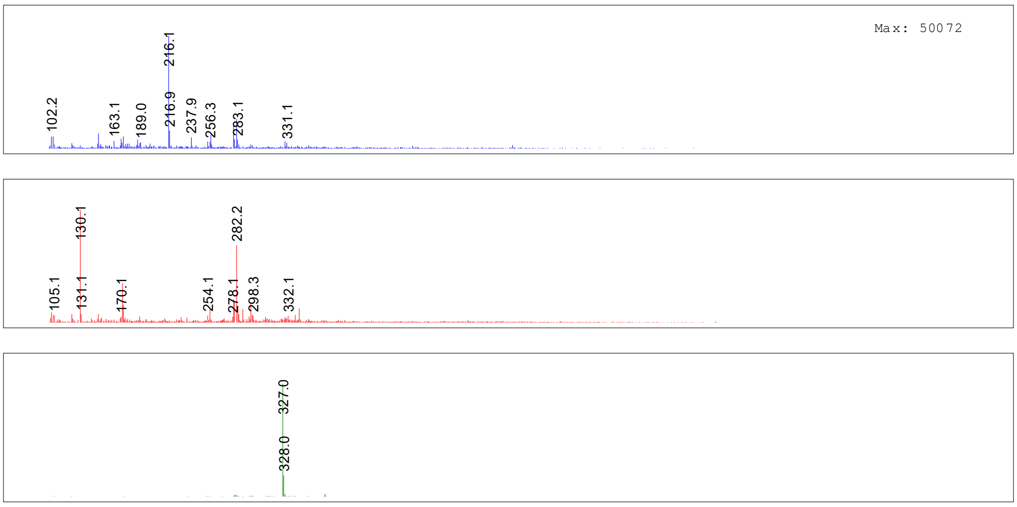

*** End of Report ***

Compound 39

```
Data File W:\analytical_LCMS_DATA\Natalia_HTS\FGHTS2_vial8 (1) 2021-03-09 10-41-09.D
Sample Name: FGHTS2_vial8

    =================================================================
    Acq. Operator   : SYSTEM
    Sample Operator : SYSTEM
    Acq. Instrument : LCMS test                     Location :   D1F-F3
    Injection Date  : 3/9/2021 12:42:25 PM               Inj :   1
                                                  Inj Volume : 1.000 µl
    Different Inj Volume from Sample Entry!   Actual Inj Volume : 1.200 µl
    Acq. Method     : C:\Users\Public\Documents\ChemStation\1\Methods\0-Std_mth\Gradient\FAST_
                      Nonpolar_General_Method_MS-PosScan-100-1000.M
    Last changed    : 2/18/2021 11:58:42 AM by SYSTEM
    Analysis Method : C:\Users\Public\Documents\ChemStation\1\Methods\DEF_LC.M
    Last changed    : 9/20/2021 3:18:40 PM by SYSTEM
                      (modified after loading)
    Additional Info : Peak(s) manually integrated
```

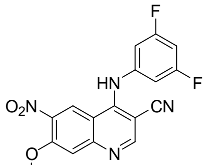

39

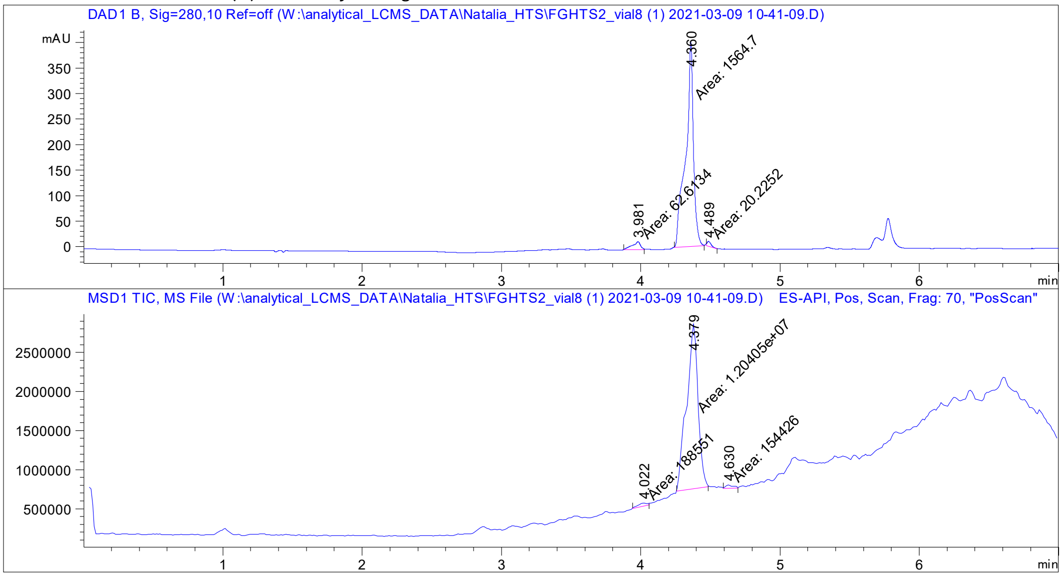

Data File W:\analytical_LCMS_DATA\Natalia_HTS\FGHTS2_vial8 (1) 2021-03-09 10-41-09.D
Sample Name: FGHTS2_vial8

MS Signal: MSD1 TIC, MS File, ES-API, Pos, Scan, Frag: 70, "PosScan"
Spectra averaged over upper half of peaks.
Noise Cutoff: 1000 counts.
Reportable Ion Abundance: > 10%.

Retention Time (MS)	MS Area	Mol. Weight or Ion
4.022	188551	343.20 I
		297.25 I
		296.20 I
		288.25 I
		283.15 I
		282.25 I
		280.20 I
		279.05 I
		256.20 I
		172.10 I
		171.10 I
		148.10 I
		130.05 I
		122.10 I
		102.20 I
4.379	12040517	358.05 I
		357.05 I
4.630	154426	316.30 I
		310.20 I
		297.20 I
		296.25 I
		295.15 I
		283.20 I
		282.20 I
		280.20 I
		279.05 I
		268.20 I
		256.25 I
		172.10 I

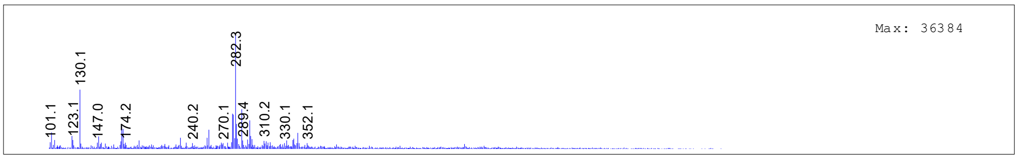

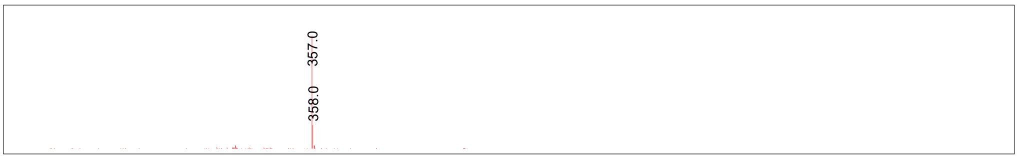

Data File W:\analytical_LCMS_DATA\Natalia_HTS\FGHTS2_vial8 (1) 2021-03-09 10-41-09.D
Sample Name: FGHTS2_vial8

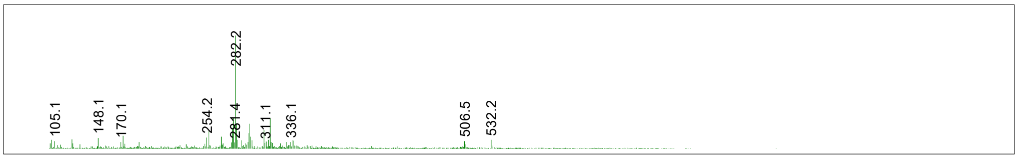

*** End of Report ***

Compound 40

```
Data File W:\analytical_LCMS_DATA\Natalia_HTS\FGMHTS2_8+11 (1) 2021-03-23 07-25-10.D
Sample Name: FGMHTS2_8+11

    =====================================================================
    Acq. Operator   : SYSTEM
    Sample Operator : SYSTEM
    Acq. Instrument : LCMS test                    Location :   D1B-A8
    Injection Date  : 3/23/2021 8:04:11 AM              Inj :   1
                                               Inj Volume : 1.000 µl
    Different Inj Volume from Sample Entry!   Actual Inj Volume : 1.500 µl
    Acq. Method     : C:\Users\Public\Documents\ChemStation\1\Methods\0-Std_mth\Gradient\FAST_
                      Nonpolar_General_Method_MS-PosScan-100-1000.M
    Last changed    : 2/18/2021 11:58:42 AM by SYSTEM
    Analysis Method : C:\Users\Public\Documents\ChemStation\1\Methods\0-Std_mth\Gradient\SLOW_
                      Nonpolar_General_Method_MS-PosScan-100-1000.M
    Last changed    : 4/22/2021 2:41:27 PM by SYSTEM
                      (modified after loading)
    Method Info     : General method-use for RX monitoring

    Additional Info : Peak(s) manually integrated
```

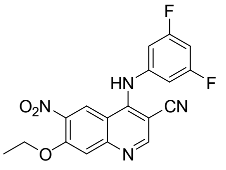

40

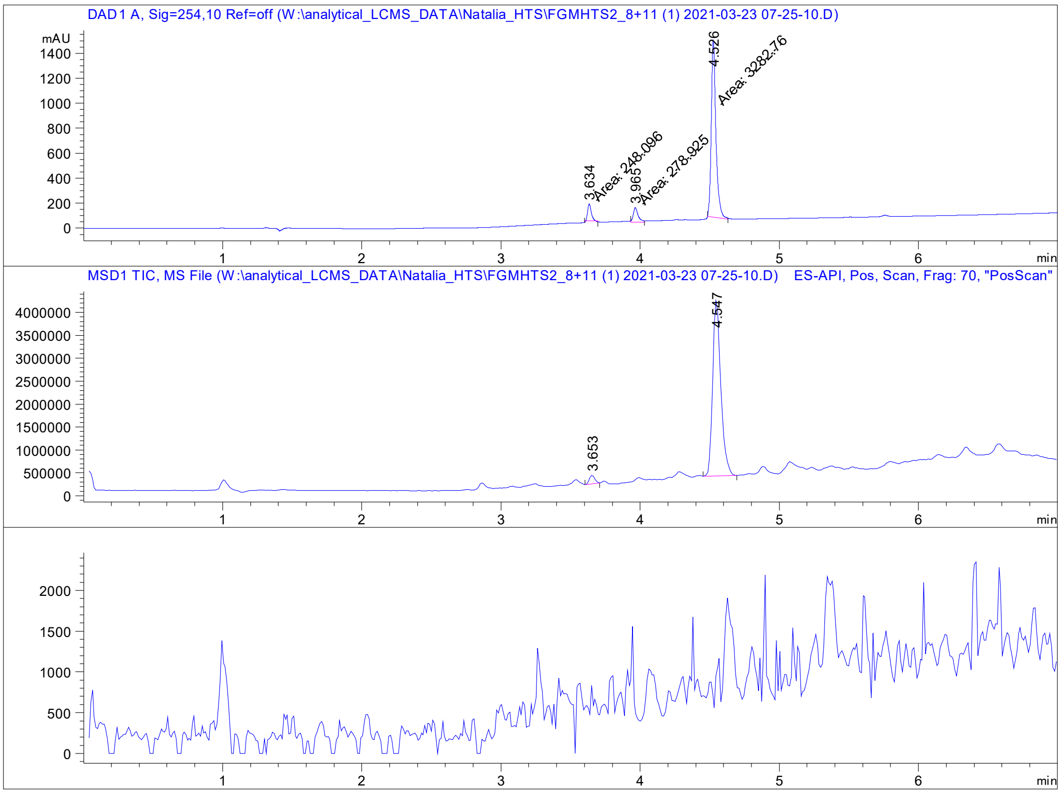

Data File W:\analytical_LCMS_DATA\Natalia_HTS\FGMHTS2_8+11 (1) 2021-03-23 07-25-10.D
Sample Name: FGMHTS2_8+11

MS Signal: MSD1 TIC, MS File, ES-API, Pos, Scan, Frag: 70, "PosScan"
Spectra averaged over upper half of peaks.
Noise Cutoff: 1000 counts.
Reportable Ion Abundance: > 10%.

Retention Time (MS)	MS Area	Mol. Weight or Ion
3.653	517966	346.05 I
		282.10 I
		261.05 I
		260.00 I
4.547	15702693	372.05 I
		371.05 I

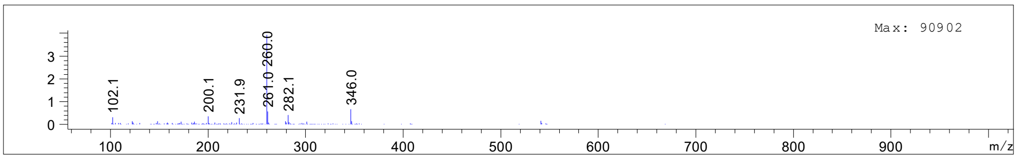

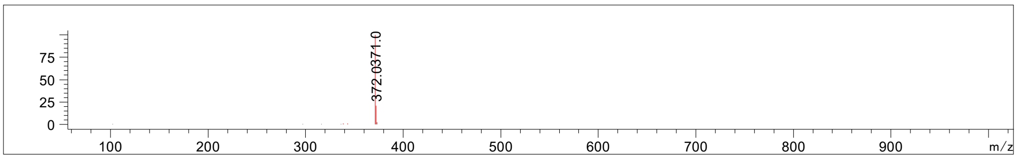

*** End of Report ***

Compound 1

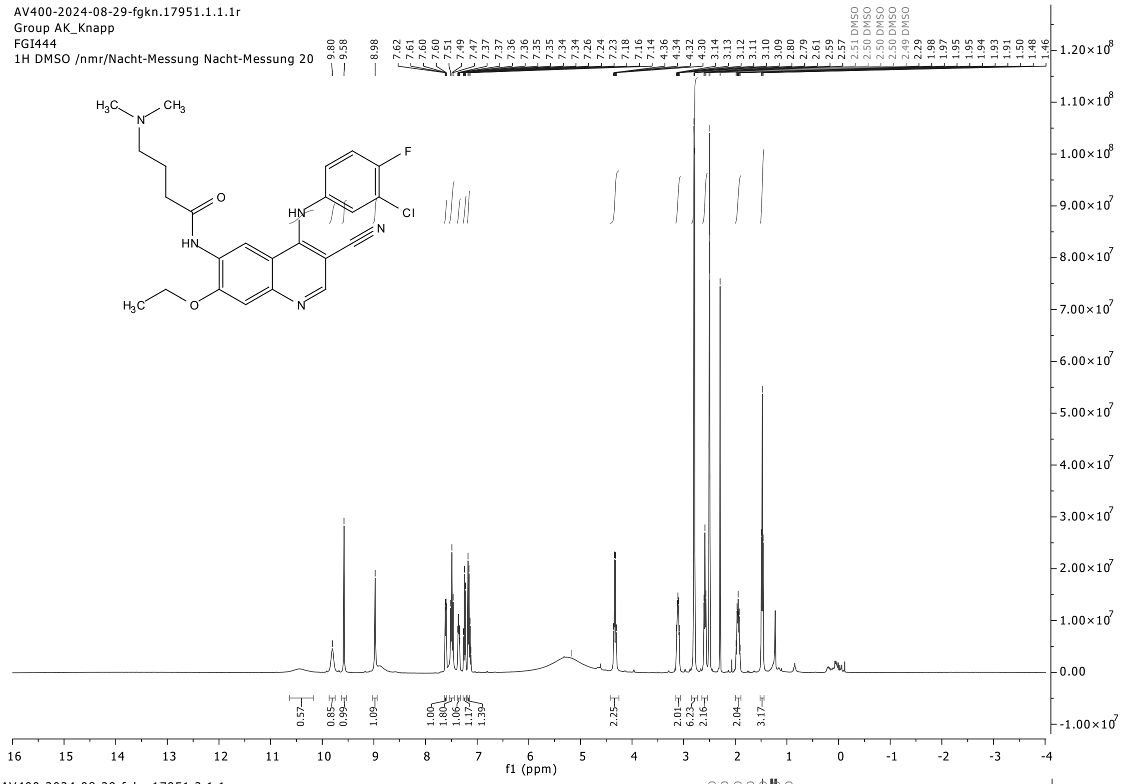
AV400-2024-08-29-fgkn.17951.1.1.1r
Group AK_Knapp
FGI444
1H DMSO /nmr/Nacht-Messung Nacht-Messung 20
f1 (ppm)

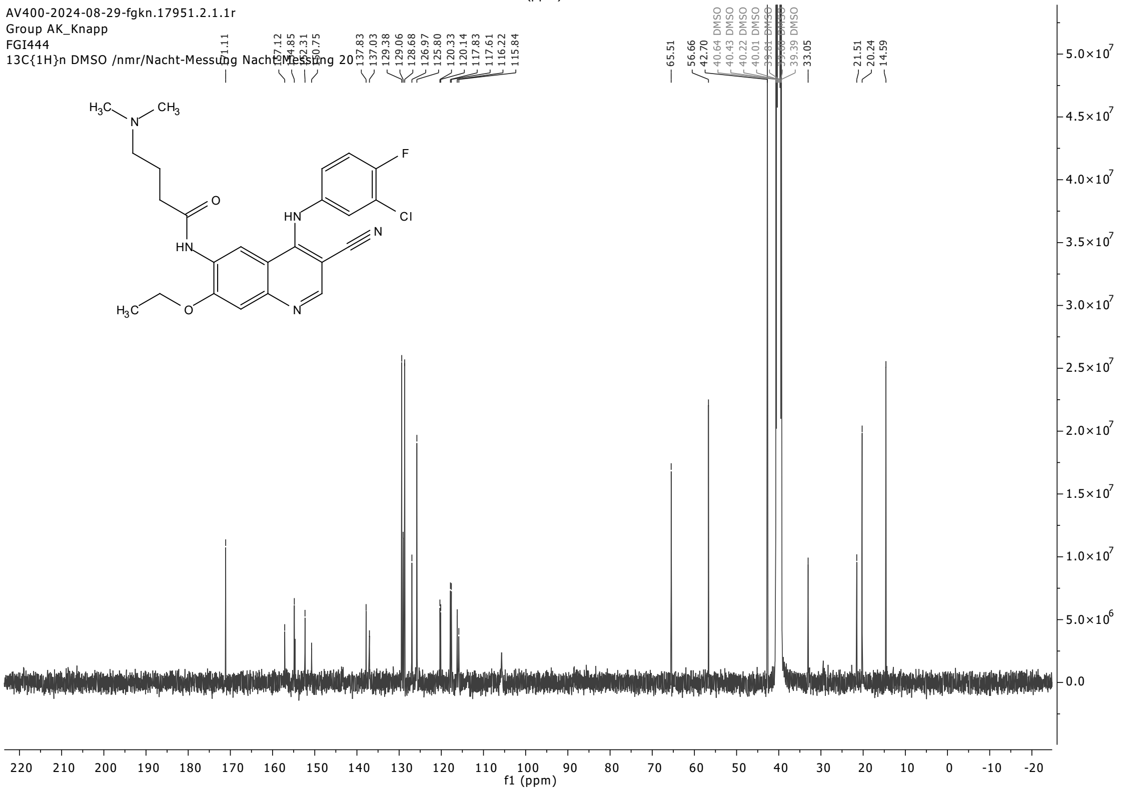
AV400-2024-08-29-fgkn.17951.2.1.1r
Group AK_Knapp
FGI444
13C{1H}n DMSO /nmr/Nacht-Messung Nacht-Messung 20
f1 (ppm)

Sample Report

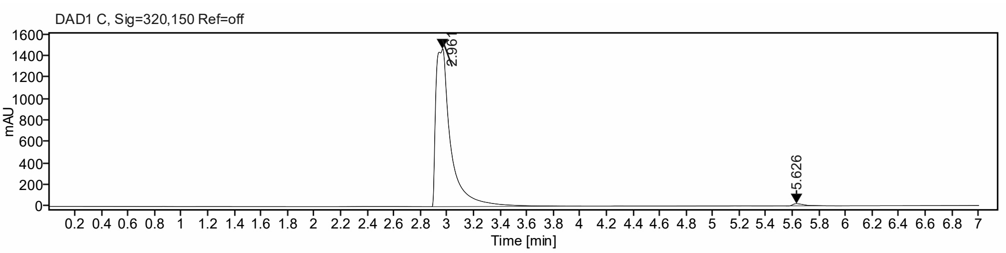

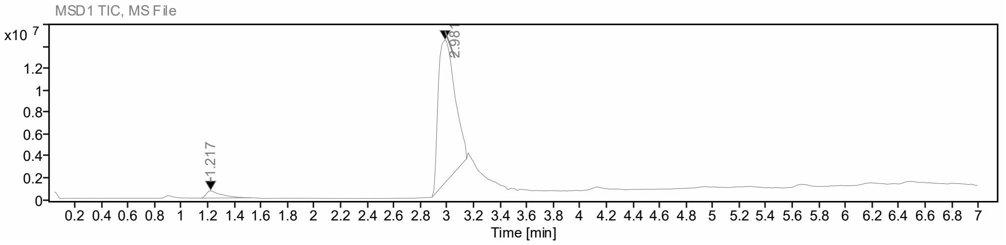

Sample Purity

Signal Description DAD1 C, Sig=320,150 Ref=off

Sample Name	Name	RT	Width	Area	Area%	Height
FGI444		2.961	0.104	11574.2412	98.94	1472.7284
FGI444		5.626	0.089	123.5799	1.06	20.1241

Max Area% 98.944

UV Signal Purity>95% Pass

UV Apex Spectra

RT:

2.961

Sample Name:

FGI444

Signal Name:

DAD1C

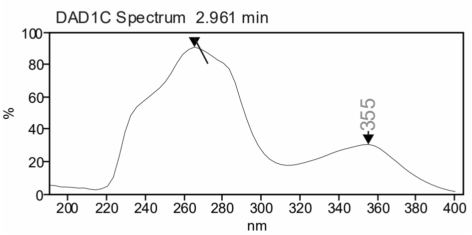

GOETHE UNIVERSITÄT FRANKFURT AM MAIN

RT:

5.626

Sample Name:

FGI444

Signal Name:

DAD1C

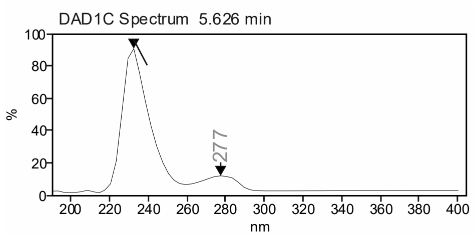

MSD Apex Spectra

RT:

1.217

Sample Name:

FGI444

Signal Name:

MSD1TIC

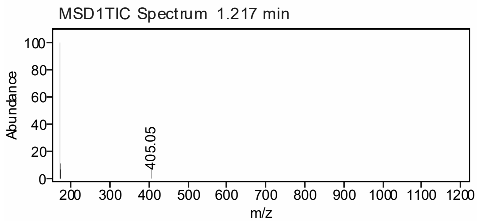

RT:

2.981

Sample Name:

FGI444

Signal Name:

MSD1TIC

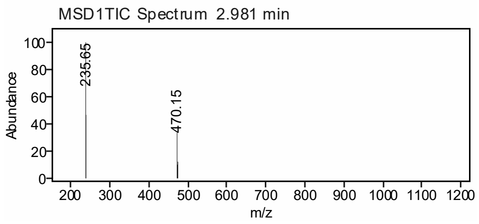

Mass Spectrum SmartFormula Report

Analysis Info

Analysis Name	D:\Data\MS_Service\2024Q3\20240830_SKN_FG_000065\SKN_FG_000065_FGI444_1.d	Acquisition Date	8/30/2024 4:14:12 PM
Method	Positive(100-1600)_Infusion_200uL-min.m	Operator	BDAL@DE
Sample Name	Te st1	Instrument	micrOTOF-Q 228888.10407
Comment			

Acquisition Parameter

Source Type	ESI	Ion Polarity	Positive	Set Nebulizer	1.8 Bar
Focus	Active	Set Capillary	4000 V	Set Dry Heater	280 °C
Scan Begin	100 m/z	Set End Plate Offset	-500 V	Set Dry Gas	8.0 l/min
Scan End	1600 m/z	Set Collision Cell RF	150.0 Vpp	Set Divert Valve	Waste

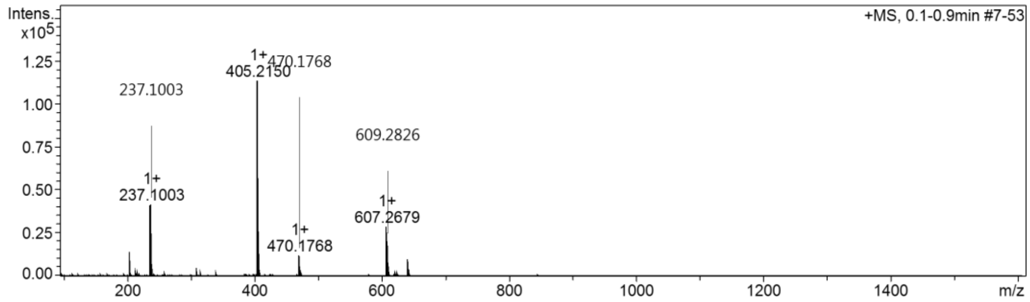

Compound 63

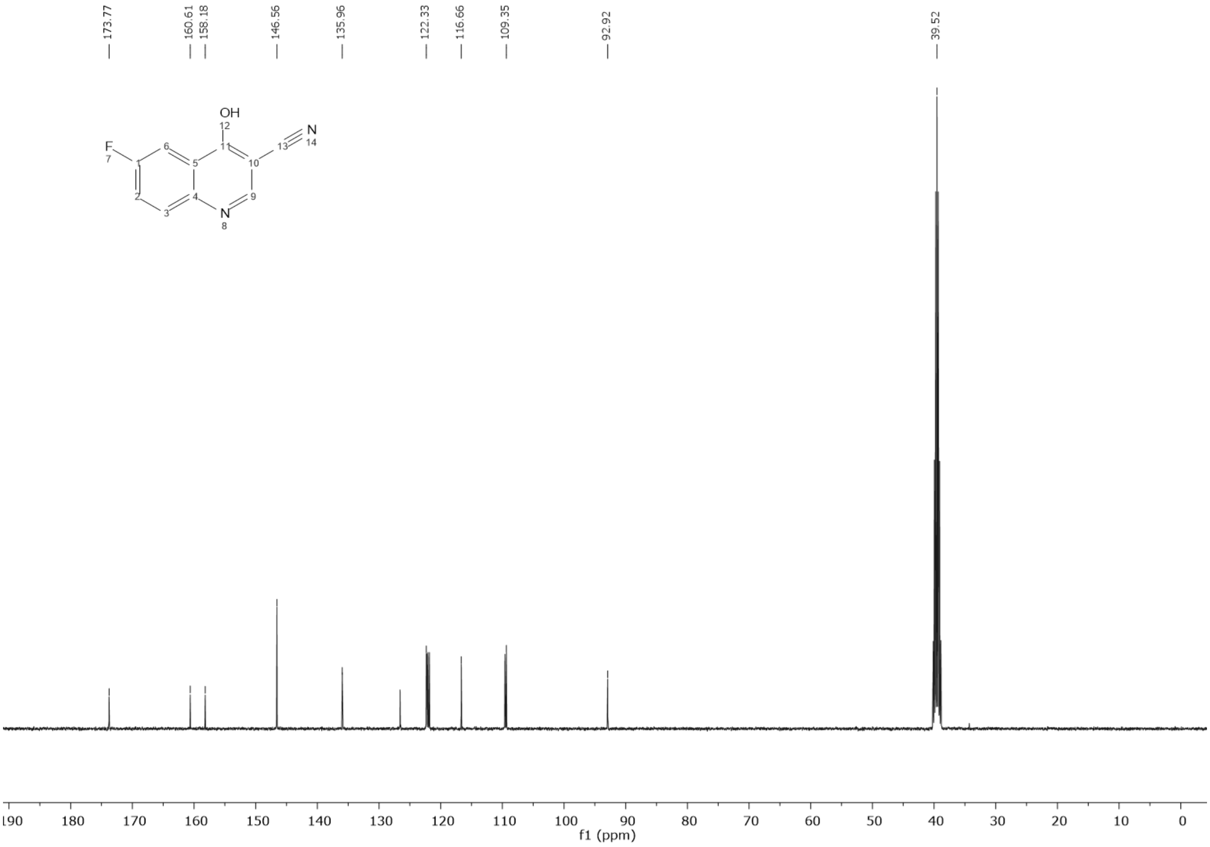
173.77
160.61
158.18
146.56
135.96
122.33
116.66
109.35
92.92
39.52
OH
F
N
N
190 180 170 160 150 140 130 120 110 100 90 80 70 60 50 40 30 20 10 0
f1 (ppm)

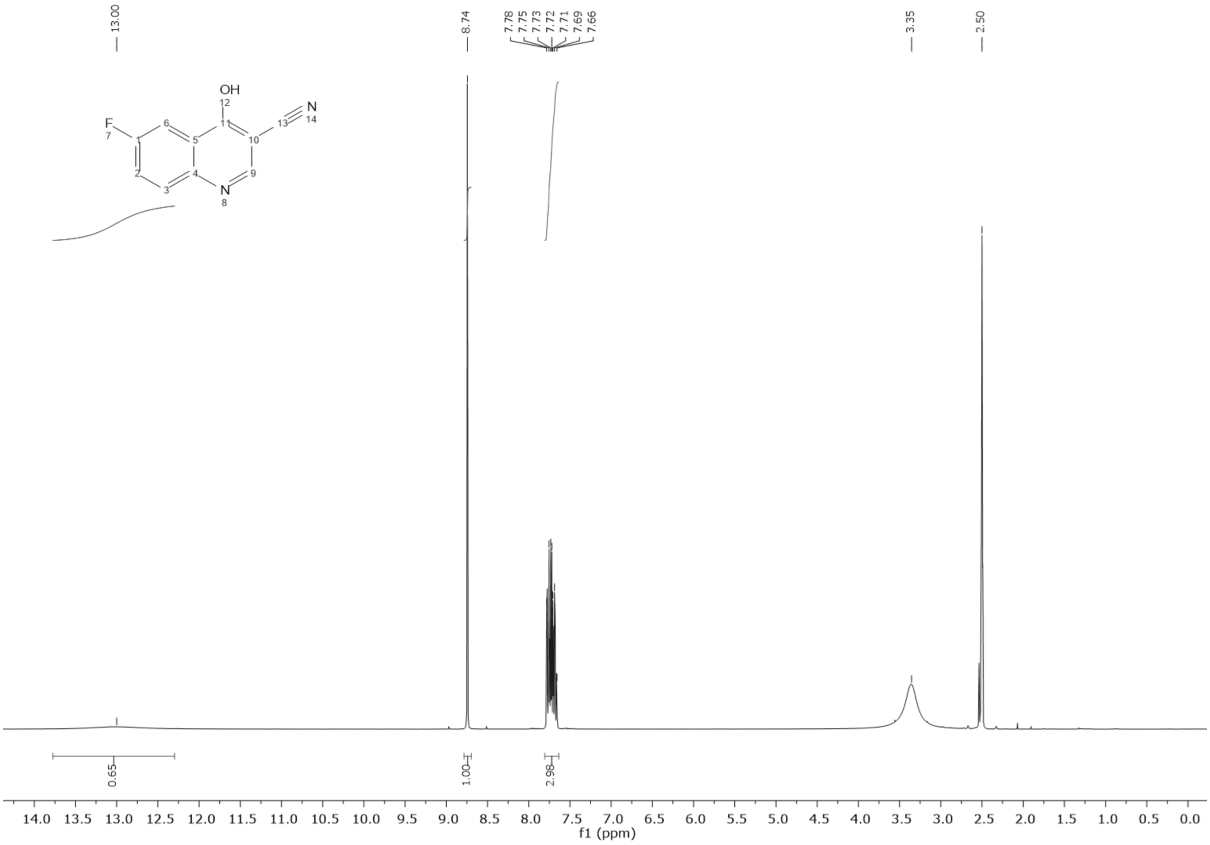
13.00
8.74
7.78
7.75
7.73
7.72
7.71
7.69
7.66
3.35
2.50
OH
F
N
N
0.65
1.00
2.98
14.0 13.5 13.0 12.5 12.0 11.5 11.0 10.5 10.0 9.5 9.0 8.5 8.0 7.5 7.0 6.5 6.0 5.5 5.0 4.5 4.0 3.5 3.0 2.5 2.0 1.5 1.0 0.5 0.0
f1 (ppm)

Sample Report

GOETHE UNIVERSITÄT FRANKFURT AM MAIN

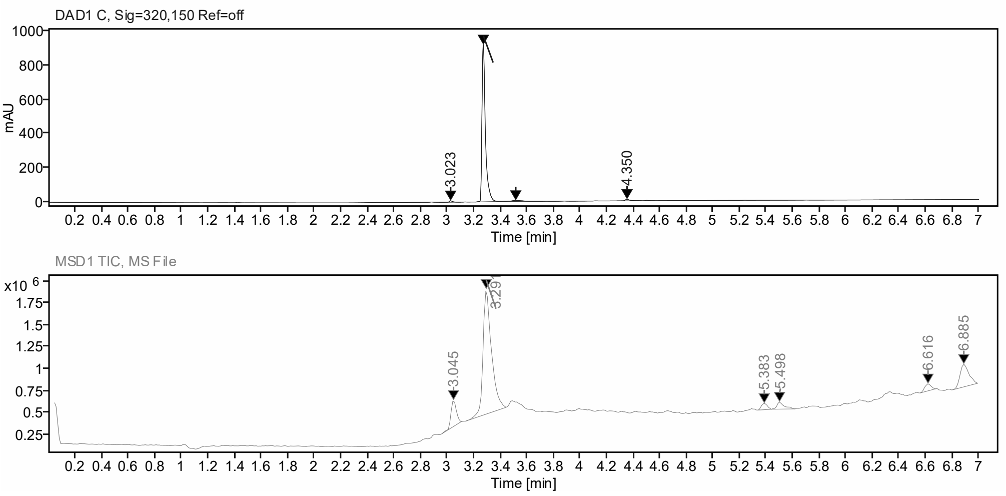

Sample Purity

Signal Description DAD1 C, Sig=320,150 Ref=off

Sample Name	Name	RT	Width	Area	Area%	Height
FGMM26re_dry		3.023	0.022	14.6501	0.84	8.8706
FGMM26re_dry		3.269	0.025	1667.5632	96.06	920.8936
FGMM26re_dry		3.513	0.087	32.4106	1.87	5.5200
FGMM26re_dry		4.350	0.032	21.3048	1.23	8.9128

Max Area% 96.062

UV Signal Purity>95% Pass

UV Apex Spectra

RT:

3.023

Sample Name:

FGMM26re_dry

Signal Name:

DAD1C

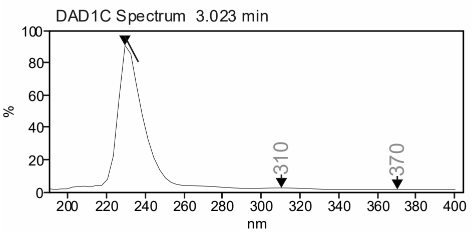

Sample Report

RT:

3.291

Sample Name:

FGMM26re_dry

Signal Name:

MSD1TIC

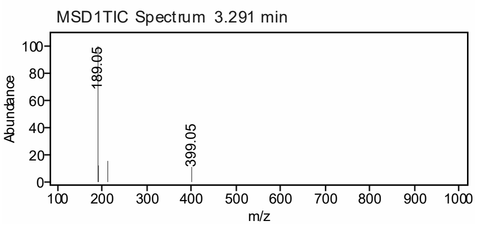

RT:

5.383

Sample Name:

FGMM26re_dry

Signal Name:

MSD1TIC

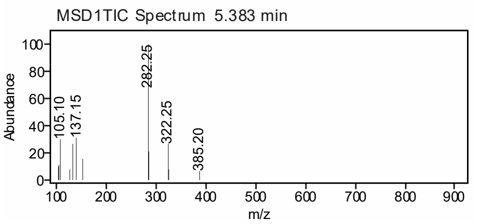

RT:

5.498

Sample Name:

FGMM26re_dry

Signal Name:

MSD1TIC

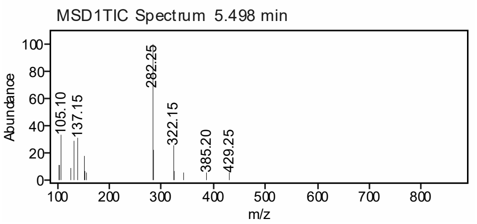

RT:

6.616

Sample Name:

FGMM26re_dry

Signal Name:

MSD1TIC

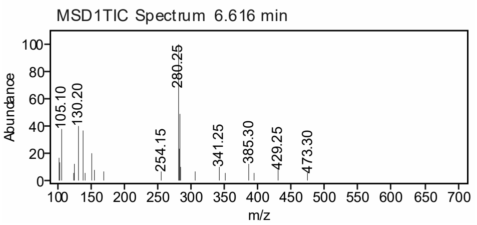

RT:

6.885

Sample Name:

FGMM26re_dry

Signal Name:

MSD1TIC

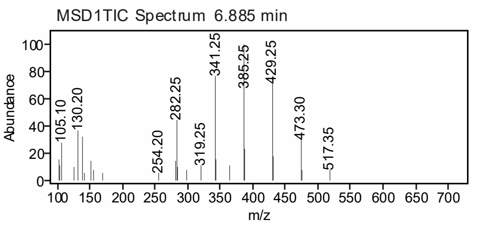

Compound 41

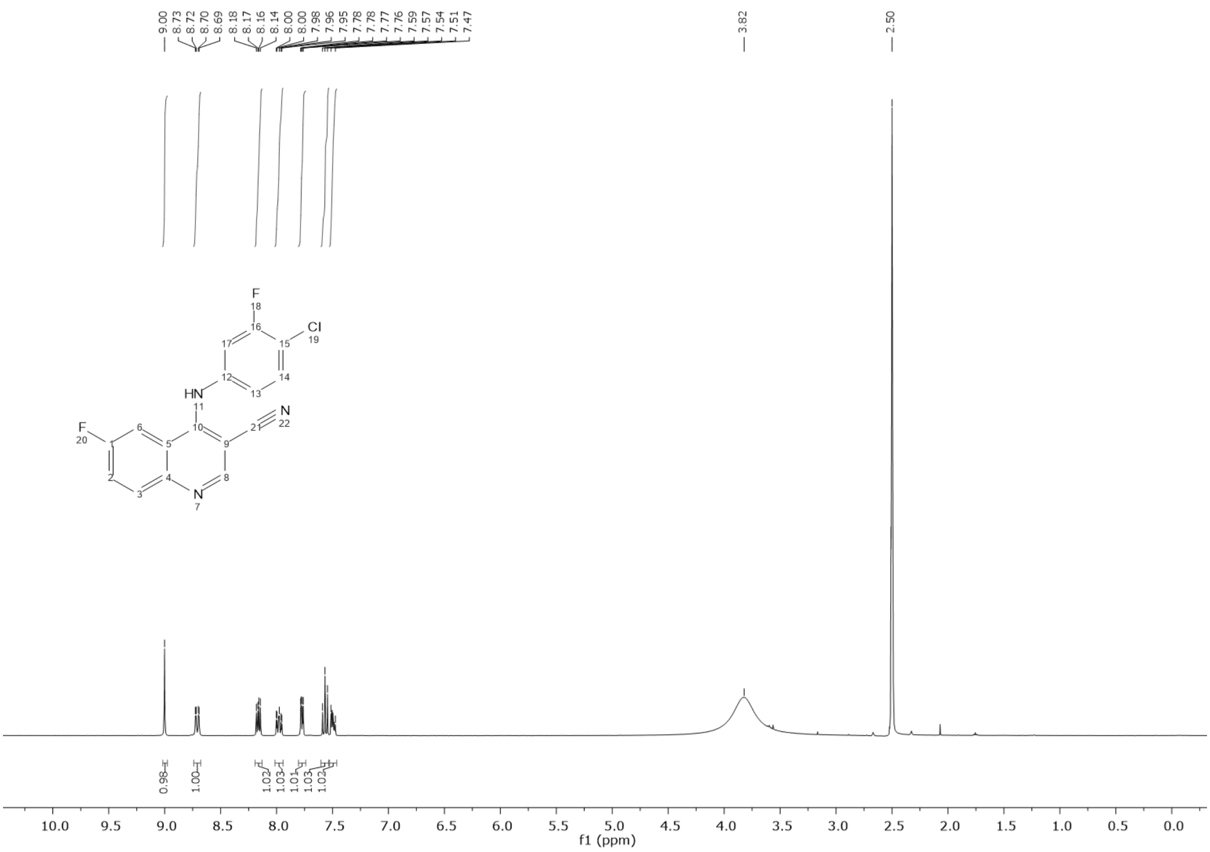
9.00 8.73 8.72 8.70 8.69 8.18 8.17 8.16 8.14 8.00 8.00 7.98 7.96 7.95 7.78 7.78 7.77 7.76 7.59 7.57 7.54 7.51 7.47
3.82
2.50
0.98 1.00 1.02 1.03 1.01 1.03 1.02
f1 (ppm)

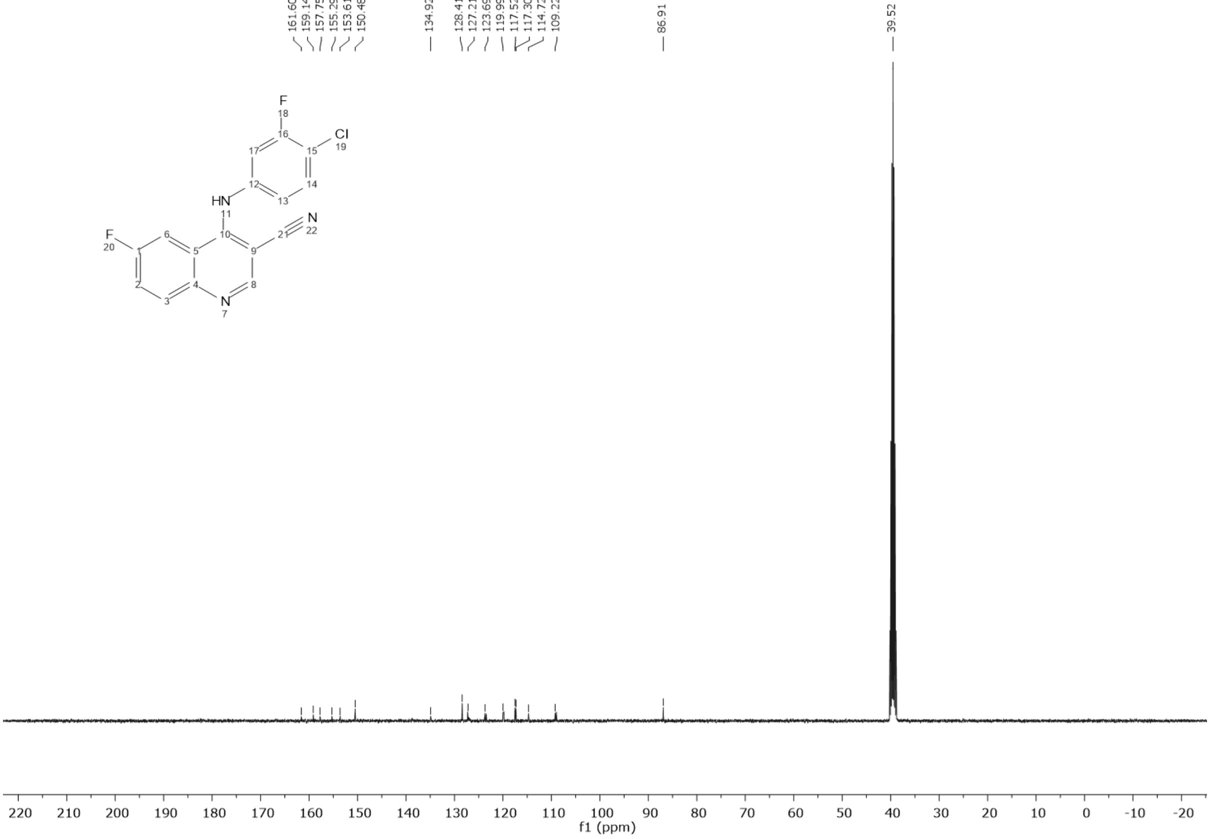
161.60 159.14 157.75 155.29 153.61 150.48
134.92
128.41 127.21 123.69 119.99 117.52 117.30 114.72 109.22
86.91
39.52
f1 (ppm)

Sample Report

GOETHE UNIVERSITÄT FRANKFURT AM MAIN

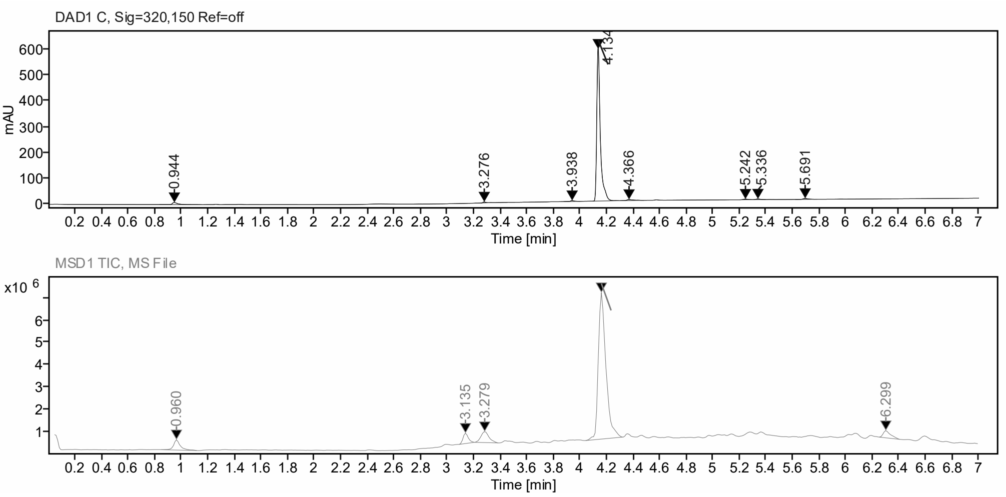

Sample Purity

Signal Description DAD1 C, Sig=320,150 Ref=off

Sample Name	Name	RT	Width	Area	Area%	Height
FGMM30_dry		0.944	0.038	25.2752	2.13	8.2166
FGMM30_dry		3.276	0.021	5.1292	0.43	3.8458
FGMM30_dry		3.938	0.026	6.8143	0.57	3.7013
FGMM30_dry		4.134	0.026	1113.4912	93.90	590.9642
FGMM30_dry		4.366	0.050	13.8344	1.17	4.2465
FGMM30_dry		5.242	0.039	5.7773	0.49	2.4315
FGMM30_dry		5.336	0.034	5.7065	0.48	2.5939
FGMM30_dry		5.691	0.037	9.7391	0.82	3.8104

Max Area% 93.905

UV Signal Purity>95% Fail

UV Apex Spectra

GOETHE UNIVERSITÄT FRANKFURT AM MAIN

RT:

3.135

Sample Name:

FGMM30_dry

Signal Name:

MSD1TIC

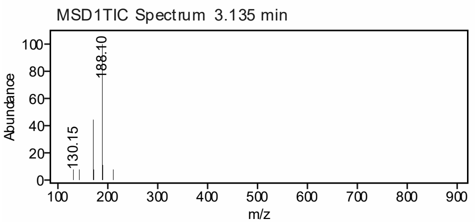

RT:

3.279

Sample Name:

FGMM30_dry

Signal Name:

MSD1TIC

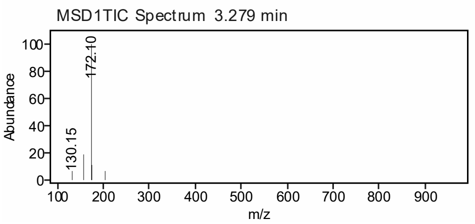

RT:

4.158

Sample Name:

FGMM30_dry

Signal Name:

MSD1TIC

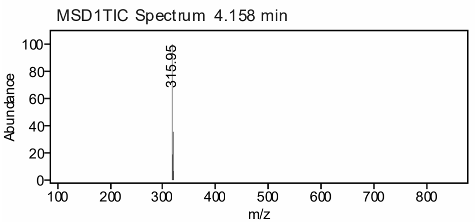

RT:

6.299

Sample Name:

FGMM30_dry

Signal Name:

MSD1TIC

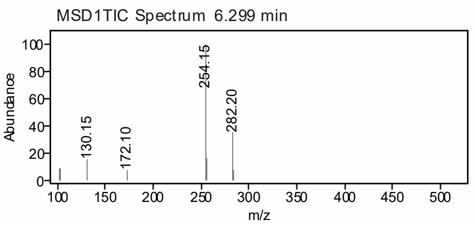

Compound 64

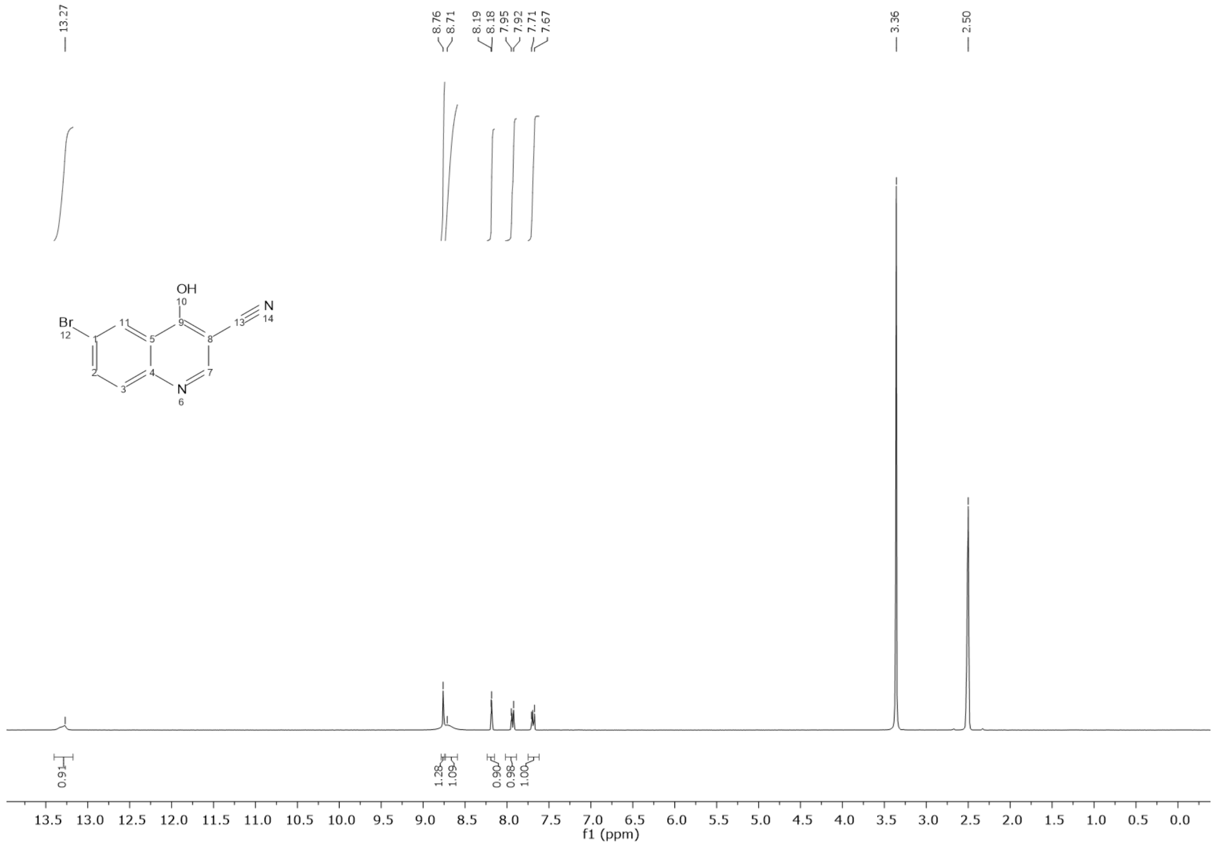

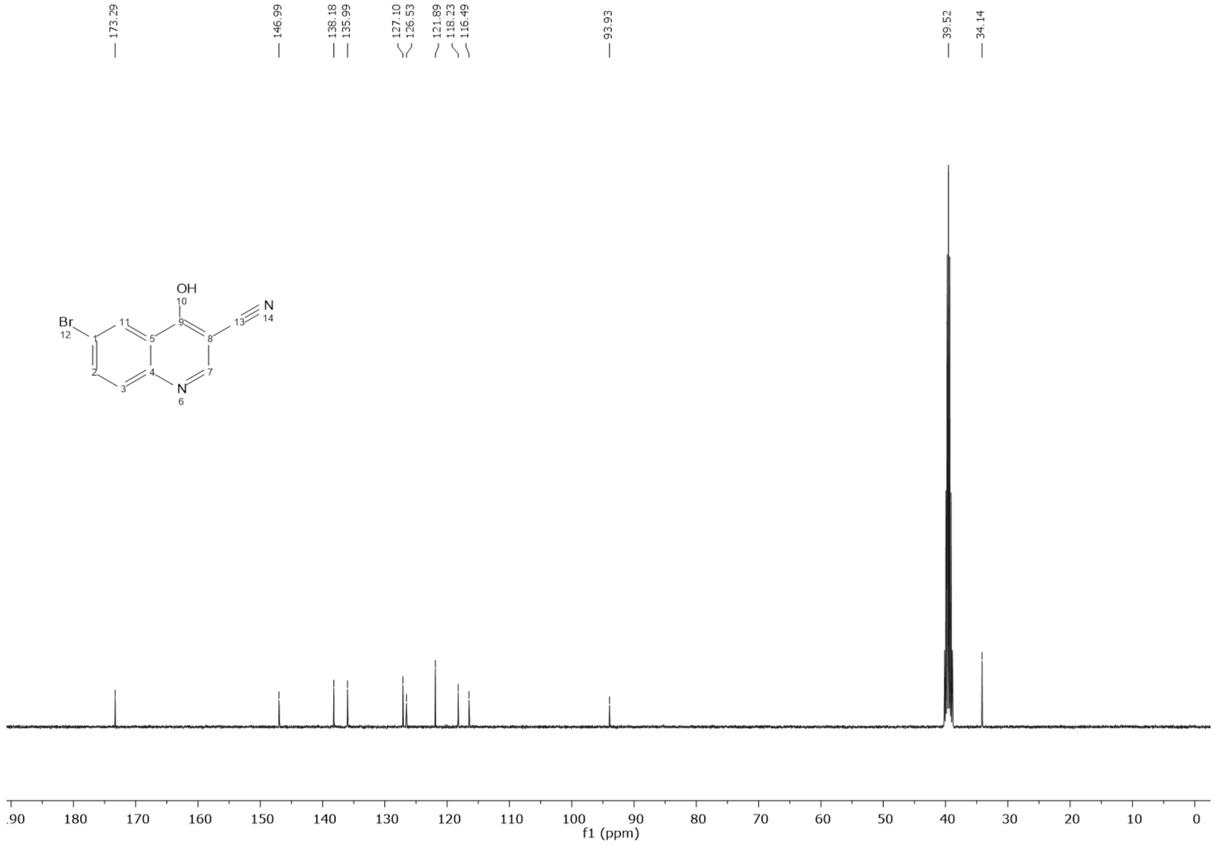

Sample Report

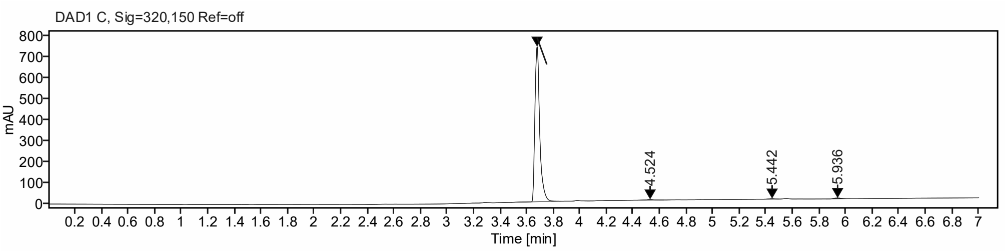

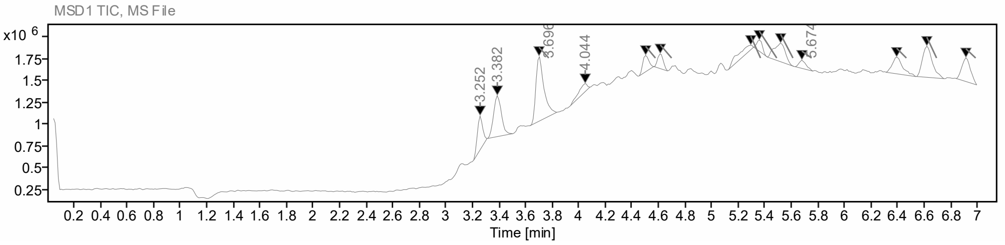

Sample Purity

Signal Description DAD1 C, Sig=320,150 Ref=off

Sample Name	Name	RT	Width	Area	Area%	Height
FGMM56_dry		3.674	0.037	1855.5162	98.12	741.2307
FGMM56_dry		4.524	0.057	13.0019	0.69	3.0177
FGMM56_dry		5.442	0.054	8.1504	0.43	2.5223
FGMM56_dry		5.936	0.062	14.3205	0.76	3.6927

Max Area% 98.124

UV Signal Purity>95% Pass

UV Apex Spectra

RT:

3.674

Sample Name:

FGMM56_dry

Signal Name:

DAD1C

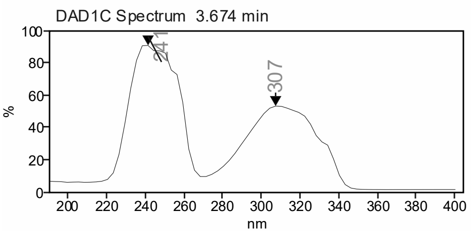

RT:

3.382

Sample Name:

FGMM56_dry

Signal Name:

MSD1TIC

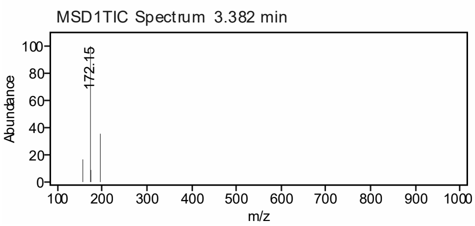

RT:

3.696

Sample Name:

FGMM56_dry

Signal Name:

MSD1TIC

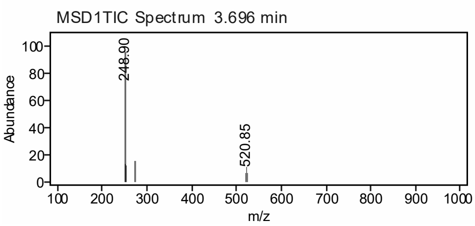

RT:

4.044

Sample Name:

FGMM56_dry

Signal Name:

MSD1TIC

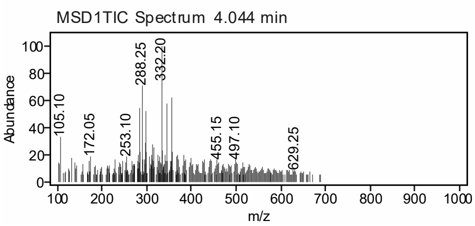

RT:

4.498

Sample Name:

FGMM56_dry

Signal Name:

MSD1TIC

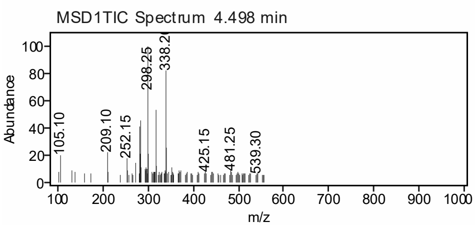

RT:

4.609

Sample Name:

FGMM56_dry

Signal Name:

MSD1TIC

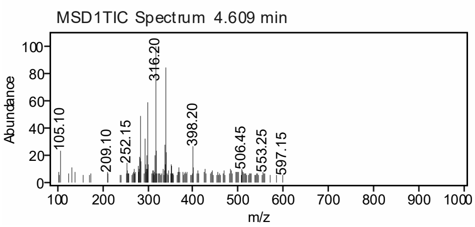

Compound 42

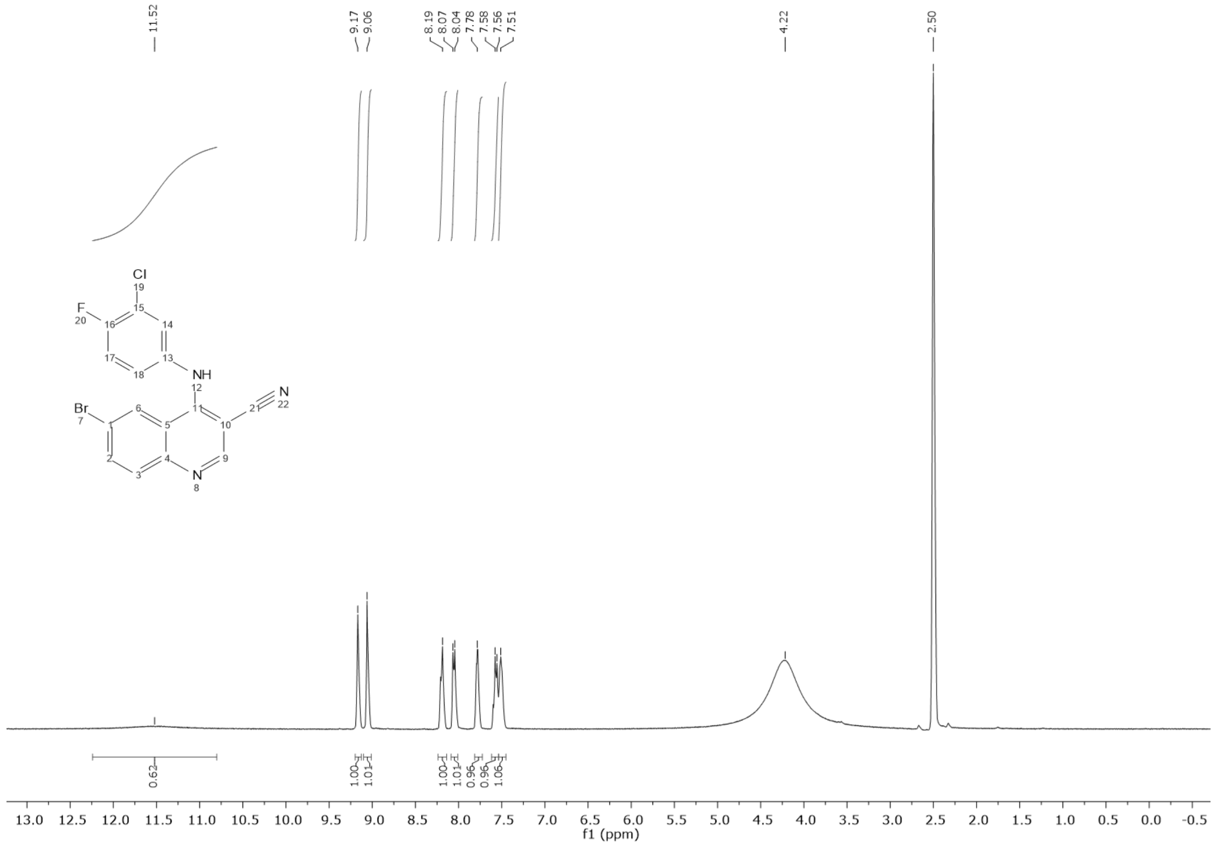
11.52
9.17
9.06
8.19
8.07
8.04
7.78
7.58
7.56
7.51
4.22
2.50
Cl
F
NH
Br
N
0.63
1.00
1.01
1.00
1.01
0.95
0.96
1.06
f1 (ppm)

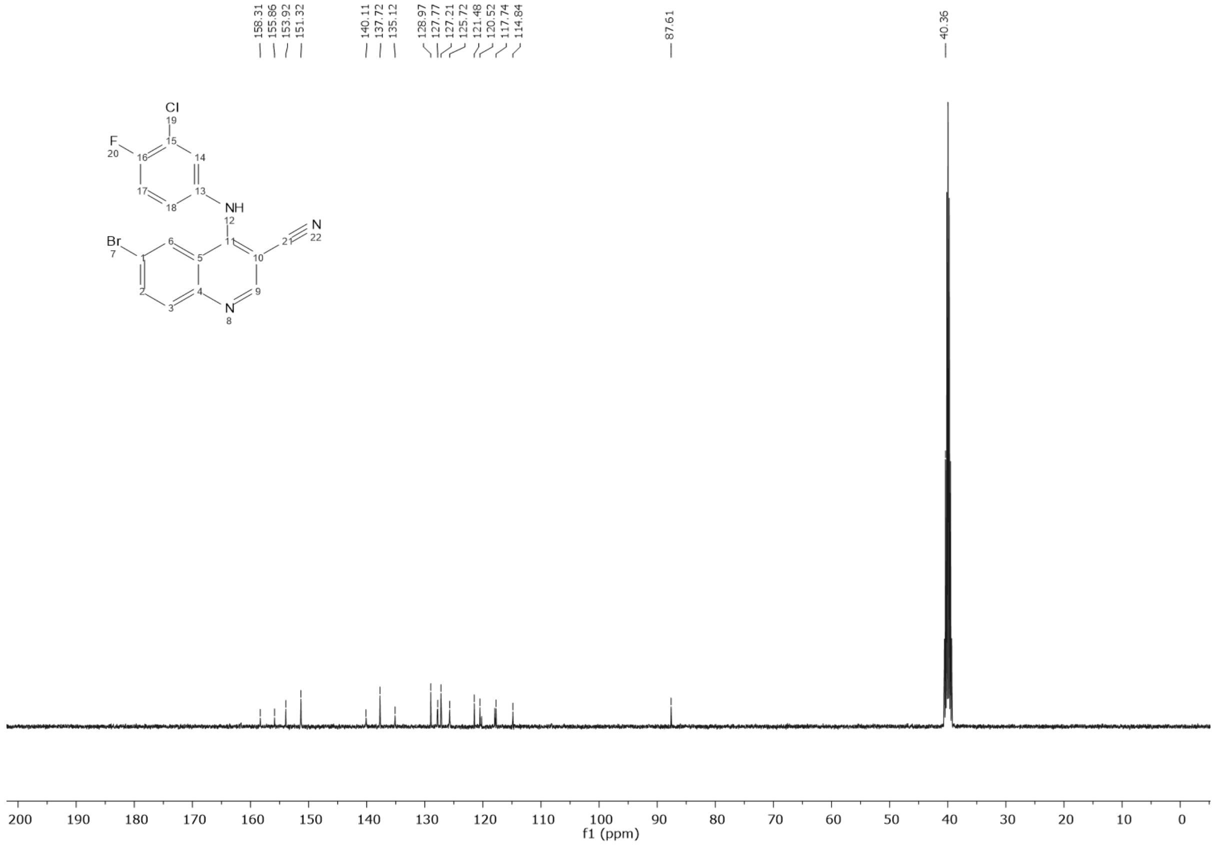
158.31
155.86
153.92
151.32
140.11
137.72
135.12
128.97
127.77
127.21
125.72
121.48
120.52
117.74
114.84
87.61
40.36
Cl
F
NH
Br
N
f1 (ppm)

GOETHE UNIVERSITÄT FRANKFURT AM MAIN

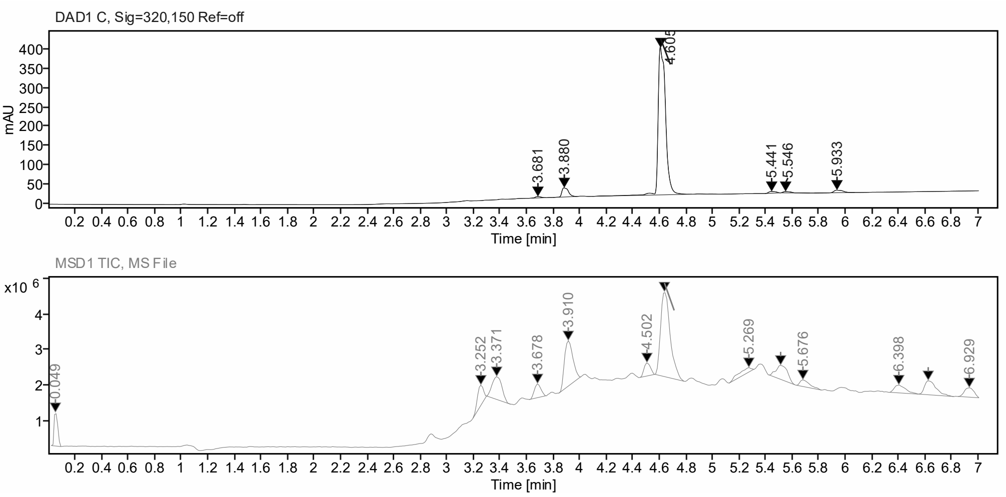

Sample Purity

Signal Description DAD1 C, Sig=320,150 Ref=off

Sample Name	Name	RT	Width	Area	Area%	Height
FGMM60_on		3.681	0.052	10.9420	0.67	3.4723
FGMM60_on		3.880	0.055	81.1126	4.95	23.7141
FGMM60_on		4.605	0.060	1483.0935	90.48	380.9886
FGMM60_on		5.441	0.071	17.1060	1.04	4.4311
FGMM60_on		5.546	0.068	15.3839	0.94	3.4119
FGMM60_on		5.933	0.079	31.5823	1.93	6.5990

Max Area% 90.476

UV Signal Purity>95% Fail

UV Apex Spectra

Sample Report

GOETHE UNIVERSITÄT FRANKFURT AM MAIN

RT:

3.678

Sample Name:

FGMM60_on

Signal Name:

MSD1TIC

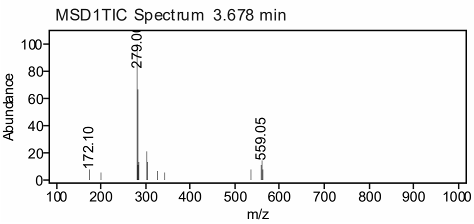

RT:

3.910

Sample Name:

FGMM60_on

Signal Name:

MSD1TIC

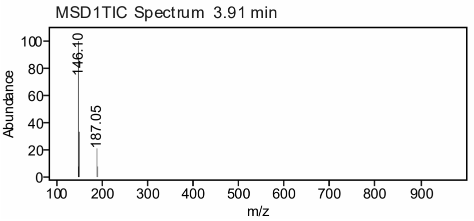

RT:

4.502

Sample Name:

FGMM60_on

Signal Name:

MSD1TIC

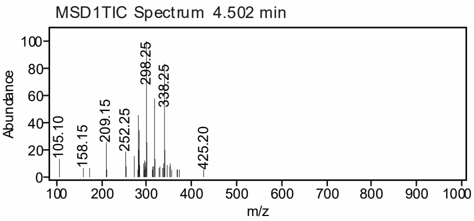

RT:

4.634

Sample Name:

FGMM60_on

Signal Name:

MSD1TIC

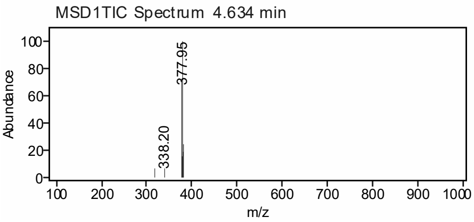

RT:

5.269

Sample Name:

FGMM60_on

Signal Name:

MSD1TIC

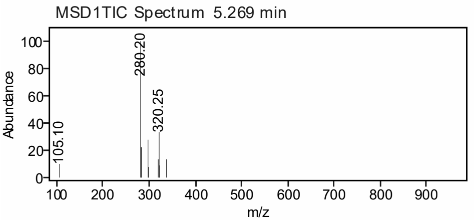

Compound 65

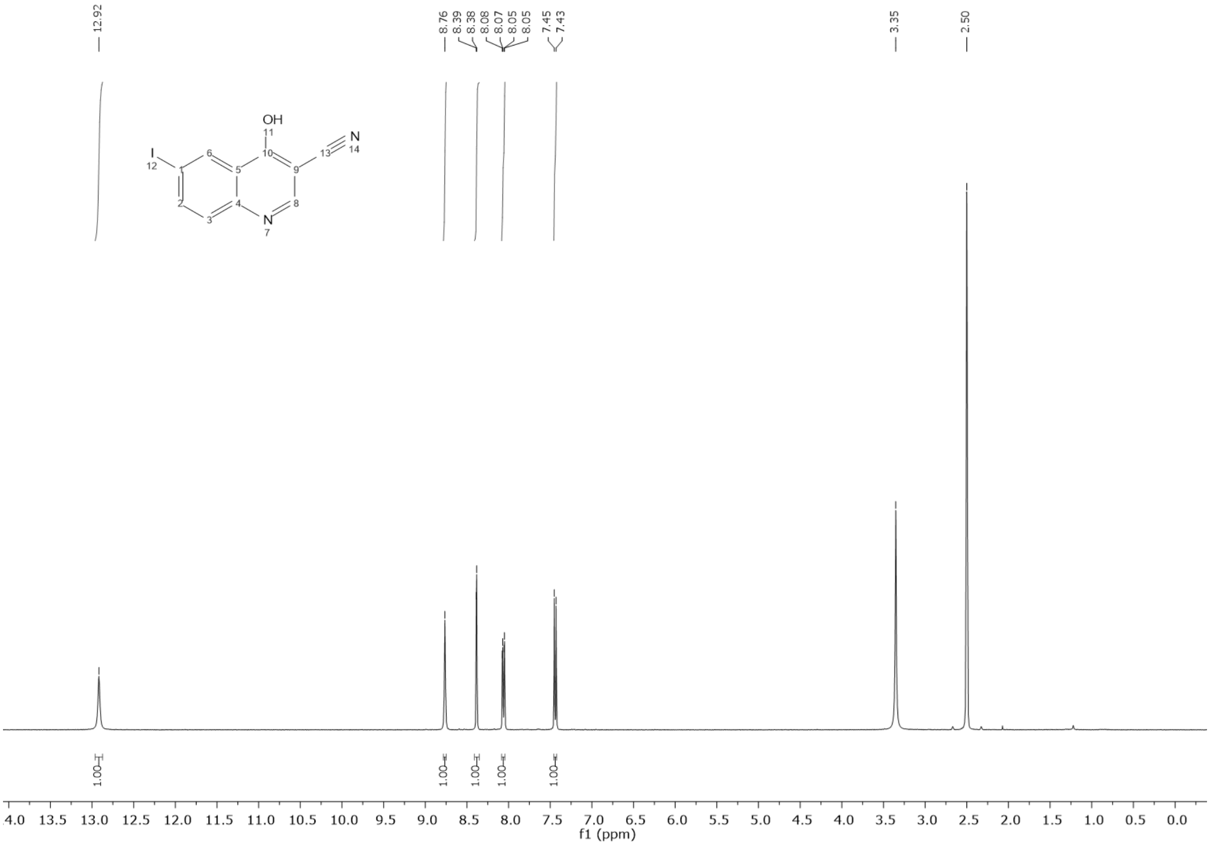
12.92
8.76
8.39
8.38
8.08
8.07
8.05
8.05
7.45
7.43
3.35
2.50
OH
N
I
N
1.00
1.00
1.00
1.00
1.00
f1 (ppm)

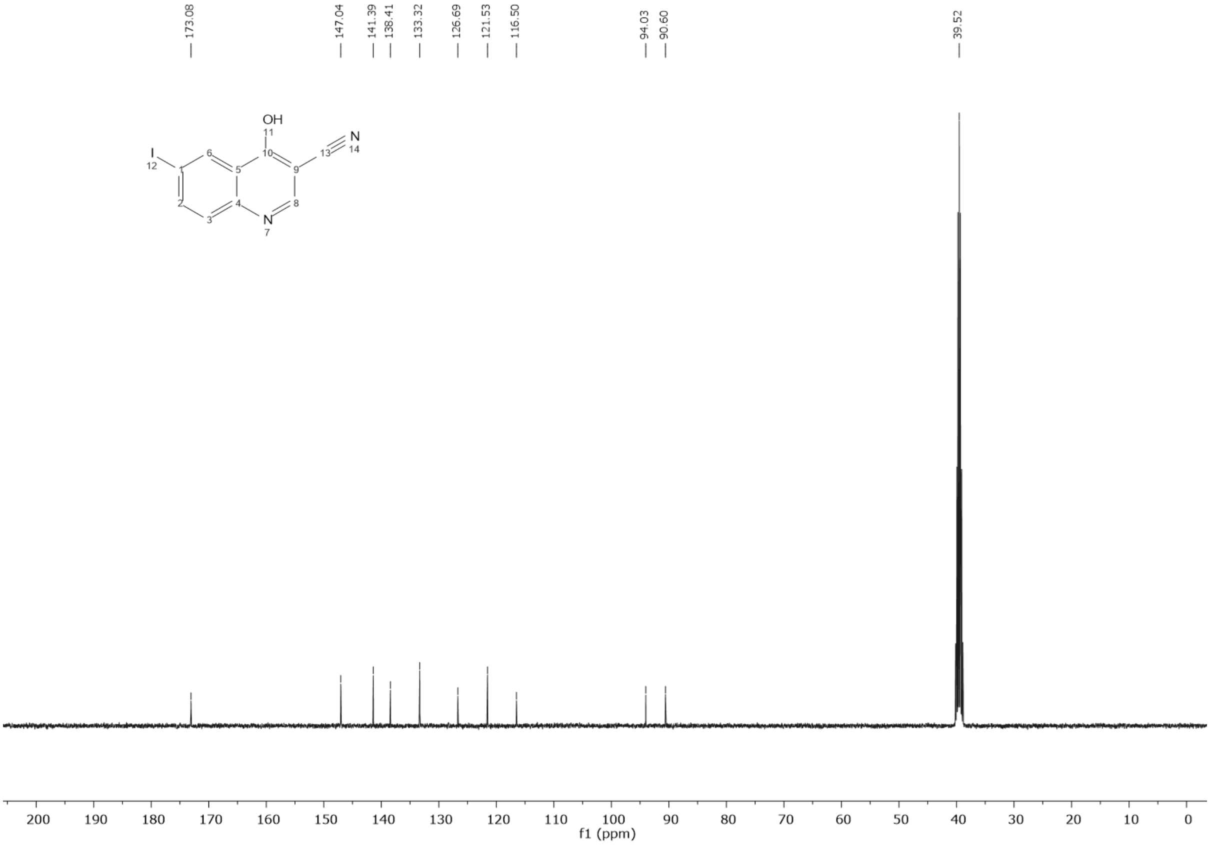
173.08
147.04
141.39
138.41
133.32
126.69
121.53
116.50
94.03
90.60
39.52
OH
N
I
N
f1 (ppm)

Sample Report

GOETHE UNIVERSITÄT FRANKFURT AM MAIN

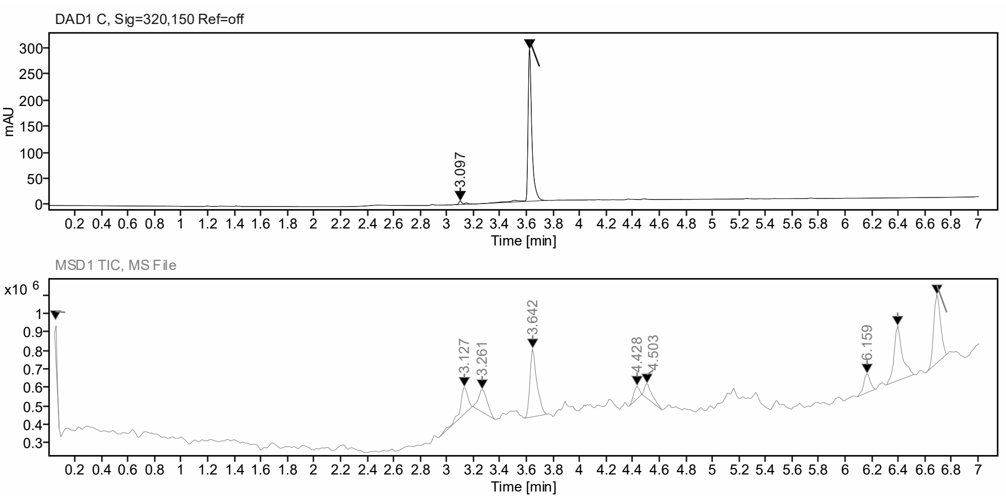

Sample Purity

Signal Description	DAD1 C, Sig=320,150 Ref=off

Sample Name	Name	RT	Width	Area	Area%	Height
FGMM122_dry		3.097	0.023	15.9014	2.60	6.7756
FGMM122_dry		3.619	0.028	594.8845	97.40	291.6194

Max Area%	97.397
UV Signal Purity>95%	Pass

UV Apex Spectra

RT:

3.097

Sample Name:

FGMM122_dry

Signal Name:

DAD1C

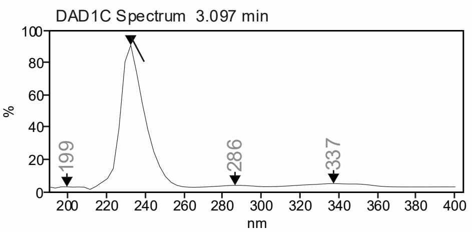

RT:

3.642

Sample Name:

FGMM122_dry

Signal Name:

MSD1TIC

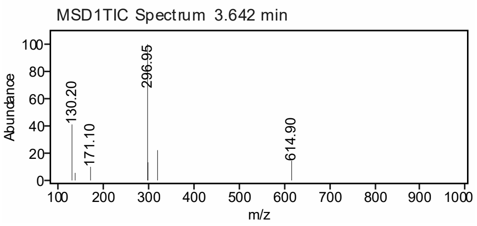

RT:

4.428

Sample Name:

FGMM122_dry

Signal Name:

MSD1TIC

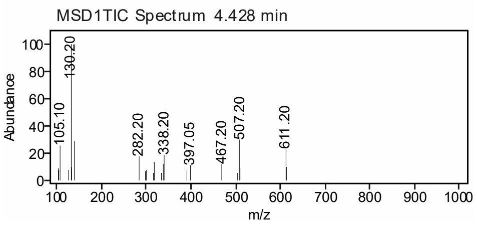

RT:

4.503

Sample Name:

FGMM122_dry

Signal Name:

MSD1TIC

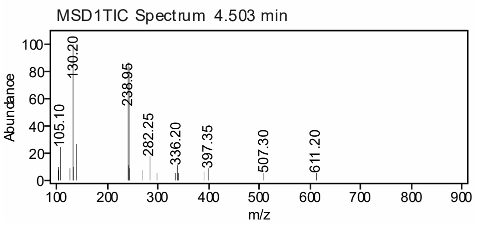

RT:

6.159

Sample Name:

FGMM122_dry

Signal Name:

MSD1TIC

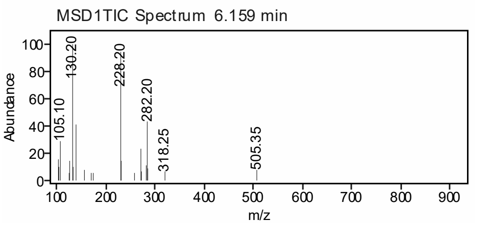

RT:

6.389

Sample Name:

FGMM122_dry

Signal Name:

MSD1TIC

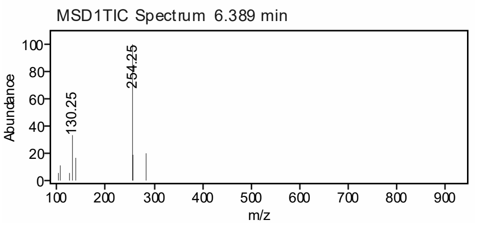

Compound 43

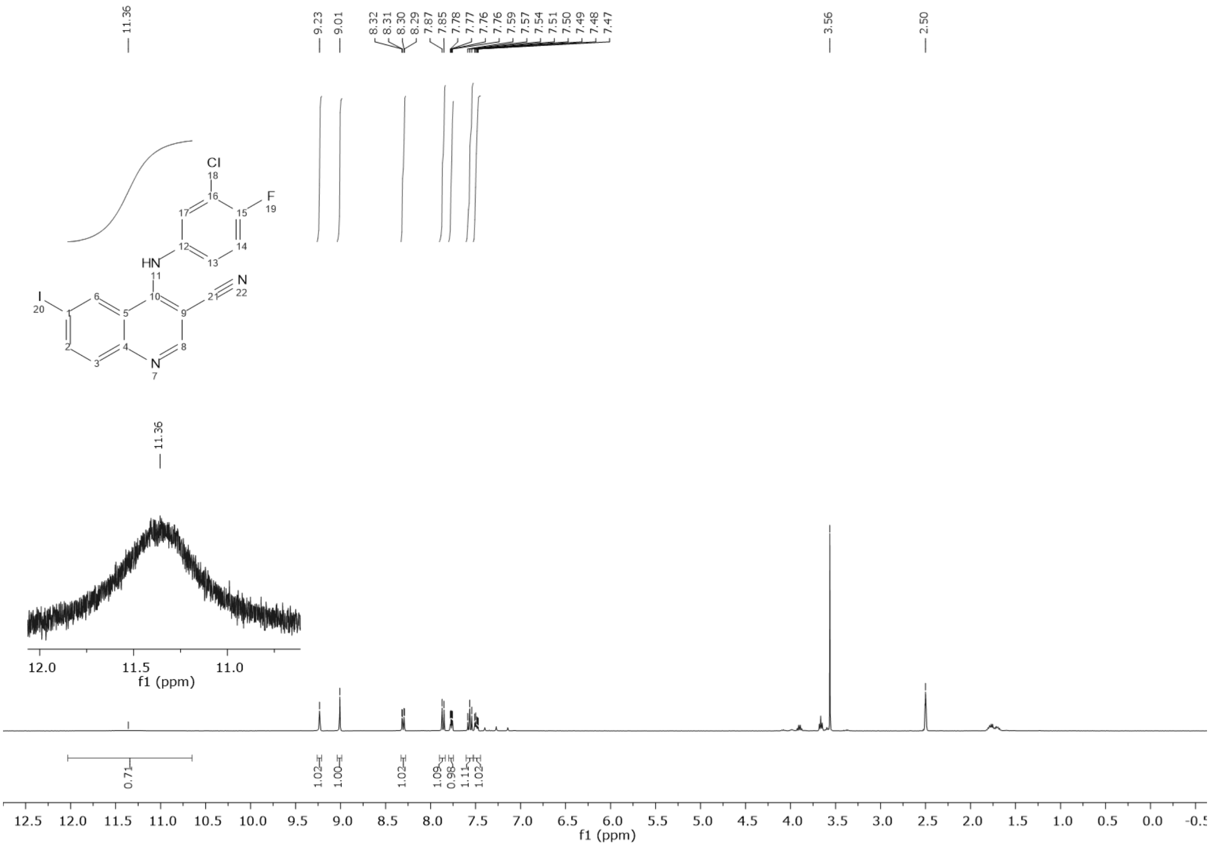

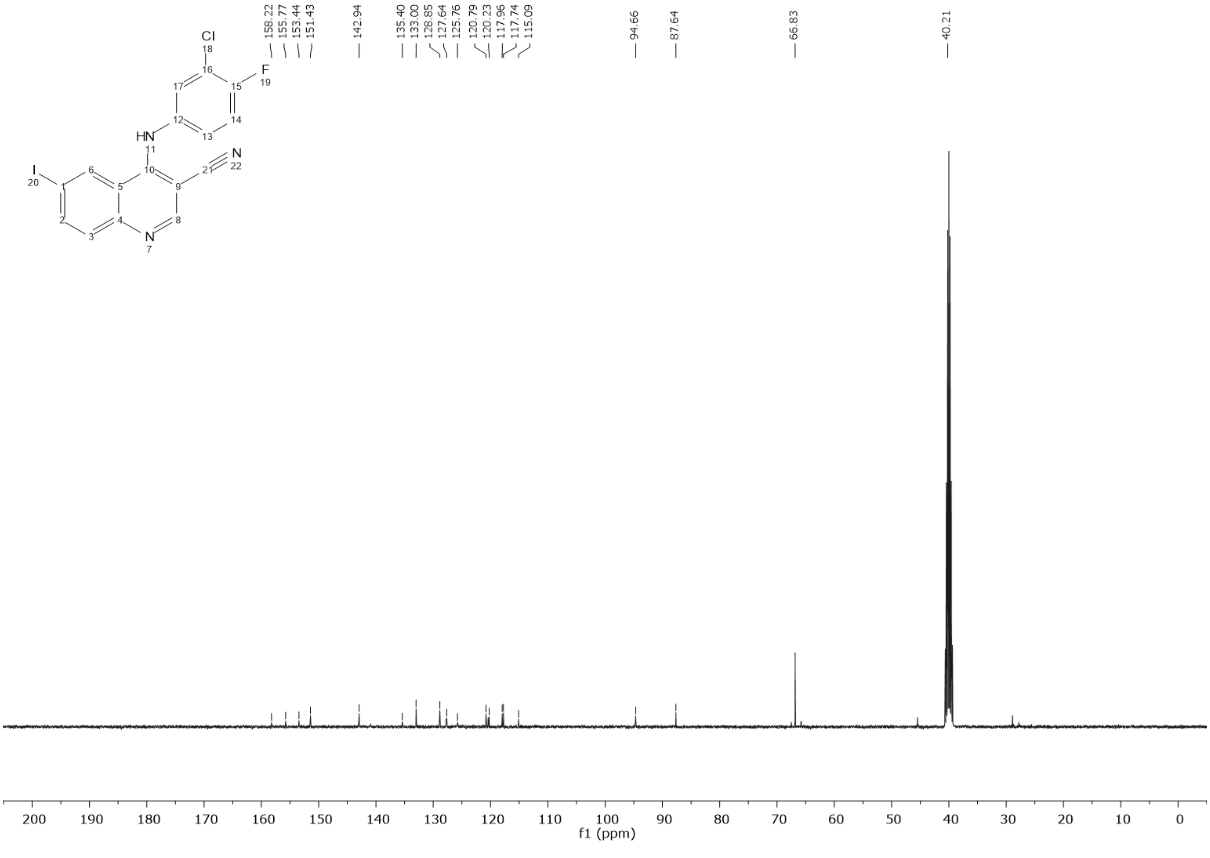

Sample Report

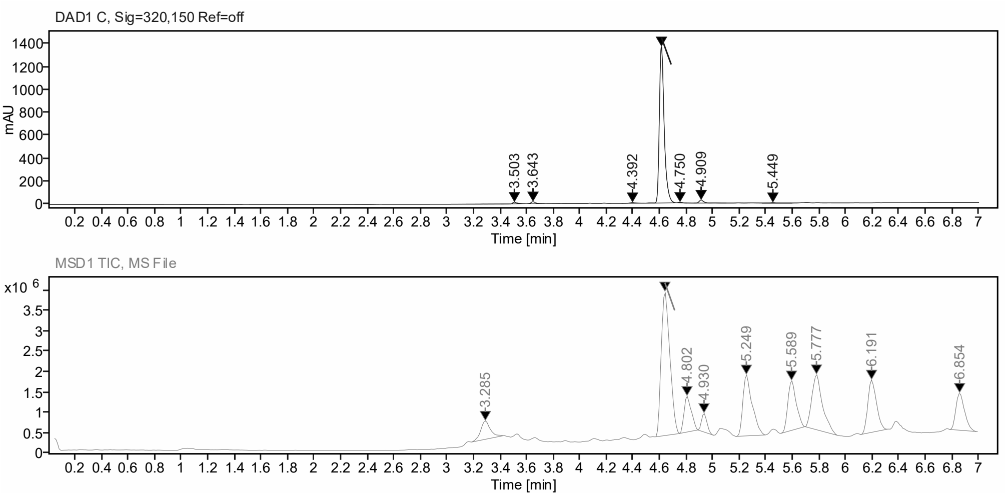

Sample Purity

Signal Description	DAD1 C, Sig=320,150 Ref=off

Sample Name	Name	RT	Width	Area	Area%	Height
FGMM133_F7		3.503	0.033	33.2770	0.91	14.4377
FGMM133_F7		3.643	0.032	39.4577	1.08	17.6363
FGMM133_F7		4.392	0.039	18.7562	0.52	6.7893
FGMM133_F7		4.610	0.037	3457.8435	95.01	1366.6313
FGMM133_F7		4.750	0.030	10.4448	0.29	5.3964
FGMM133_F7		4.909	0.034	70.0267	1.92	26.3559
FGMM133_F7		5.449	0.040	9.4615	0.26	2.9928

Max Area%	95.015
UV Signal Purity>95%	Pass

UV Apex Spectra

GOETHE UNIVERSITÄT FRANKFURT AM MAIN

RT:

4.909

Sample Name:

FGMM133_F7

Signal Name:

DAD1C

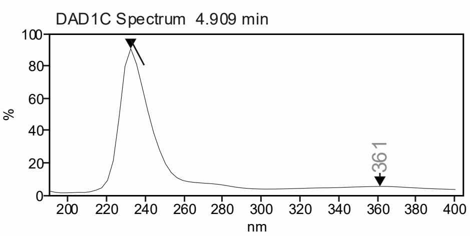

RT:

5.449

Sample Name:

FGMM133_F7

Signal Name:

DAD1C

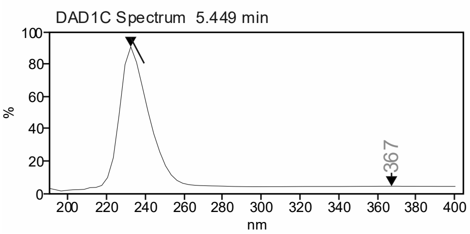

MSD Apex Spectra

RT:

3.285

Sample Name:

FGMM133_F7

Signal Name:

MSD1TIC

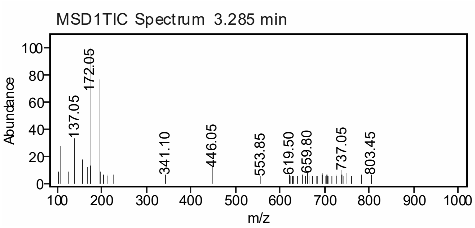

RT:

4.636

Sample Name:

FGMM133_F7

Signal Name:

MSD1TIC

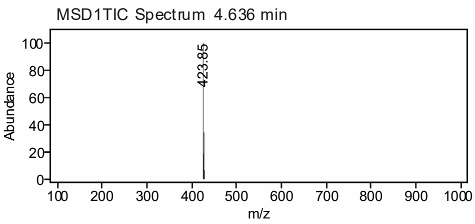

Compound 66

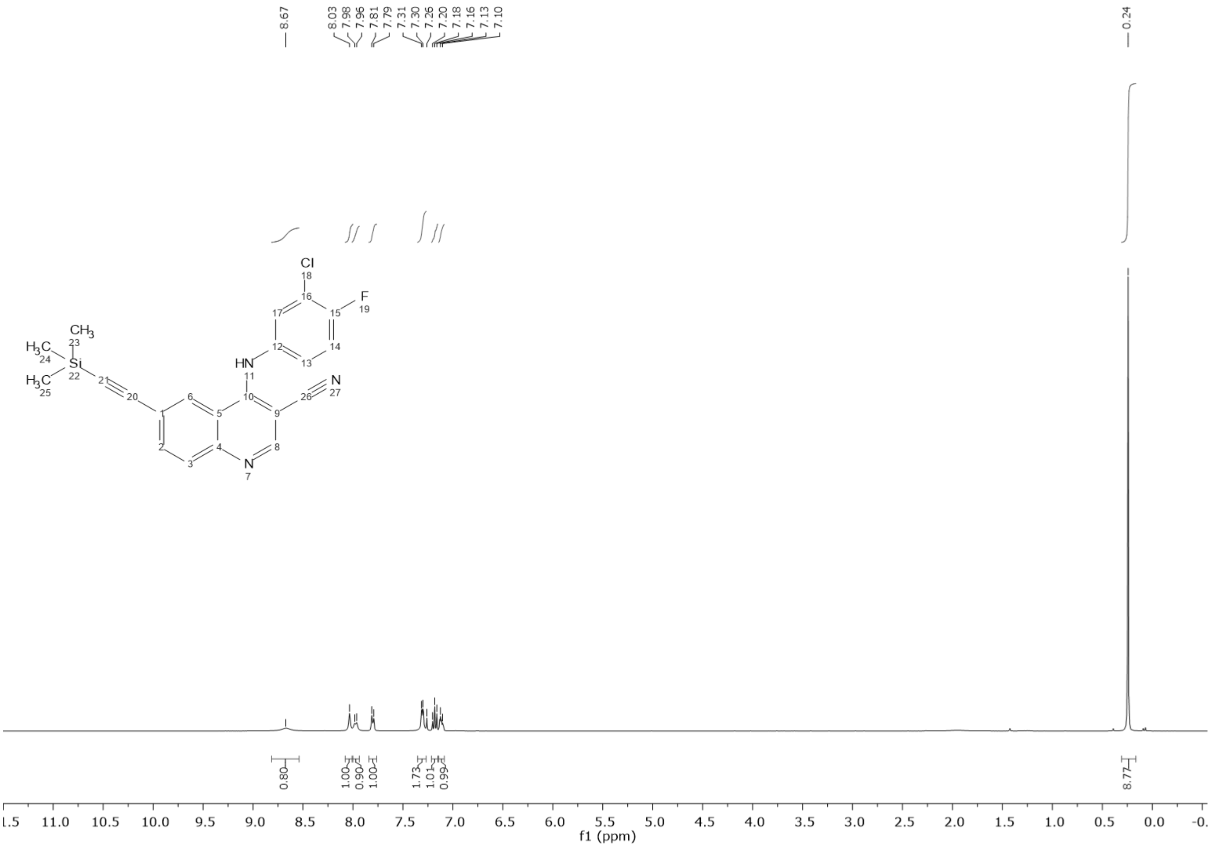

8.67
8.03
7.98
7.96
7.81
7.79
7.31
7.30
7.26
7.20
7.18
7.16
7.13
7.10
0.24
0.80
1.00
0.90
1.00
1.73
1.01
0.99
8.77
f1 (ppm)

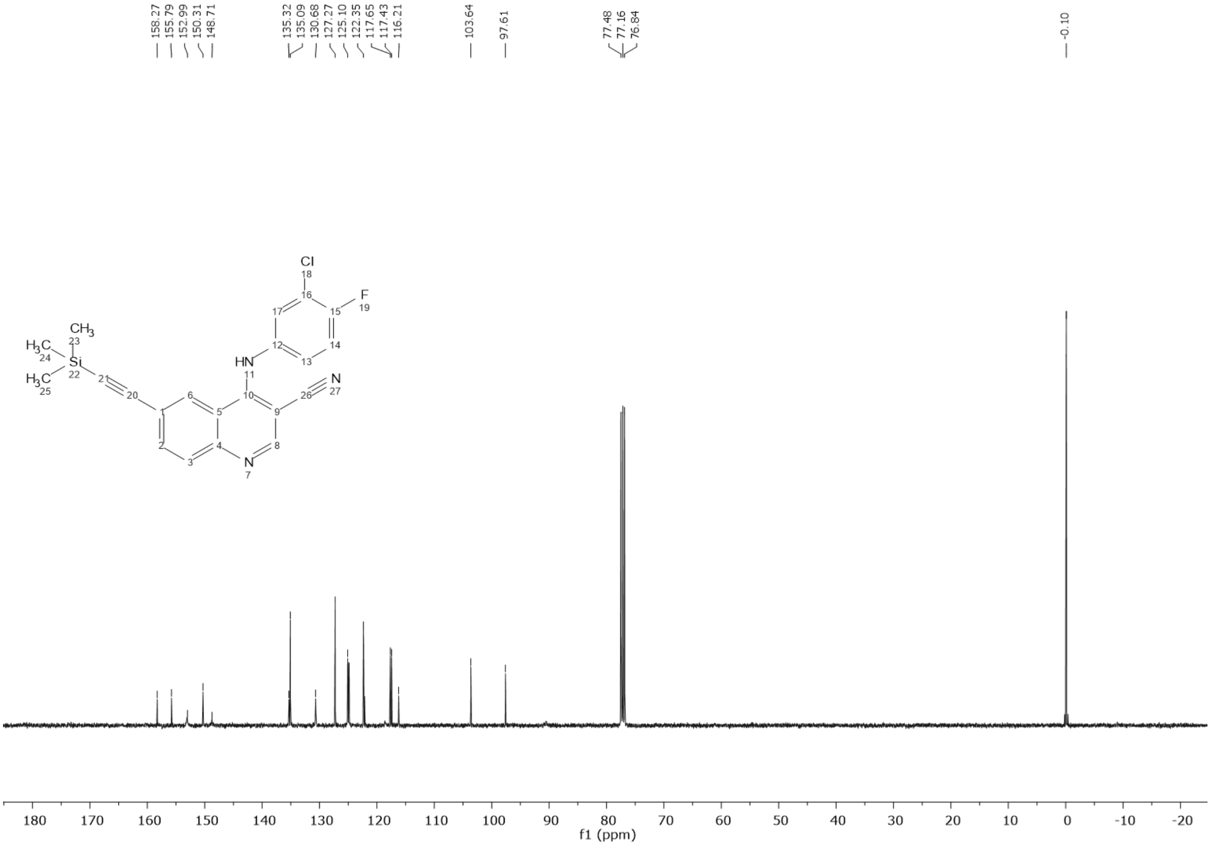

158.27
155.79
152.99
150.31
148.71
135.32
135.09
130.68
127.27
125.10
122.35
117.65
117.43
116.21
103.64
97.61
77.48
77.16
76.84
-0.10
f1 (ppm)

Sample Report

GOETHE UNIVERSITÄT FRANKFURT AM MAIN

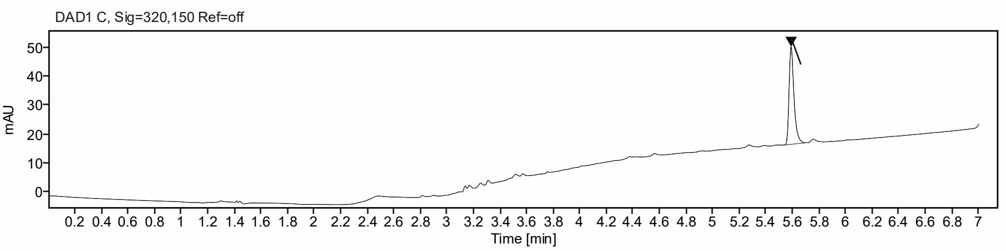

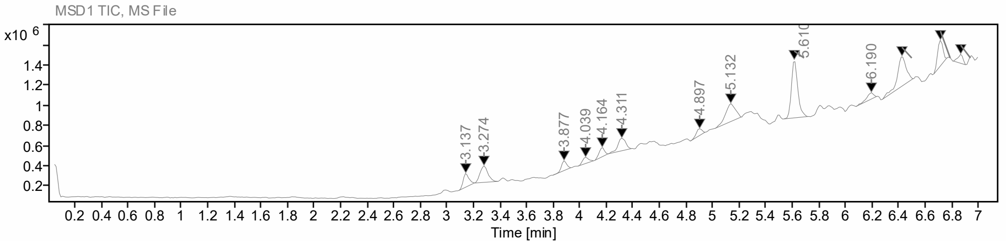

Sample Purity

Signal Description DAD1 C, Sig=320,150 Ref=off

Sample Name	Name	RT	Width	Area	Area%	Height
FGMM137_dry		5.586	0.038	86.8320	100.00	33.6661

Max Area% 100.000

UV Signal Purity>95% Pass

UV Apex Spectra

RT:

5.586

Sample Name:

FGMM137_dry

Signal Name:

DAD1C

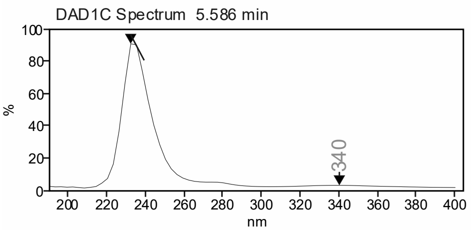

GOETHE UNIVERSITÄT FRANKFURT AM MAIN

RT:

4.164

Sample Name:

FGMM137_dry

Signal Name:

MSD1TIC

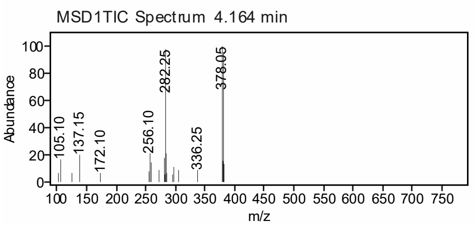

RT:

4.311

Sample Name:

FGMM137_dry

Signal Name:

MSD1TIC

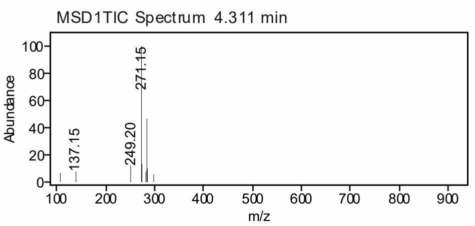

RT:

4.897

Sample Name:

FGMM137_dry

Signal Name:

MSD1TIC

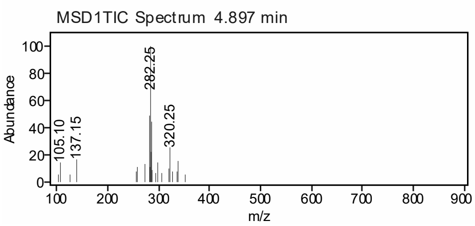

RT:

5.132

Sample Name:

FGMM137_dry

Signal Name:

MSD1TIC

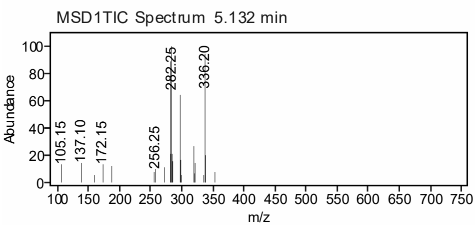

RT:

5.610

Sample Name:

FGMM137_dry

Signal Name:

MSD1TIC

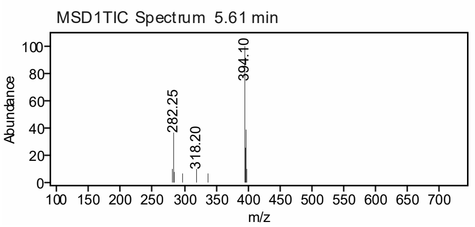

Compound 44

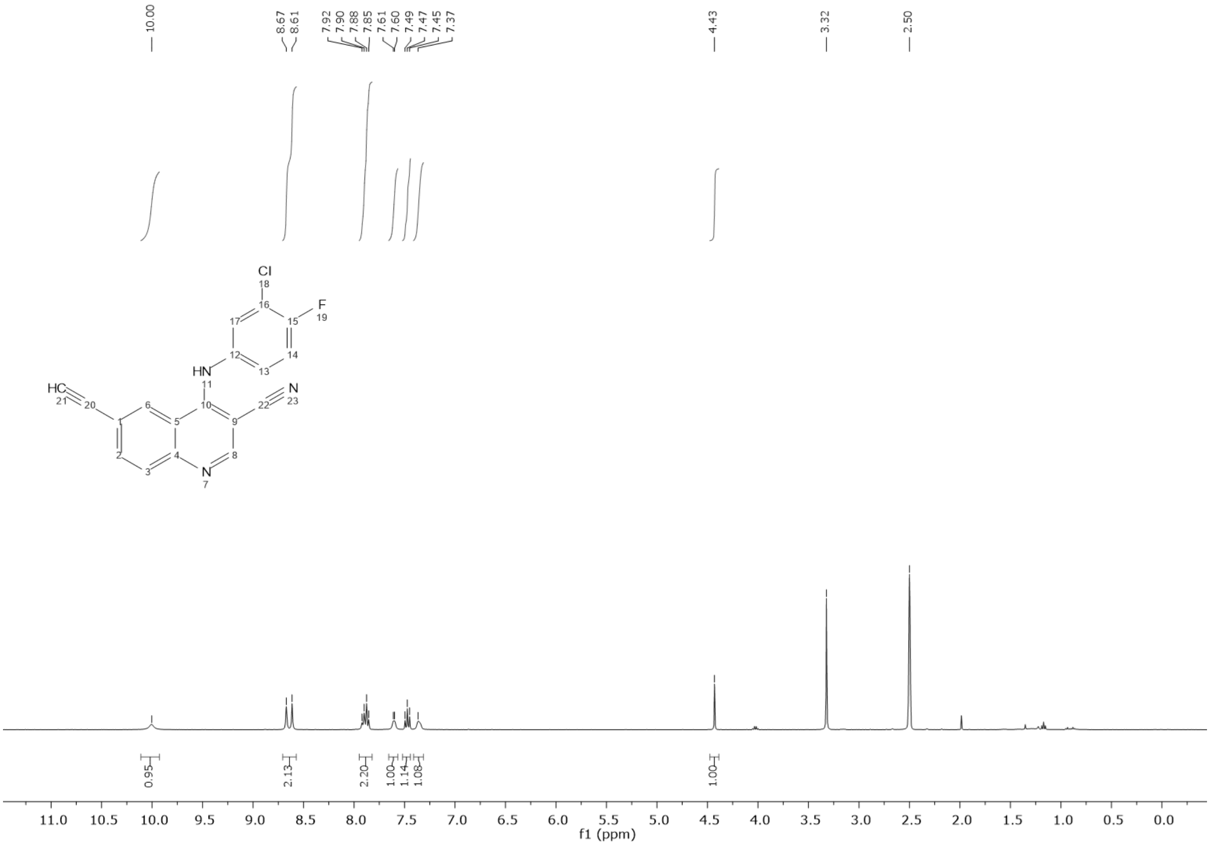

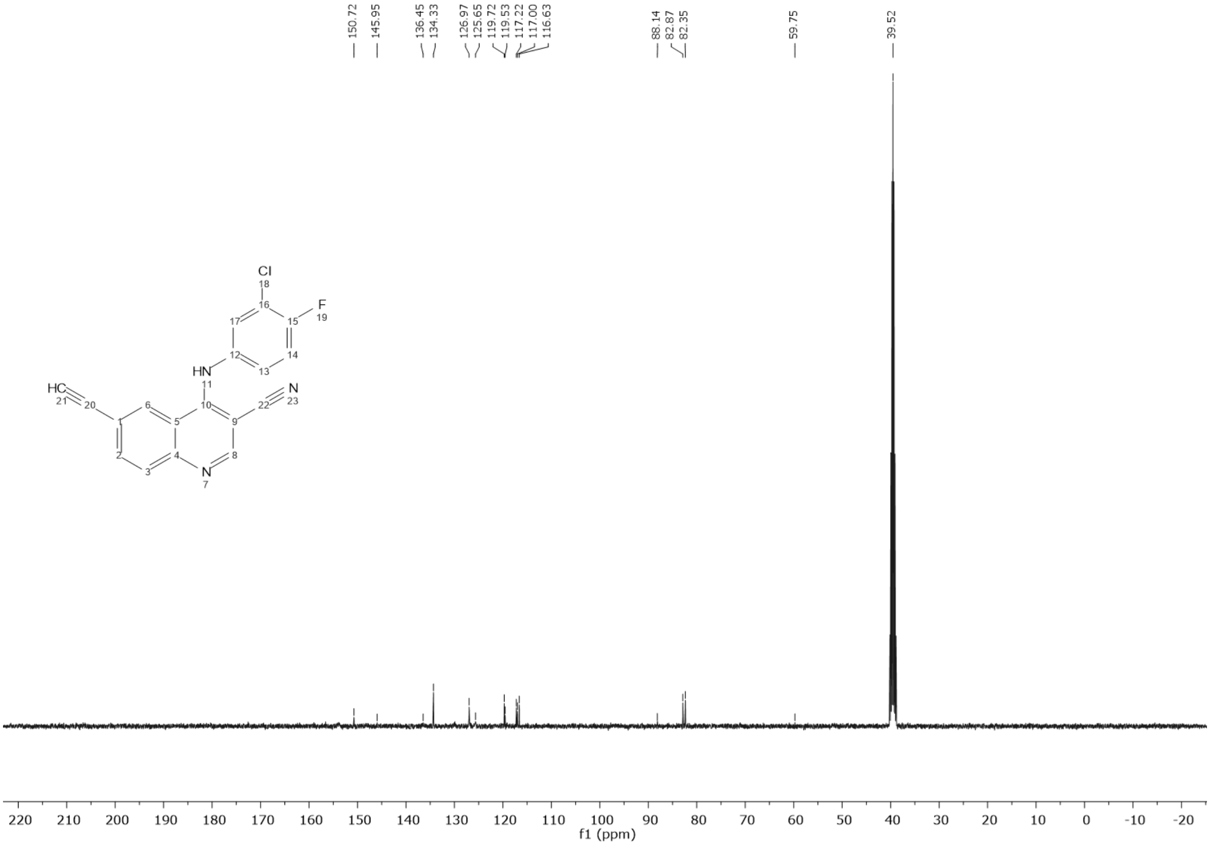

Sample Report

GOETHE UNIVERSITÄT FRANKFURT AM MAIN

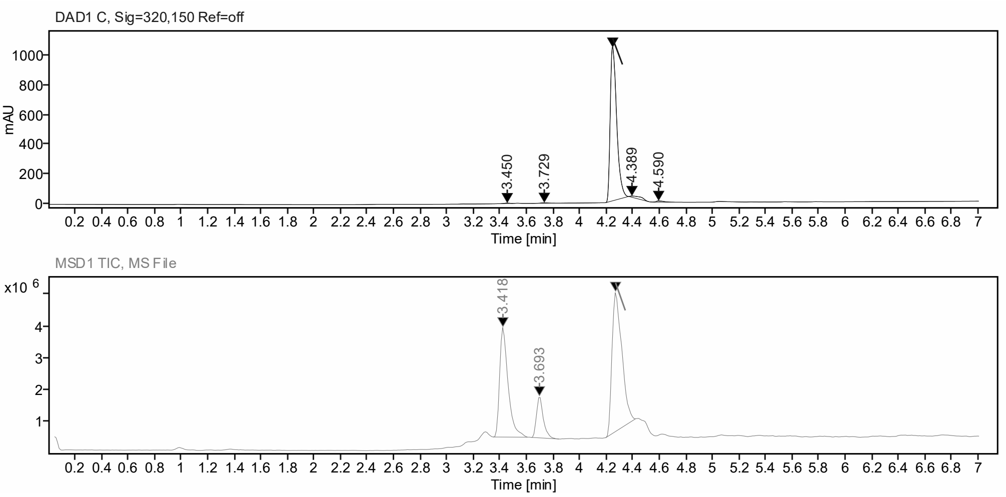

Sample Purity

Signal Description DAD1 C, Sig=320,150 Ref=off

Sample Name	Name	RT	Width	Area	Area%	Height
FGMM165_16h		3.450	0.046	8.2911	0.23	3.0771
FGMM165_16h		3.729	0.053	12.2348	0.34	3.4388
FGMM165_16h		4.244	0.052	3511.9519	96.24	1033.9667
FGMM165_16h		4.389	0.064	86.3850	2.37	8.5977
FGMM165_16h		4.590	0.058	30.2145	0.83	8.1270

Max Area% 96.242

UV Signal Purity>95% Pass

UV Apex Spectra

GOETHE UNIVERSITÄT FRANKFURT AM MAIN

MSD Apex Spectra

RT:

3.418

Sample Name:

FGMM165_16h

Signal Name:

MSD1TIC

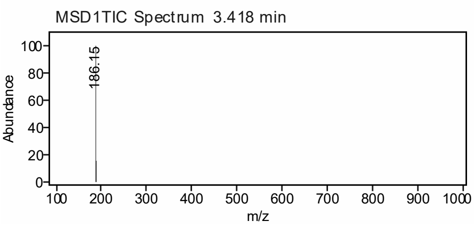

RT:

3.693

Sample Name:

FGMM165_16h

Signal Name:

MSD1TIC

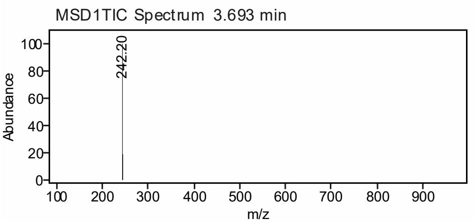

RT:

4.266

Sample Name:

FGMM165_16h

Signal Name:

MSD1TIC

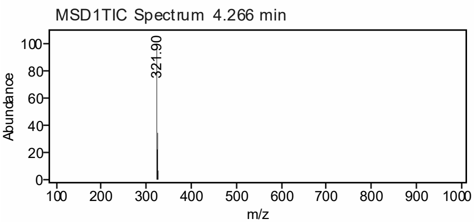

Compound 67

Sample Report

GOETHE UNIVERSITÄT FRANKFURT AM MAIN

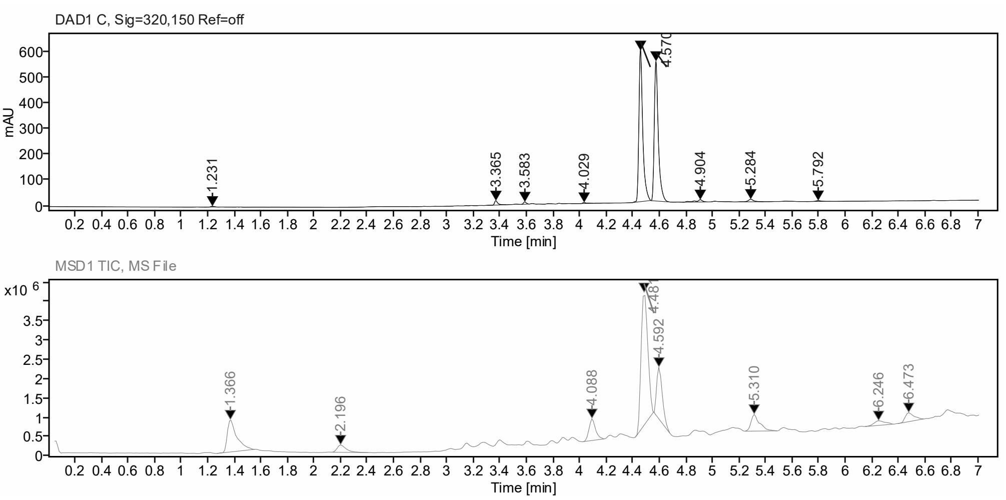

Sample Purity

Signal Description DAD1 C, Sig=320,150 Ref=off

Sample Name	Name	RT	Width	Area	Area%	Height
FGMM136		1.231	0.028	5.9811	0.24	3.0875
FGMM136		3.365	0.025	29.7010	1.21	16.6391
FGMM136		3.583	0.024	13.3297	0.54	8.9333
FGMM136		4.029	0.026	8.3179	0.34	3.9757
FGMM136		4.454	0.029	1185.1271	48.38	584.8193
FGMM136		4.570	0.030	1135.8829	46.37	541.2429
FGMM136		4.904	0.033	32.3904	1.32	8.6790
FGMM136		5.284	0.039	29.5119	1.20	10.0226
FGMM136		5.792	0.041	9.3926	0.38	3.3052

Max Area% 48.380

UV Signal Purity>95% Fail

UV Apex Spectra

RT:

1.366

Sample Name:

FGMM136

Signal Name:

MSD1TIC

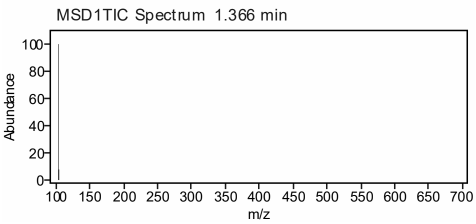

RT:

2.196

Sample Name:

FGMM136

Signal Name:

MSD1TIC

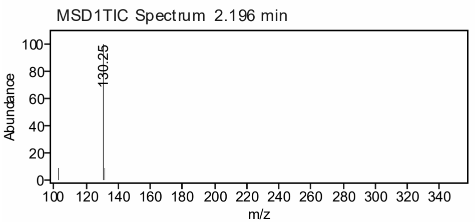

RT:

4.088

Sample Name:

FGMM136

Signal Name:

MSD1TIC

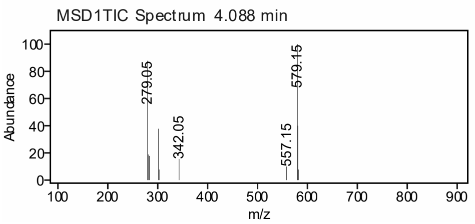

RT:

4.481

Sample Name:

FGMM136

Signal Name:

MSD1TIC

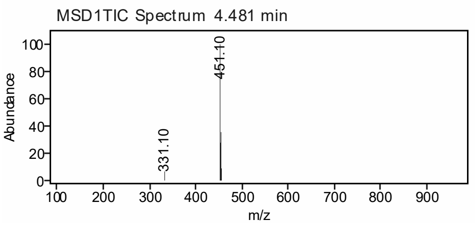

RT:

4.592

Sample Name:

FGMM136

Signal Name:

MSD1TIC

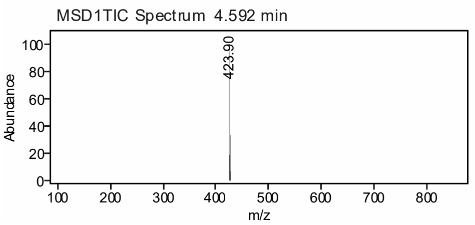

Compound 45

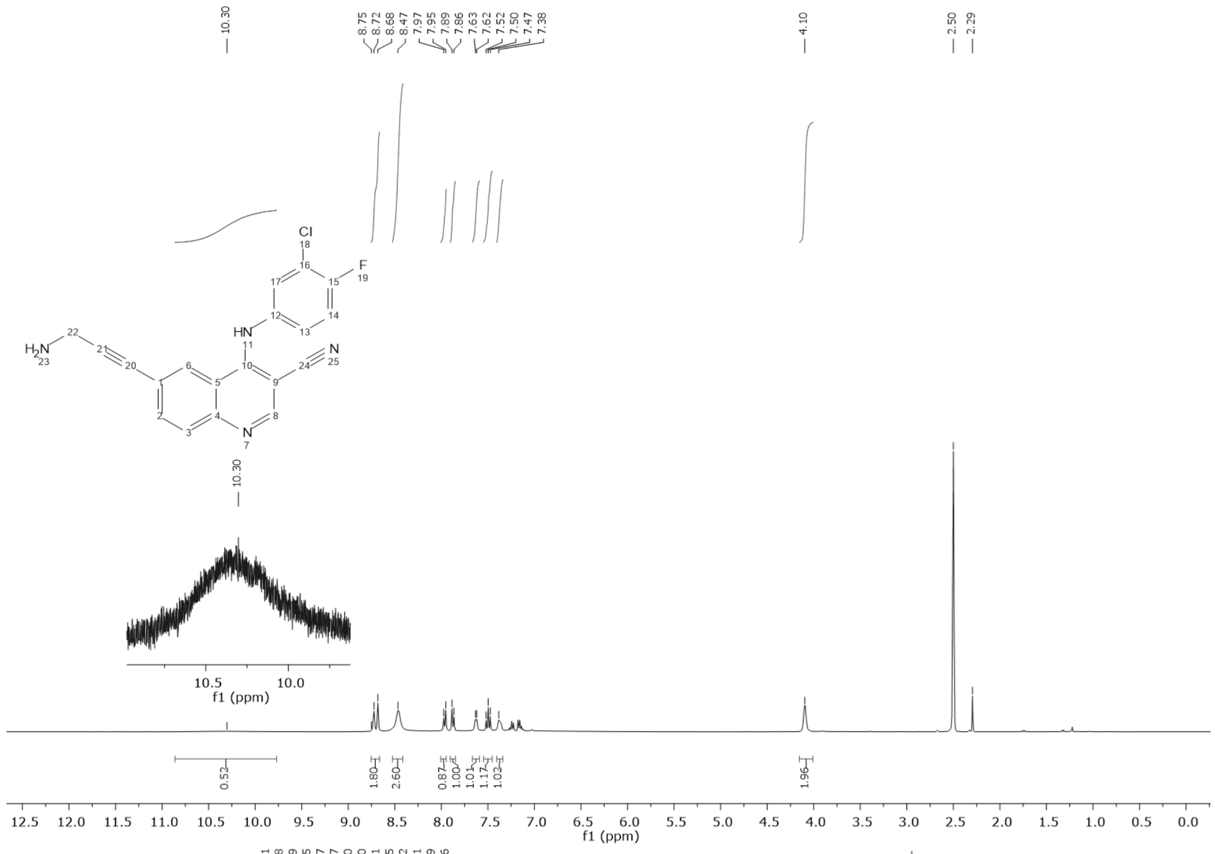

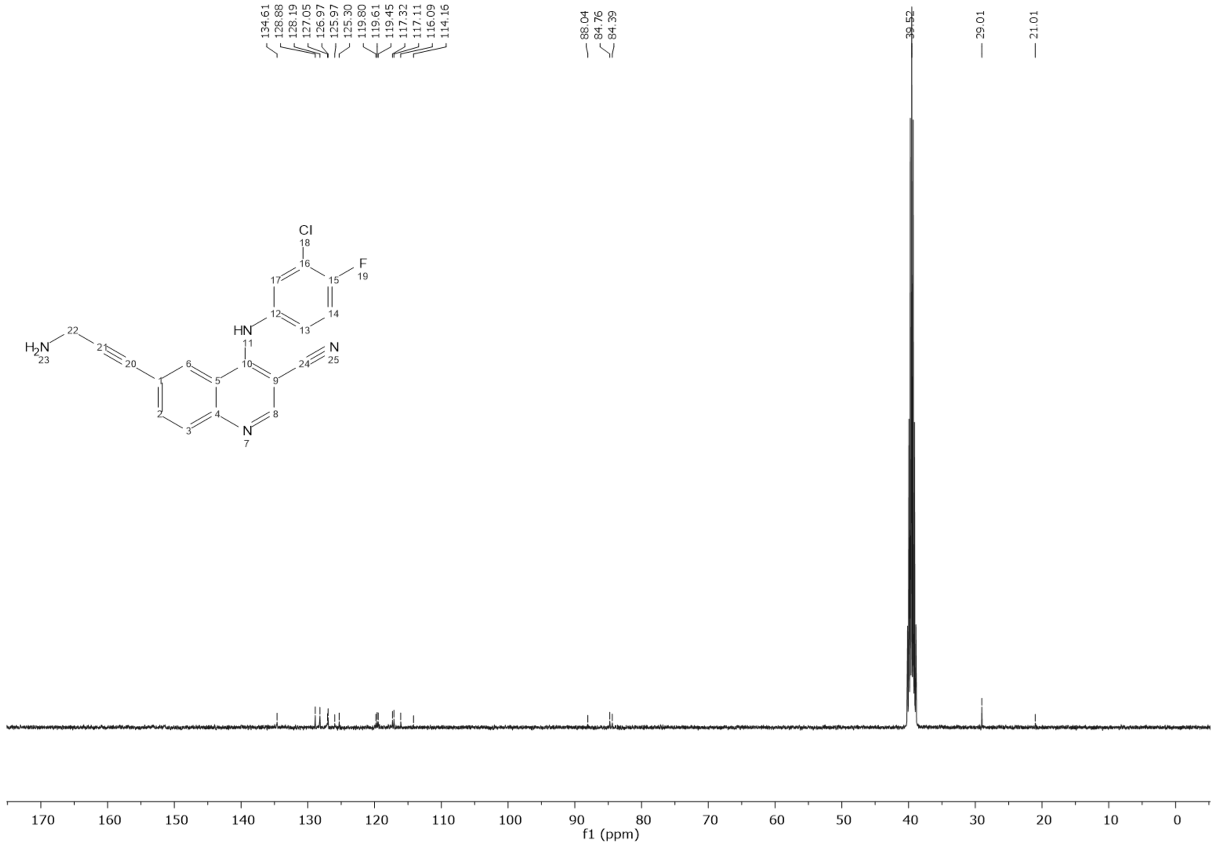

GOETHE UNIVERSITÄT FRANKFURT AM MAIN

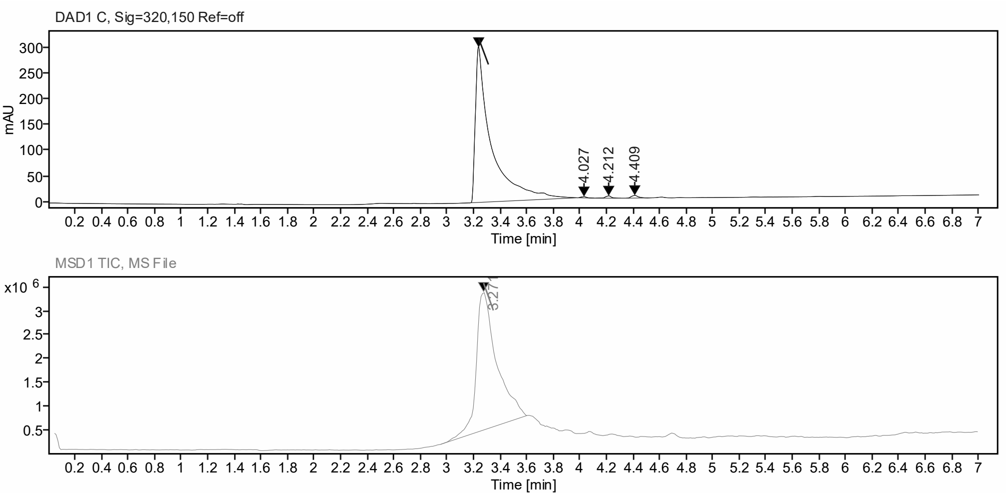

Sample Purity

Signal Description DAD1 C, Sig=320,150 Ref=off

Sample Name	Name	RT	Width	Area	Area%	Height
FGMM149_F2		3.234	0.090	2525.6697	98.48	301.8723
FGMM149_F2		4.027	0.047	7.0034	0.27	2.4188
FGMM149_F2		4.212	0.045	13.4713	0.53	4.4922
FGMM149_F2		4.409	0.055	18.5195	0.72	5.2088

Max Area% 98.480

UV Signal Purity>95% Pass

UV Apex Spectra

RT:

3.234

Sample Name:

FGMM149_F2

Signal Name:

DAD1C

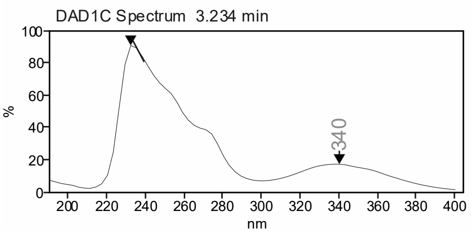

GOETHE UNIVERSITÄT FRANKFURT AM MAIN

RT:

4.027

Sample Name:

FGMM149_F2

Signal Name:

DAD1C

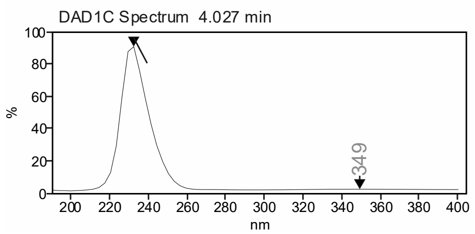

RT:

4.212

Sample Name:

FGMM149_F2

Signal Name:

DAD1C

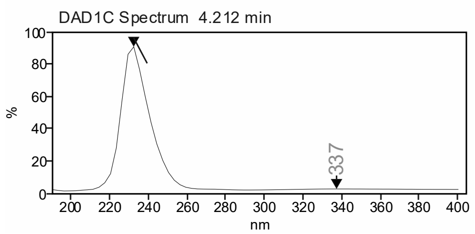

RT:

4.409

Sample Name:

FGMM149_F2

Signal Name:

DAD1C

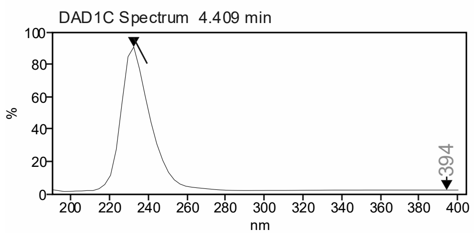

MSD Apex Spectra

RT:

3.271

Sample Name:

FGMM149_F2

Signal Name:

MSD1TIC

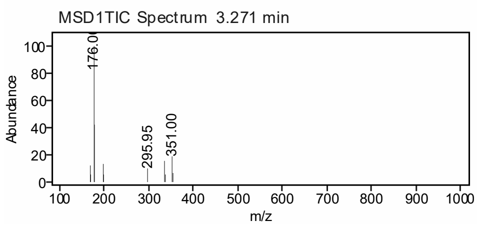

Compound 46

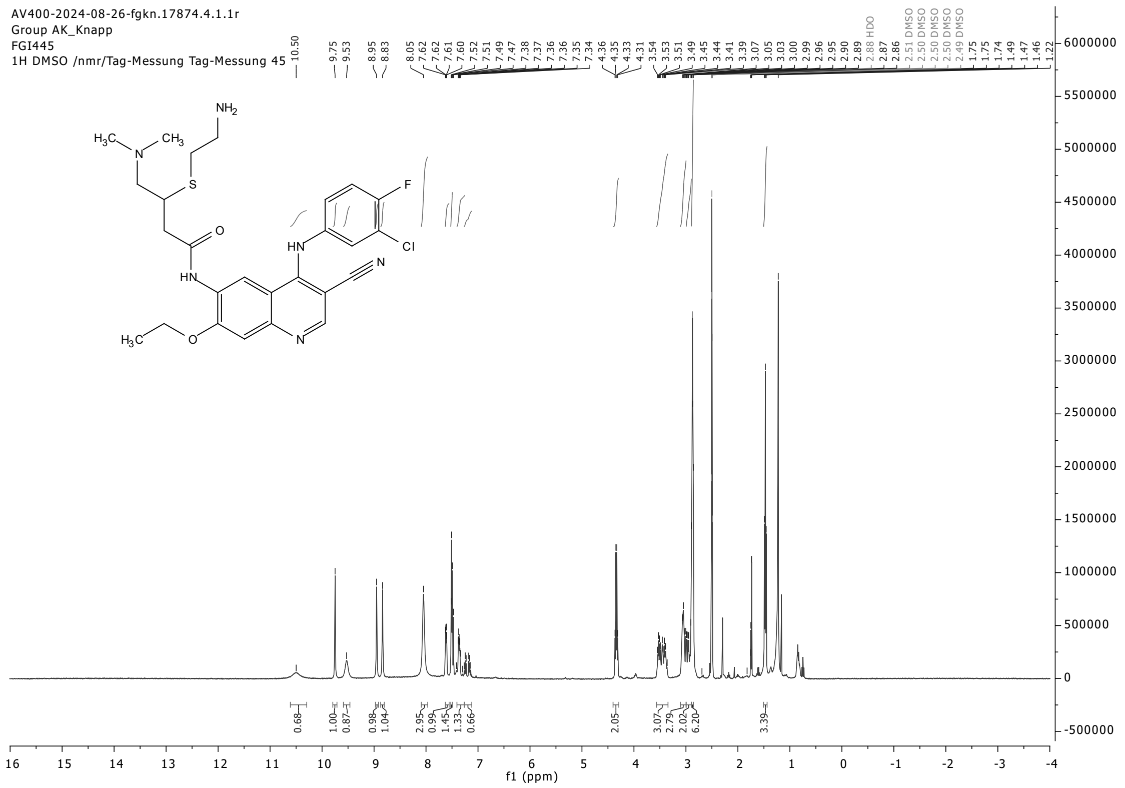

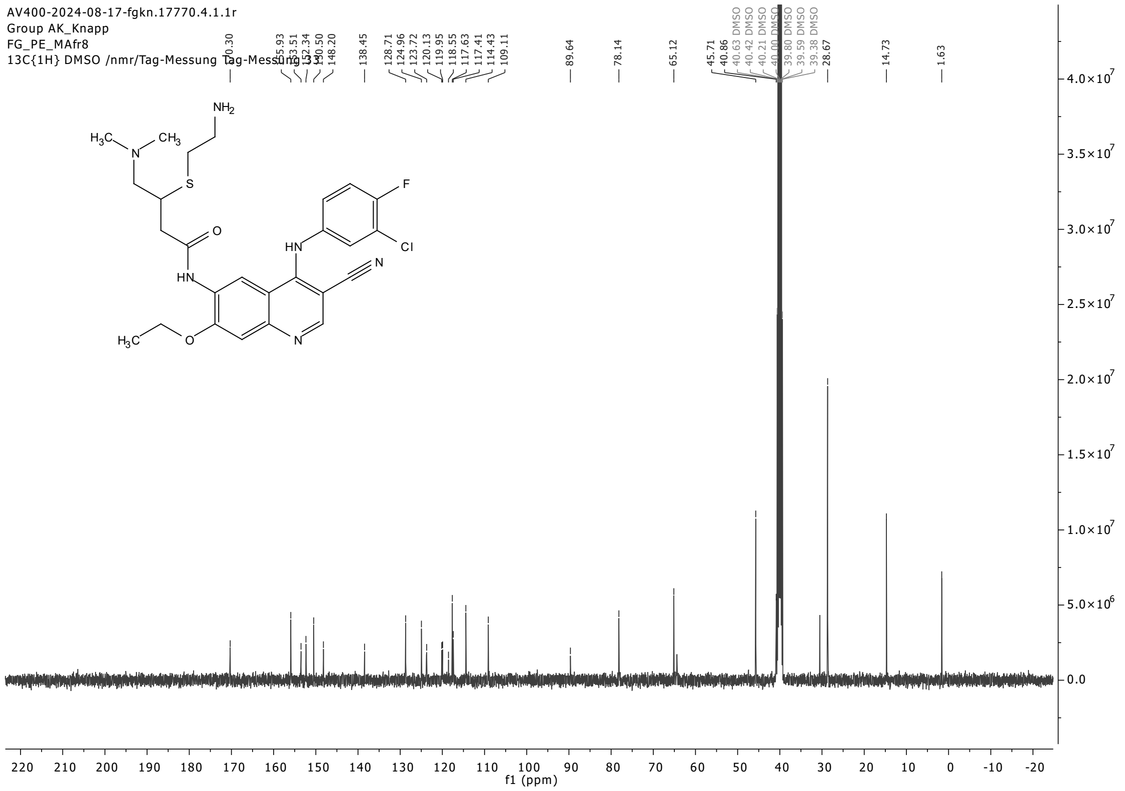

Sample Report

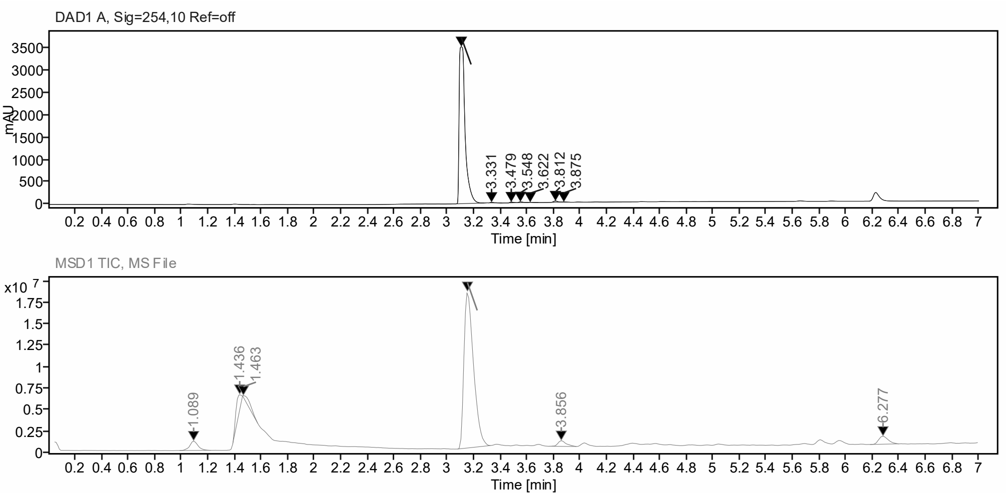

Sample Purity

Signal Description DAD1 A, Sig=254,10 Ref=off

Sample Name	Name	RT	Width	Area	Area%	Height
FGI445		3.104	0.045	10972.7598	98.45	3513.7026
FGI445		3.331	0.026	29.2957	0.26	13.5666
FGI445		3.479	0.030	32.5358	0.29	18.0961
FGI445		3.548	0.030	34.4748	0.31	18.1410
FGI445		3.622	0.026	10.4407	0.09	5.9035
FGI445		3.812	0.034	58.5482	0.53	28.9146
FGI445		3.875	0.032	7.6550	0.07	3.6100

Max Area% 98.448

UV Signal Purity>95% Pass

UV Apex Spectra

GOETHE UNIVERSITÄT FRANKFURT AM MAIN

RT:

1.463

Sample Name:

FGI445

Signal Name:

MSD1TIC

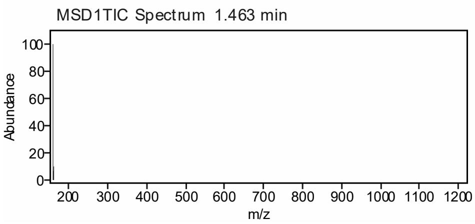

RT:

3.150

Sample Name:

FGI445

Signal Name:

MSD1TIC

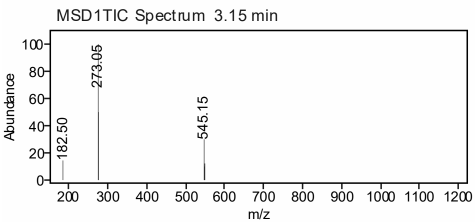

RT:

3.856

Sample Name:

FGI445

Signal Name:

MSD1TIC

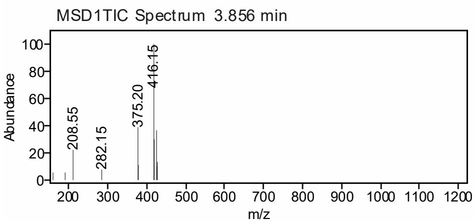

RT:

6.277

Sample Name:

FGI445

Signal Name:

MSD1TIC

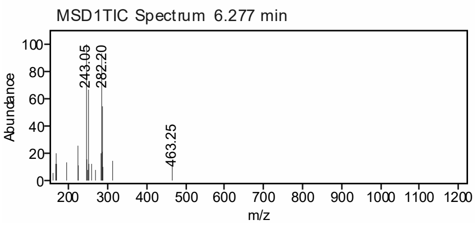

Compound 47

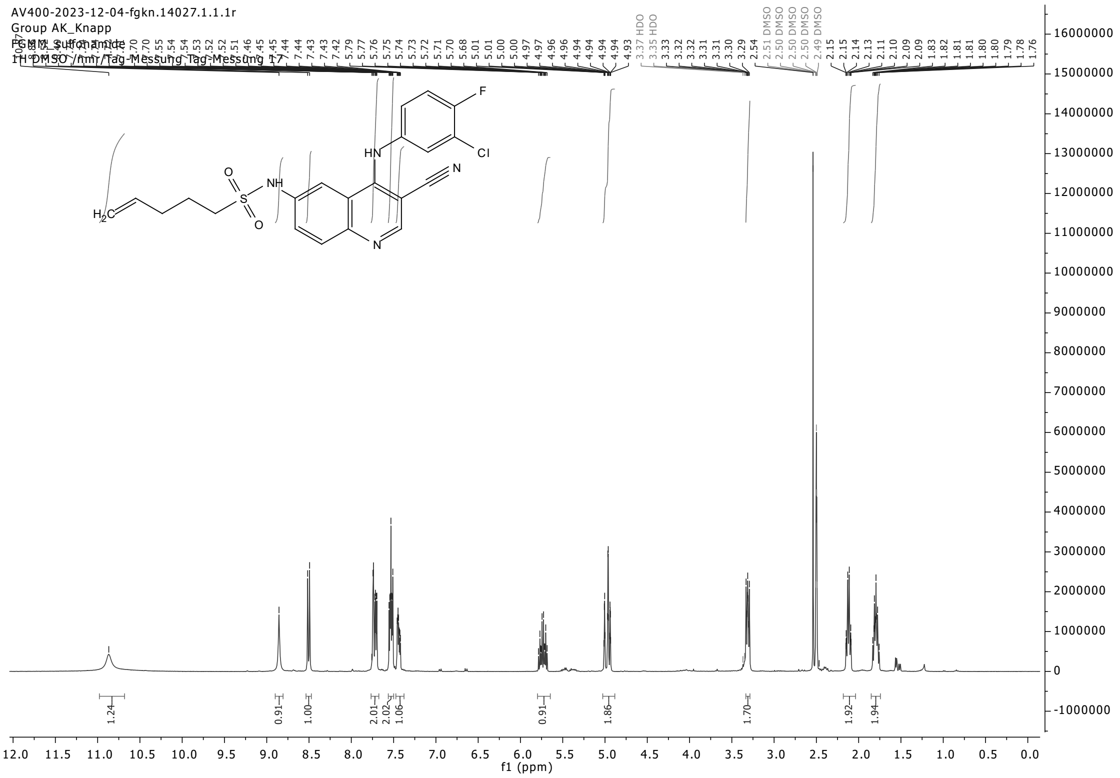

AV400-2023-12-04-fgkn.14027.1.1.1r
Group AK_Knapp
FGMM_sulfonamide
1H DMSO /nmr/Tag-Messung Tag-Messung 17
f1 (ppm)

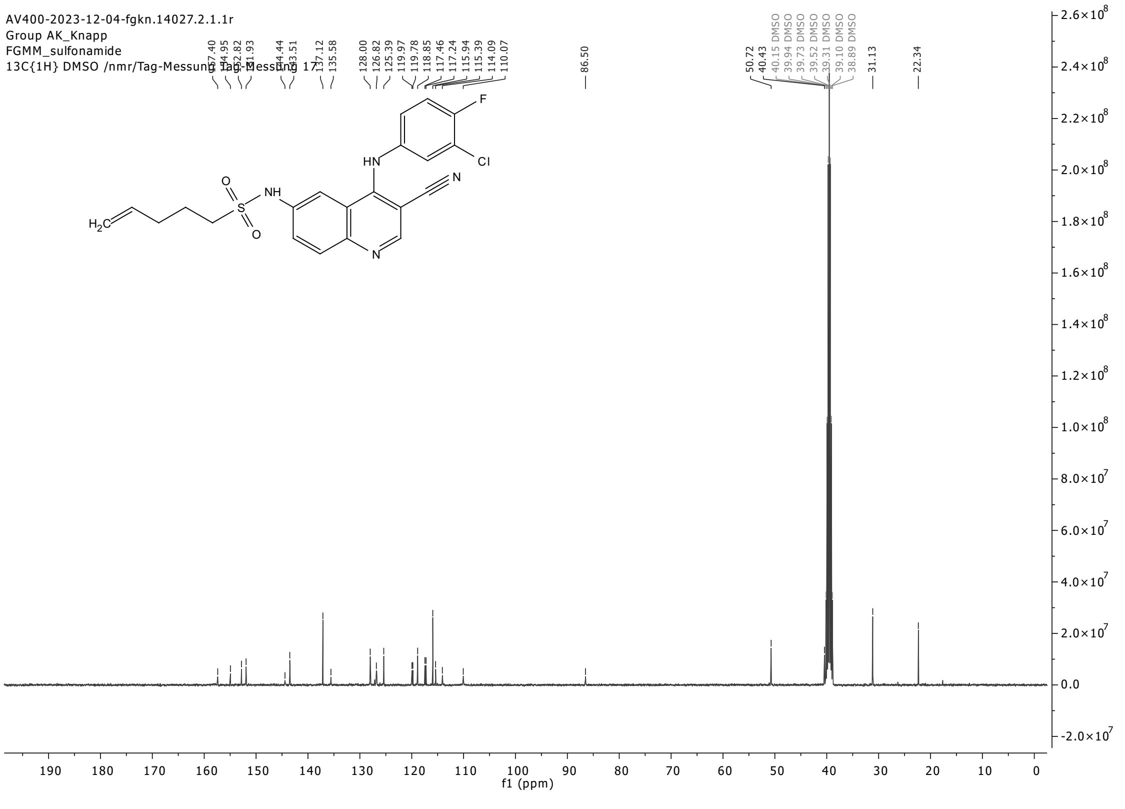

AV400-2023-12-04-fgkn.14027.2.1.1r
Group AK_Knapp
FGMM_sulfonamide
13C{1H} DMSO /nmr/Tag-Messung Tag-Messung 17
f1 (ppm)

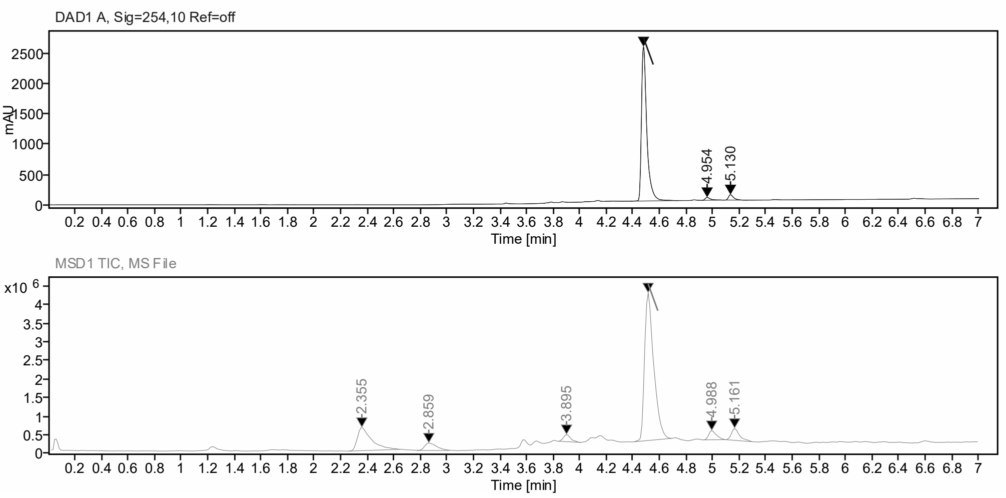

Sample Purity

Signal Description	DAD1 A, Sig=254,10 Ref=off

Sample Name	Name	RT	Width	Area	Area%	Height
FGMM_sulfonamide_f9		4.475	0.041	7190.1650	95.06	2563.8198
FGMM_sulfonamide_f9		4.954	0.041	137.8349	1.82	46.7500
FGMM_sulfonamide_f9		5.130	0.040	236.0561	3.12	87.9588

Max Area%	95.057
UV Signal Purity>95%	Pass

UV Apex Spectra

RT:

4.475

Sample Name:

FGMM_sulfonamide_f9

Signal Name:

DAD1A

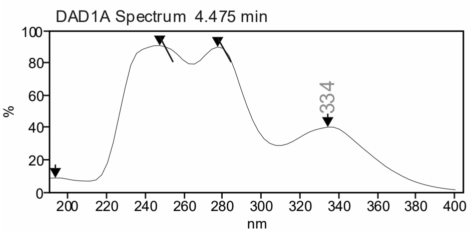

GOETHE UNIVERSITÄT FRANKFURT AM MAIN

RT:

3.895

Sample Name:

FGMM_sulfonamide_f9

Signal Name:

MSD1TIC

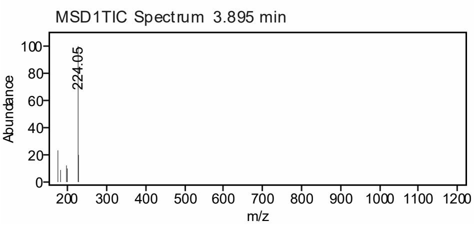

RT:

4.509

Sample Name:

FGMM_sulfonamide_f9

Signal Name:

MSD1TIC

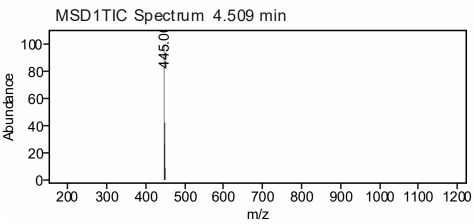

RT:

4.988

Sample Name:

FGMM_sulfonamide_f9

Signal Name:

MSD1TIC

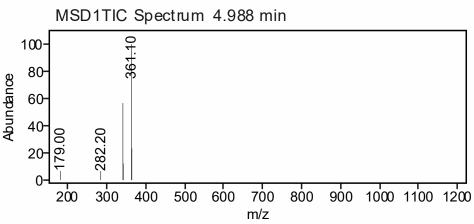

RT:

5.161

Sample Name:

FGMM_sulfonamide_f9

Signal Name:

MSD1TIC

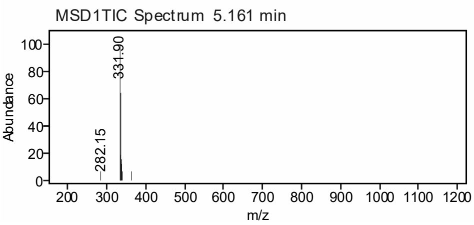

Compound 48

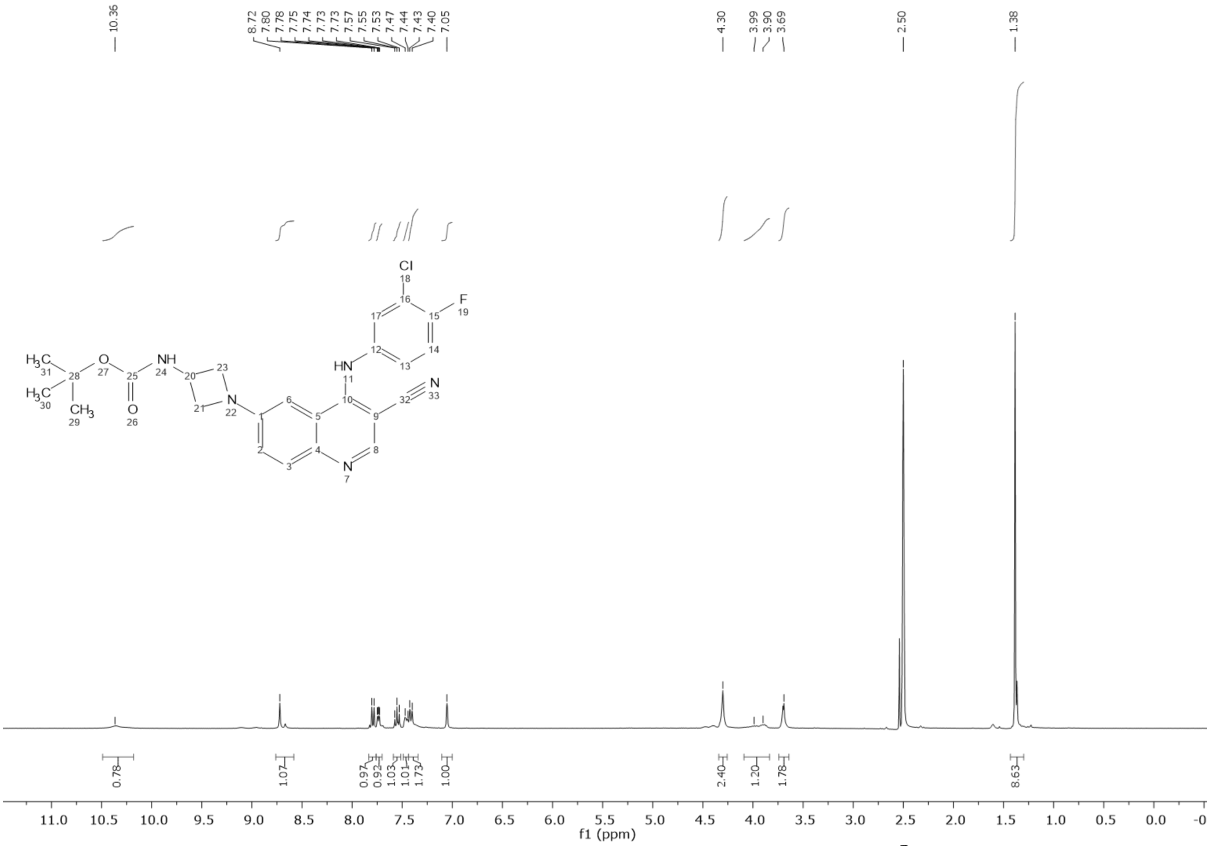

10.36
8.72
7.80
7.78
7.75
7.74
7.73
7.73
7.57
7.55
7.53
7.47
7.44
7.43
7.40
7.05
4.30
3.99
3.90
3.69
2.50
1.38
0.78
1.07
0.97
0.92
1.03
1.01
1.73
1.00
2.40
1.20
1.78
8.63
Cl
F
HN
N
NH
O
H_3C
CH_3
f1 (ppm)

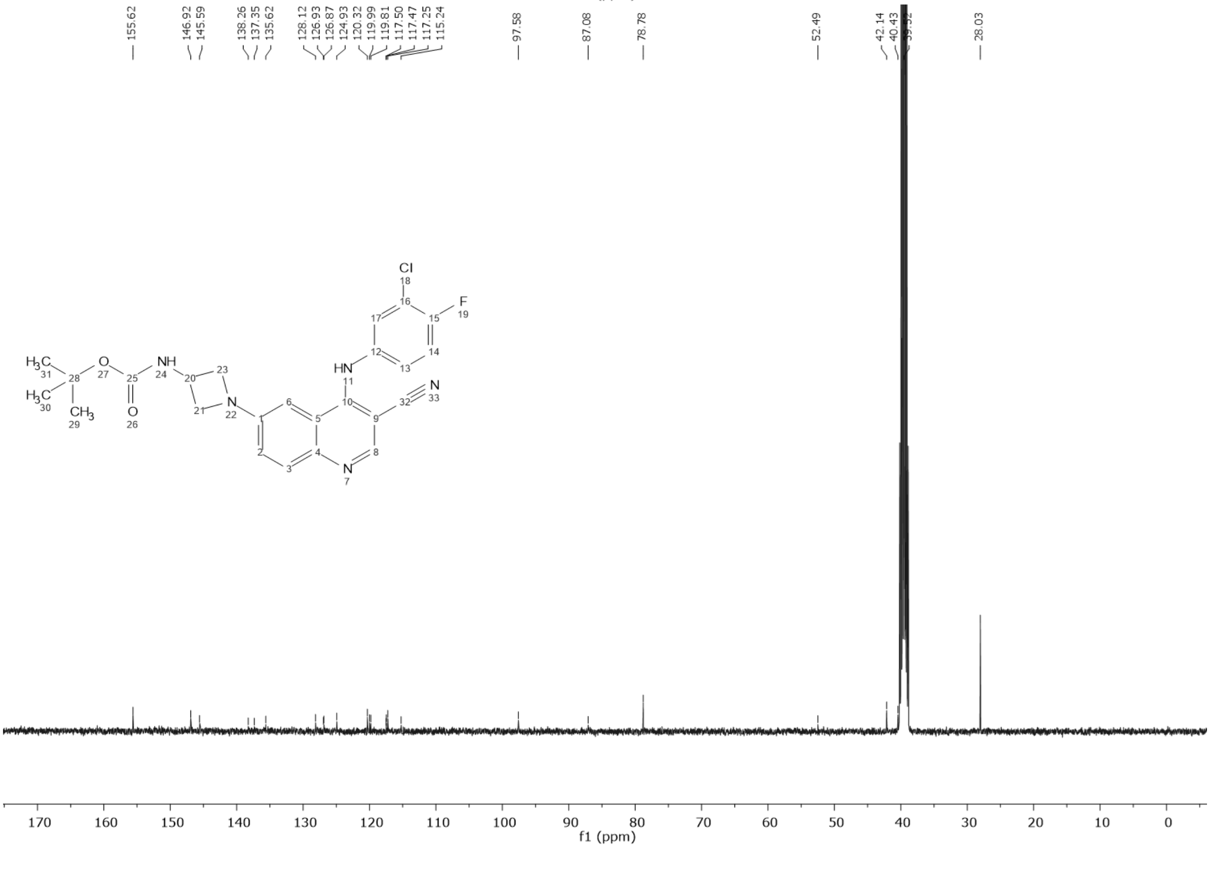

155.62
146.92
145.59
138.26
137.35
135.62
128.12
126.93
126.87
124.93
120.32
119.99
119.81
117.50
117.47
117.25
115.24
97.58
87.08
78.78
52.49
42.14
40.43
28.03
Cl
F
HN
N
NH
O
H_3C
CH_3
f1 (ppm)

Sample Report

GOETHE UNIVERSITÄT FRANKFURT AM MAIN

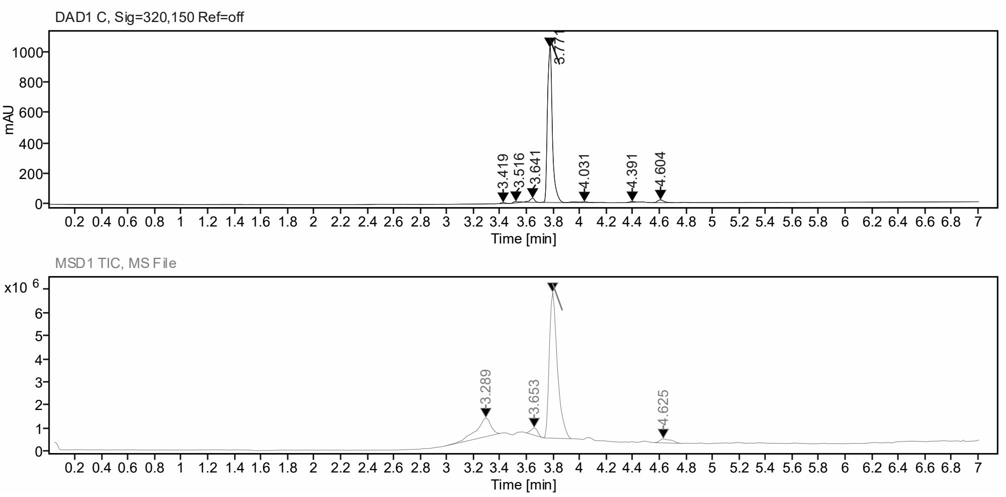

Sample Purity

Signal Description DAD1 C, Sig=320,150 Ref=off

Sample Name	Name	RT	Width	Area	Area%	Height
FGMM150_F23		3.419	0.035	17.0017	0.59	7.4304
FGMM150_F23		3.516	0.073	28.1829	0.99	9.6274
FGMM150_F23		3.641	0.038	70.9679	2.48	27.1334
FGMM150_F23		3.771	0.039	2635.5095	92.17	1023.1948
FGMM150_F23		4.031	0.138	36.5019	1.28	5.0460
FGMM150_F23		4.391	0.043	13.9739	0.49	5.5183
FGMM150_F23		4.604	0.050	57.3722	2.01	17.8827

Max Area% 92.166

UV Signal Purity>95% Fail

UV Apex Spectra

Sample Report

GOETHE UNIVERSITÄT FRANKFURT AM MAIN

RT:

3.792

Sample Name:

FGMM150_F23

Signal Name:

MSD1TIC

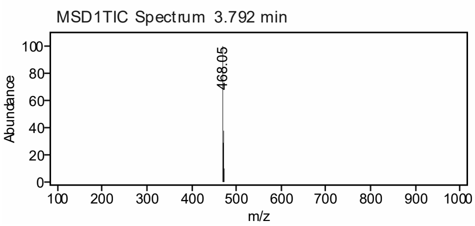

RT:

4.625

Sample Name:

FGMM150_F23

Signal Name:

MSD1TIC

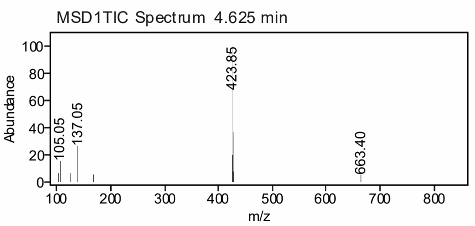

Compound 49

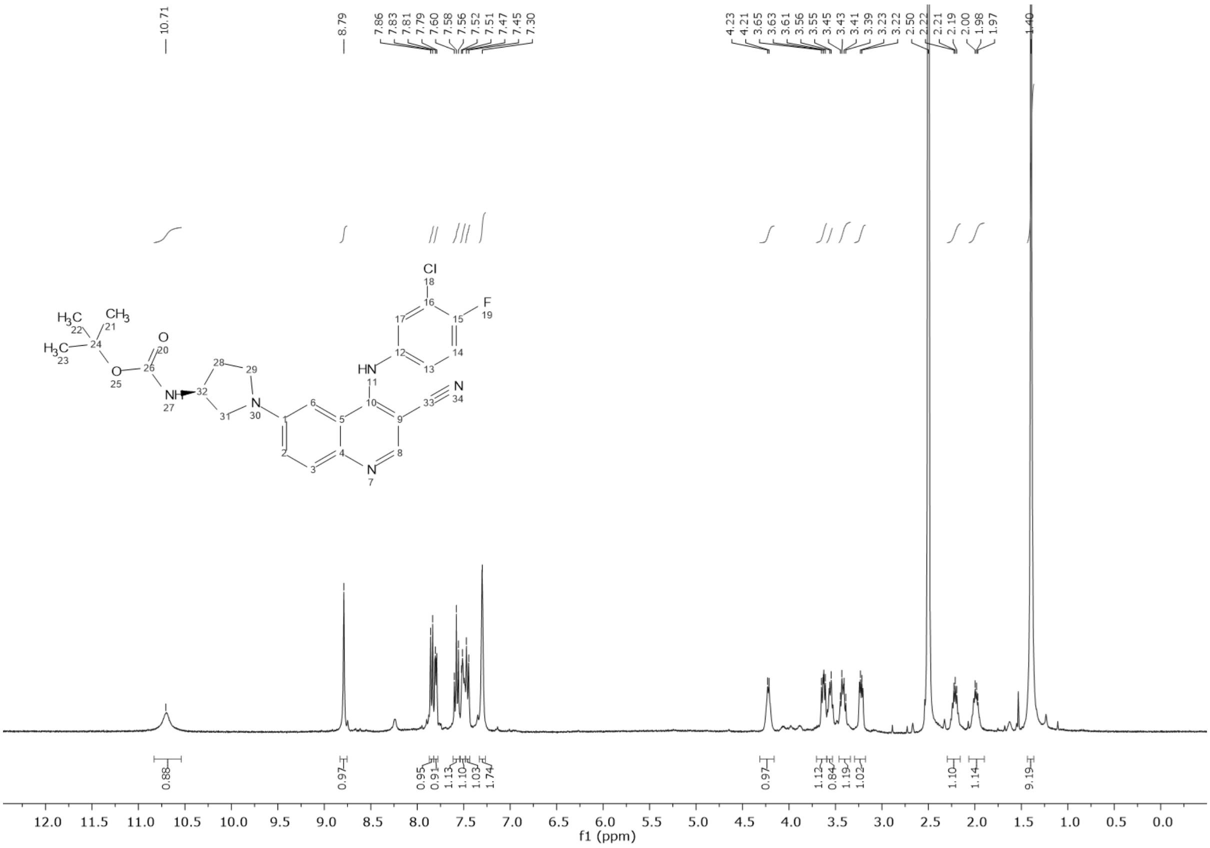

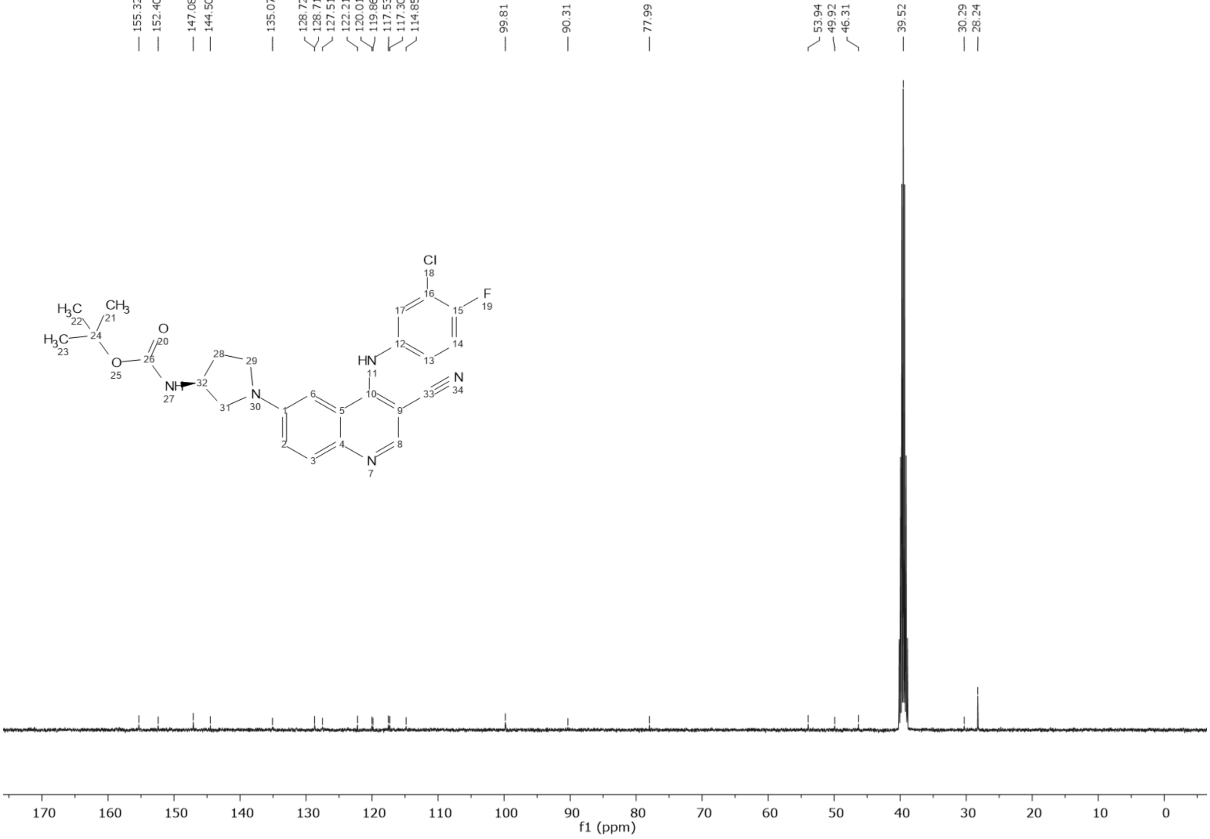

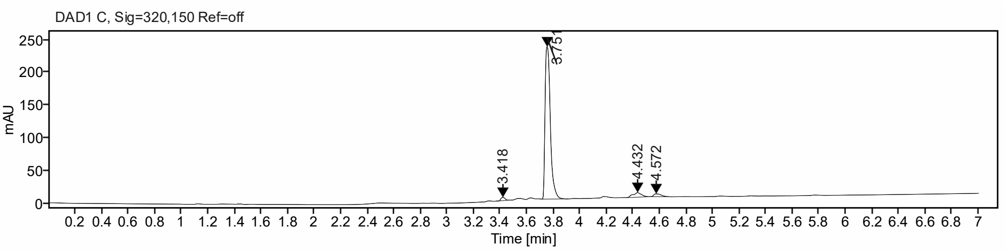

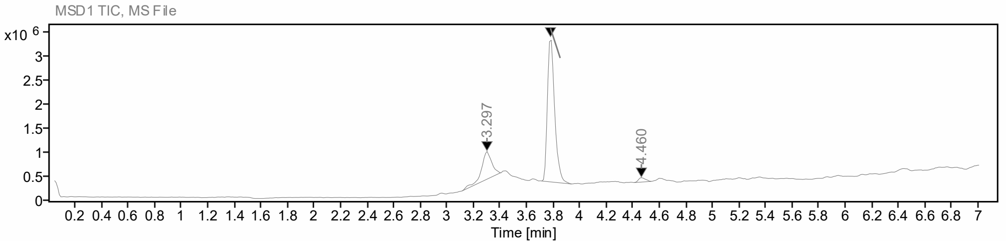

Sample Purity

Signal Description DAD1 C, Sig=320,150 Ref=off

Sample Name	Name	RT	Width	Area	Area%	Height
FGMM152_F23		3.418	0.038	10.6572	1.52	4.7254
FGMM152_F23		3.751	0.043	642.8449	91.60	231.7890
FGMM152_F23		4.432	0.081	31.1003	4.43	6.8787
FGMM152_F23		4.572	0.063	17.2000	2.45	4.2990

Max Area% 91.599
UV Signal Purity>95% Fail

UV Apex Spectra

RT:

3.418

Sample Name:

FGMM152_F23

Signal Name:

DAD1C

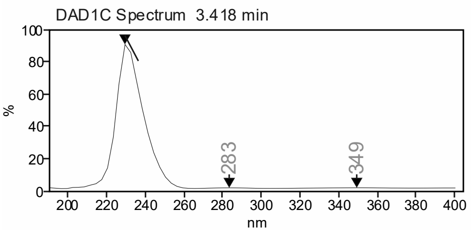

RT:

3.775

Sample Name:

FGMM152_F23

Signal Name:

MSD1TIC

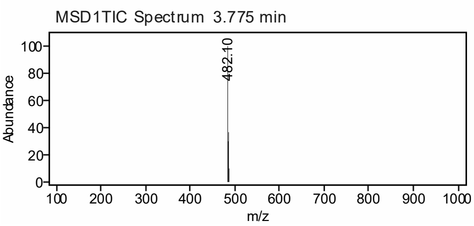

RT:

4.460

Sample Name:

FGMM152_F23

Signal Name:

MSD1TIC

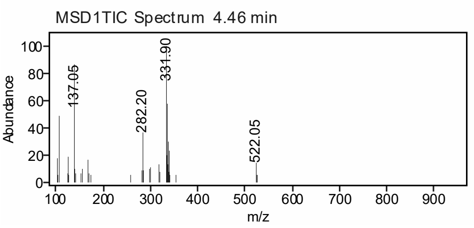

Compound S1

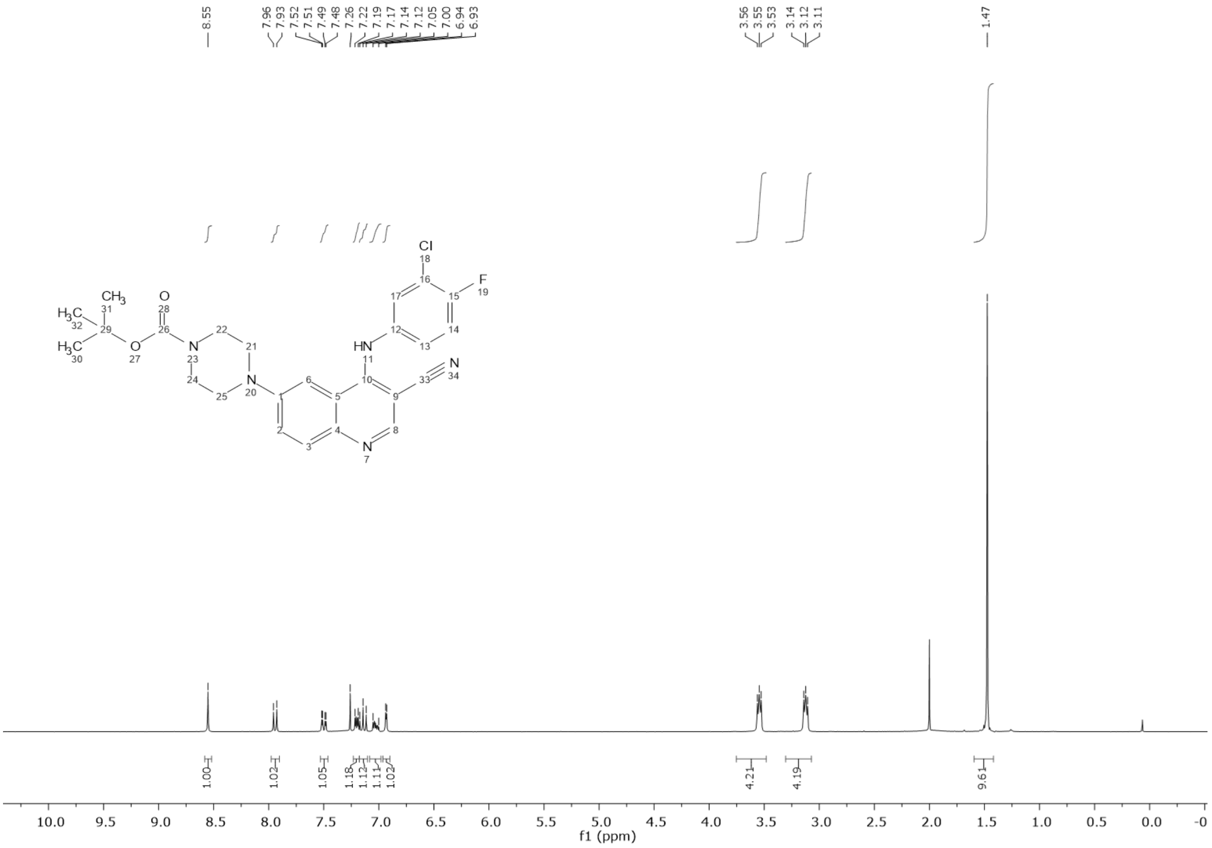
8.55
7.96
7.93
7.52
7.51
7.49
7.48
7.26
7.22
7.19
7.17
7.14
7.12
7.05
7.00
6.94
6.93
3.56
3.55
3.53
3.14
3.12
3.11
1.47
1.00
1.02
1.05
1.18
1.13
1.11
1.02
4.21
4.19
9.61
f1 (ppm)

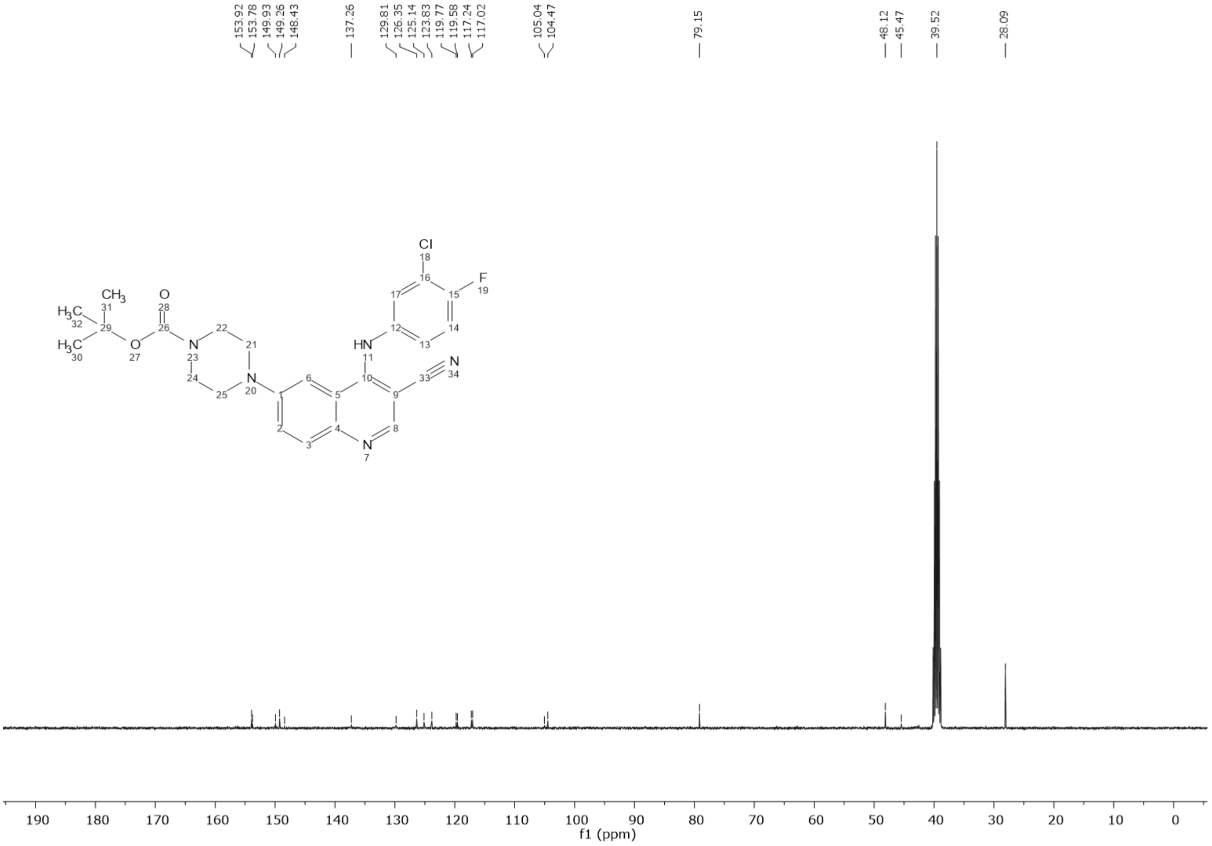
153.92
153.78
149.93
149.26
148.43
137.26
129.81
126.35
125.14
123.83
119.77
119.58
117.24
117.02
105.04
104.47
79.15
48.12
45.47
39.52
28.09
f1 (ppm)

GOETHE UNIVERSITÄT FRANKFURT AM MAIN

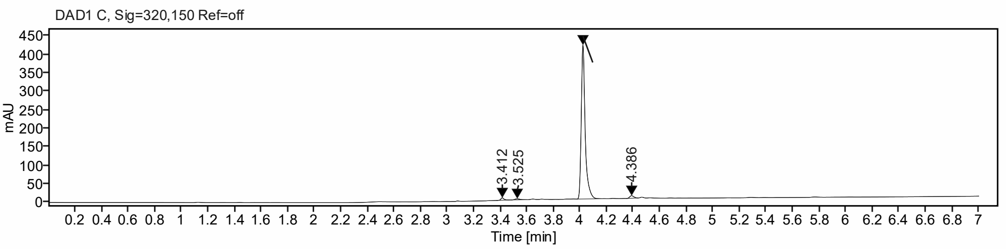

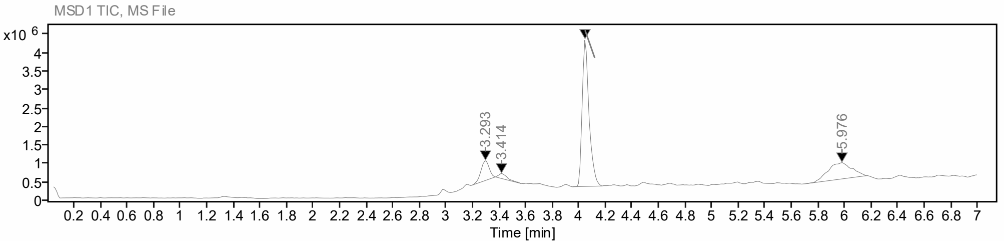

Sample Purity

Signal Description DAD1 C, Sig=320,150 Ref=off

Sample Name	Name	RT	Width	Area	Area%	Height
FGMM140re_F20		3.412	0.025	11.0592	1.25	6.3924
FGMM140re_F20		3.525	0.040	7.7316	0.87	2.8985
FGMM140re_F20		4.019	0.029	850.4661	96.17	415.9816
FGMM140re_F20		4.386	0.035	15.0452	1.70	6.8806

Max Area% 96.174

UV Signal Purity>95% Pass

UV Apex Spectra

RT:

3.412

Sample Name:

FGMM140re_F20

Signal Name:

DAD1C

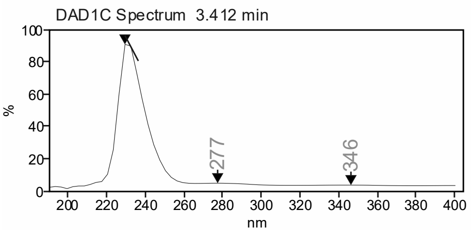

GOETHE UNIVERSITÄT FRANKFURT AM MAIN

RT:

3.414

Sample Name:

FGMM140re_F20

Signal Name:

MSD1TIC

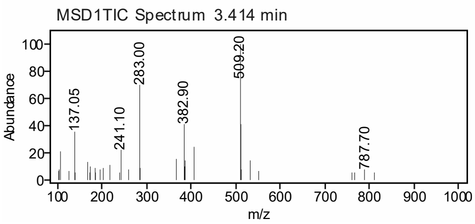

RT:

4.042

Sample Name:

FGMM140re_F20

Signal Name:

MSD1TIC

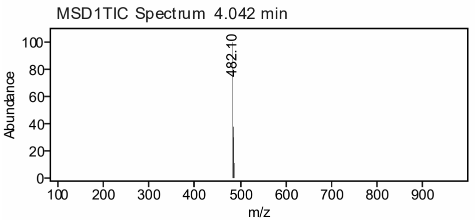

RT:

5.976

Sample Name:

FGMM140re_F20

Signal Name:

MSD1TIC

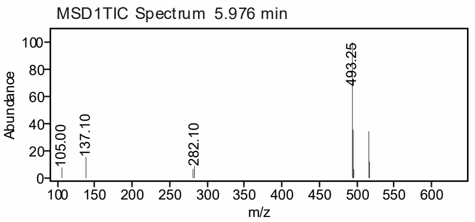

Compound 50

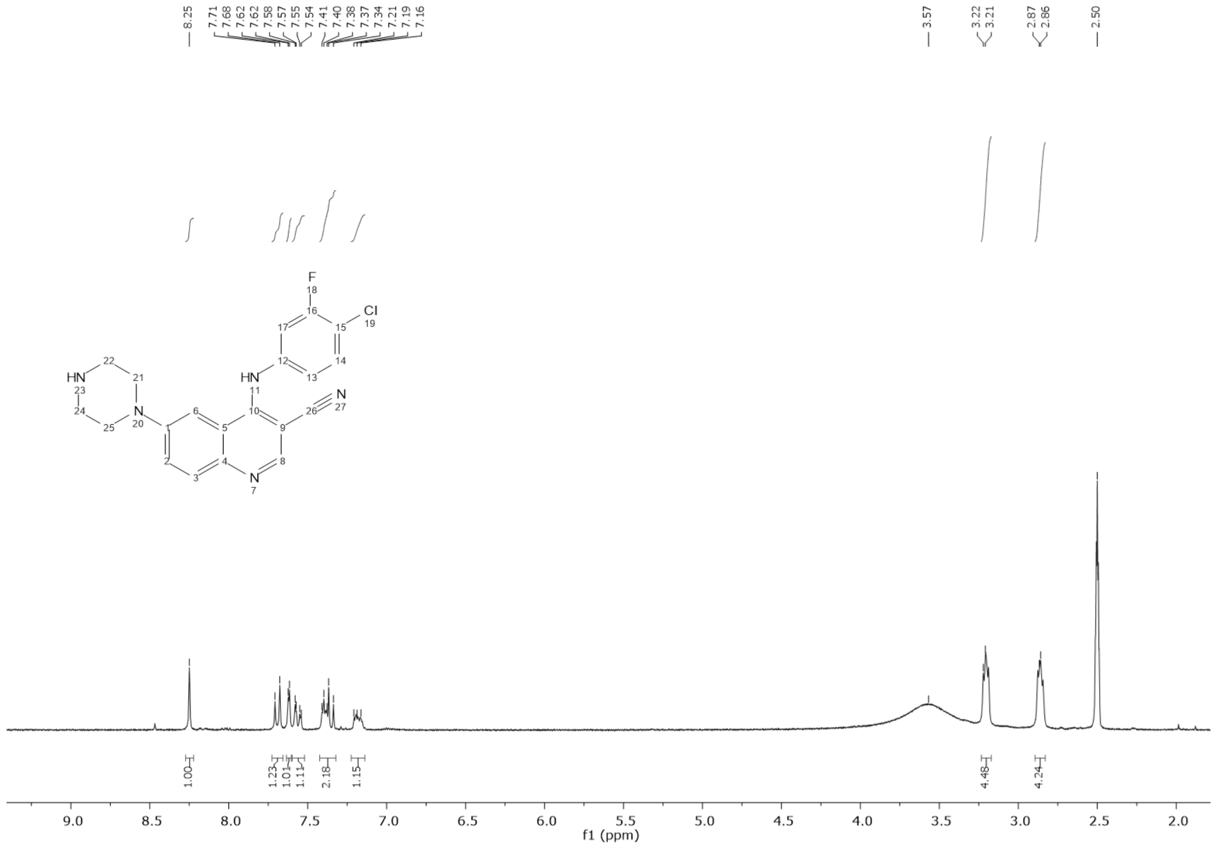
8.25
7.71 7.68 7.62 7.62 7.58 7.57 7.55 7.54 7.41 7.40 7.38 7.37 7.34 7.21 7.19 7.16
3.57
3.22 3.21
2.87 2.86
2.50
1.00 1.23 1.01 1.11 2.18 1.15 4.48 4.24
9.0 8.5 8.0 7.5 7.0 6.5 6.0 5.5 5.0 4.5 4.0 3.5 3.0 2.5 2.0
f1 (ppm)

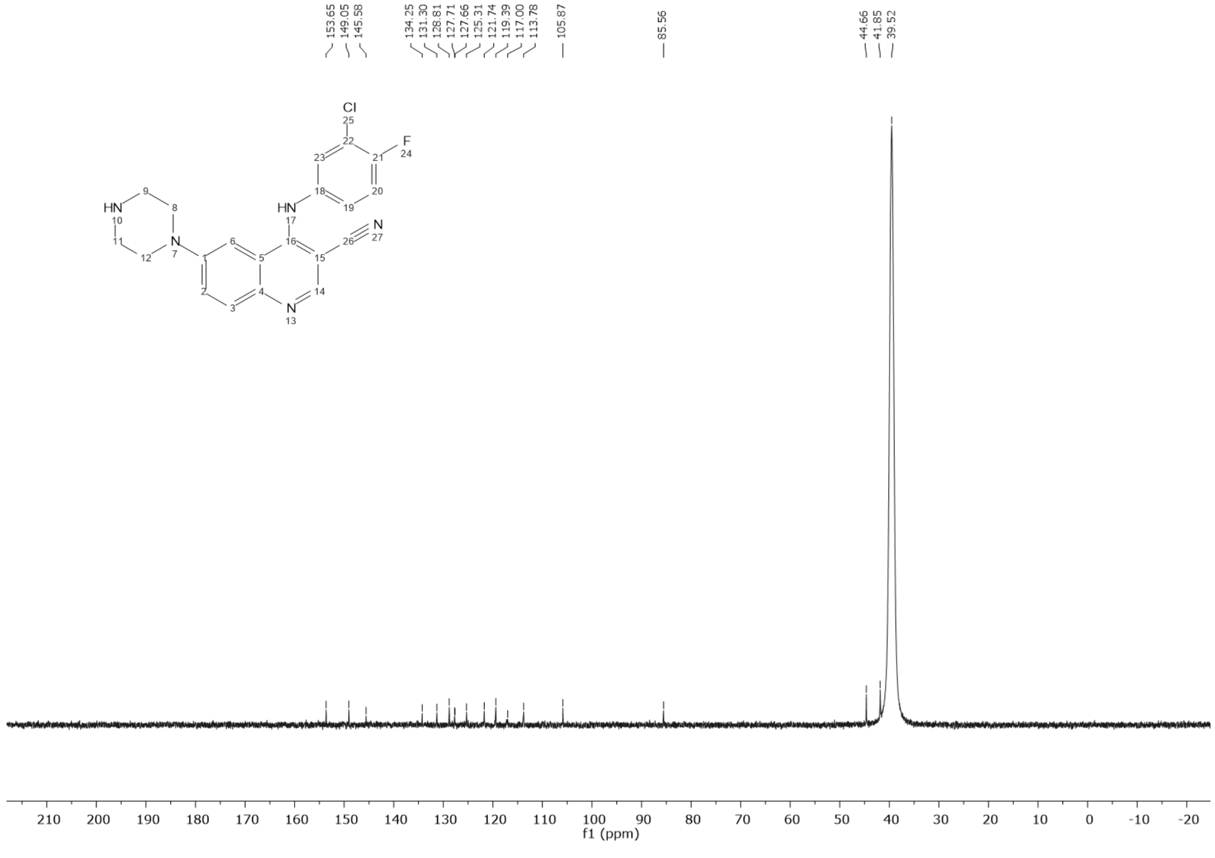
153.65 149.05 145.58
134.25 131.30 128.81 127.71 127.66 125.31 121.74 119.39 117.00 113.78
105.87
85.56
44.66 41.85 39.52
210 200 190 180 170 160 150 140 130 120 110 100 90 80 70 60 50 40 30 20 10 0 -10 -20
f1 (ppm)

GOETHE UNIVERSITÄT FRANKFURT AM MAIN

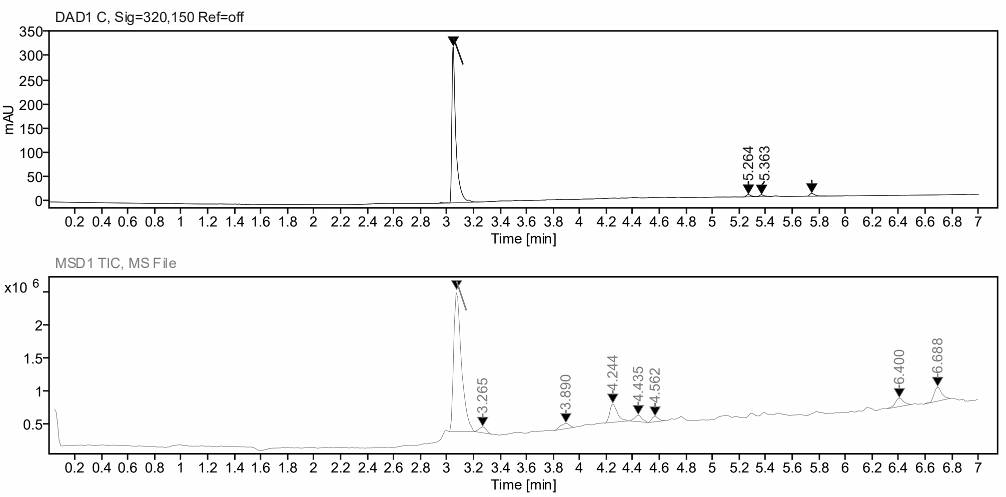

Sample Purity

Signal Description DAD1 C, Sig=320,150 Ref=off

Sample Name	Name	RT	Width	Area	Area%	Height
FGMM95_F14		3.043	0.028	691.9782	95.14	324.1008
FGMM95_F14		5.264	0.037	11.1225	1.53	4.7141
FGMM95_F14		5.363	0.034	8.6443	1.19	3.7665
FGMM95_F14		5.741	0.038	15.6002	2.14	5.9502

Max Area% 95.138

UV Signal Purity>95% Pass

UV Apex Spectra

RT:

3.043

Sample Name:

FGMM95_F14

Signal Name:

DAD1C

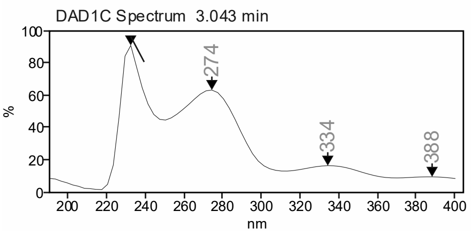

RT:

5.264

Sample Name:

FGMM95_F14

Signal Name:

DAD1C

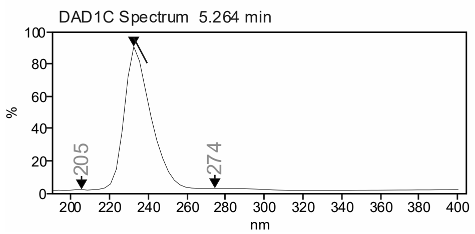

RT:

5.363

Sample Name:

FGMM95_F14

Signal Name:

DAD1C

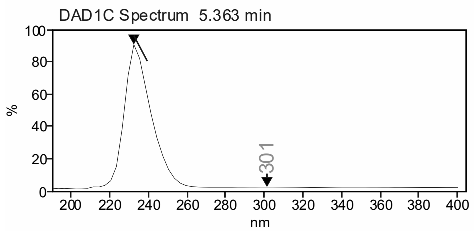

RT:

5.741

Sample Name:

FGMM95_F14

Signal Name:

DAD1C

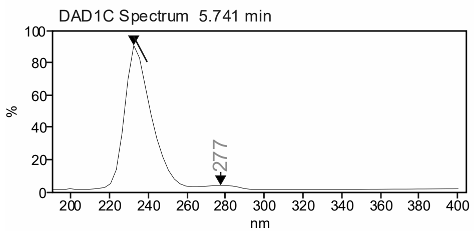

MSD Apex Spectra

RT:

3.068

Sample Name:

FGMM95_F14

Signal Name:

MSD1TIC

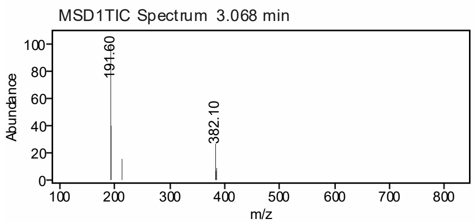

Compound 51

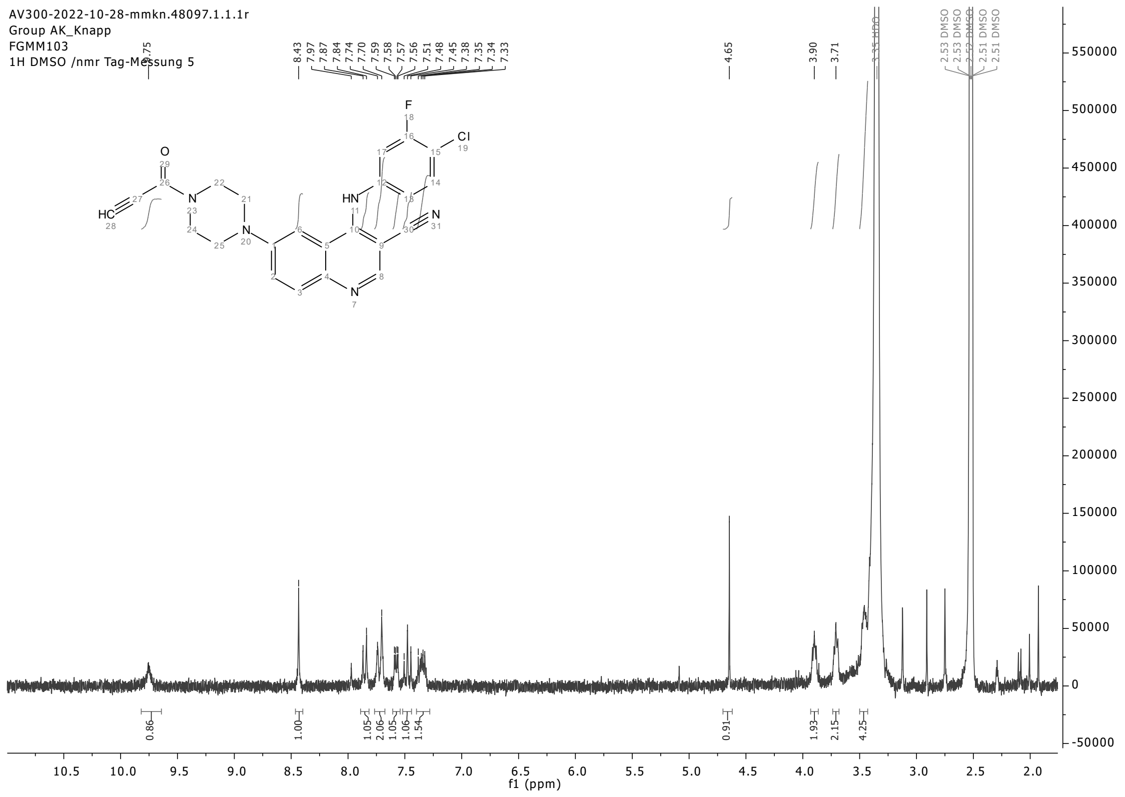

Sample Report

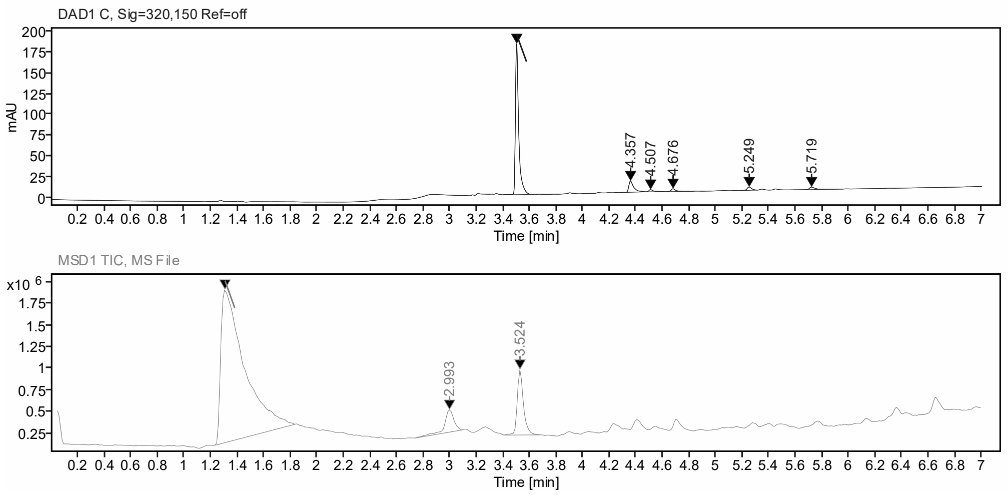

Sample Report

GOETHE UNIVERSITÄT FRANKFURT AM MAIN

Sample Purity

Signal Description DAD1 C, Sig=320,150 Ref=off

Sample Name	Name	RT	Width	Area	Area%	Height
FGMM103_F14-16_dry		3.500	0.024	308.5505	81.90	181.7772
FGMM103_F14-16_dry		4.357	0.036	35.6687	9.47	13.6065
FGMM103_F14-16_dry		4.507	0.031	6.2428	1.66	2.5695
FGMM103_F14-16_dry		4.676	0.032	8.4389	2.24	3.3743
FGMM103_F14-16_dry		5.249	0.037	9.5610	2.54	3.9290
FGMM103_F14-16_dry		5.719	0.044	8.2903	2.20	2.9432

Max Area% 81.897

UV Signal Purity>95% Fail

MSD Apex Spectra

RT:

1.303

Sample Name:

FGMM103_F14-16_dry

Signal Name:

MSD1TIC

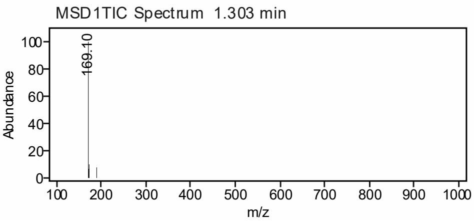

RT:

2.993

Sample Name:

FGMM103_F14-16_dry

Signal Name:

MSD1TIC

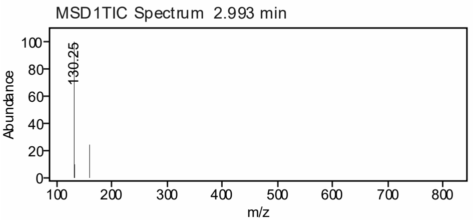

RT:

3.524

Sample Name:

FGMM103_F14-16_dry

Signal Name:

MSD1TIC

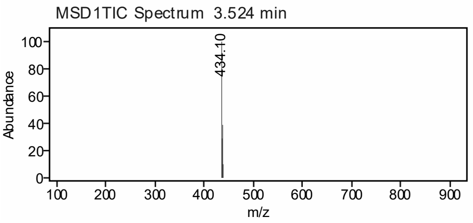

Compound 52

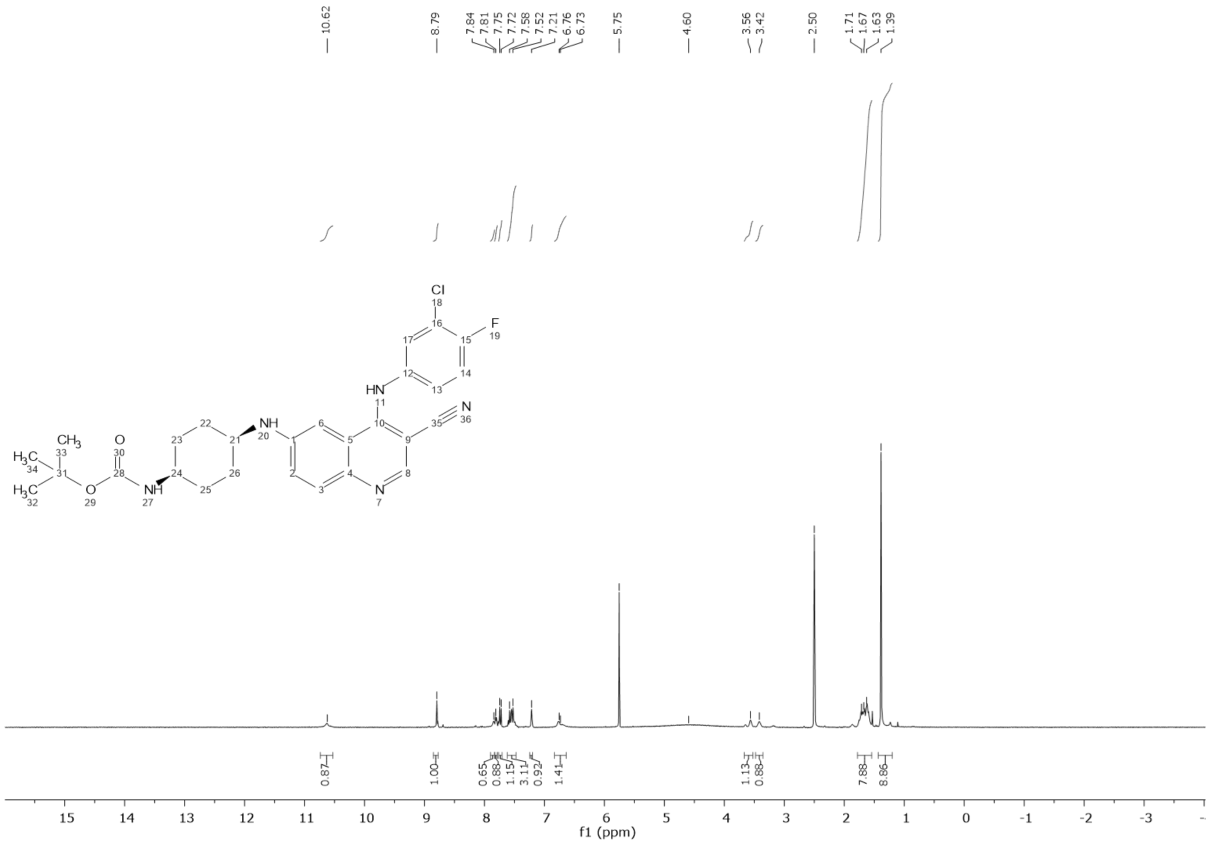

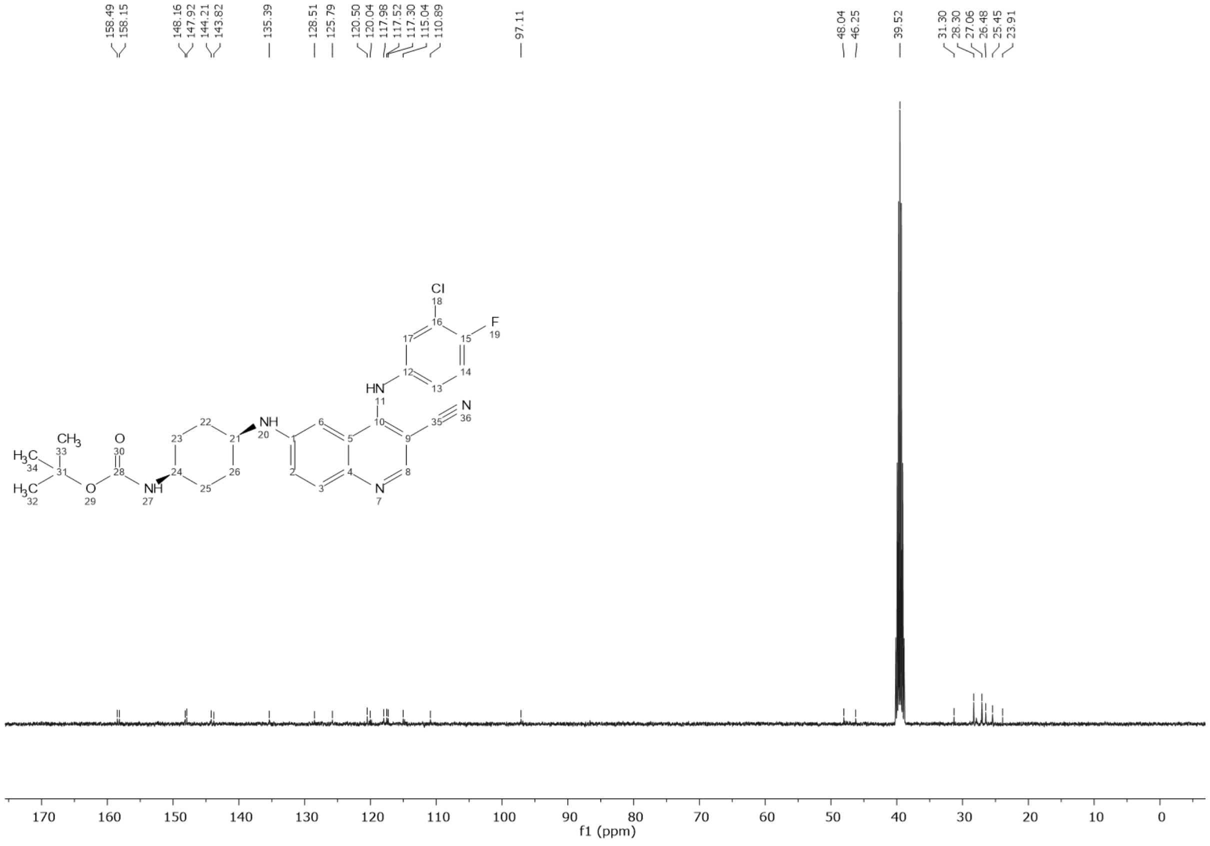

Sample Report

GOETHE UNIVERSITÄT FRANKFURT AM MAIN

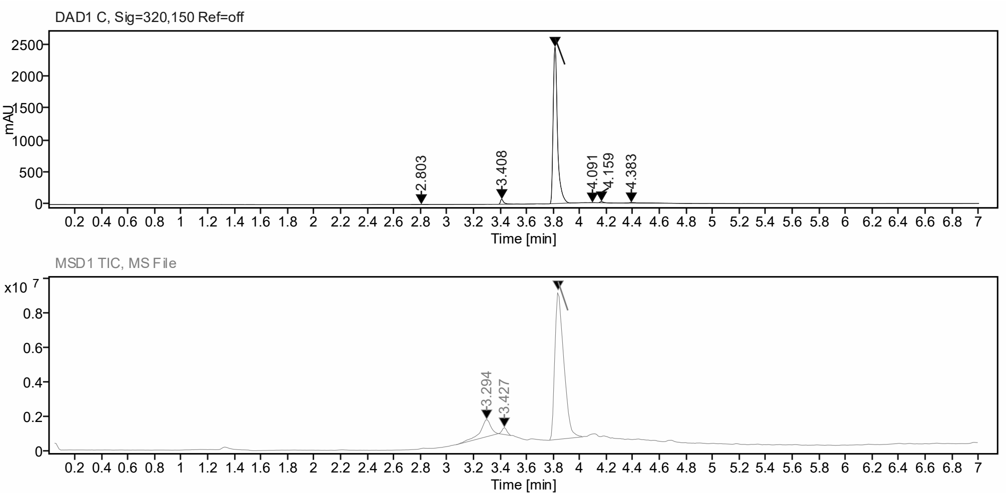

Sample Purity

Signal Description DAD1 C, Sig=320,150 Ref=off

Sample Name	Name	RT	Width	Area	Area%	Height
FGMM141_F15		2.803	0.036	6.8473	0.12	2.7464
FGMM141_F15		3.408	0.023	131.8636	2.26	78.1862
FGMM141_F15		3.809	0.033	5561.9272	95.52	2448.3313
FGMM141_F15		4.091	0.107	16.3210	0.28	2.2234
FGMM141_F15		4.159	0.025	36.9672	0.63	20.8879
FGMM141_F15		4.383	0.057	68.9300	1.18	9.0426

Max Area% 95.519

UV Signal Purity>95% Pass

UV Apex Spectra

Sample Report

RT:

4.383

Sample Name:

FGMM141_F15

Signal Name:

DAD1C

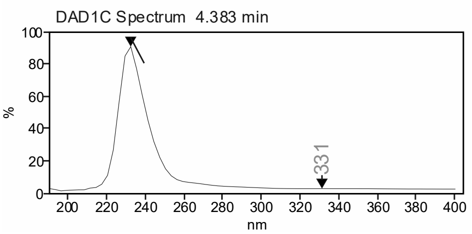

MSD Apex Spectra

RT:

3.294

Sample Name:

FGMM141_F15

Signal Name:

MSD1TIC

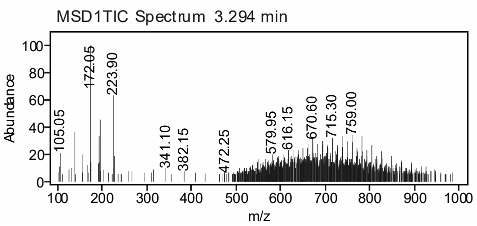

RT:

3.427

Sample Name:

FGMM141_F15

Signal Name:

MSD1TIC

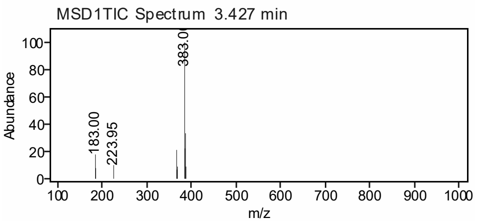

RT:

3.832

Sample Name:

FGMM141_F15

Signal Name:

MSD1TIC

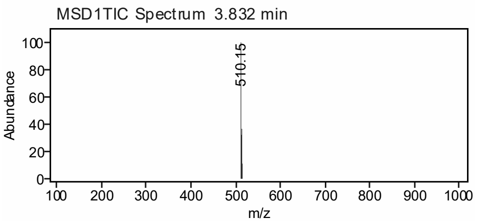

Compound 53

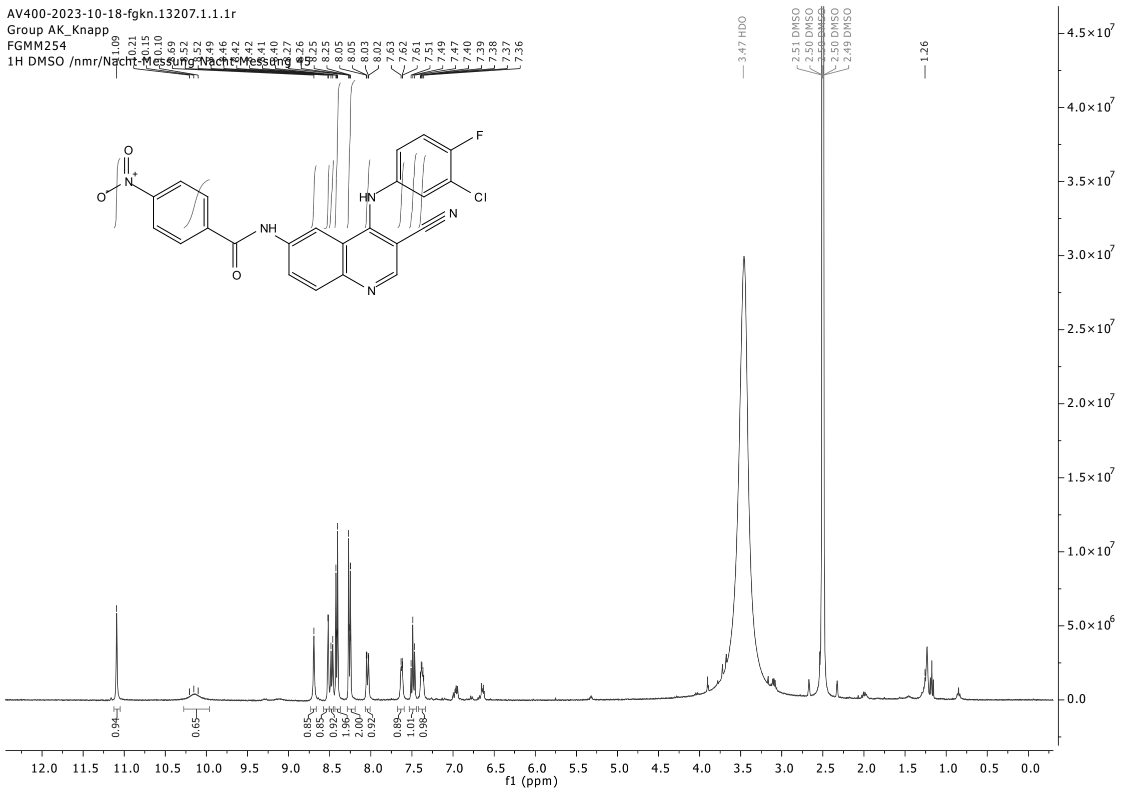
AV400-2023-10-18-fgkn.13207.1.1.1r
Group AK_Knapp
FGMM254
1H DMSO /nmr/Nacht-Messung Nacht-Messung 45
3.47 HDO
2.51 DMSO
2.50 DMSO
2.49 DMSO
1.26
f1 (ppm)

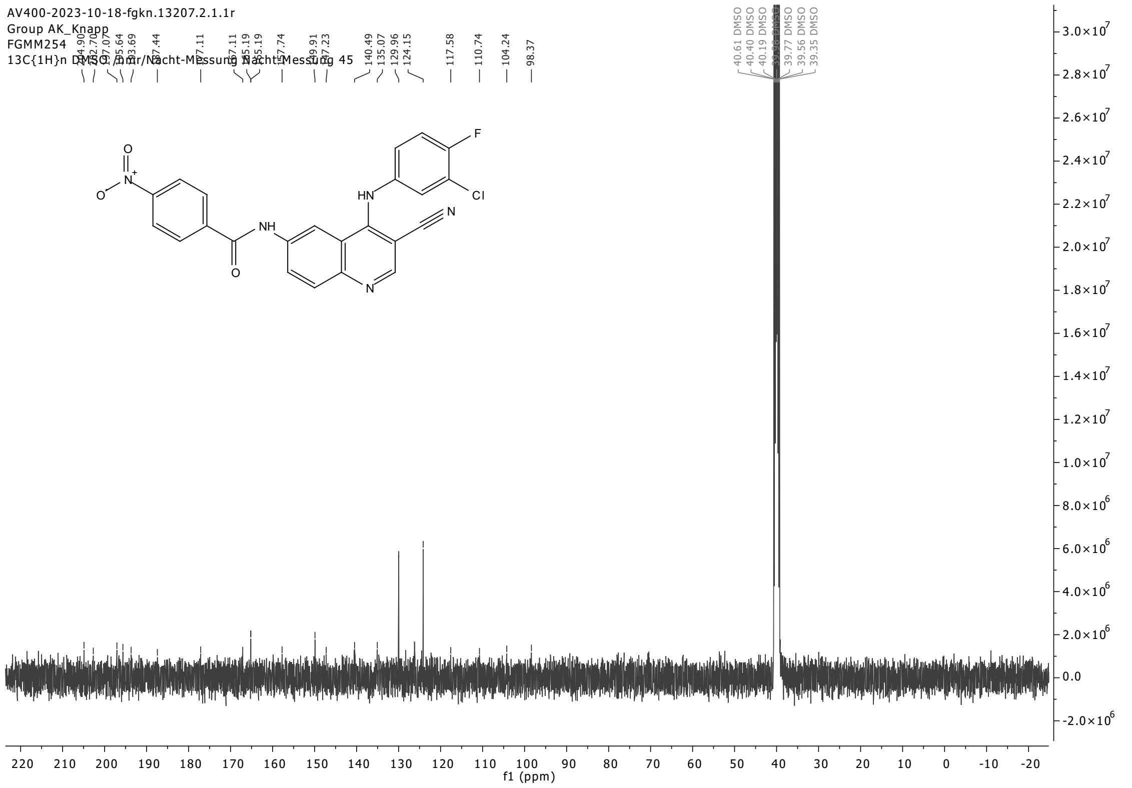
AV400-2023-10-18-fgkn.13207.2.1.1r
Group AK_Knapp
FGMM254
13C{1H}n DMSO /nmr/Nacht-Messung Nacht-Messung 45
140.49
135.07
129.96
124.15
117.58
110.74
104.24
98.37
40.61 DMSO
40.40 DMSO
40.19 DMSO
39.77 DMSO
39.56 DMSO
39.35 DMSO
f1 (ppm)

Sample Report

GOETHE UNIVERSITÄT FRANKFURT AM MAIN

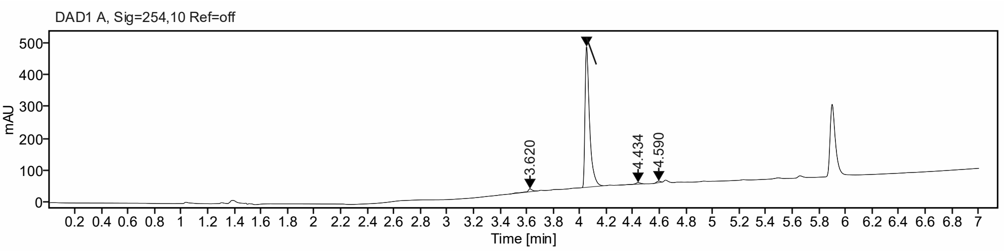

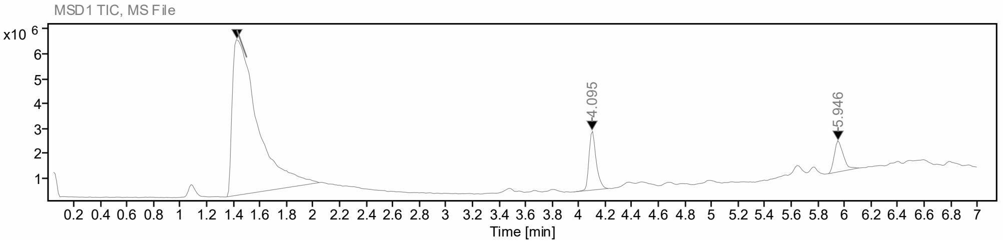

Sample Purity

Signal Description DAD1 A, Sig=254,10 Ref=off

Sample Name	Name	RT	Width	Area	Area%	Height
FGMM254		3.620	0.033	25.4216	2.31	9.1145
FGMM254		4.046	0.034	1052.9288	95.64	440.9478
FGMM254		4.434	0.040	13.2292	1.20	4.9540
FGMM254		4.590	0.035	9.3787	0.85	4.6986

Max Area% 95.637

UV Signal Purity>95% Pass

UV Apex Spectra

RT:

3.620

Sample Name:

FGMM254

Signal Name:

DAD1A

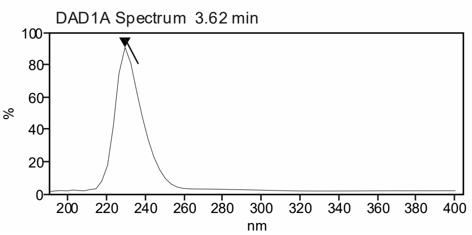

Sample Report

GOETHE UNIVERSITÄT FRANKFURT AM MAIN

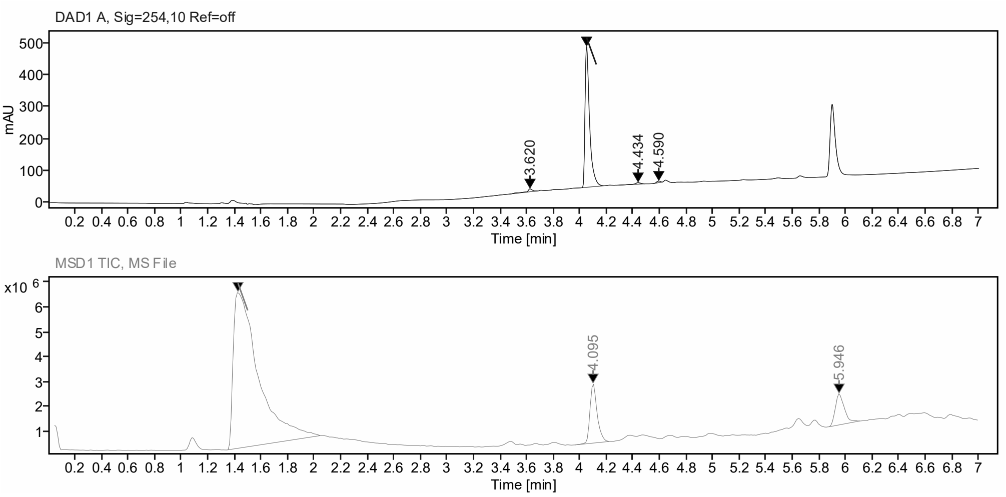

Sample Purity

Signal Description DAD1 A, Sig=254,10 Ref=off

Sample Name	Name	RT	Width	Area	Area%	Height
FGMM254		3.620	0.033	25.4216	2.31	9.1145
FGMM254		4.046	0.034	1052.9288	95.64	440.9478
FGMM254		4.434	0.040	13.2292	1.20	4.9540
FGMM254		4.590	0.035	9.3787	0.85	4.6986

Max Area% 95.637

UV Signal Purity>95% Pass

UV Apex Spectra

RT:

3.620

Sample Name:

FGMM254

Signal Name:

DAD1A

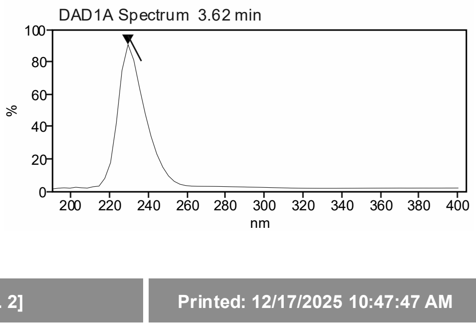

RT:

4.095

Sample Name:

FGMM254

Signal Name:

MSD1TIC

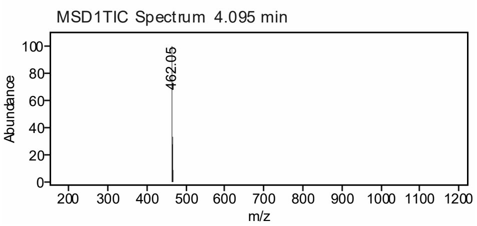

RT:

5.946

Sample Name:

FGMM254

Signal Name:

MSD1TIC

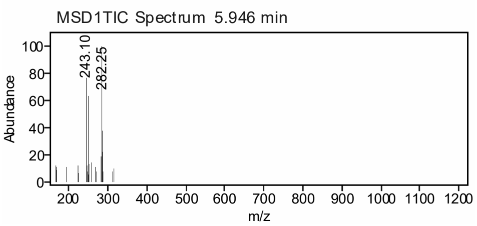

Compound S2

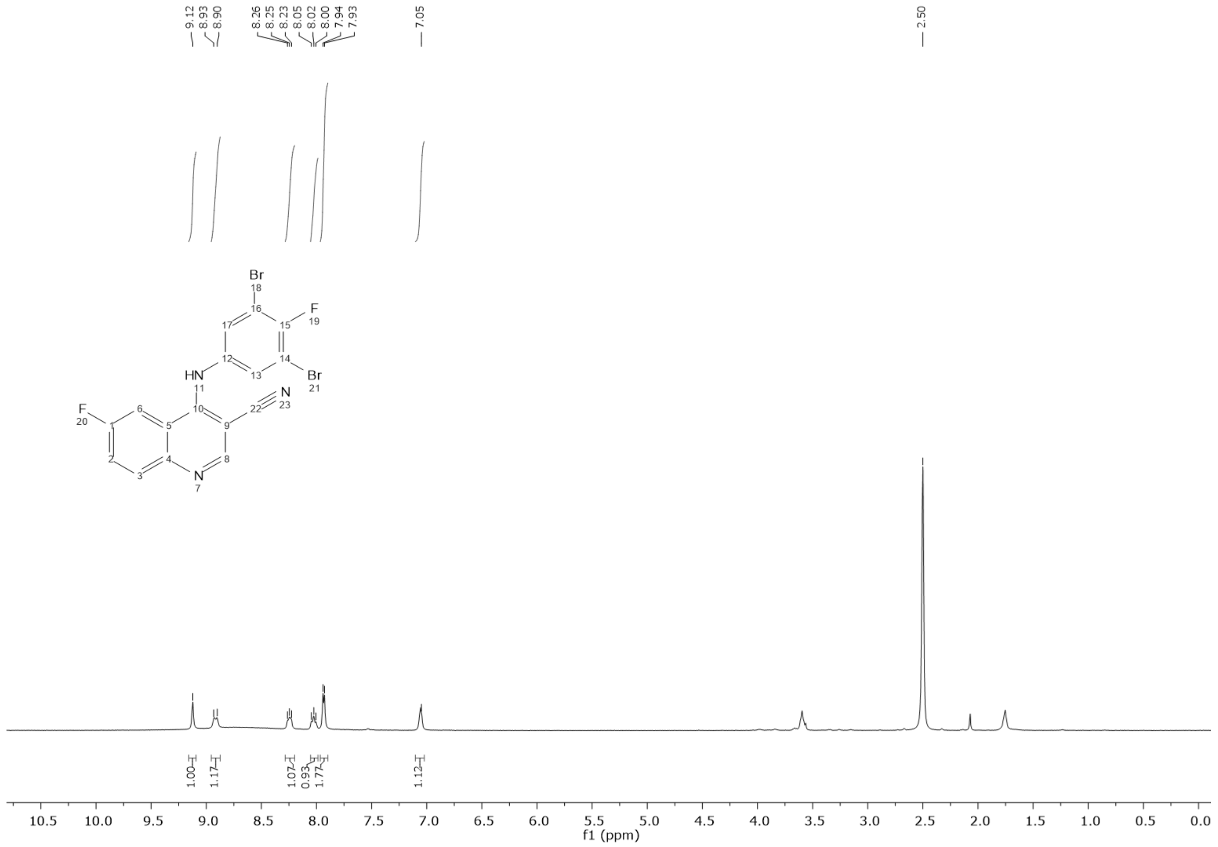

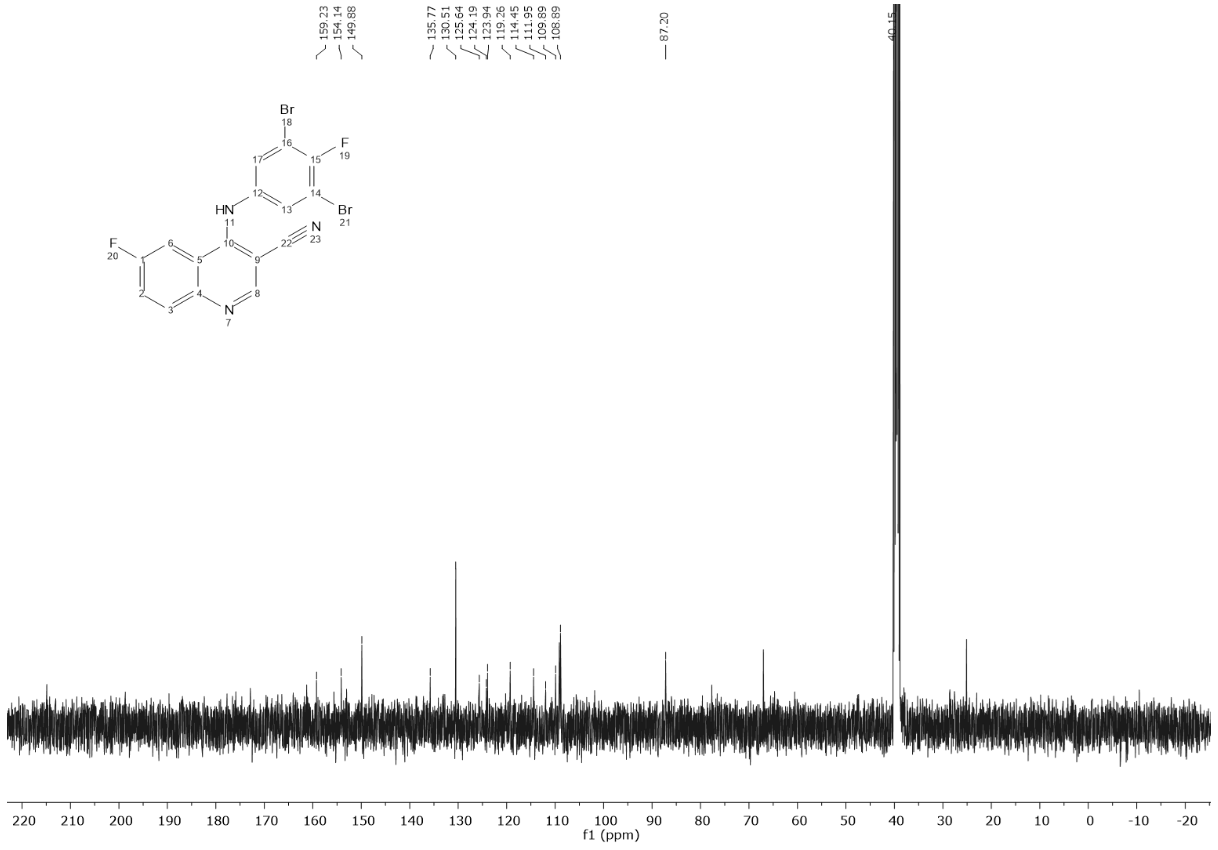

Sample Report

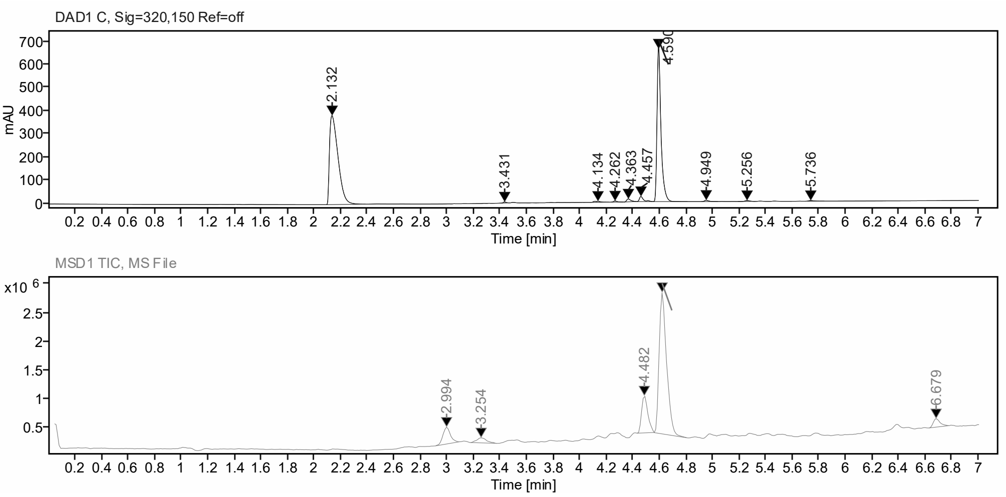

Sample Purity

Signal Description DAD1 C, Sig=320,150 Ref=off

Sample Name	Name	RT	Width	Area	Area%	Height
FGMM107_dry		2.132	0.069	1710.9692	52.91	384.4010
FGMM107_dry		3.431	0.023	6.9919	0.22	4.6701
FGMM107_dry		4.134	0.055	15.6632	0.48	4.4388
FGMM107_dry		4.262	0.028	10.0034	0.31	5.1395
FGMM107_dry		4.363	0.034	27.5921	0.85	12.5789
FGMM107_dry		4.457	0.028	47.2112	1.46	21.7242
FGMM107_dry		4.590	0.030	1384.2659	42.80	659.4569
FGMM107_dry		4.949	0.033	13.1190	0.41	5.8239
FGMM107_dry		5.256	0.037	9.9578	0.31	4.0751
FGMM107_dry		5.736	0.041	8.2201	0.25	2.9787

Max Area% 52.906
UV Signal Purity>95% Fail

UV Apex Spectra

GOETHE UNIVERSITÄT FRANKFURT AM MAIN

MSD Apex Spectra

RT:

2.994

Sample Name:

FGMM107_dry

Signal Name:

MSD1TIC

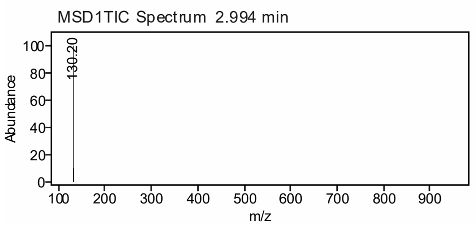

RT:

3.254

Sample Name:

FGMM107_dry

Signal Name:

MSD1TIC

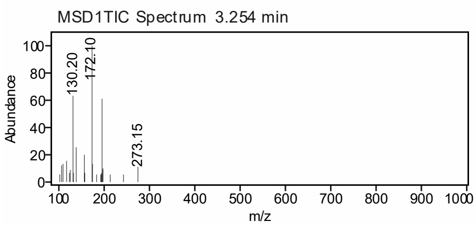

RT:

4.482

Sample Name:

FGMM107_dry

Signal Name:

MSD1TIC

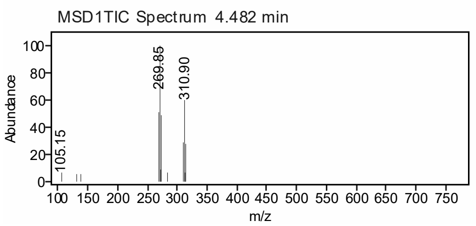

RT:

4.616

Sample Name:

FGMM107_dry

Signal Name:

MSD1TIC

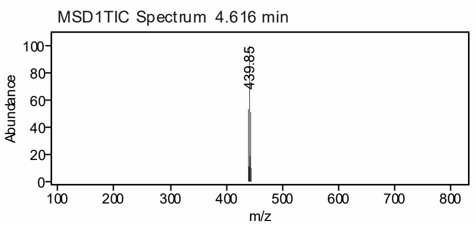

Compound 54

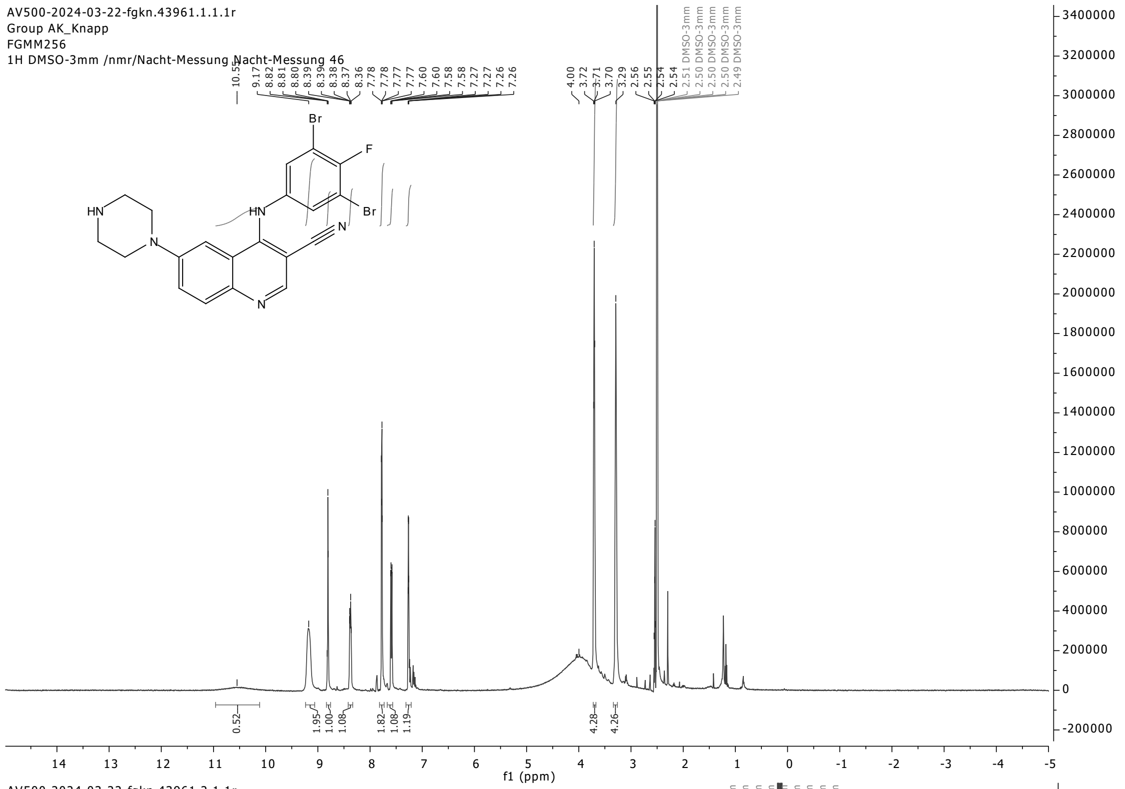

AV500-2024-03-22-fgkn.43961.1.1.1r
Group AK_Knapp
FGMM256
1H DMSO-3mm /nmr/Nacht-Messung Nacht-Messung 46
f1 (ppm)

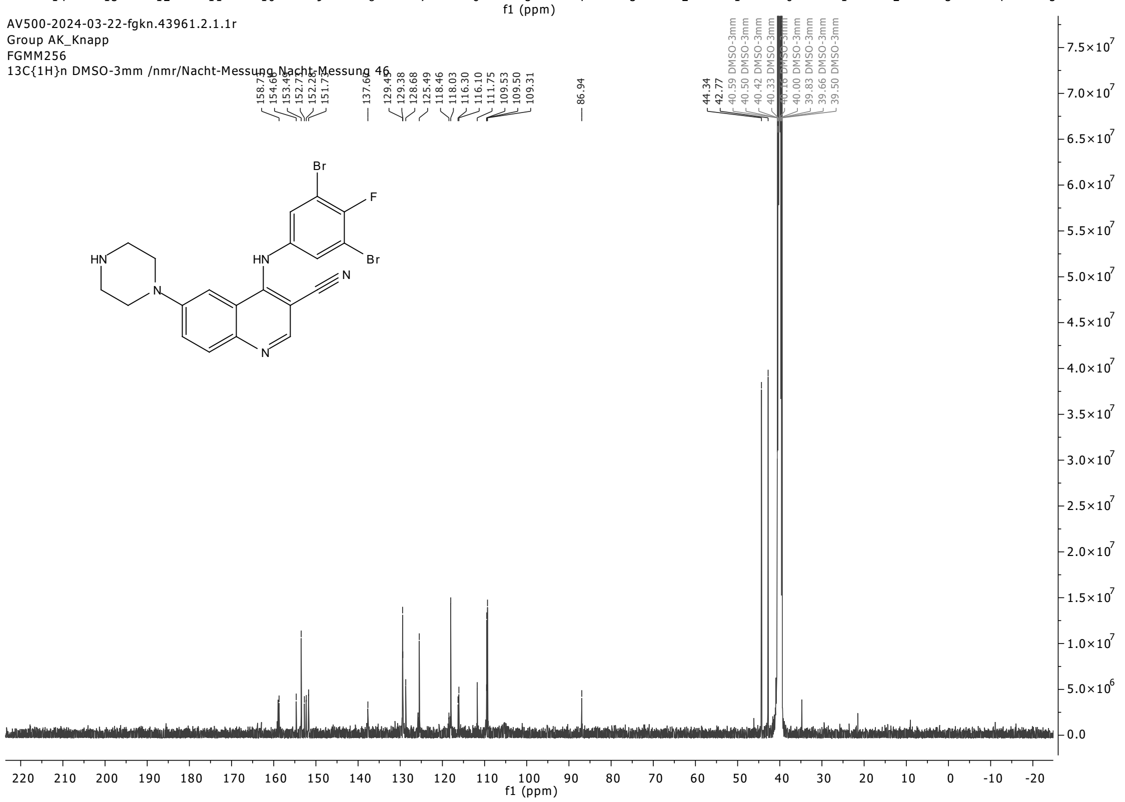

AV500-2024-03-22-fgkn.43961.2.1.1r
Group AK_Knapp
FGMM256
13C{1H}n DMSO-3mm /nmr/Nacht-Messung Nacht-Messung 46
f1 (ppm)

Sample Report

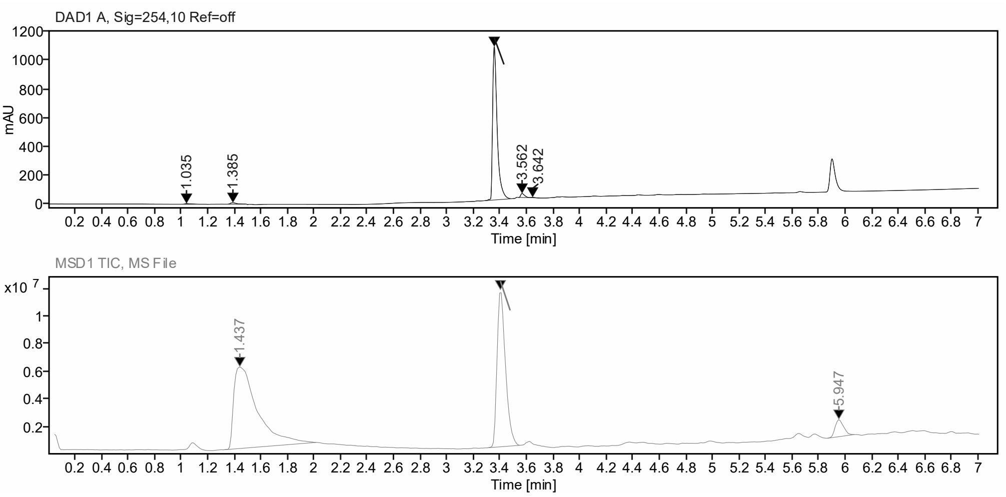

Sample Purity

Signal Description DAD1 A, Sig=254,10 Ref=off

Sample Name	Name	RT	Width	Area	Area%	Height
FGMM262		1.035	0.049	14.7787	0.56	4.5412
FGMM262		1.385	0.046	30.6531	1.16	9.9524
FGMM262		3.353	0.035	2536.6619	95.97	1069.3068
FGMM262		3.562	0.031	55.8426	2.11	27.9132
FGMM262		3.642	0.028	5.1829	0.20	2.6598

Max Area% 95.972

UV Signal Purity>95% Pass

UV Apex Spectra

MSD Apex Spectra

RT:

1.437

Sample Name:

FGMM262

Signal Name:

MSD1TIC

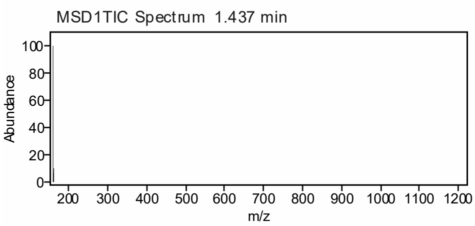

RT:

3.399

Sample Name:

FGMM262

Signal Name:

MSD1TIC

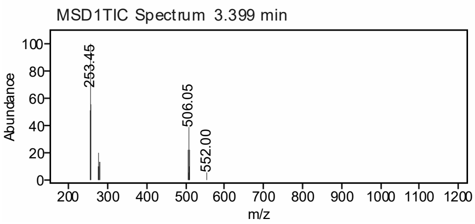

RT:

5.947

Sample Name:

FGMM262

Signal Name:

MSD1TIC

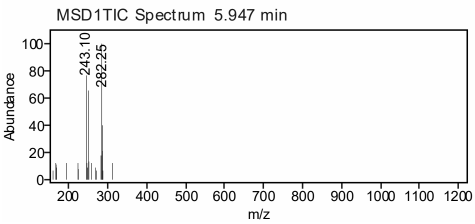

Mass Spectrum SmartFormula Report

Analysis Info

Analysis Name	D:\Data\MS_Service\2024Q2\20240618_SKN_FG\SKN_FG_000049_FGMM262_1.d	Acquisition Date	6/20/2024 10:52:35 AM
Method	Positive(100-2000)_Infusion_200uL-min.m	Operator	BDAL@DE
Sample Name	Test1	Instrument	micrOTOF-Q 228888.10407
Comment			

Acquisition Parameter

Source Type	ESI	Ion Polarity	Positive	Set Nebulizer	1.8 Bar
Focus	Active	Set Capillary	4000 V	Set Dry Heater	250 °C
Scan Begin	100 m/z	Set End Plate Offset	-500 V	Set Dry Gas	8.0 l/min
Scan End	2000 m/z	Set Collision Cell RF	150.0 Vpp	Set Divert Valve	Waste

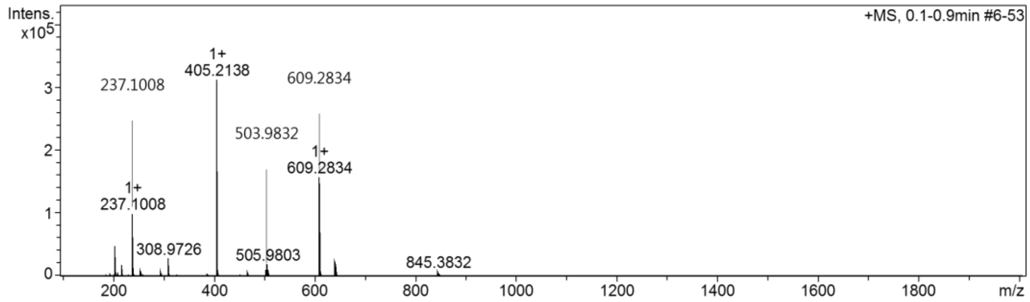

Compound S3

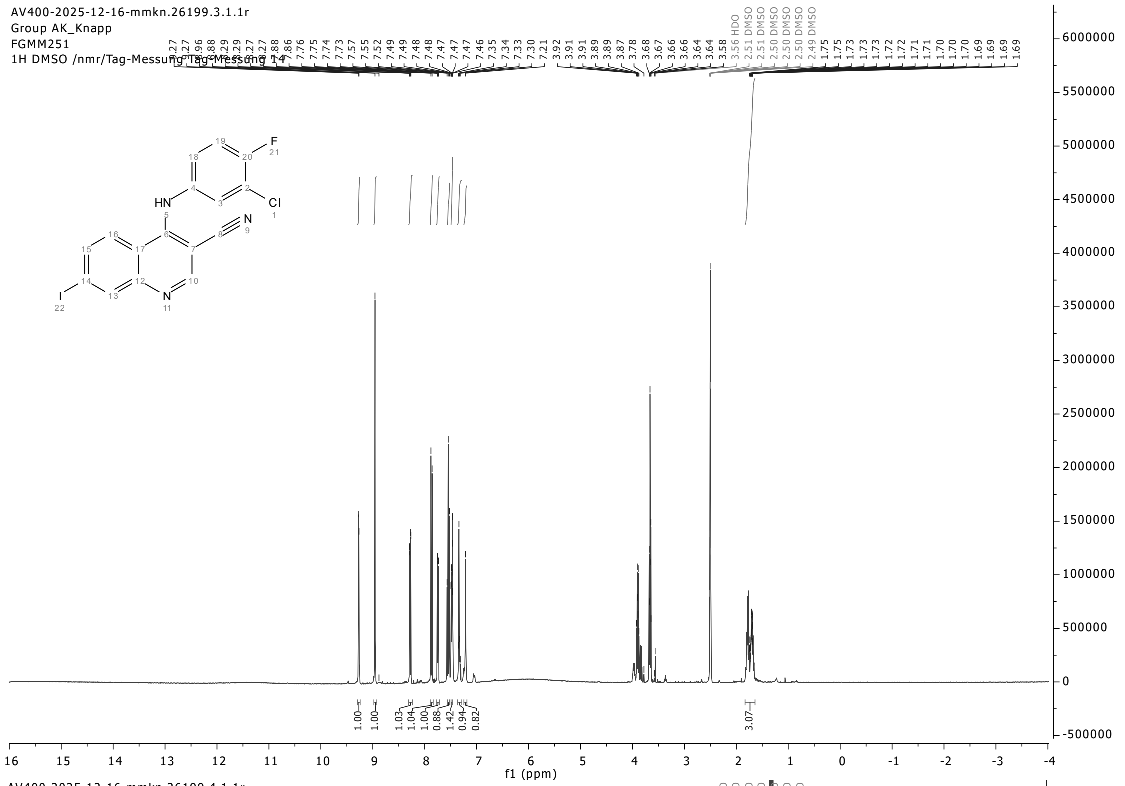
AV400-2025-12-16-mmkn.26199.3.1.1r
Group AK_Knapp
FGMM251
1H DMSO /nmr/Tag-Messung Tag-Messung 14
f1 (ppm)

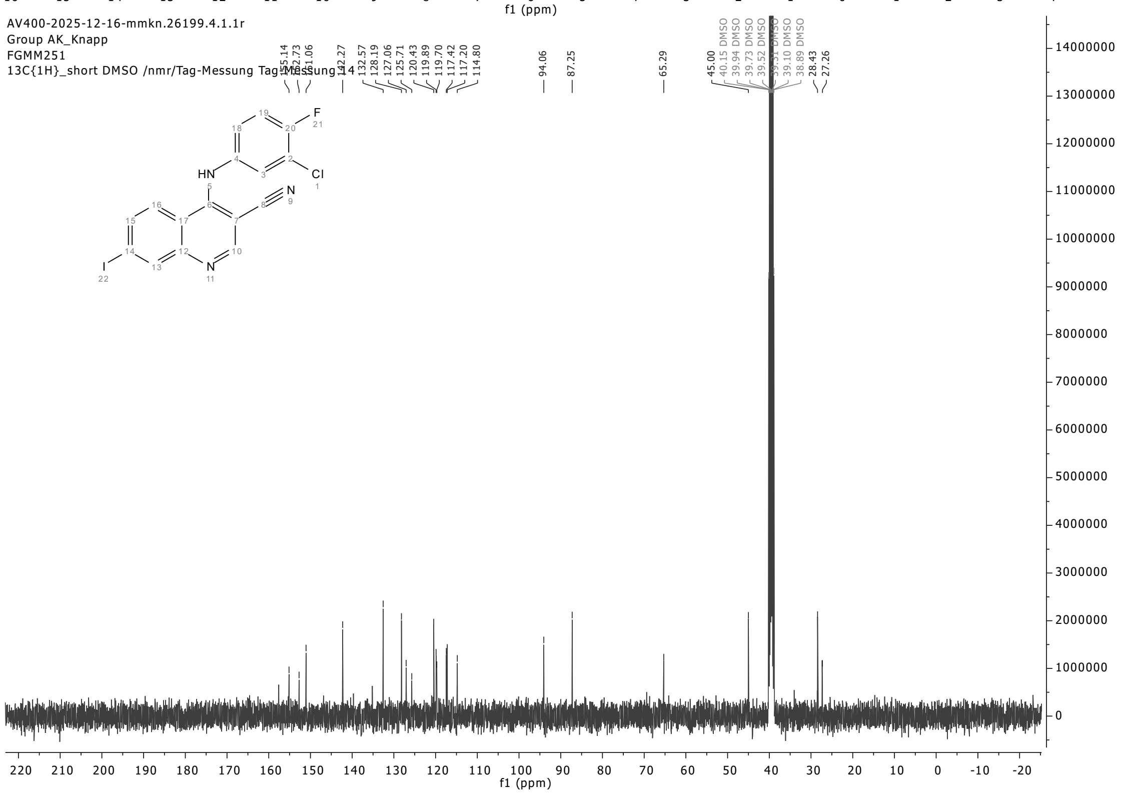
AV400-2025-12-16-mmkn.26199.4.1.1r
Group AK_Knapp
FGMM251
13C{1H}_short DMSO /nmr/Tag-Messung Tag-Messung 14
f1 (ppm)

Sample Report

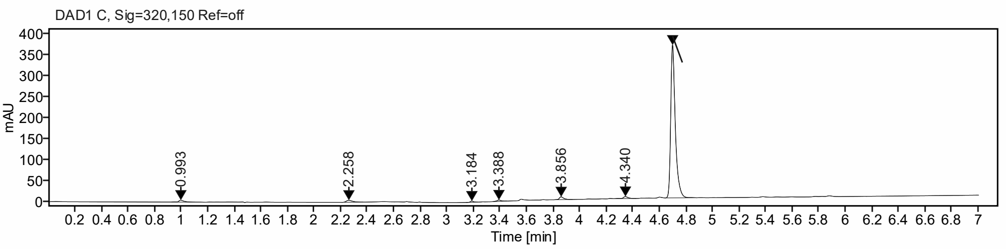

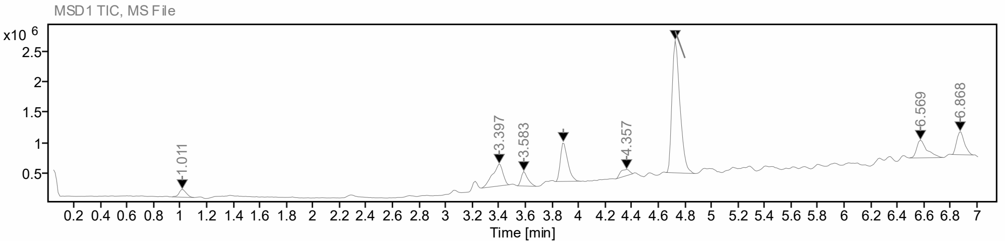

Sample Purity

Signal Description DAD1 C, Sig=320,150 Ref=off

Sample Name	Name	RT	Width	Area	Area%	Height
FGMM251_dry		0.993	0.039	14.4480	1.52	4.6007
FGMM251_dry		2.258	0.050	15.4587	1.63	4.6663
FGMM251_dry		3.184	0.026	5.1644	0.54	2.9089
FGMM251_dry		3.388	0.034	8.0271	0.85	3.3611
FGMM251_dry		3.856	0.035	15.6196	1.65	6.6519
FGMM251_dry		4.340	0.036	11.8755	1.25	4.7047
FGMM251_dry		4.695	0.035	878.8159	92.56	365.4074

Max Area% 92.565

UV Signal Purity>95% Fail

UV Apex Spectra

GOETHE UNIVERSITÄT FRANKFURT AM MAIN

RT:

3.583

Sample Name:

FGMM251_dry

Signal Name:

MSD1TIC

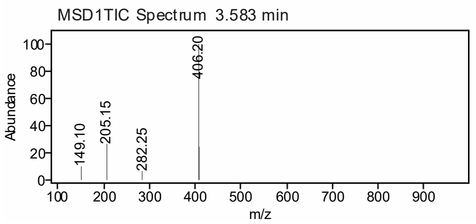

RT:

3.880

Sample Name:

FGMM251_dry

Signal Name:

MSD1TIC

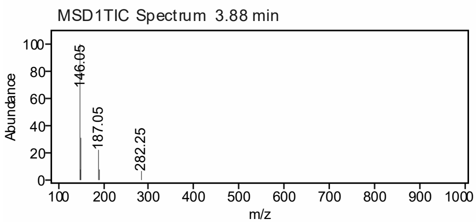

RT:

4.357

Sample Name:

FGMM251_dry

Signal Name:

MSD1TIC

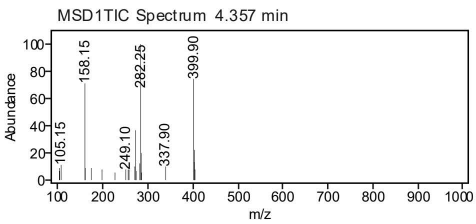

RT:

4.722

Sample Name:

FGMM251_dry

Signal Name:

MSD1TIC

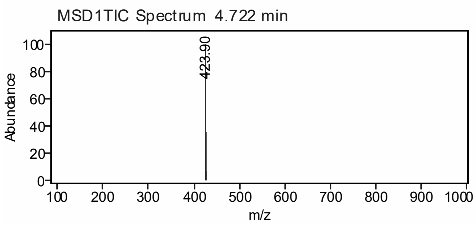

RT:

6.569

Sample Name:

FGMM251_dry

Signal Name:

MSD1TIC

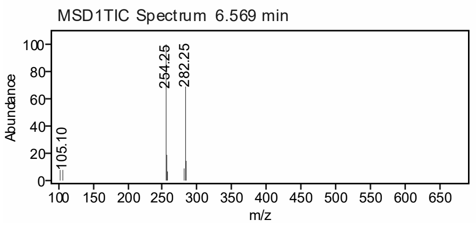

Compound 55

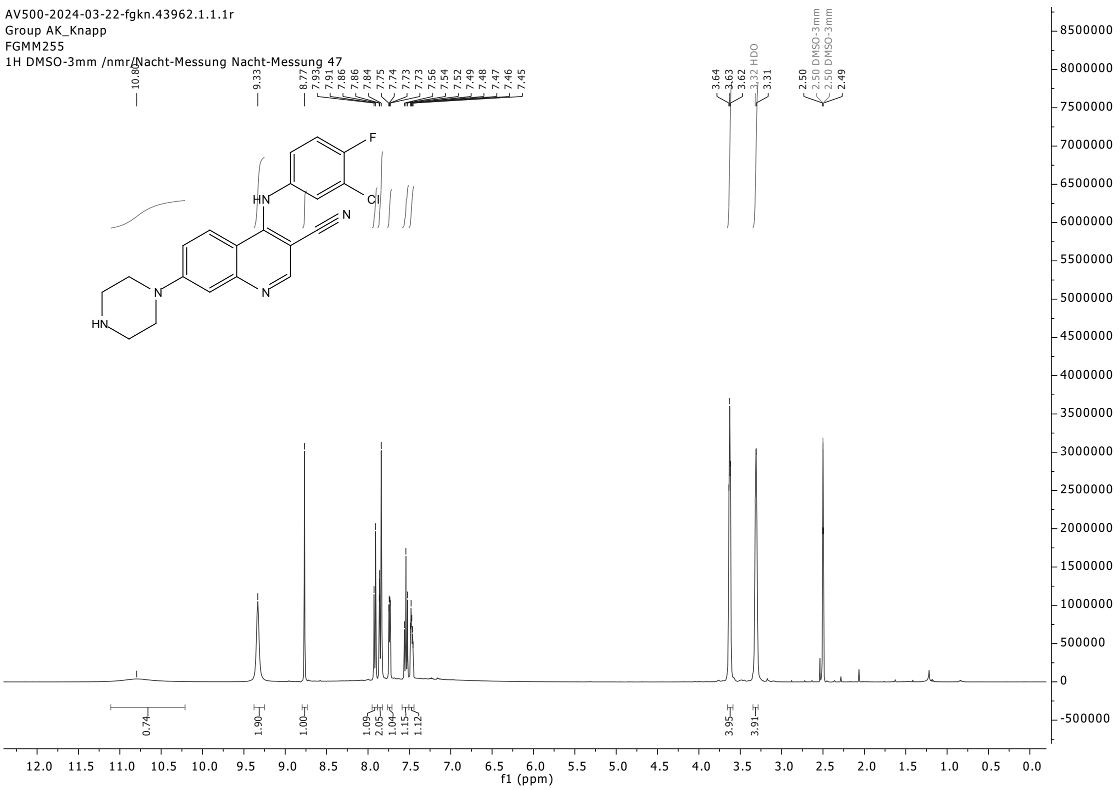

AV500-2024-03-22-fgkn.43962.1.1.1r
Group AK_Knapp
FGMM255
1H DMSO-3mm /nmr/Nacht-Messung Nacht-Messung 47
10.80
9.33
8.77
7.93
7.91
7.86
7.84
7.75
7.74
7.73
7.73
7.56
7.54
7.52
7.49
7.48
7.47
7.46
7.45
3.64
3.63
3.62
3.32 HDO
3.31
2.50
2.50 DMSO-3mm
2.50 DMSO-3mm
2.49
F
HN
Cl
N
N
N
HN
0.74
1.90
1.00
1.09
2.05
1.04
1.15
1.12
3.95
3.91
f1 (ppm)

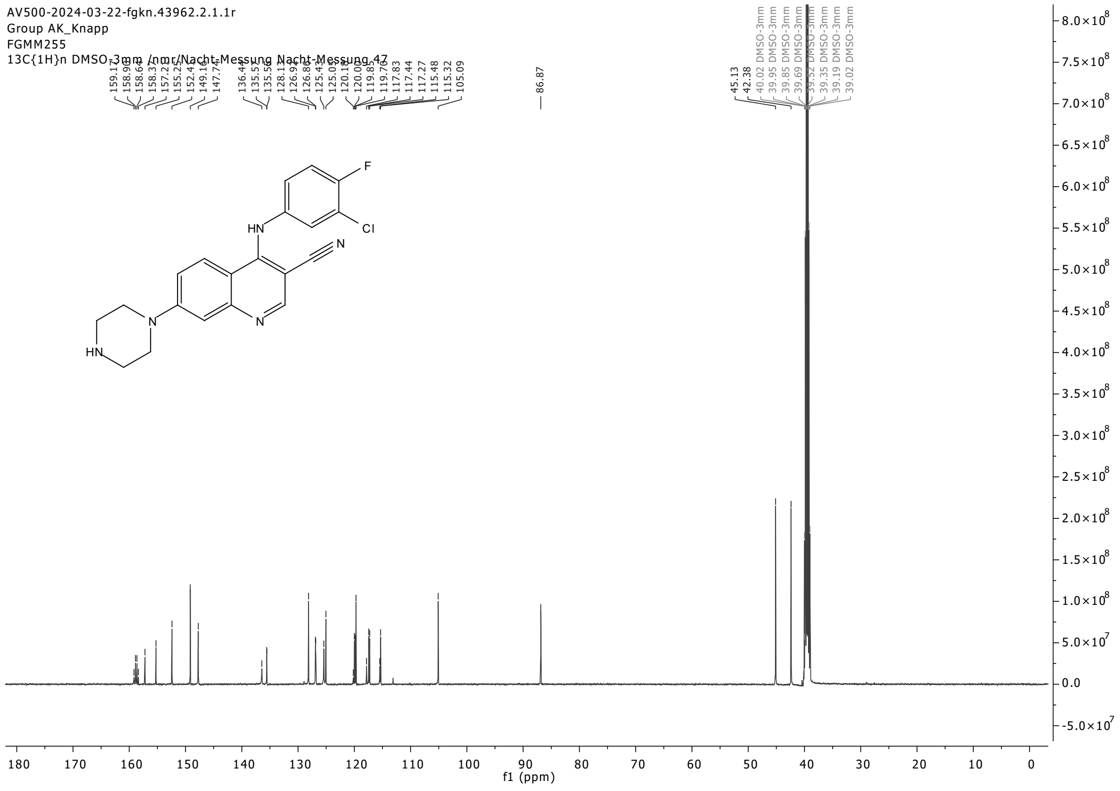

AV500-2024-03-22-fgkn.43962.2.1.1r
Group AK_Knapp
FGMM255
13C{1H}n DMSO-3mm /nmr/Nacht-Messung Nacht-Messung 47
86.87
45.13
42.38
40.02 DMSO-3mm
39.95 DMSO-3mm
39.85 DMSO-3mm
39.69 DMSO-3mm
39.52 DMSO-3mm
39.35 DMSO-3mm
39.19 DMSO-3mm
39.02 DMSO-3mm
F
HN
Cl
N
N
N
HN
f1 (ppm)

Sample Report

GOETHE UNIVERSITÄT FRANKFURT AM MAIN

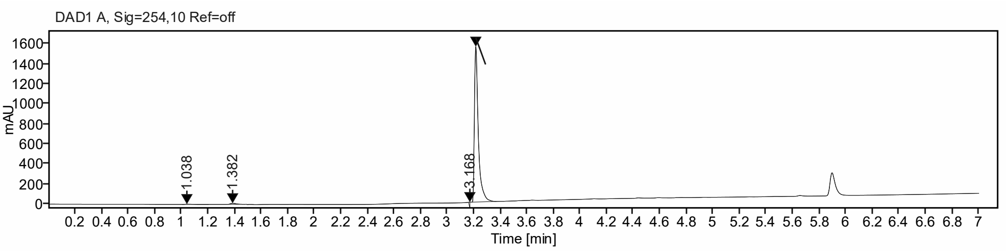

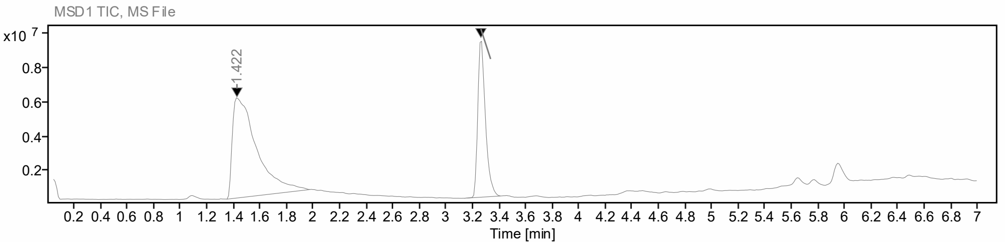

Sample Purity

Signal Description DAD1 A, Sig=254,10 Ref=off

Sample Name	Name	RT	Width	Area	Area%	Height
FGMM261		1.038	0.060	8.9832	0.28	2.4608
FGMM261		1.382	0.047	32.0726	0.99	10.3347
FGMM261		3.168	0.009	19.5259	0.60	58.7587
FGMM261		3.213	0.029	3172.9900	98.13	1552.5470

Max Area% 98.126

UV Signal Purity>95% Pass

UV Apex Spectra

RT:

1.038

Sample Name:

FGMM261

Signal Name:

DAD1A

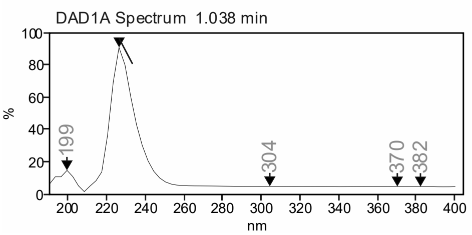

GOETHE UNIVERSITÄT FRANKFURT AM MAIN

RT:

3.258

Sample Name:

FGMM261

Signal Name:

MSD1TIC

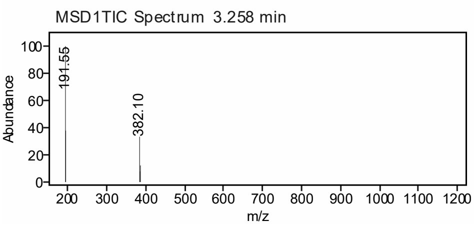

Mass Spectrum SmartFormula Report

Analysis Info

Analysis Name	D:\Data\MS_Service\2024Q2\20240618_SKN_FG\SKN_FG_000048_FGMM261_1.d	Acquisition Date	6/20/2024 10:44:26 AM
Method	Positive(100-2000)_Infusion_200uL-min.m	Operator	BDAL@DE
Sample Name	Test1	Instrument	micrOTOF-Q 228888.10407
Comment			

Acquisition Parameter

Source Type	ESI	Ion Polarity	Positive	Set Nebulizer	1.8 Bar
Focus	Active	Set Capillary	4000 V	Set Dry Heater	250 °C
Scan Begin	100 m/z	Set End Plate Offset	-500 V	Set Dry Gas	8.0 l/min
Scan End	2000 m/z	Set Collision Cell RF	150.0 Vpp	Set Divert Valve	Waste

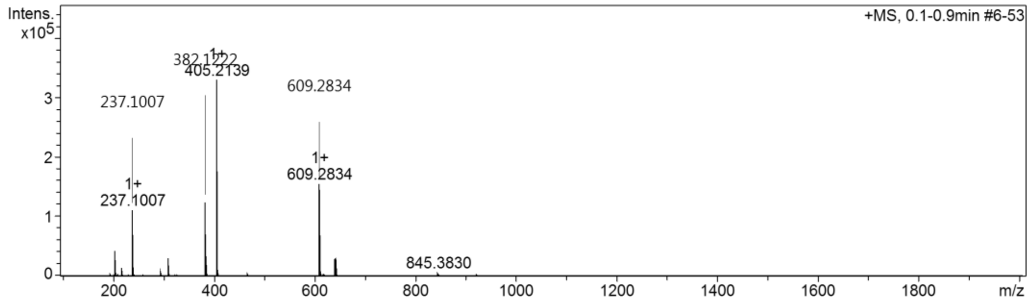

Compound S4

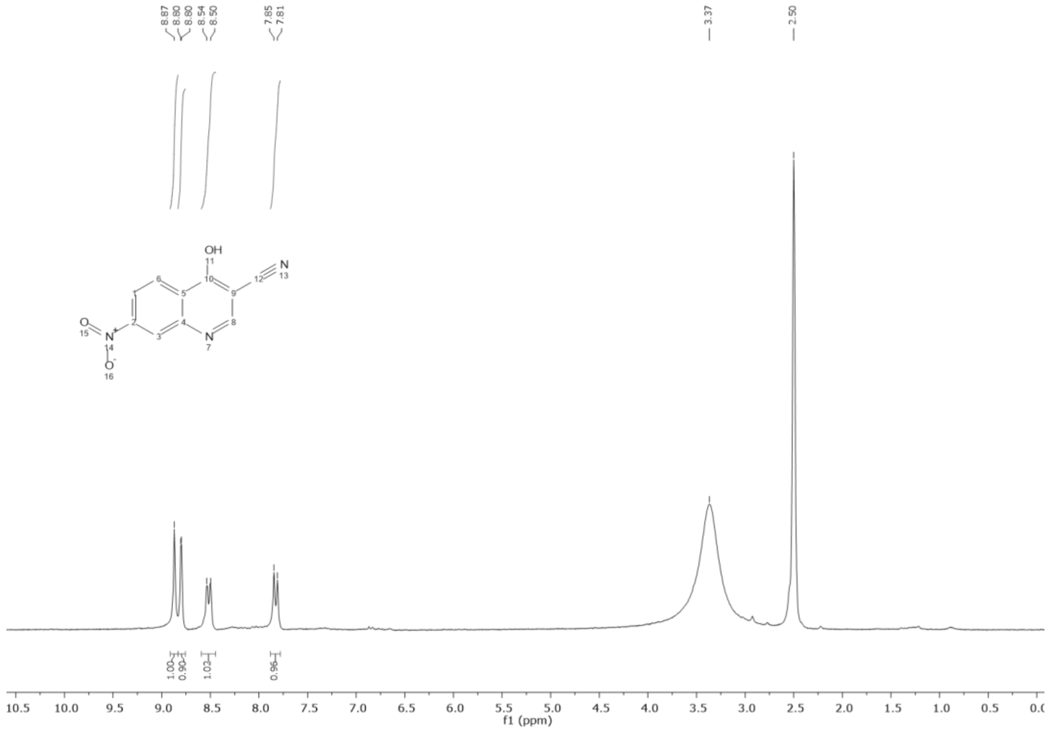
8.87
8.80
8.80
8.54
8.50
7.85
7.81
3.37
2.50
1.00
0.90
1.03
0.96
f1 (ppm)

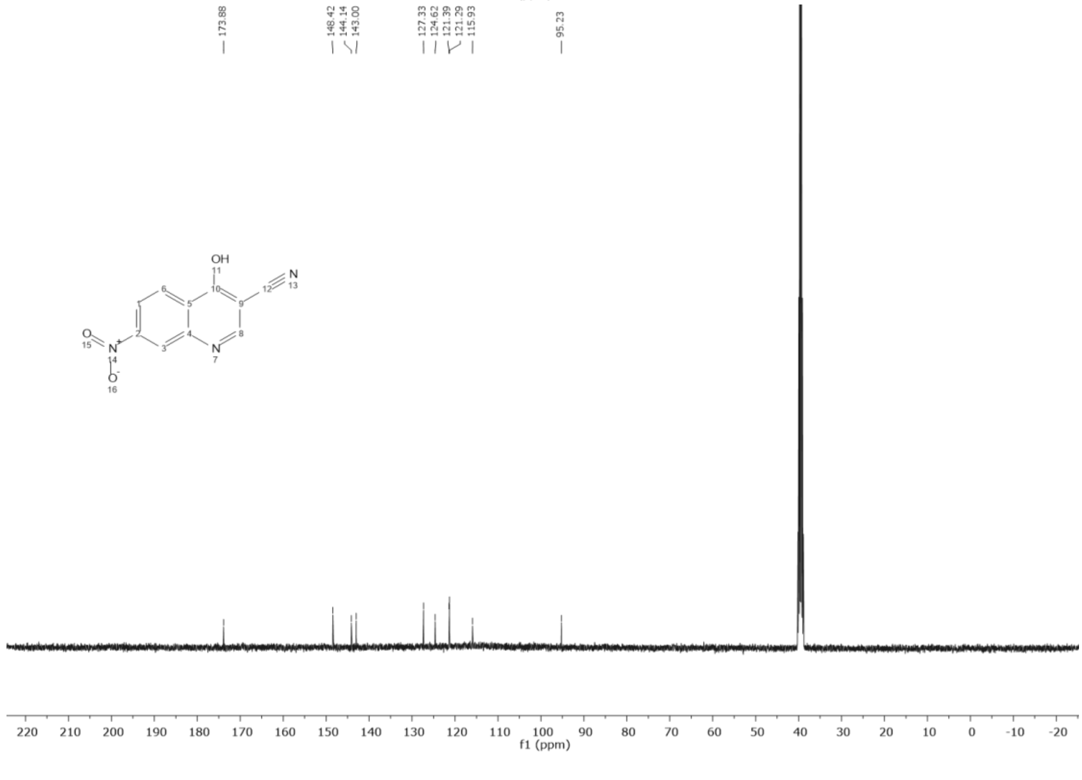
173.88
148.42
144.14
143.00
127.33
124.62
121.39
121.29
115.93
95.23
f1 (ppm)

GOETHE UNIVERSITÄT FRANKFURT AM MAIN

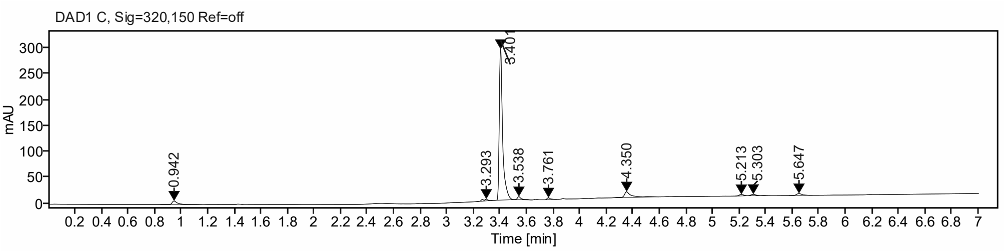

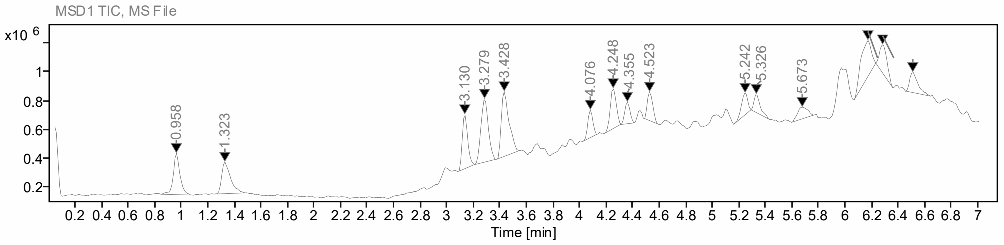

Sample Purity

Signal Description DAD1 C, Sig=320,150 Ref=off

Sample Name	Name	RT	Width	Area	Area%	Height
FGMM12		0.942	0.039	21.2122	3.37	6.8648
FGMM12		3.293	0.025	8.9747	1.42	3.1741
FGMM12		3.401	0.024	515.1714	81.76	291.3107
FGMM12		3.538	0.025	11.6312	1.85	5.8522
FGMM12		3.761	0.026	6.2594	0.99	3.3979
FGMM12		4.350	0.043	42.0600	6.67	11.0557
FGMM12		5.213	0.042	6.6416	1.05	2.6351
FGMM12		5.303	0.039	7.1337	1.13	2.5768
FGMM12		5.647	0.044	11.0448	1.75	3.7085

Max Area% 81.756

UV Signal Purity>95% Fail

UV Apex Spectra

RT:

0.958

Sample Name:

FGMM12

Signal Name:

MSD1TIC

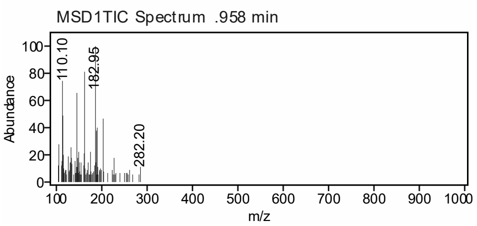

RT:

1.323

Sample Name:

FGMM12

Signal Name:

MSD1TIC

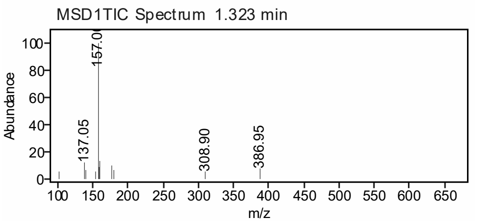

RT:

3.130

Sample Name:

FGMM12

Signal Name:

MSD1TIC

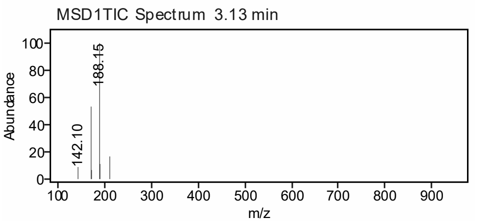

RT:

3.279

Sample Name:

FGMM12

Signal Name:

MSD1TIC

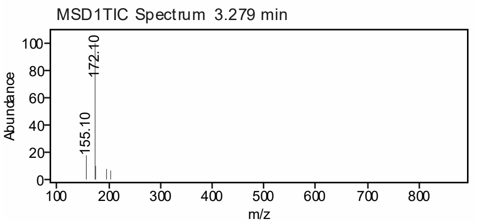

RT:

3.428

Sample Name:

FGMM12

Signal Name:

MSD1TIC

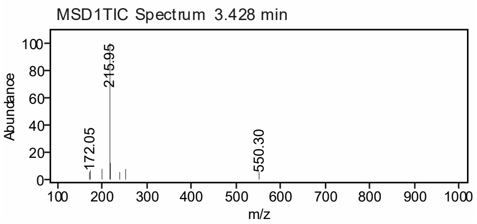

Compound S5

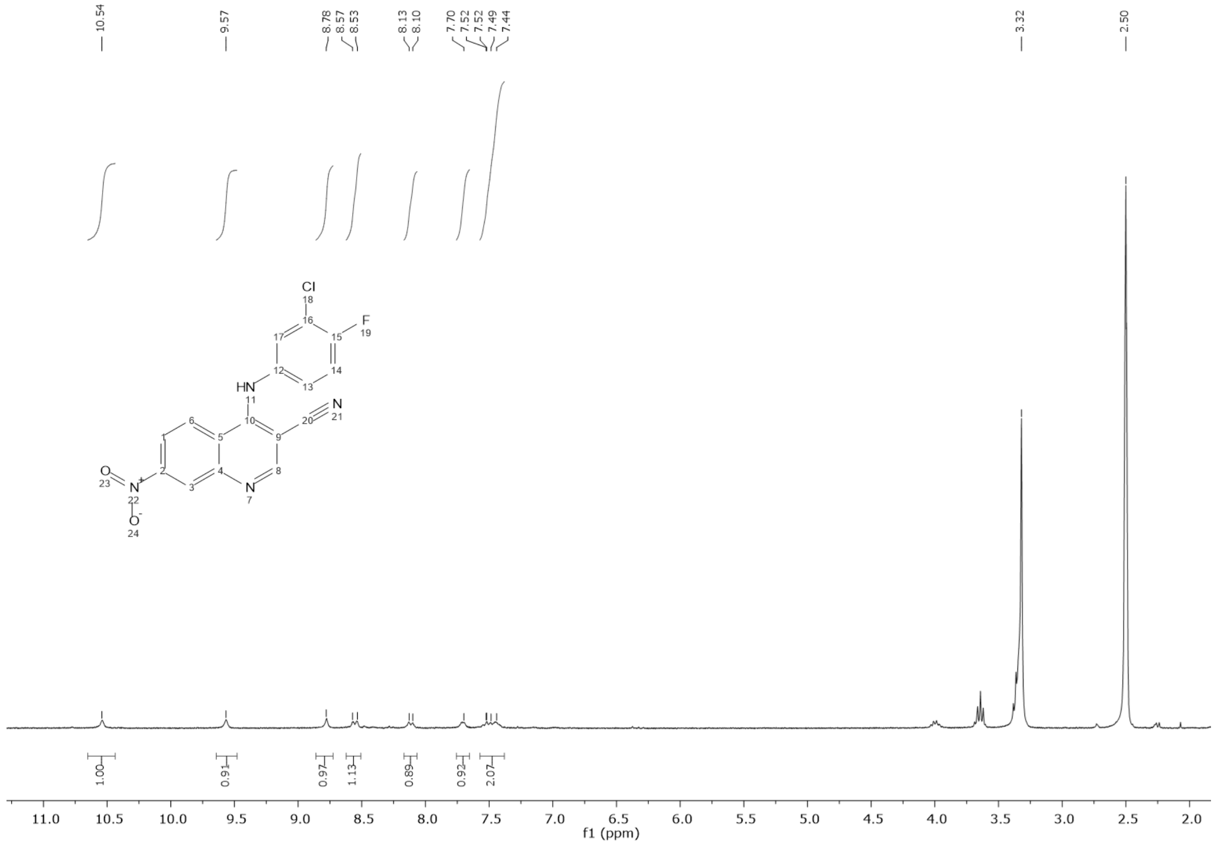

10.54
9.57
8.78
8.57
8.53
8.13
8.10
7.70
7.52
7.52
7.49
7.44
3.32
2.50
Cl
F
HN
N
O
N+
O-
1.00
0.91
0.97
1.13
0.89
0.92
2.07
f1 (ppm)

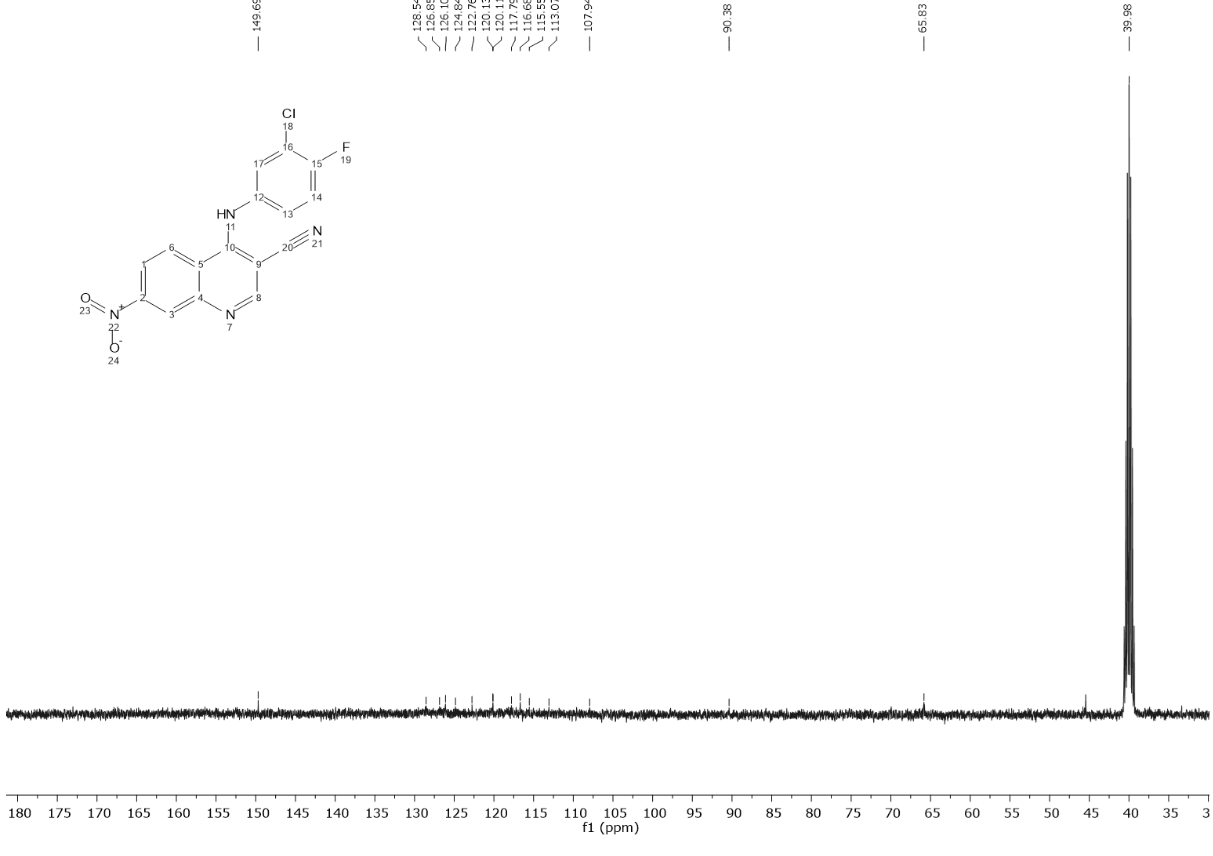

149.69
128.54
126.85
126.10
124.84
122.76
120.13
120.11
117.79
116.68
115.55
113.07
107.94
90.38
65.83
39.98
Cl
F
HN
N
O
N+
O-
f1 (ppm)

Sample Report

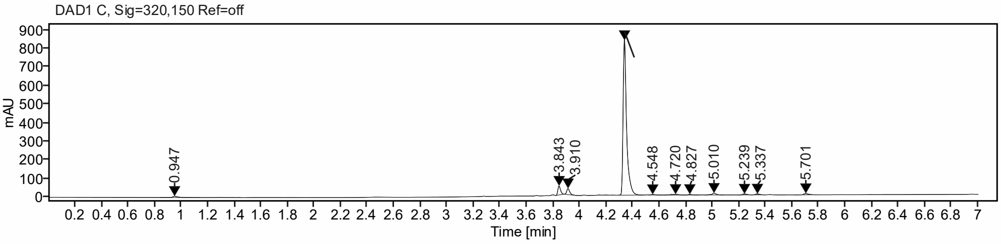

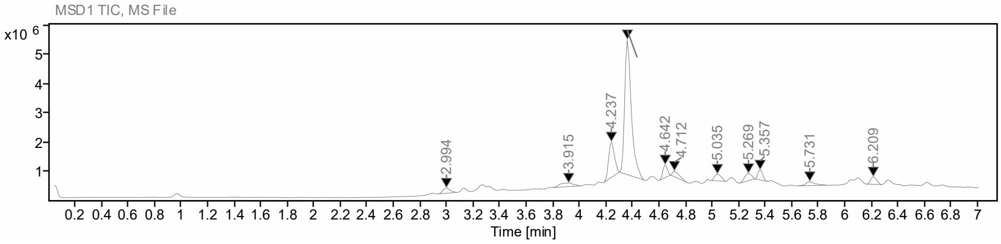

Sample Purity

Signal Description DAD1 C, Sig=320,150 Ref=off

Sample Name	Name	RT	Width	Area	Area%	Height
FGMM14_dry		0.947	0.037	20.5539	1.17	7.0253
FGMM14_dry		3.843	0.022	68.6302	3.90	48.1197
FGMM14_dry		3.910	0.023	51.2407	2.91	32.4987
FGMM14_dry		4.336	0.026	1527.3207	86.76	834.3433
FGMM14_dry		4.548	0.030	5.1649	0.29	2.5634
FGMM14_dry		4.720	0.066	16.2972	0.93	3.9991
FGMM14_dry		4.827	0.027	6.8776	0.39	3.7029
FGMM14_dry		5.010	0.030	22.5814	1.28	9.7833
FGMM14_dry		5.239	0.036	11.9920	0.68	5.2055
FGMM14_dry		5.337	0.032	11.1190	0.63	5.2176
FGMM14_dry		5.701	0.036	18.5281	1.05	7.2634

Max Area% 86.765

UV Signal Purity>95% Fail

UV Apex Spectra

RT:

4.357

Sample Name:

FGMM14_dry

Signal Name:

MSD1TIC

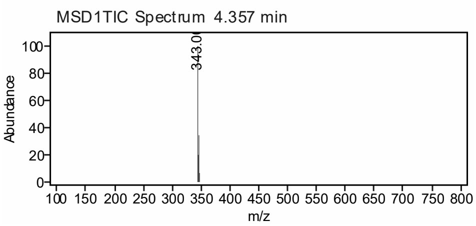

RT:

4.642

Sample Name:

FGMM14_dry

Signal Name:

MSD1TIC

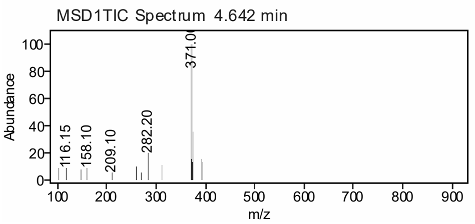

RT:

4.712

Sample Name:

FGMM14_dry

Signal Name:

MSD1TIC

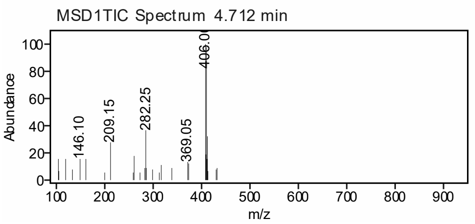

RT:

5.035

Sample Name:

FGMM14_dry

Signal Name:

MSD1TIC

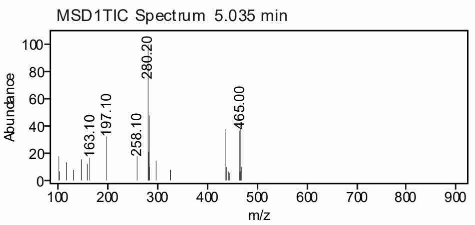

RT:

5.269

Sample Name:

FGMM14_dry

Signal Name:

MSD1TIC

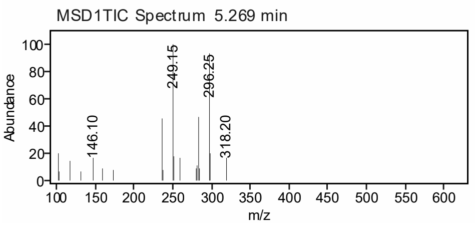

Compound S6

Sample Report

GOETHE
UNIVERSITÄT
FRANKFURT AM MAIN

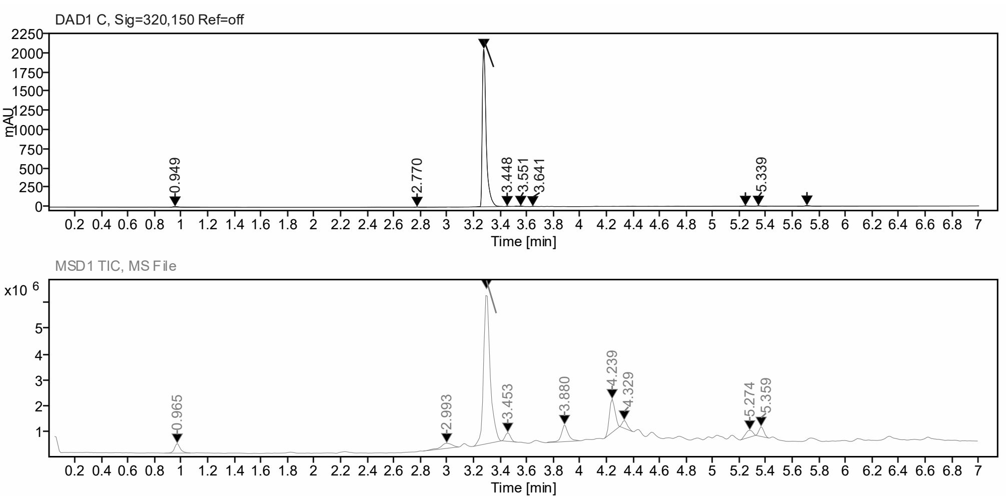

Sample Purity

Signal Description DAD1 C, Sig=320,150 Ref=off

Sample Name	Name	RT	Width	Area	Area%	Height
FGMM17_F8		0.949	0.045	30.3033	0.70	9.7498
FGMM17_F8		2.770	0.021	7.2269	0.17	4.4396
FGMM17_F8		3.273	0.029	4222.9604	97.37	2056.6299
FGMM17_F8		3.448	0.020	6.3850	0.15	5.0420
FGMM17_F8		3.551	0.024	17.6602	0.41	7.4269
FGMM17_F8		3.641	0.022	5.8895	0.14	3.7740
FGMM17_F8		5.241	0.037	13.2710	0.31	5.5619
FGMM17_F8		5.339	0.033	12.6399	0.29	5.8046
FGMM17_F8		5.705	0.037	20.6799	0.48	8.1041

Max Area% 97.370

UV Signal Purity>95% Pass

UV Apex Spectra

Sample Report

GOETHE UNIVERSITÄT FRANKFURT AM MAIN

RT:

0.965

Sample Name:

FGMM17_F8

Signal Name:

MSD1TIC

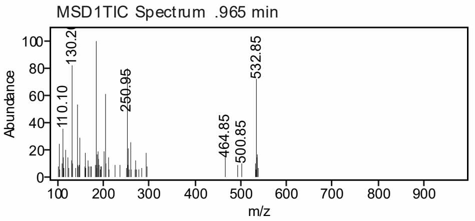

RT:

2.993

Sample Name:

FGMM17_F8

Signal Name:

MSD1TIC

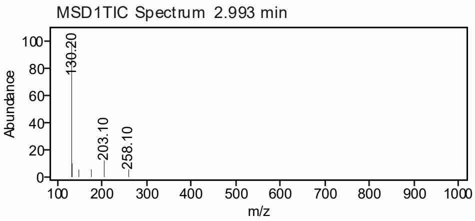

RT:

3.293

Sample Name:

FGMM17_F8

Signal Name:

MSD1TIC

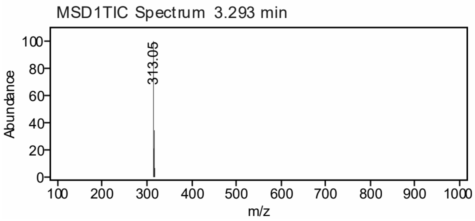

RT:

3.453

Sample Name:

FGMM17_F8

Signal Name:

MSD1TIC

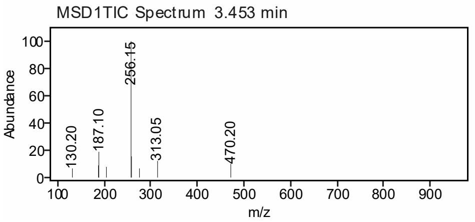

RT:

3.880

Sample Name:

FGMM17_F8

Signal Name:

MSD1TIC

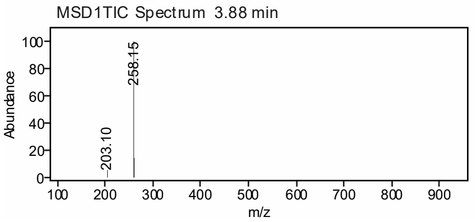

Compound S7

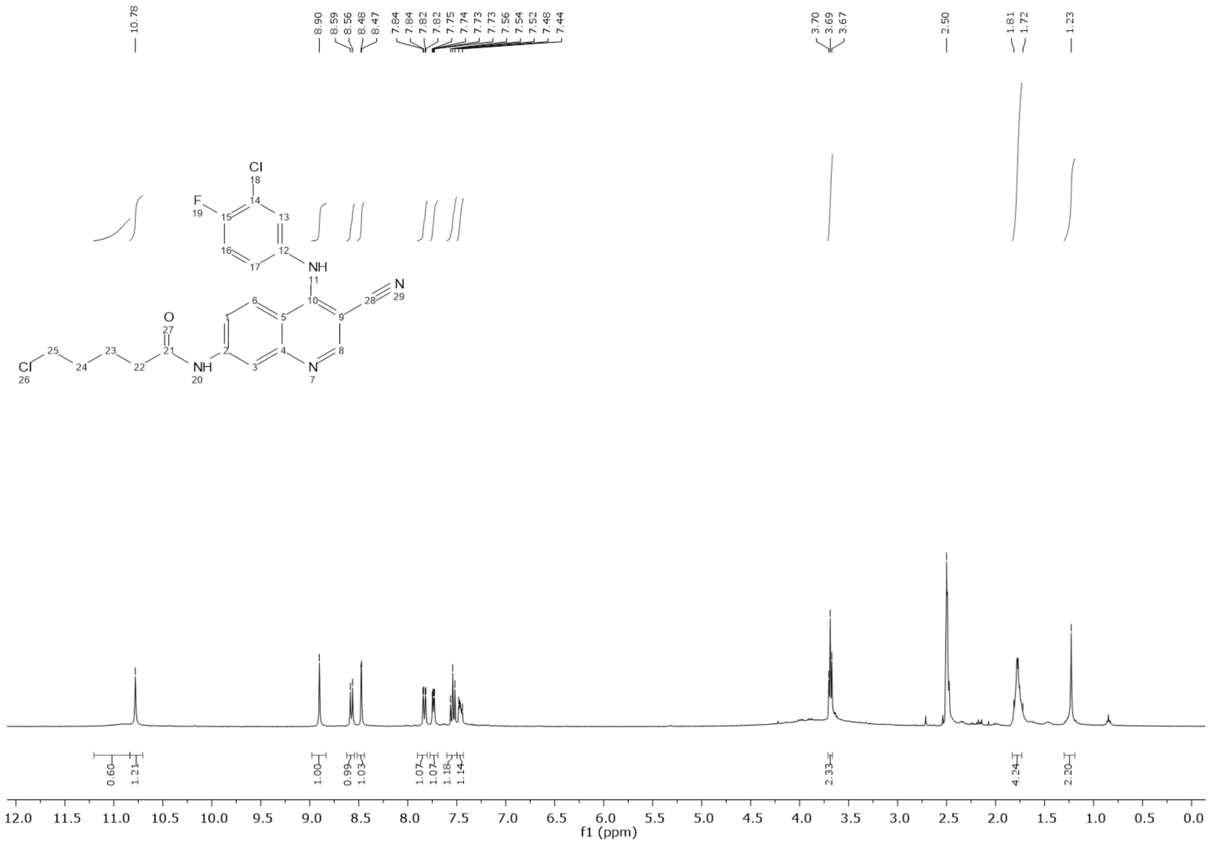
10.78
8.90 8.59 8.56 8.48 8.47 7.84 7.84 7.82 7.82 7.75 7.74 7.73 7.73 7.56 7.54 7.52 7.48 7.44
3.70 3.69 3.67
2.50
1.81 1.72
1.23
0.60 1.21 1.00 0.99 1.03 1.07 1.07 1.18 1.14 2.33 4.24 2.20
12.0 11.5 11.0 10.5 10.0 9.5 9.0 8.5 8.0 7.5 7.0 6.5 6.0 5.5 5.0 4.5 4.0 3.5 3.0 2.5 2.0 1.5 1.0 0.5 0.0
f1 (ppm)

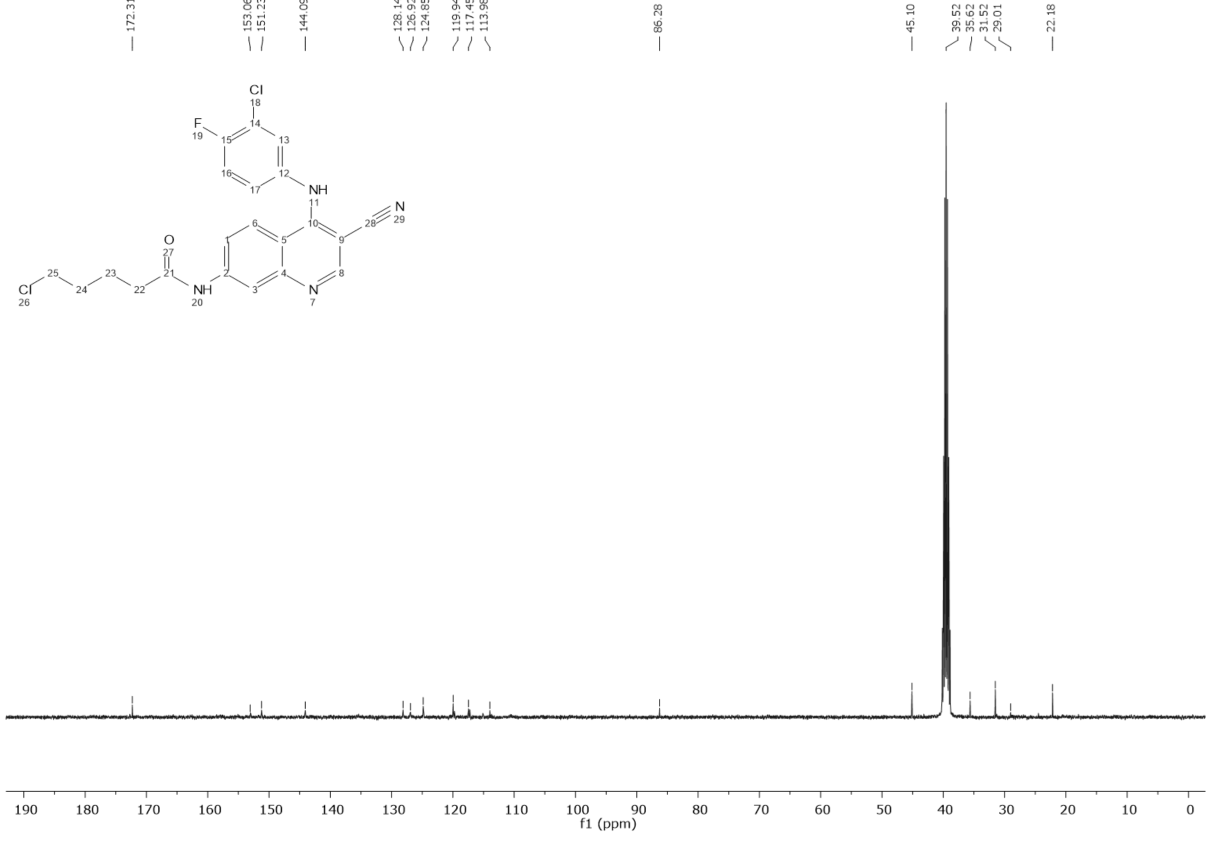
172.31
153.06 151.23
144.09
128.14 126.92 124.85 119.94 117.45 113.98
86.28
45.10
39.52 35.62 31.52 29.01
22.18
190 180 170 160 150 140 130 120 110 100 90 80 70 60 50 40 30 20 10 0
f1 (ppm)

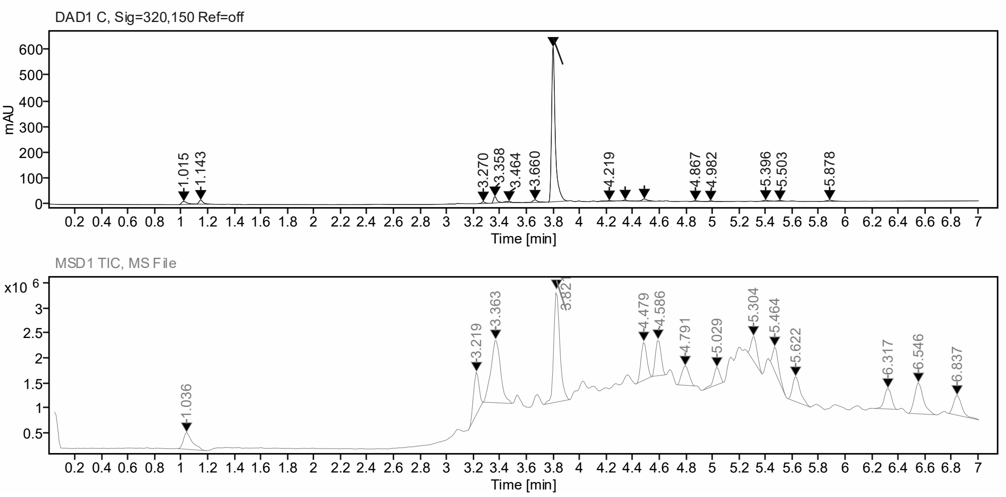

Sample Purity

Signal Description DAD1 C, Sig=320,150 Ref=off

Sample Name	Name	RT	Width	Area	Area%	Height
FGMM62_dry		1.015	0.045	34.6992	2.44	11.7382
FGMM62_dry		1.143	0.031	34.4553	2.42	15.7009
FGMM62_dry		3.270	0.025	12.3912	0.87	7.1301
FGMM62_dry		3.358	0.026	43.5793	3.07	24.9199
FGMM62_dry		3.464	0.051	13.9950	0.98	4.8402
FGMM62_dry		3.660	0.038	27.6134	1.94	9.5512
FGMM62_dry		3.796	0.027	1161.4742	81.71	602.9103
FGMM62_dry		4.219	0.034	9.3917	0.66	2.5294
FGMM62_dry		4.338	0.030	9.8524	0.69	4.1714
FGMM62_dry		4.482	0.039	26.3223	1.85	8.9518
FGMM62_dry		4.867	0.037	7.6196	0.54	2.8173
FGMM62_dry		4.982	0.033	5.7863	0.41	2.4470
FGMM62_dry		5.396	0.042	11.2207	0.79	4.0103
FGMM62_dry		5.503	0.039	6.8037	0.48	2.7679
FGMM62_dry		5.878	0.048	16.3246	1.15	4.6937

Max Area% 81.706

RT:

3.821

Sample Name:

FGMM62_dry

Signal Name:

MSD1TIC

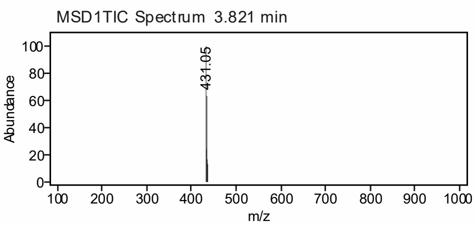

RT:

4.479

Sample Name:

FGMM62_dry

Signal Name:

MSD1TIC

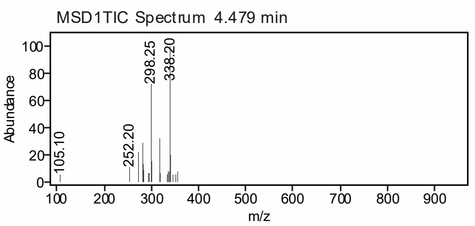

RT:

4.586

Sample Name:

FGMM62_dry

Signal Name:

MSD1TIC

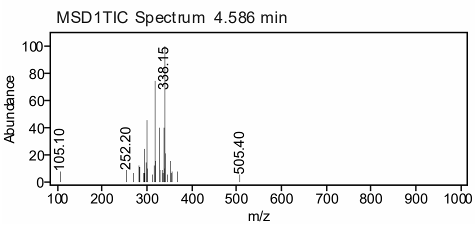

RT:

4.791

Sample Name:

FGMM62_dry

Signal Name:

MSD1TIC

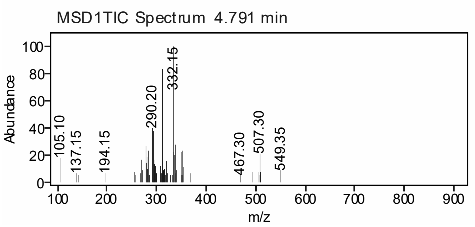

RT:

5.029

Sample Name:

FGMM62_dry

Signal Name:

MSD1TIC

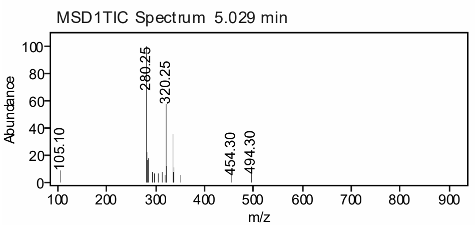

Compound 56

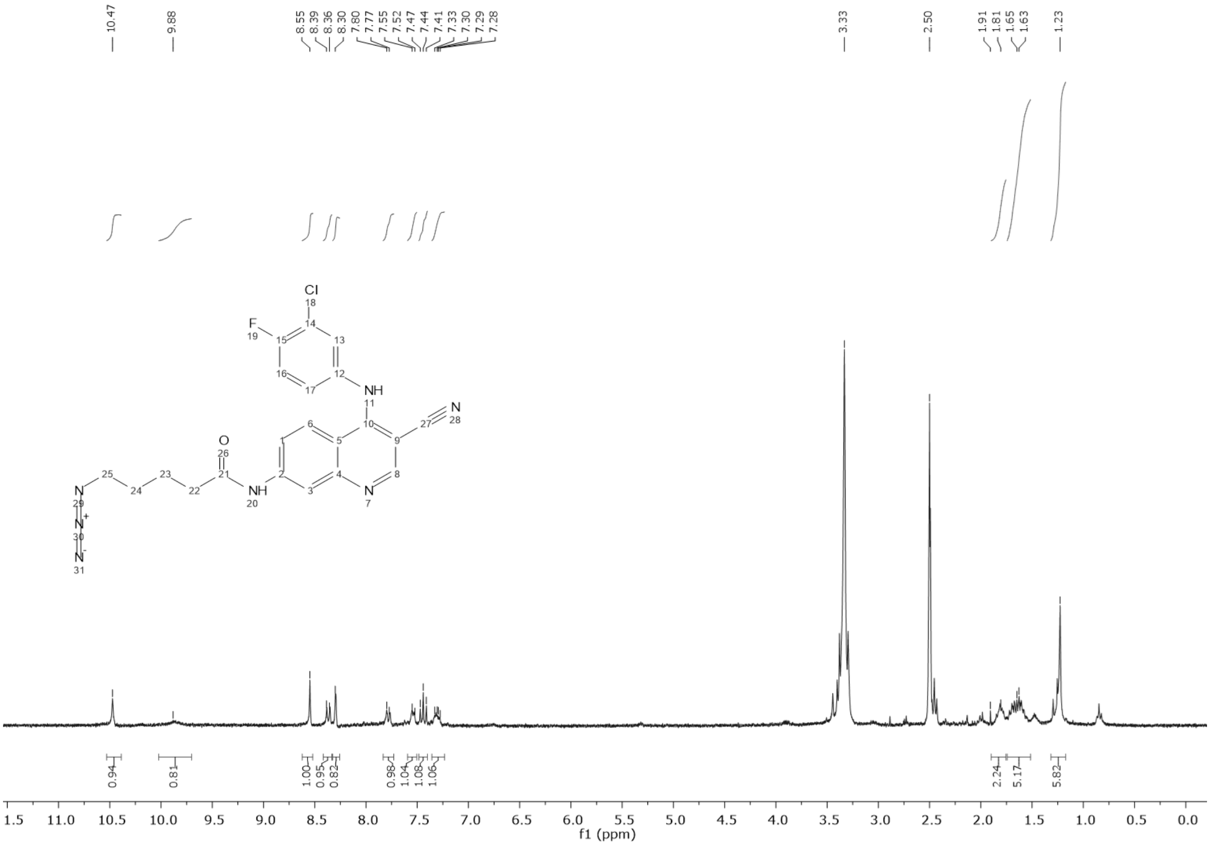

GOETHE UNIVERSITÄT FRANKFURT AM MAIN

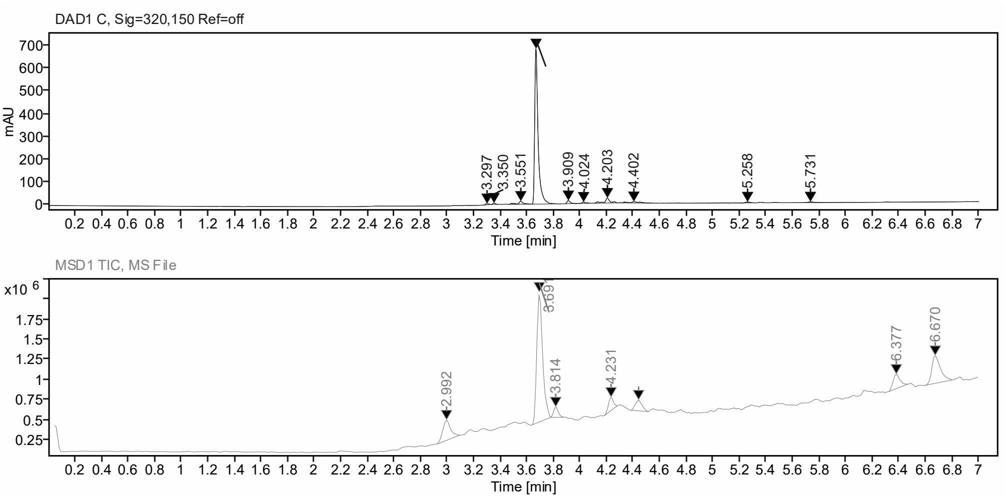

Sample Purity

Signal Description DAD1 C, Sig=320,150 Ref=off

Sample Name	Name	RT	Width	Area	Area%	Height
FGMM102rp_dry		3.297	0.036	8.7995	0.62	4.8679
FGMM102rp_dry		3.350	0.021	7.7446	0.55	5.8965
FGMM102rp_dry		3.551	0.025	36.8595	2.60	13.6184
FGMM102rp_dry		3.666	0.024	1212.8698	85.44	682.2627
FGMM102rp_dry		3.909	0.026	22.8763	1.61	13.4340
FGMM102rp_dry		4.024	0.026	13.1646	0.93	5.8210
FGMM102rp_dry		4.203	0.027	62.8819	4.43	21.7437
FGMM102rp_dry		4.402	0.041	34.8717	2.46	5.9140
FGMM102rp_dry		5.258	0.037	10.0403	0.71	4.2091
FGMM102rp_dry		5.731	0.041	9.3820	0.66	3.4910

Max Area% 85.444
UV Signal Purity>95% Fail

UV Apex Spectra

GOETHE UNIVERSITÄT FRANKFURT AM MAIN

MSD Apex Spectra

RT:

2.992

Sample Name:

FGMM102rp_dry

Signal Name:

MSD1TIC

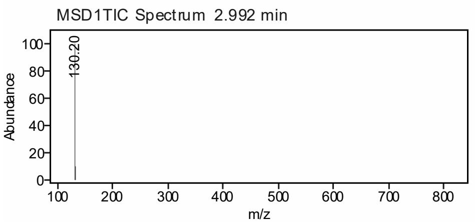

RT:

3.691

Sample Name:

FGMM102rp_dry

Signal Name:

MSD1TIC

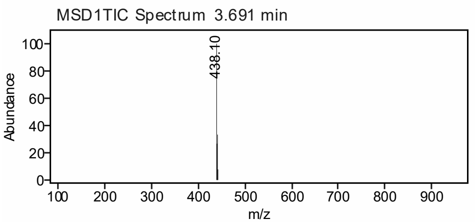

RT:

3.814

Sample Name:

FGMM102rp_dry

Signal Name:

MSD1TIC

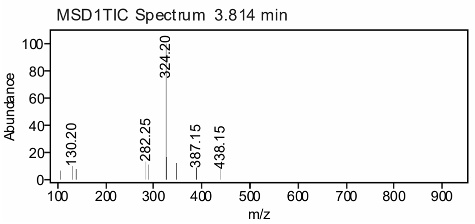

RT:

4.231

Sample Name:

FGMM102rp_dry

Signal Name:

MSD1TIC

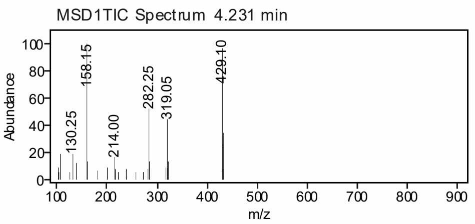

Compound S8

Sample Report

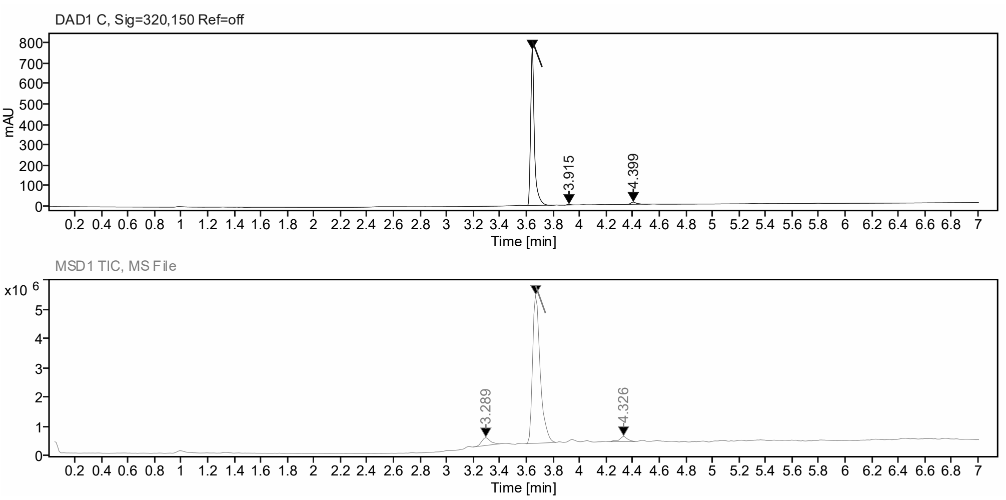

Sample Purity

Signal Description DAD1 C, Sig=320,150 Ref=off

Sample Name	Name	RT	Width	Area	Area%	Height
FGMM156_F10		3.639	0.027	1493.7979	97.32	764.6455
FGMM156_F10		3.915	0.029	6.5655	0.43	3.5854
FGMM156_F10		4.399	0.044	34.6067	2.25	11.2738

Max Area% 97.318

UV Signal Purity>95% Pass

UV Apex Spectra

RT:

3.639

Sample Name:

FGMM156_F10

Signal Name:

DAD1C

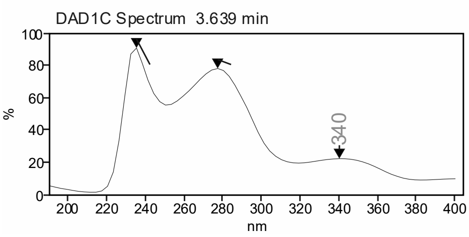

RT:

3.915

Sample Name:

FGMM156_F10

Signal Name:

DAD1C

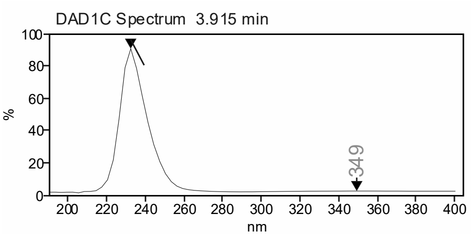

RT:

4.399

Sample Name:

FGMM156_F10

Signal Name:

DAD1C

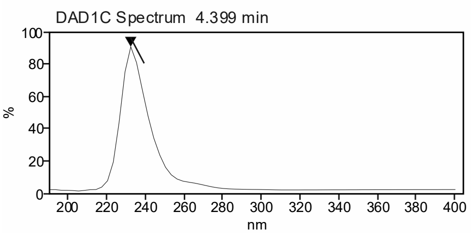

MSD Apex Spectra

RT:

3.289

Sample Name:

FGMM156_F10

Signal Name:

MSD1TIC

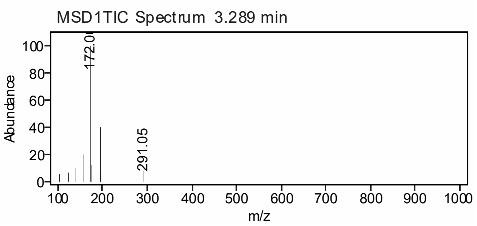

RT:

3.665

Sample Name:

FGMM156_F10

Signal Name:

MSD1TIC

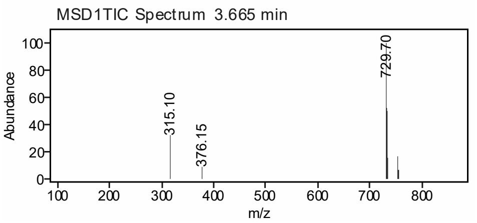

Compound S9

Sample Report

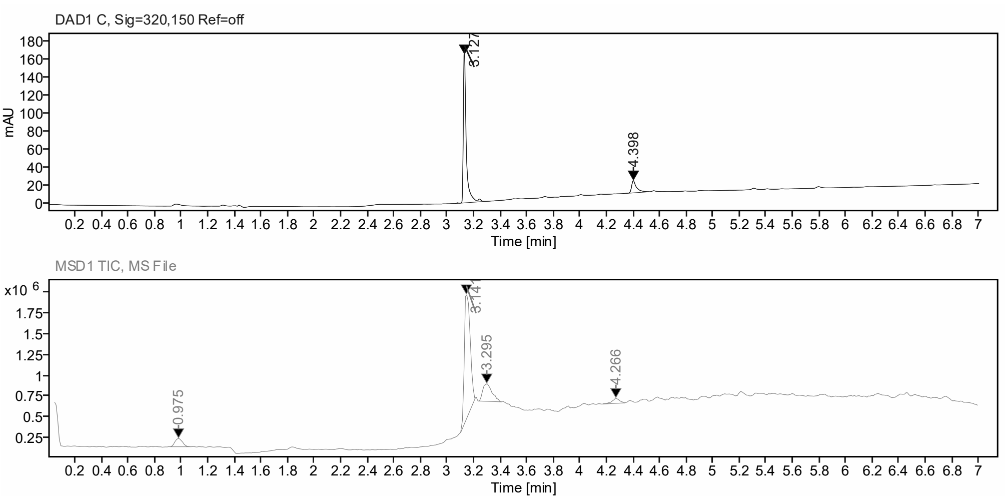

Sample Purity

Signal Description DAD1 C, Sig=320,150 Ref=off

Sample Name	Name	RT	Width	Area	Area%	Height
FGMM173_30min		3.127	0.020	257.9437	87.82	163.8641
FGMM173_30min		4.398	0.035	35.7795	12.18	13.5148

Max Area% 87.819

UV Signal Purity>95% Fail

UV Apex Spectra

RT:

3.127

Sample Name:

FGMM173_30min

Signal Name:

DAD1C

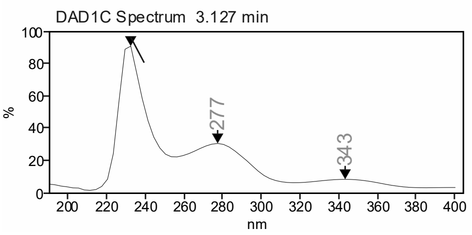

GOETHE UNIVERSITÄT FRANKFURT AM MAIN

RT:

4.398

Sample Name:

FGMM173_30min

Signal Name:

DAD1C

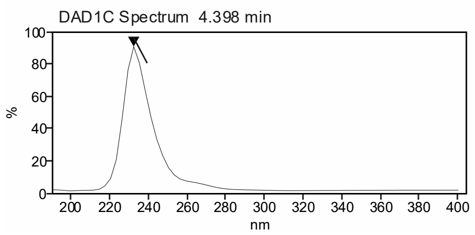

MSD Apex Spectra

RT:

0.975

Sample Name:

FGMM173_30min

Signal Name:

MSD1TIC

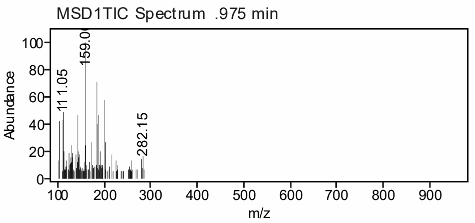

RT:

3.141

Sample Name:

FGMM173_30min

Signal Name:

MSD1TIC

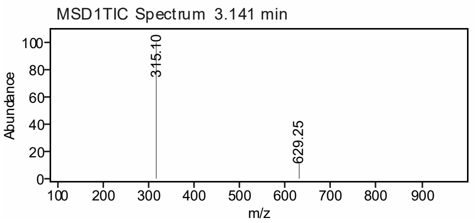

RT:

3.295

Sample Name:

FGMM173_30min

Signal Name:

MSD1TIC

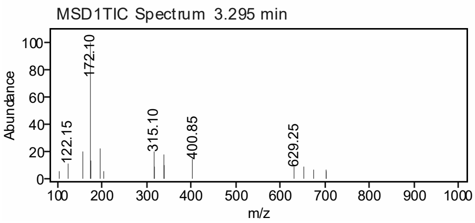

Compound 68

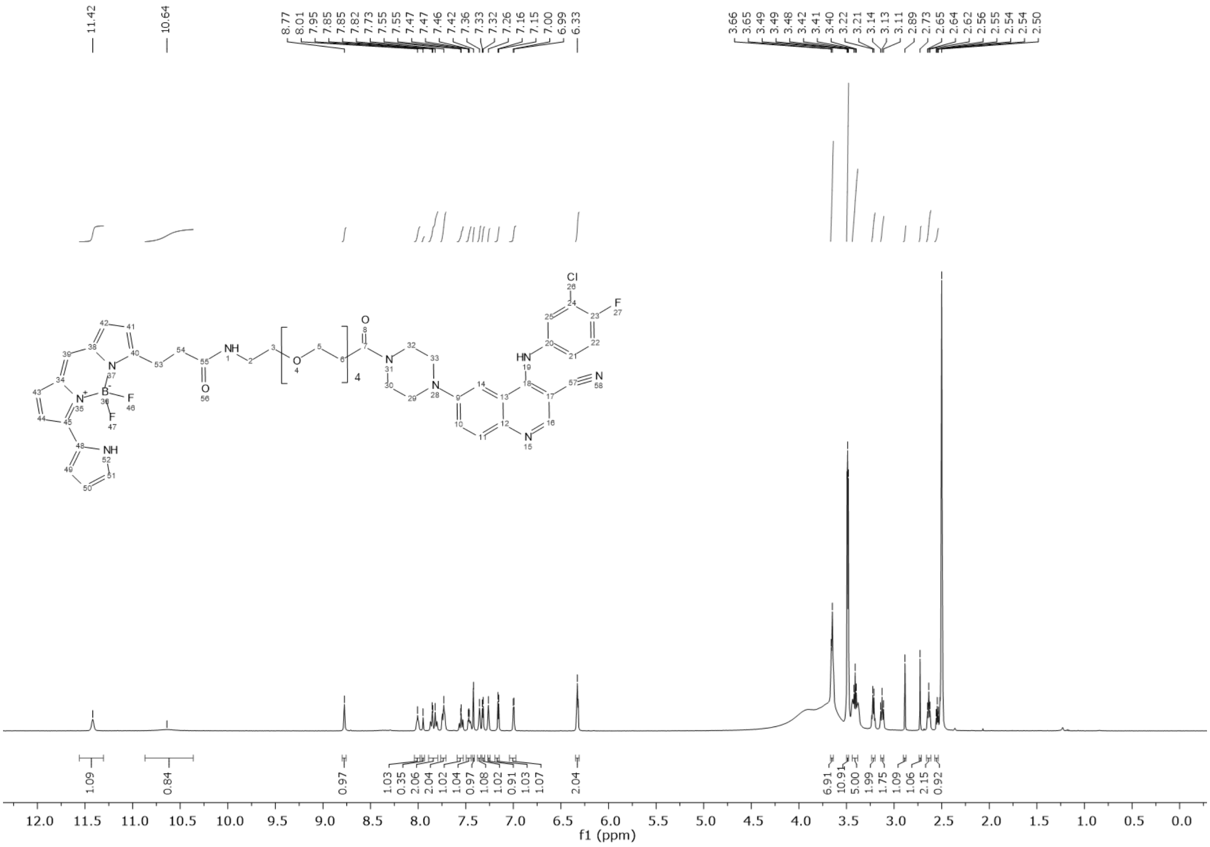

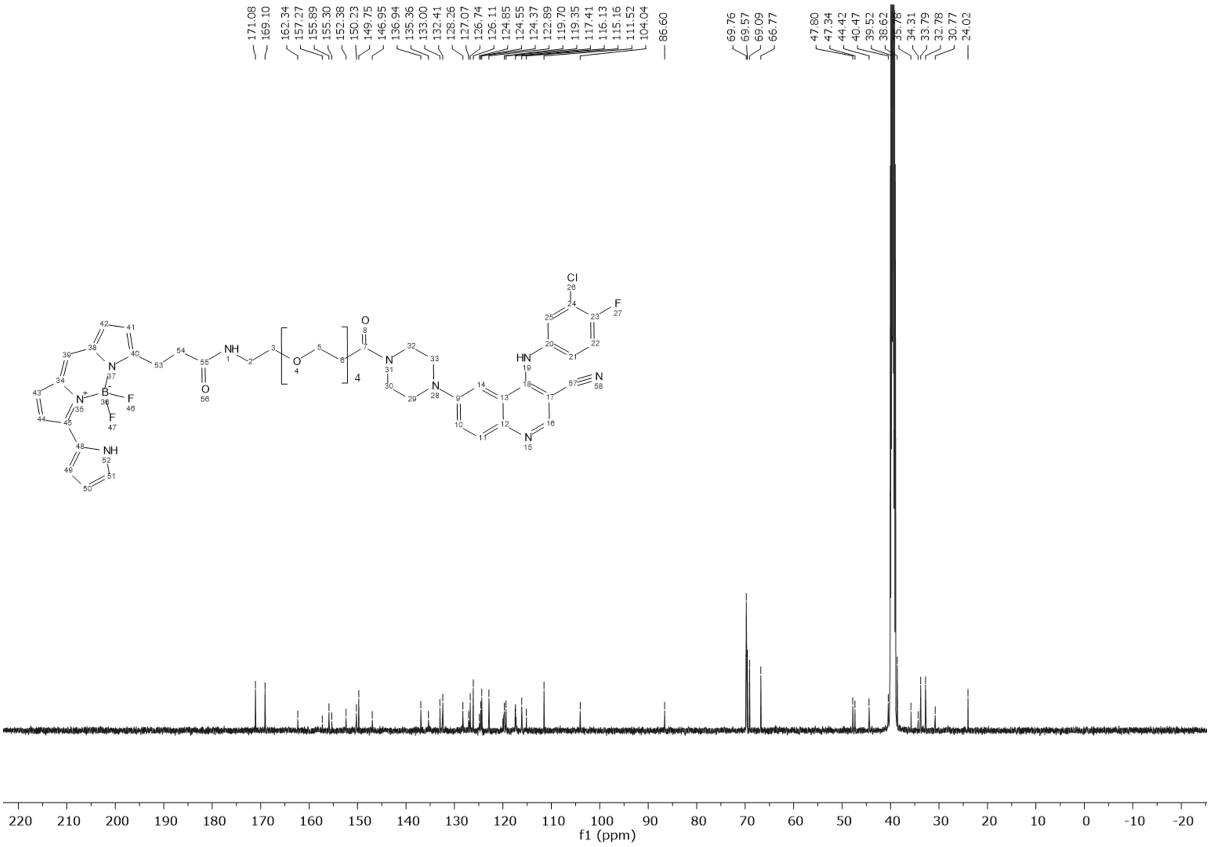

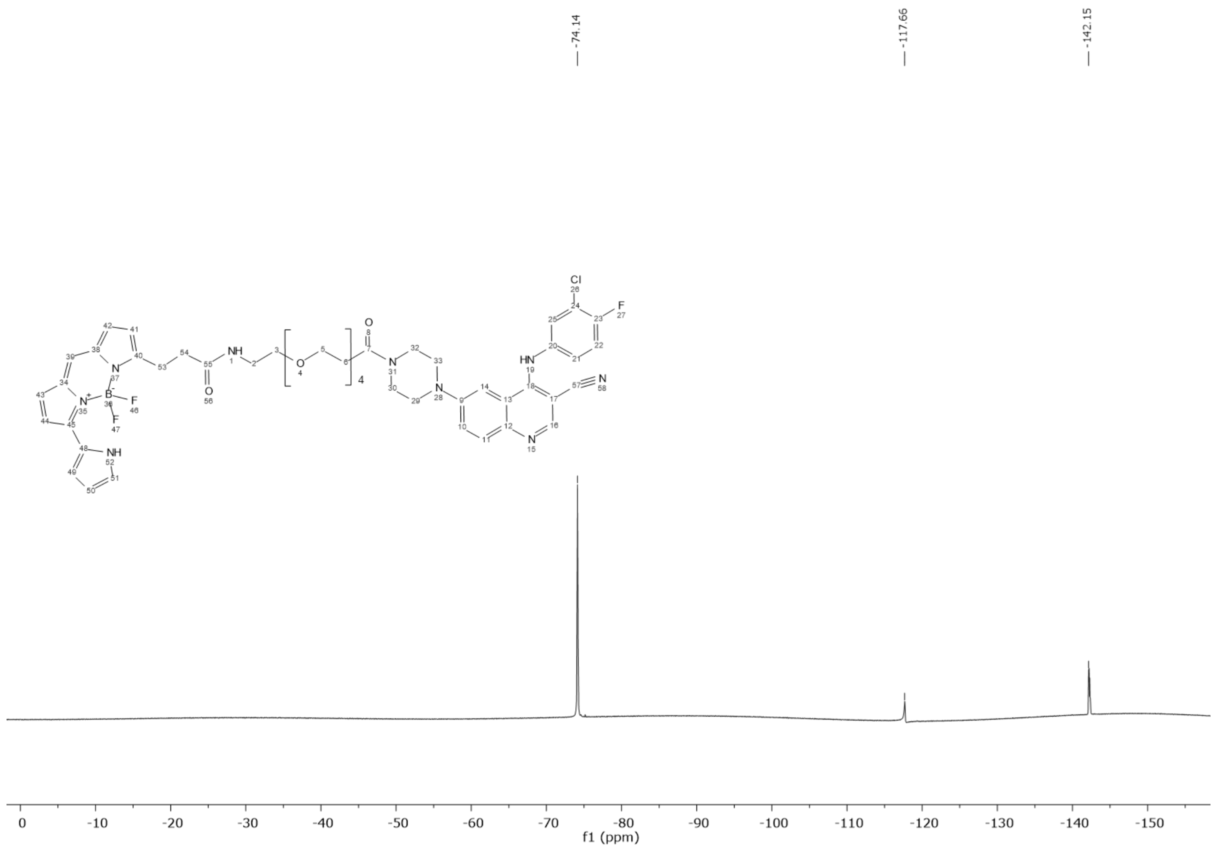
-74.14
-117.66
-142.15
0
-10
-20
-30
-40
-50
-60
-70
-80
-90
-100
-110
-120
-130
-140
-150
f1 (ppm)

GOETHE UNIVERSITÄT FRANKFURT AM MAIN

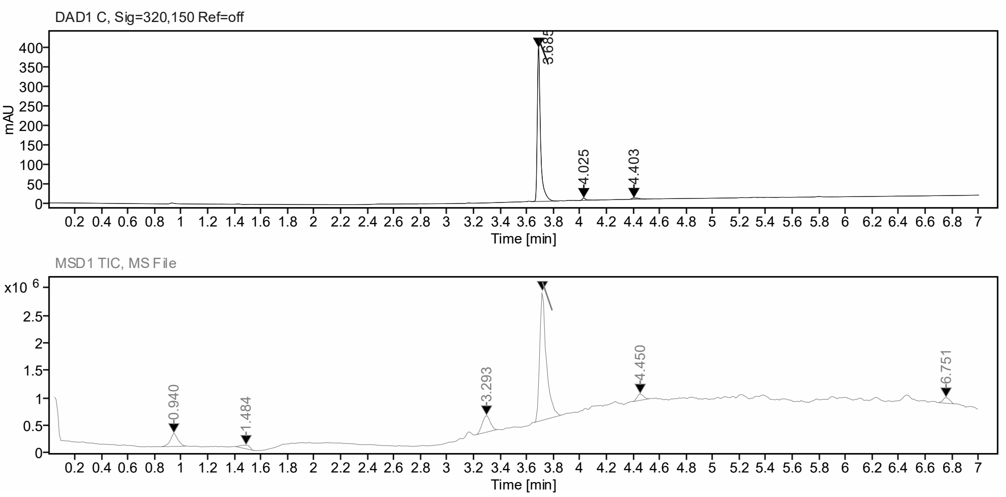

Sample Purity

Signal Description DAD1 C, Sig=320,150 Ref=off

Sample Name	Name	RT	Width	Area	Area%	Height
FGMM175_F1		3.685	0.023	670.6725	96.85	395.3347
FGMM175_F1		4.025	0.025	9.8688	1.43	5.8786
FGMM175_F1		4.403	0.061	11.9576	1.73	3.4573

Max Area% 96.848

UV Signal Purity>95% Pass

UV Apex Spectra

RT:

3.685

Sample Name:

FGMM175_F1

Signal Name:

DAD1C

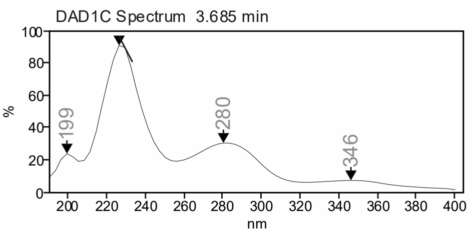

GOETHE UNIVERSITÄT FRANKFURT AM MAIN

RT:

3.293

Sample Name:

FGMM175_F1

Signal Name:

MSD1TIC

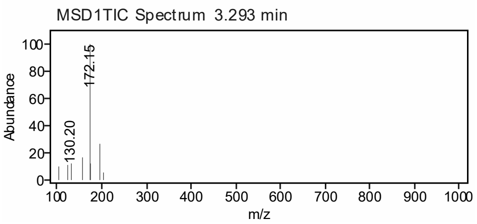

RT:

3.714

Sample Name:

FGMM175_F1

Signal Name:

MSD1TIC

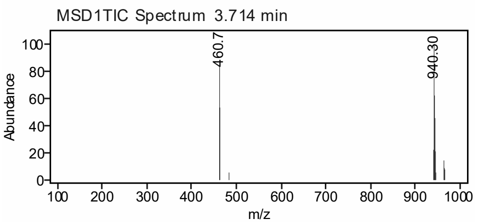

RT:

4.450

Sample Name:

FGMM175_F1

Signal Name:

MSD1TIC

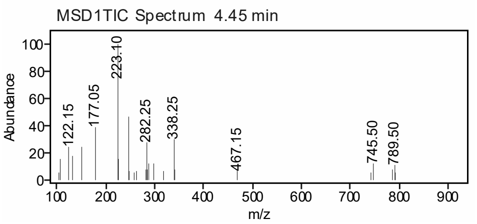

RT:

6.751

Sample Name:

FGMM175_F1

Signal Name:

MSD1TIC

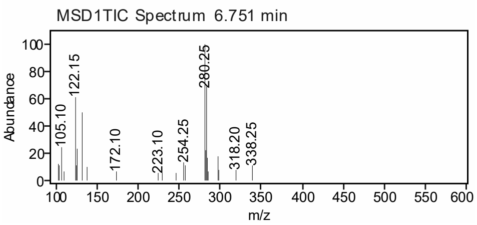

C:\User\...\2023\15.02.2023\FGMM175_J2 2/15/2023 11:27:05 AM FGMM175 mit HCCA gemessen.

FGMM175_J2 #1-7 RT: 0.00-0.25 AV: 7 NL: 3.19E7
T: FTMS + p MALDI Full ms [700.00-1450.00]

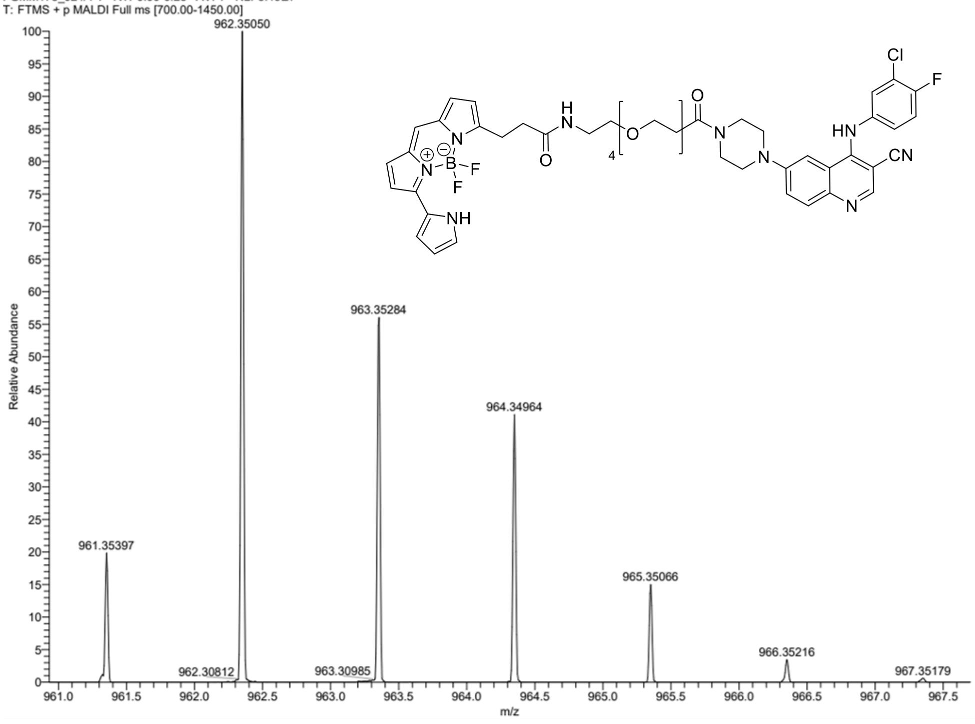

Compound S10

Sample Report

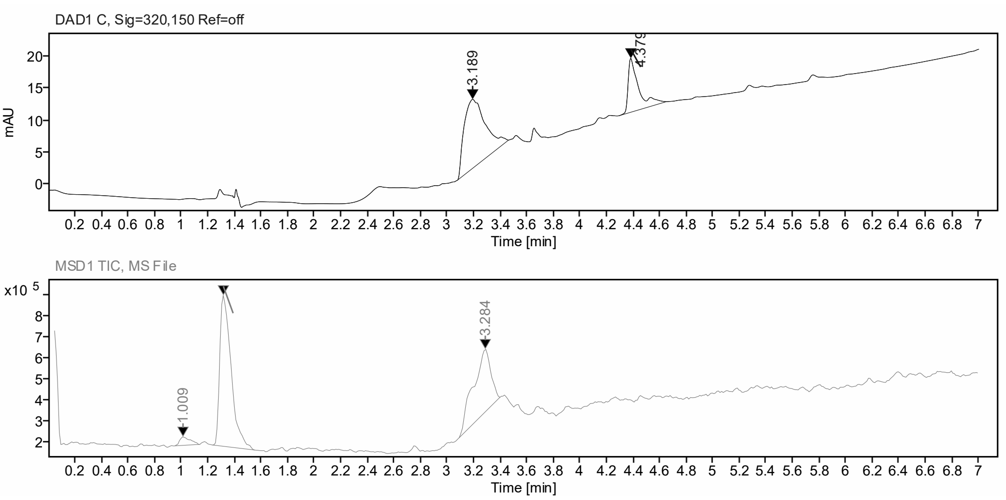

Sample Purity

Signal Description DAD1 C, Sig=320,150 Ref=off

Sample Name	Name	RT	Width	Area	Area%	Height
FGMM155re_2h		3.189	0.219	119.1046	72.29	10.7555
FGMM155re_2h		4.379	0.076	45.6654	27.71	8.4067

Max Area% 72.285

UV Signal Purity>95% Fail

UV Apex Spectra

RT:

3.189

Sample Name:

FGMM155re_2h

Signal Name:

DAD1C

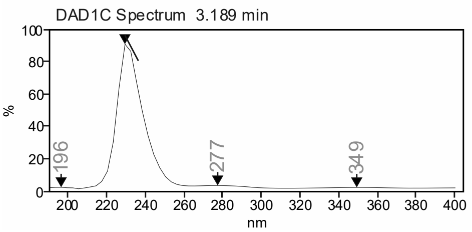

Sample Report

RT:

4.379

Sample Name:

FGMM155re_2h

Signal Name:

DAD1C

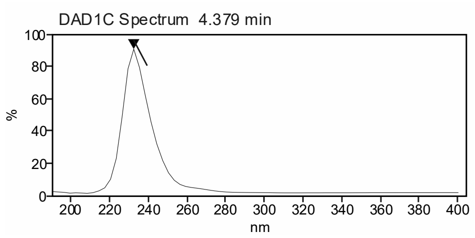

MSD Apex Spectra

RT:

1.009

Sample Name:

FGMM155re_2h

Signal Name:

MSD1TIC

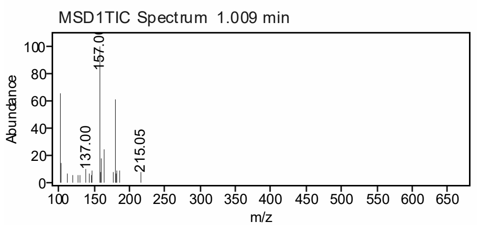

RT:

1.313

Sample Name:

FGMM155re_2h

Signal Name:

MSD1TIC

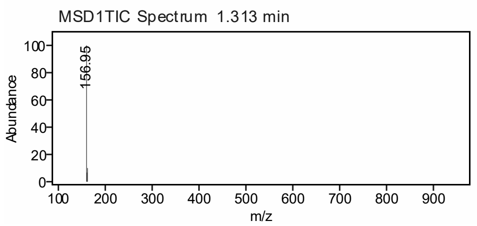

RT:

3.284

Sample Name:

FGMM155re_2h

Signal Name:

MSD1TIC

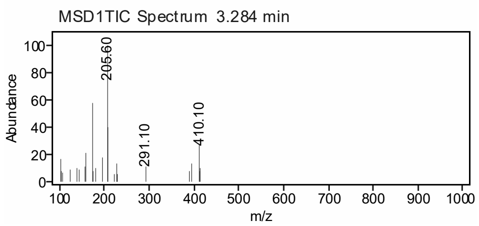

Compound S11

Sample Report

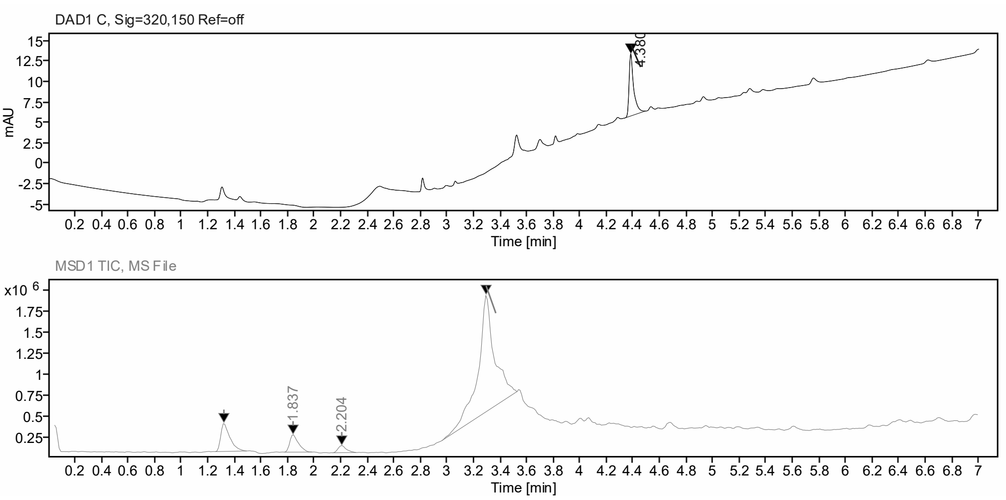

Sample Purity

Signal Description DAD1 C, Sig=320,150 Ref=off

Sample Name	Name	RT	Width	Area	Area%	Height
FGMM157_2h		4.380	0.033	18.1563	100.00	7.5996

Max Area% 100.000

UV Signal Purity>95% Pass

UV Apex Spectra

RT:

4.380

Sample Name:

FGMM157_2h

Signal Name:

DAD1C

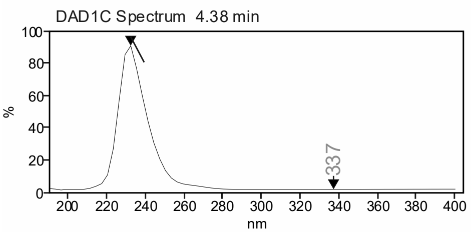

GOETHE UNIVERSITÄT FRANKFURT AM MAIN

MSD Apex Spectra

RT:

1.317

Sample Name:

FGMM157_2h

Signal Name:

MSD1TIC

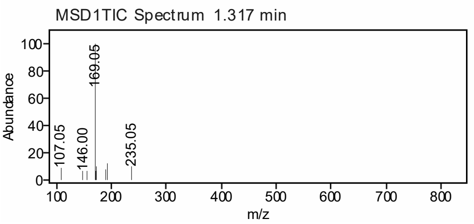

RT:

1.837

Sample Name:

FGMM157_2h

Signal Name:

MSD1TIC

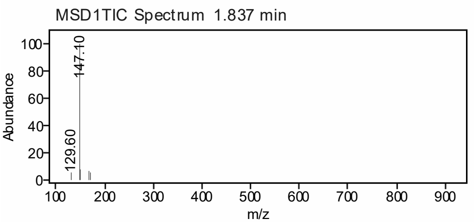

RT:

2.204

Sample Name:

FGMM157_2h

Signal Name:

MSD1TIC

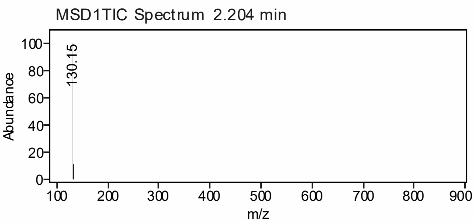

RT:

3.290

Sample Name:

FGMM157_2h

Signal Name:

MSD1TIC

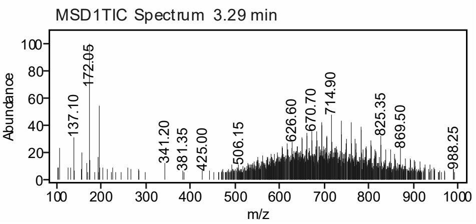

Compound S13

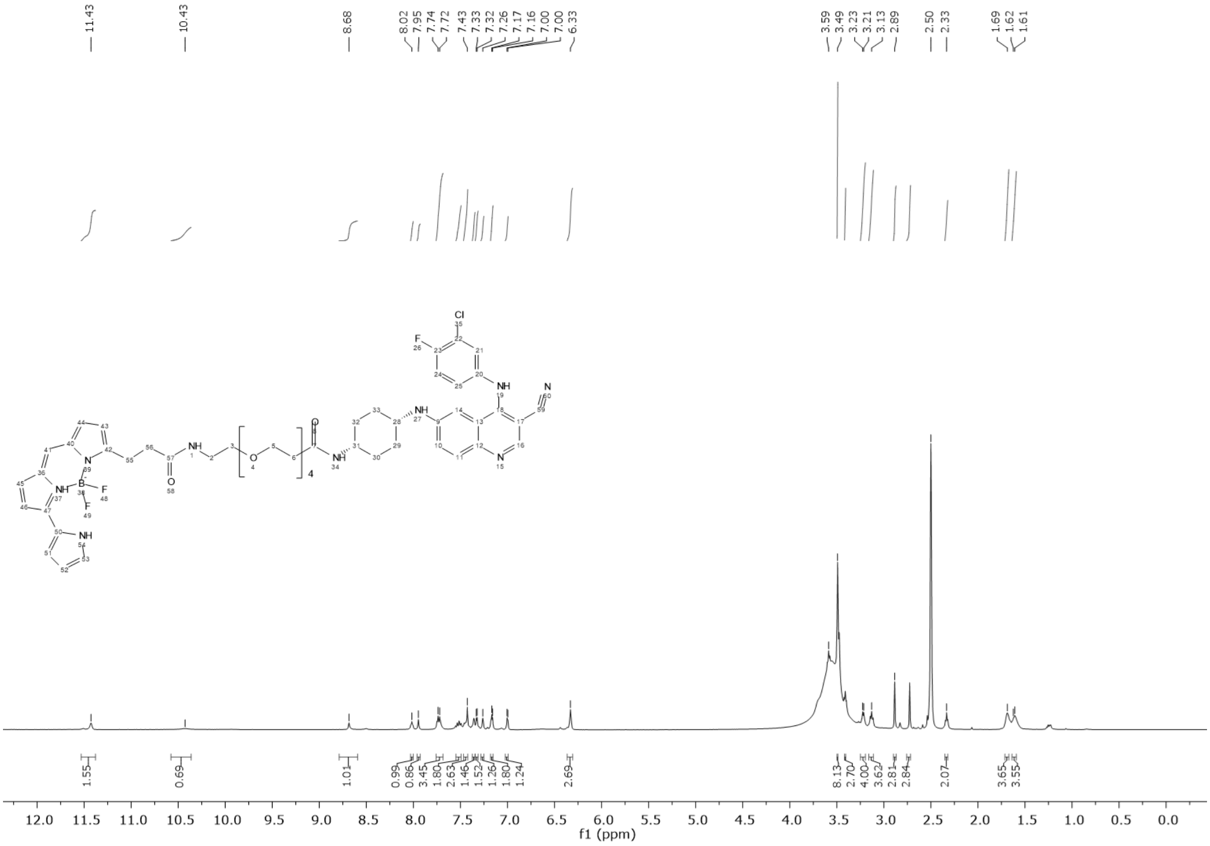

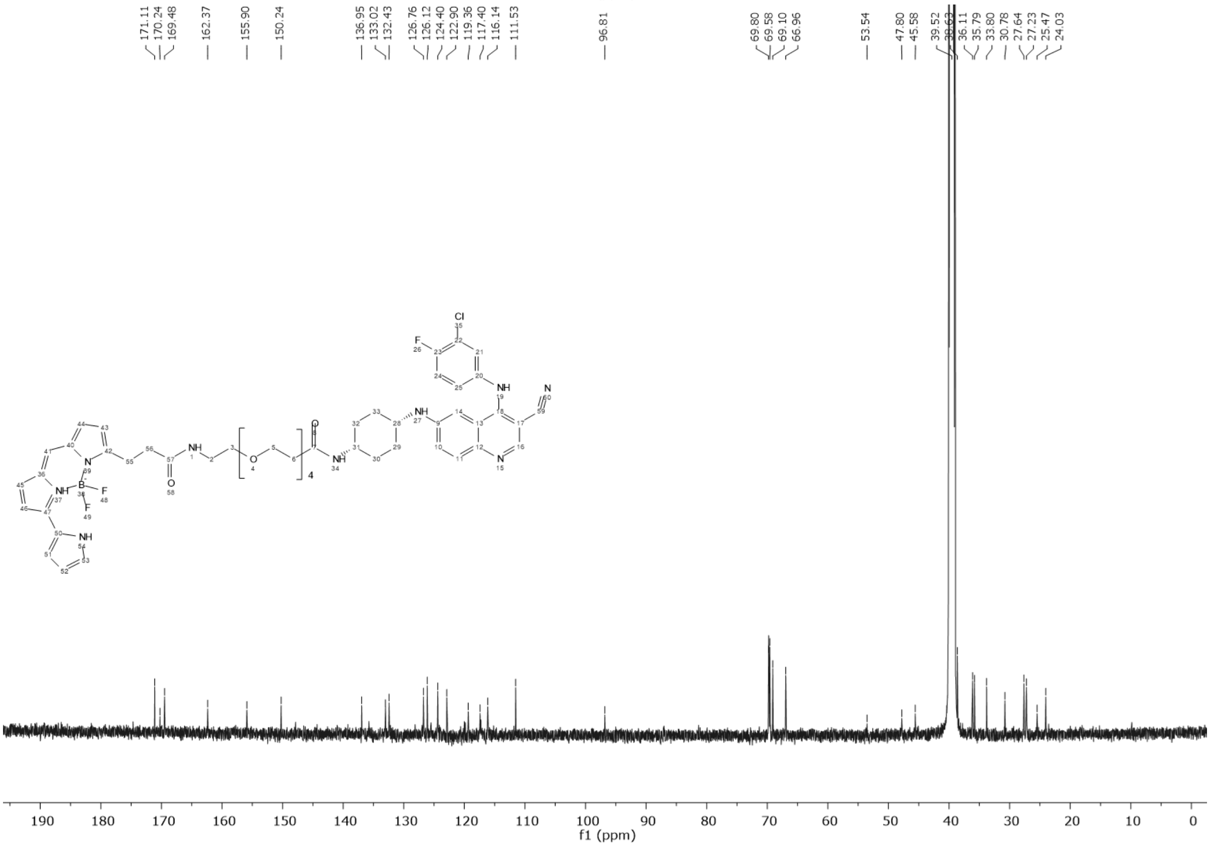

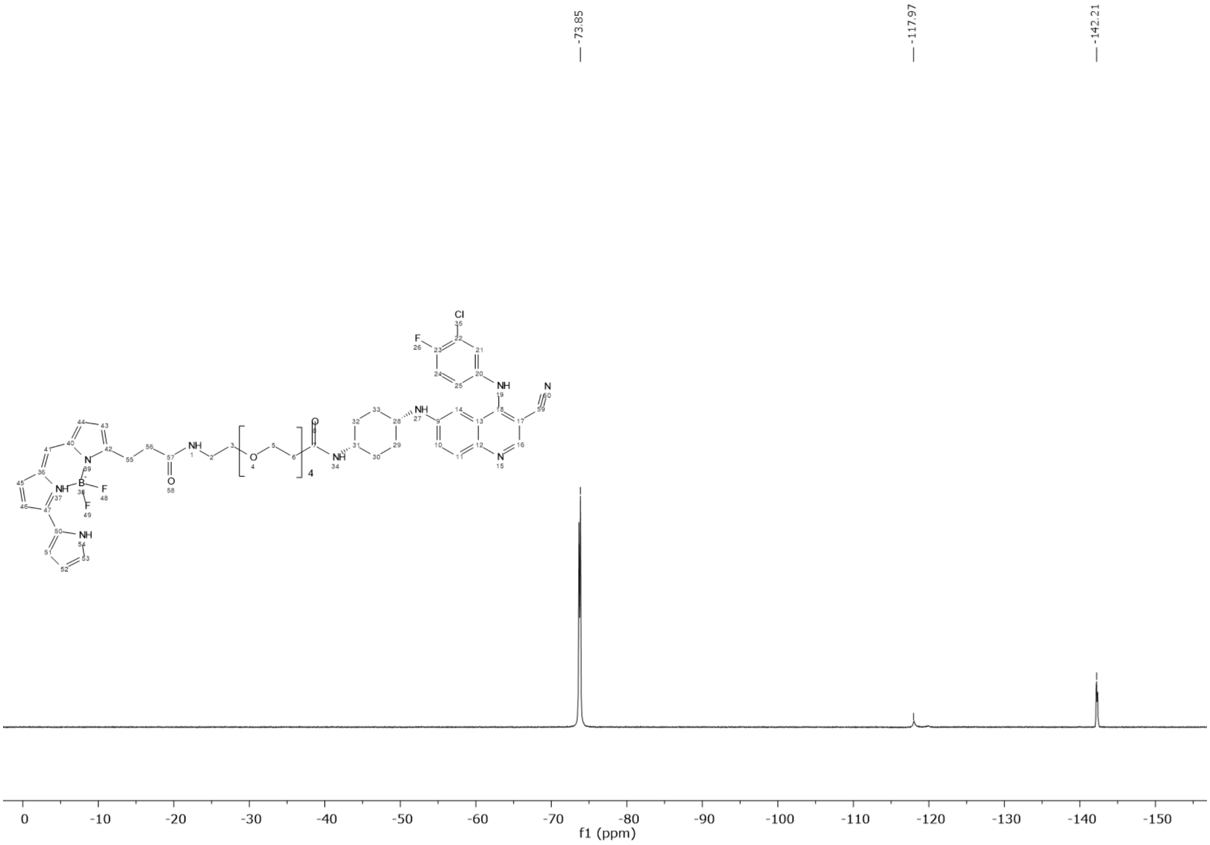
-73.85
-117.97
-142.21
0 -10 -20 -30 -40 -50 -60 -70 -80 -90 -100 -110 -120 -130 -140 -150
f1 (ppm)

Sample Report

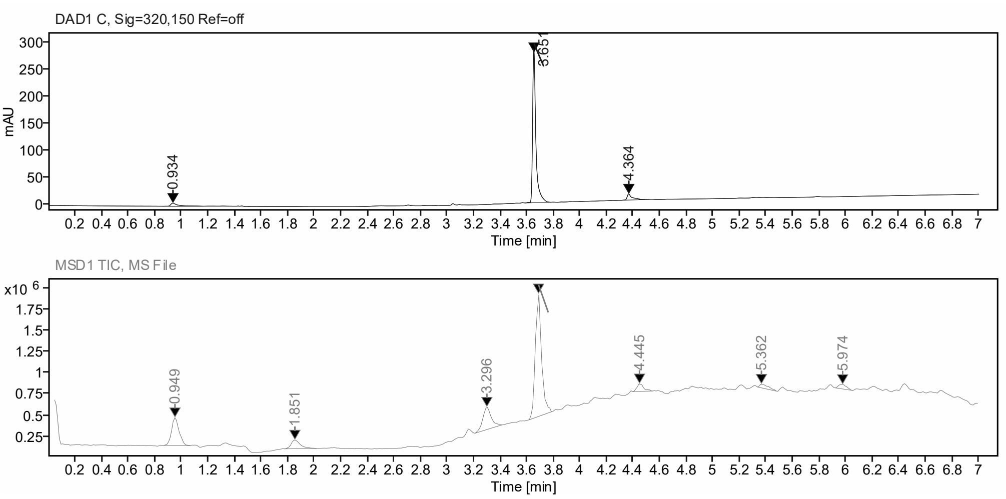

Sample Purity

Signal Description DAD1 C, Sig=320,150 Ref=off

Sample Name	Name	RT	Width	Area	Area%	Height
FGMM176_F6		0.934	0.044	22.9283	4.27	5.6722
FGMM176_F6		3.651	0.023	482.6148	89.94	278.7817
FGMM176_F6		4.364	0.030	31.0273	5.78	11.2430

Max Area% 89.944

UV Signal Purity>95% Fail

UV Apex Spectra

RT:

0.934

Sample Name:

FGMM176_F6

Signal Name:

DAD1C

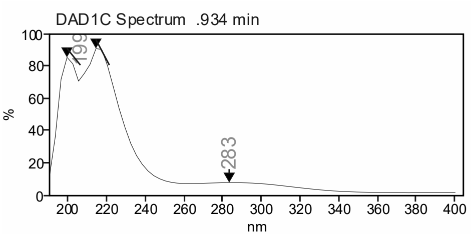

GOETHE UNIVERSITÄT FRANKFURT AM MAIN

RT:

3.296

Sample Name:

FGMM176_F6

Signal Name:

MSD1TIC

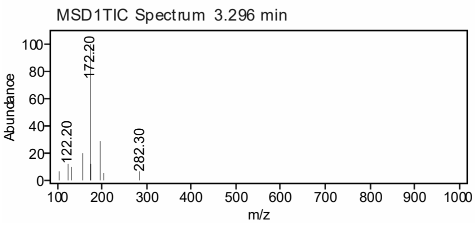

RT:

3.685

Sample Name:

FGMM176_F6

Signal Name:

MSD1TIC

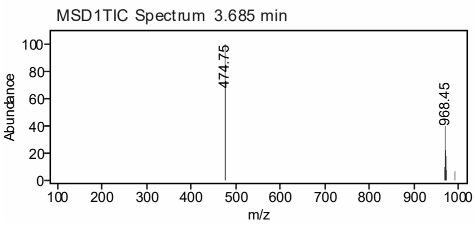

RT:

4.445

Sample Name:

FGMM176_F6

Signal Name:

MSD1TIC

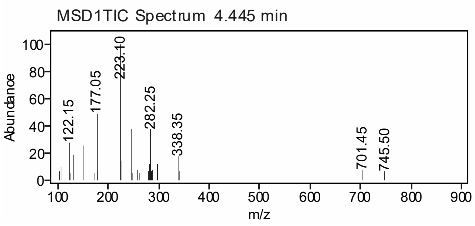

RT:

5.362

Sample Name:

FGMM176_F6

Signal Name:

MSD1TIC

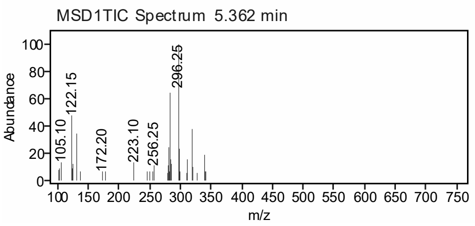

RT:

5.974

Sample Name:

FGMM176_F6

Signal Name:

MSD1TIC

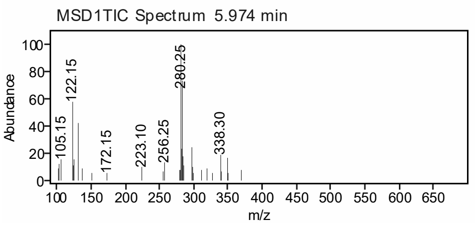

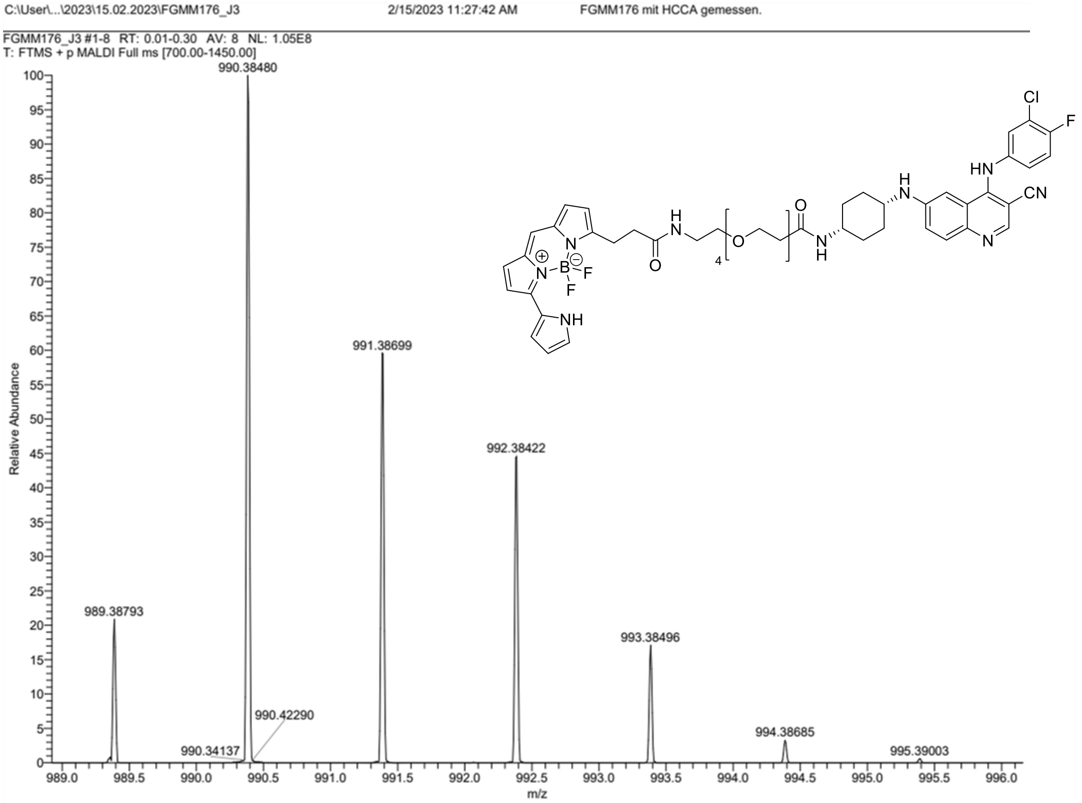
C:\User\...\2023\15.02.2023\FGMM176_J3
2/15/2023 11:27:42 AM
FGMM176 mit HCCA gemessen.
FGMM176_J3 #1-8 RT: 0.01-0.30 AV: 8 NL: 1.05E8
T: FTMS + p MALDI Full ms [700.00-1450.00]
990.38480
991.38699
992.38422
989.38793
993.38496
990.42290
990.34137
994.38685
995.39003
Relative Abundance
m/z
Cl
F
HN
H
N
CN
N
O
H
N
O
4
O
N
H
N
B
F
F
NH

Compound S14

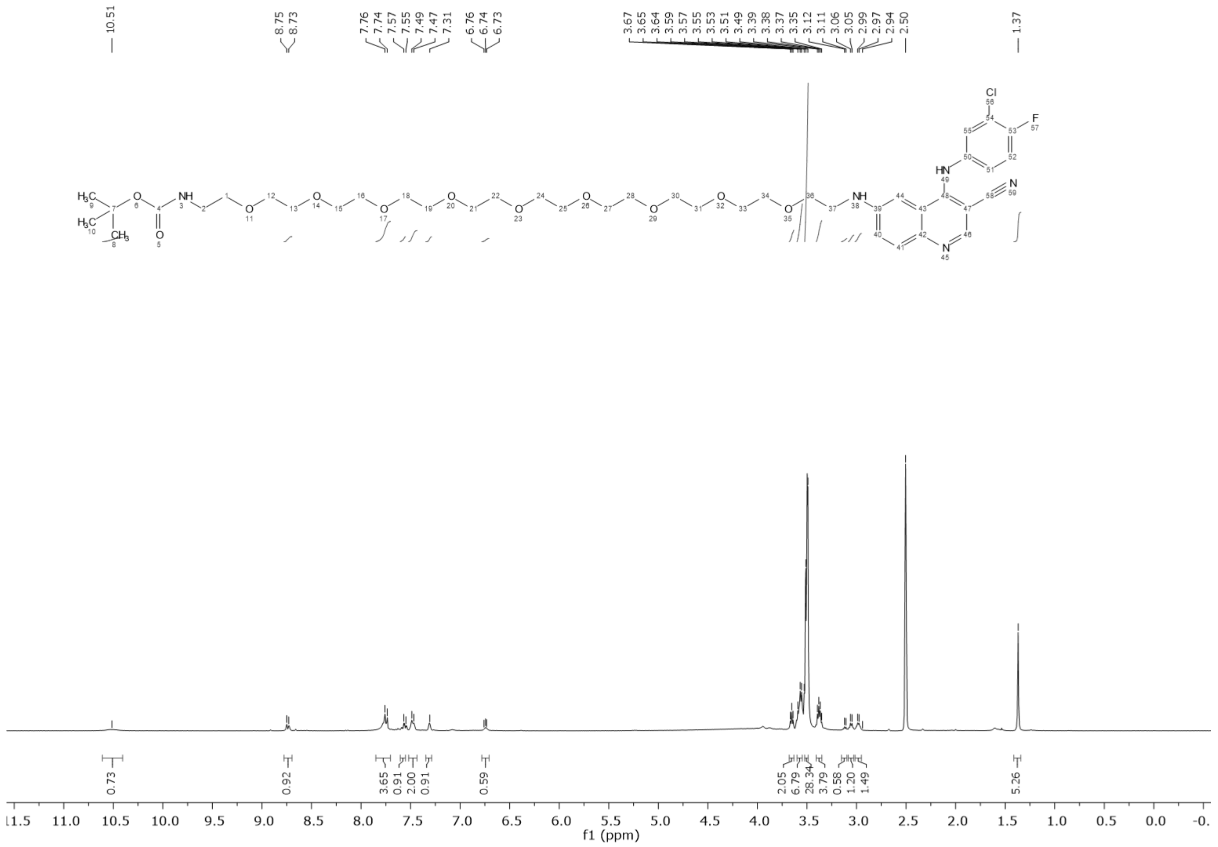

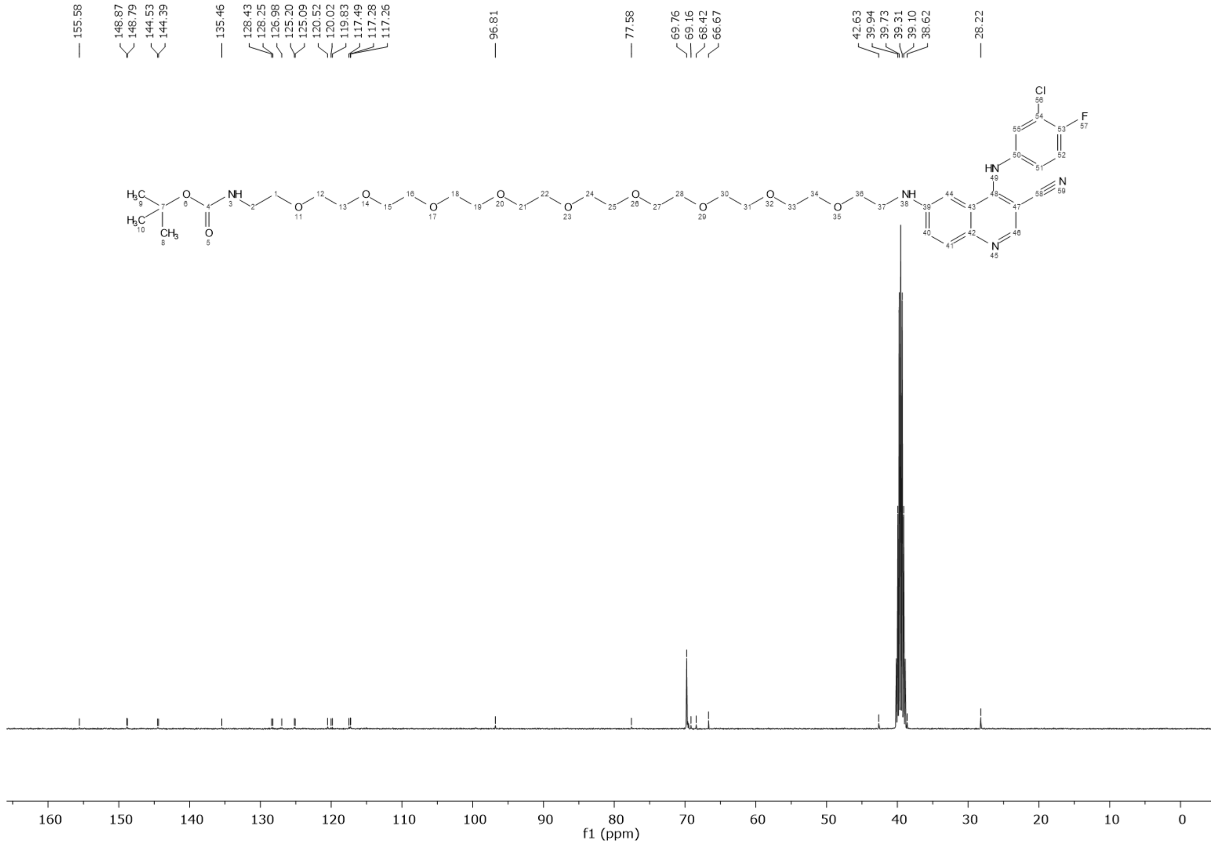

Sample Report

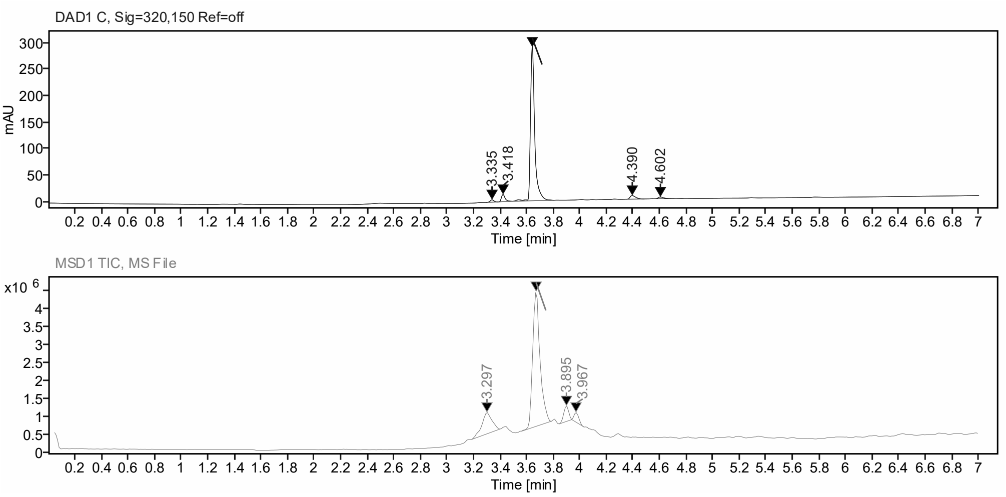

Sample Purity

Signal Description DAD1 C, Sig=320,150 Ref=off

Sample Name	Name	RT	Width	Area	Area%	Height
FGMM158_F17-18		3.335	0.029	8.2849	1.16	4.4345
FGMM158_F17-18		3.418	0.029	26.3113	3.70	13.5398
FGMM158_F17-18		3.638	0.031	648.2817	91.12	289.4995
FGMM158_F17-18		4.390	0.045	19.8453	2.79	6.8612
FGMM158_F17-18		4.602	0.044	8.7591	1.23	3.1306

Max Area% 91.117

UV Signal Purity>95% Fail

UV Apex Spectra

GOETHE UNIVERSITÄT FRANKFURT AM MAIN

MSD Apex Spectra

RT:

3.297

Sample Name:

FGMM158_F17-18

Signal Name:

MSD1TIC

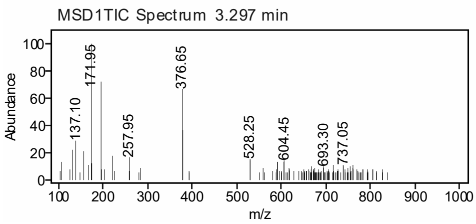

RT:

3.667

Sample Name:

FGMM158_F17-18

Signal Name:

MSD1TIC

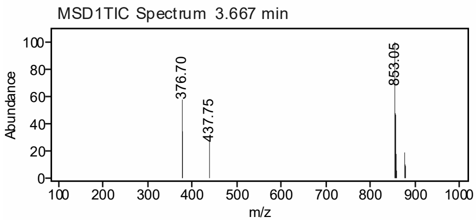

RT:

3.895

Sample Name:

FGMM158_F17-18

Signal Name:

MSD1TIC

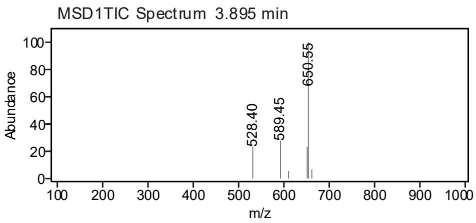

RT:

3.967

Sample Name:

FGMM158_F17-18

Signal Name:

MSD1TIC

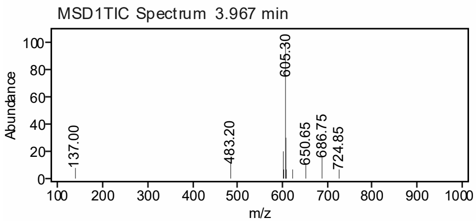

Compound S15

Sample Report

GOETHE UNIVERSITÄT FRANKFURT AM MAIN

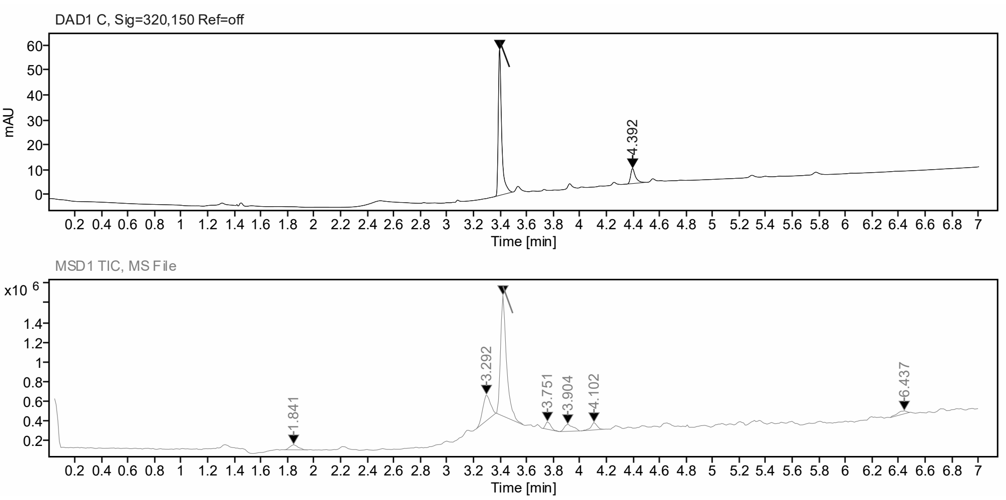

Sample Purity

Signal Description DAD1 C, Sig=320,150 Ref=off

Sample Name	Name	RT	Width	Area	Area%	Height
FGMM159_30min		3.392	0.024	103.0930	87.09	58.5004
FGMM159_30min		4.392	0.036	15.2796	12.91	6.0464

Max Area% 87.092

UV Signal Purity>95% Fail

UV Apex Spectra

RT:

3.392

Sample Name:

FGMM159_30min

Signal Name:

DAD1C

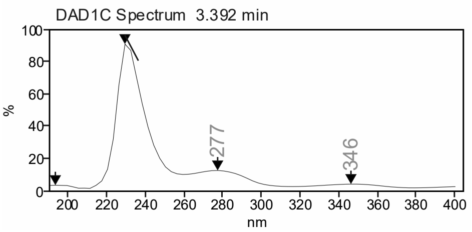

Sample Report

GOETHE UNIVERSITÄT FRANKFURT AM MAIN

RT:

3.751

Sample Name:

FGMM159_30min

Signal Name:

MSD1TIC

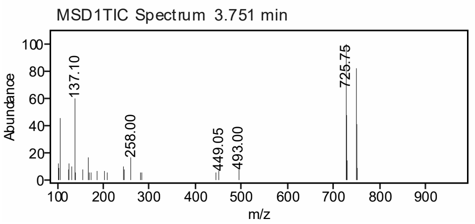

RT:

3.904

Sample Name:

FGMM159_30min

Signal Name:

MSD1TIC

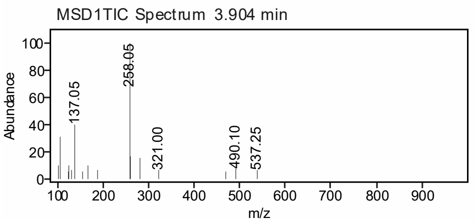

RT:

4.102

Sample Name:

FGMM159_30min

Signal Name:

MSD1TIC

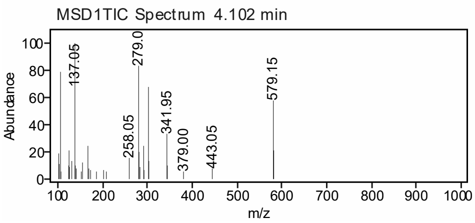

RT:

6.437

Sample Name:

FGMM159_30min

Signal Name:

MSD1TIC

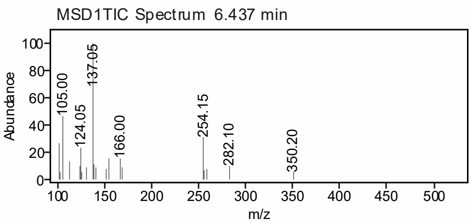

Compound S16

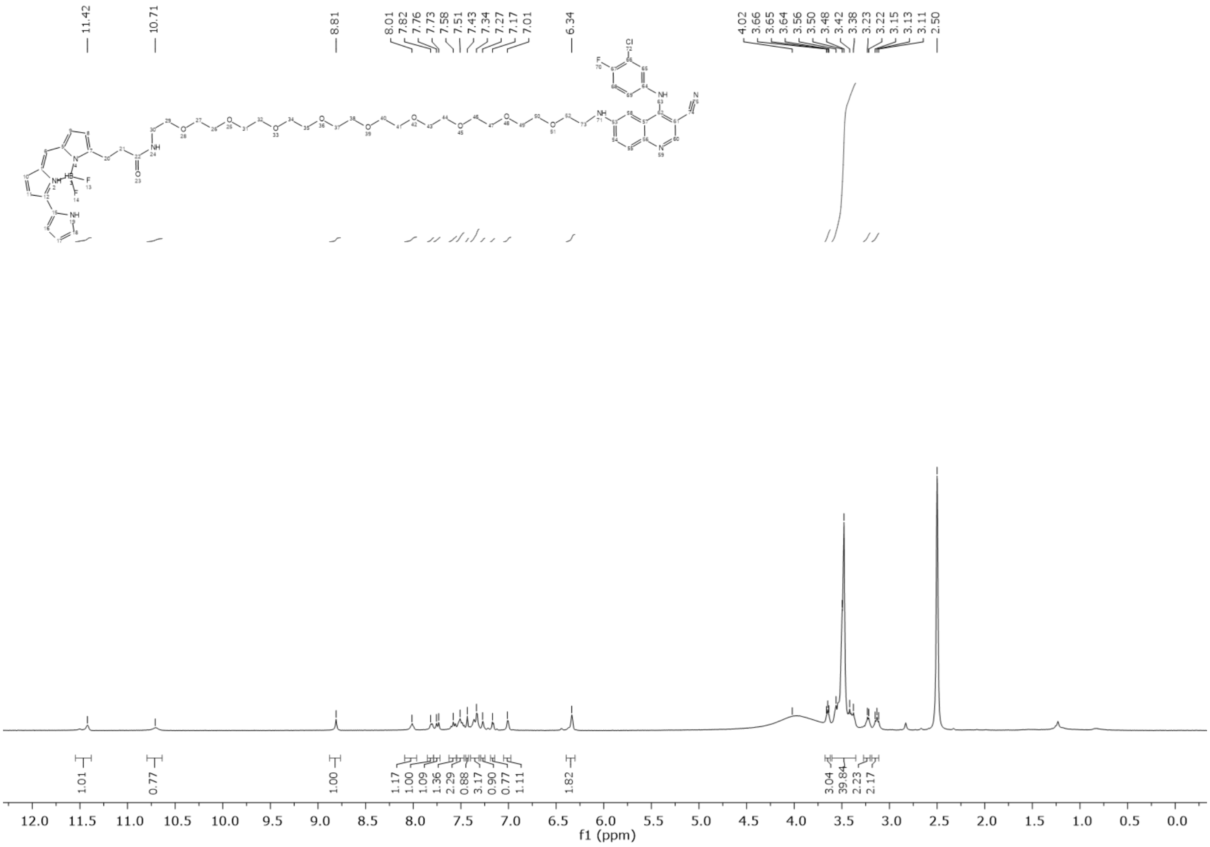

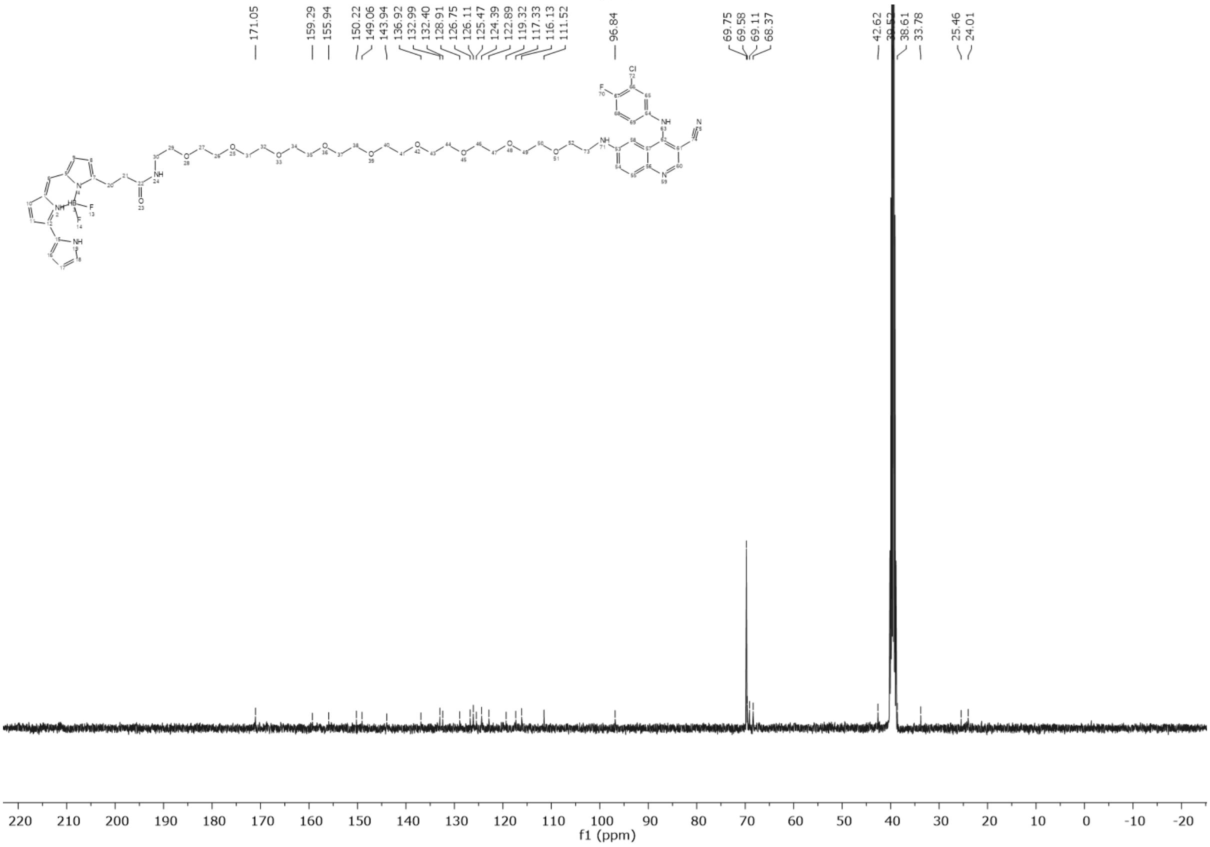

Sample Report

GOETHE UNIVERSITÄT FRANKFURT AM MAIN

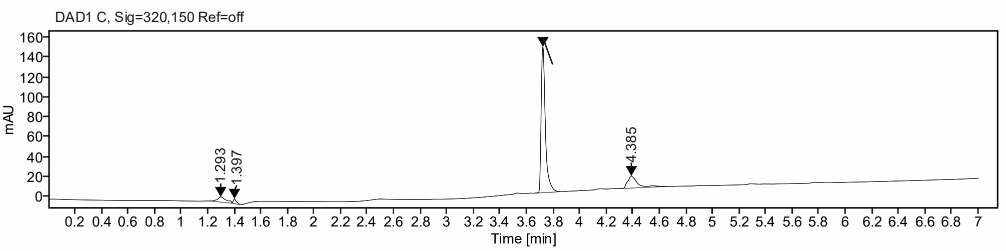

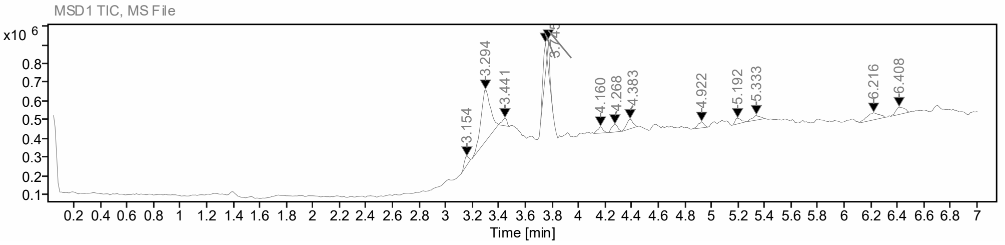

Sample Purity

Signal Description DAD1 C, Sig=320,150 Ref=off

Sample Name	Name	RT	Width	Area	Area%	Height
FGMM163_F12		1.293	0.058	24.0427	5.75	5.1380
FGMM163_F12		1.397	0.022	7.8877	1.89	4.7910
FGMM163_F12		3.719	0.032	323.5927	77.40	146.1619
FGMM163_F12		4.385	0.072	62.5453	14.96	12.0723

Max Area% 77.402

UV Signal Purity>95% Fail

UV Apex Spectra

RT:

1.293

Sample Name:

FGMM163_F12

Signal Name:

DAD1C

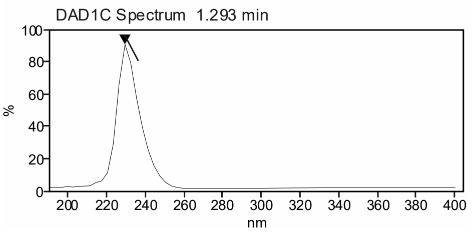

GOETHE UNIVERSITÄT FRANKFURT AM MAIN

RT:

3.294

Sample Name:

FGMM163_F12

Signal Name:

MSD1TIC

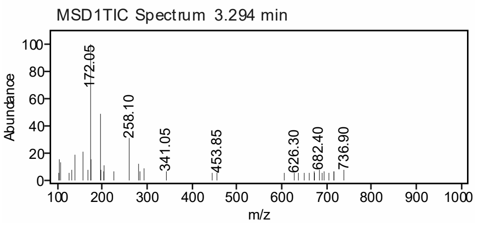

RT:

3.441

Sample Name:

FGMM163_F12

Signal Name:

MSD1TIC

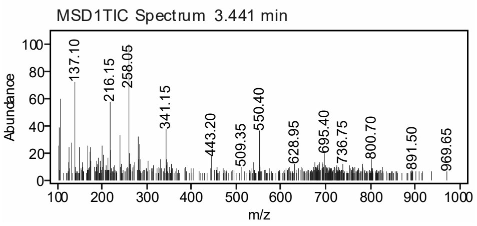

RT:

3.745

Sample Name:

FGMM163_F12

Signal Name:

MSD1TIC

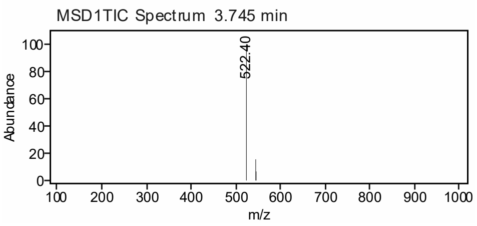

RT:

3.767

Sample Name:

FGMM163_F12

Signal Name:

MSD1TIC

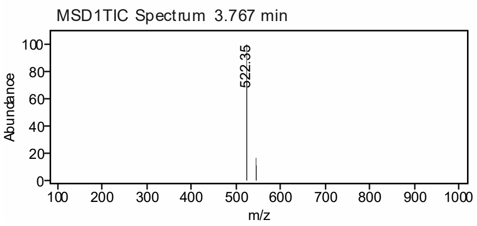

RT:

4.160

Sample Name:

FGMM163_F12

Signal Name:

MSD1TIC

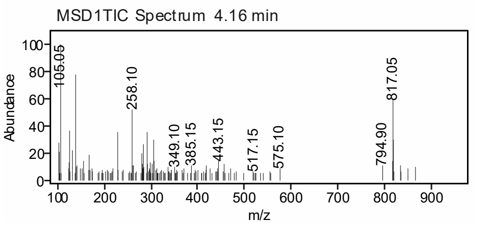

C:\User\...\2023\26.01.2023\FGMM163_E12 1/26/2023 11:26:01 AM FGMM163 mit HCCA gemessen.

FGMM163_E12 #1-19 RT: 0.01-0.74 AV: 19 NL: 1.58E7
T: FTMS + p MALDI Full ms [800.00-1200.00]

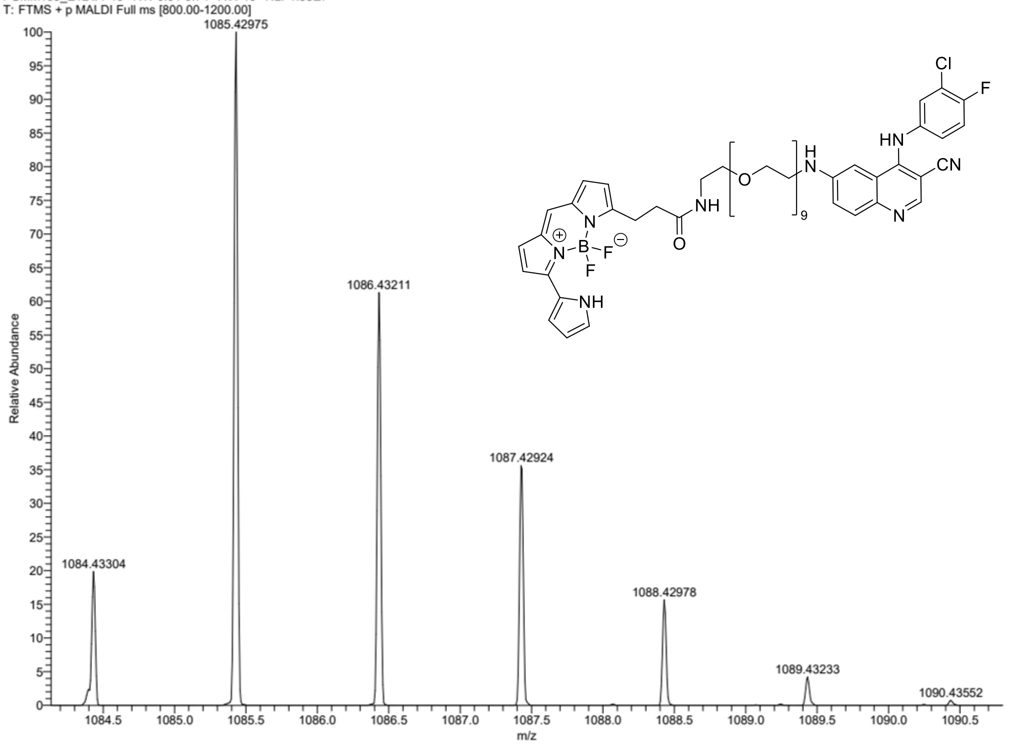

HPLC blank run

Sample Report

GOETHE UNIVERSITÄT FRANKFURT AM MAIN

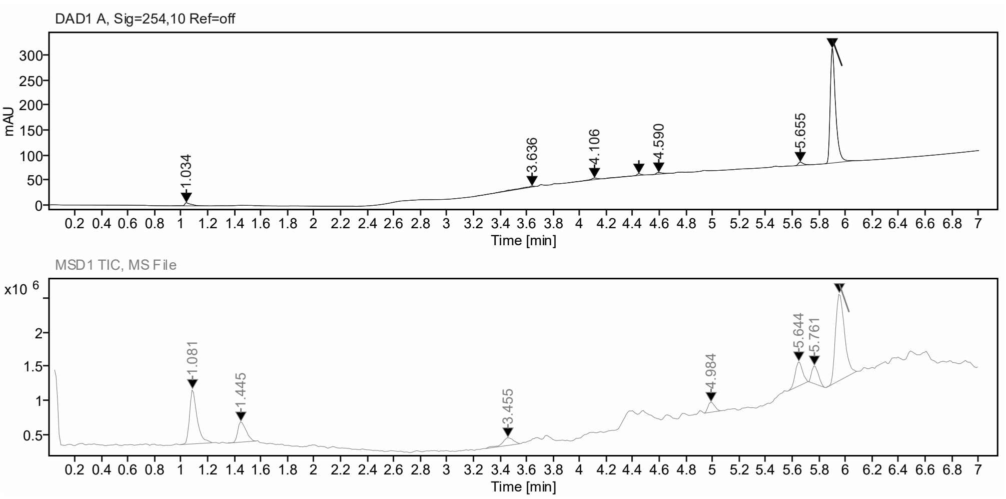

Sample Purity

Signal Description DAD1 A, Sig=254,10 Ref=off

Sample Name	Name	RT	Width	Area	Area%	Height
FGI445		1.034	0.049	20.8062	2.80	6.2196
FGI445		3.636	0.042	20.0277	2.70	1.9422
FGI445		4.106	0.063	12.5636	1.69	3.3002
FGI445		4.440	0.038	12.5296	1.69	3.4550
FGI445		4.590	0.100	10.5126	1.42	3.2430
FGI445		5.655	0.041	17.2492	2.32	6.7977
FGI445		5.896	0.041	648.7751	87.38	230.8066

Max Area% 87.381

UV Signal Purity>95% Fail

UV Apex Spectra

HPLC blank run + 5% DMSO

Sample Report

GOETHE UNIVERSITÄT FRANKFURT AM MAIN

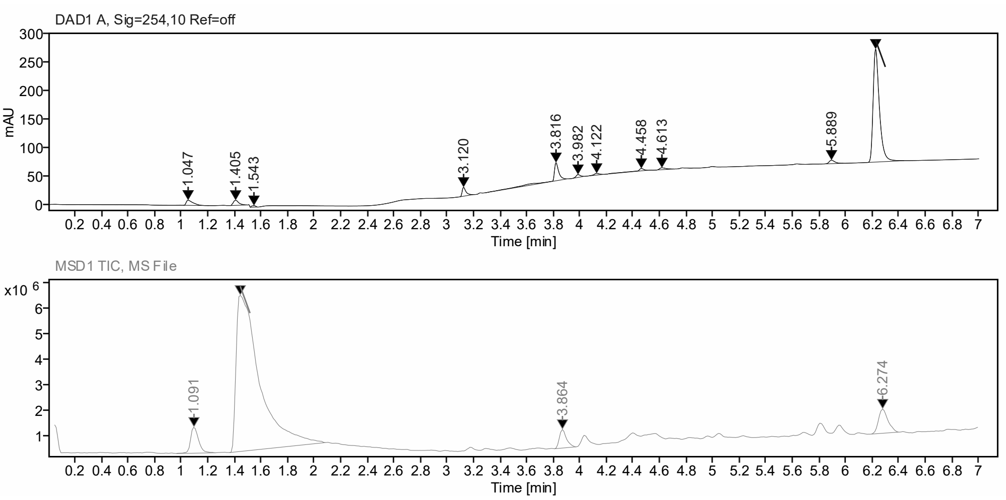

Sample Purity

Signal Description DAD1 A, Sig=254,10 Ref=off

Sample Name	Name	RT	Width	Area	Area%	Height
blank_5%DMSO		1.047	0.055	32.3043	3.61	8.9663
blank_5%DMSO		1.405	0.044	25.7533	2.87	8.7424
blank_5%DMSO		1.543	0.038	5.6328	0.63	2.6938
blank_5%DMSO		3.120	0.030	31.0623	3.47	15.4874
blank_5%DMSO		3.816	0.033	95.1996	10.63	31.5228
blank_5%DMSO		3.982	0.041	11.1314	1.24	4.9388
blank_5%DMSO		4.122	0.053	8.0933	0.90	2.9044
blank_5%DMSO		4.458	0.038	14.6536	1.64	4.0623
blank_5%DMSO		4.613	0.062	11.1464	1.24	3.2503
blank_5%DMSO		5.889	0.046	18.0571	2.02	6.1327
blank_5%DMSO		6.221	0.048	642.8242	71.76	196.8157

Max Area% 71.755

UV Signal Purity>95% Fail

UV Apex Spectra
